# Selective *ortho*-C–H Activation in Arenes without Functional Groups

**DOI:** 10.1021/jacs.2c04621

**Published:** 2022-06-21

**Authors:** Antony P. Y. Chan, Martin Jakoobi, Chenxu Wang, Robert T. O’Neill, Gülsevim S. S. Aydin, Nathan Halcovitch, Roman Boulatov, Alexey G. Sergeev

**Affiliations:** † Department of Chemistry, University of Liverpool, Crown Street, Liverpool L69 7ZD, U.K.; ‡ Department of Chemistry, Lancaster University, Bailrigg, Lancaster LA1 4YW, U.K.; § State Key Laboratory of Supramolecular Structure and Materials, College of Chemistry, Jilin University, Changchun 130012, P. R. China

## Abstract

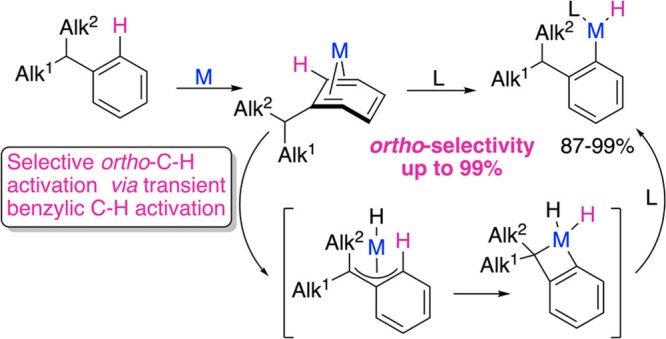

Aromatic C–H activation in alkylarenes is a key step for the synthesis of functionalized organic molecules from simple hydrocarbon precursors. Known examples of such C–H activations often yield mixtures of products resulting from activation of the least hindered C–H bonds. Here we report highly selective *ortho*-C–H activation in alkylarenes by simple iridium complexes. We demonstrate that the capacity of the alkyl substituent to override the typical preference of metal-mediated C–H activation for the least hindered aromatic C–H bonds results from transient insertion of iridium into the benzylic C–H bond. This enables fast iridium insertion into the *ortho*-C–H bond, followed by regeneration of the benzylic C–H bond by reductive elimination. Bulkier alkyl substituents increase the *ortho* selectivity. The described chemistry represents a conceptually new alternative to existing approaches for aromatic C–H bond activation.

Site-selective activation of aromatic C–H bonds is a challenging step that is important for the synthesis of a range of functionalized aromatic molecules, from pharmaceuticals to polymers.^1^ An established way to achieve high regioselectivity is to use arenes with heteroatom-containing functionalities that can direct the reagent attack at the *ortho*, *meta*, or even *para* position.^2^ Much more appealing is the activation of nonfunctionalized alkylarenes, which are readily available from petrochemical feedstocks; however, such activation remains challenging because alkyl groups have limited directing capacity, which leads to mixtures of products.^3^ The exceptions are symmetrical dialkylarenes, in which functionalization occurs at the least sterically hindered position,^4^ and a few monoalkylarenes, in which selective activation of *meta*-C–H^5^ and *para*-C–H bonds has recently been reported.^6^

Arenes without functional groups are generally modified by electrophilic or transition metal species, yet the yields of *ortho*-substituted products are less than 67% (Figure 1). Electrophilic functionalization usually yields mixtures of *ortho*- and *para*-substituted products because the regioselectivity is controlled by electronic factors, resulting in nearly isoenergetic *ortho*- and *para*-arenium-cation-like transition states and less stable *meta* transition states (Figure 1A). As a result, the *ortho*/*para* selectivity does not exceed the statistical ratio of 2:1.^7^ In contrast, metal-mediated C–H activation in alkylarenes typically yields mixtures of the *meta*- and *para*-substituted products, with the typical ratios of 2:1^4^ reflecting the steric accessibility of C–H bonds to the metal center. Few such C–H functionalizations yield more than traces of *ortho* isomers,^4^ and in no case does the *ortho* regioselectivity exceed 58%.^8^

Here we report an approach for regioselective activation of *ortho*-C–H bonds in alkylarenes using simple iridium complexes (Figure 1C). The high regioselectivity results from an alkyl substituent acting as an efficient directing group that binds the metal via initial benzylic C–H activation, which triggers subsequent oxidative addition of an *ortho*-C–H bond, reformation of the benzylic C–H bond, and release of the *o*-alkylaryl metal species. The key to enabling this approach was the use of rare iridium complexes, Cp*Ir(η^4^-alkylarene), which bear a nonplanar, “spring-loaded” alkylarene ligand with enhanced reactivity.

**Figure 1:**
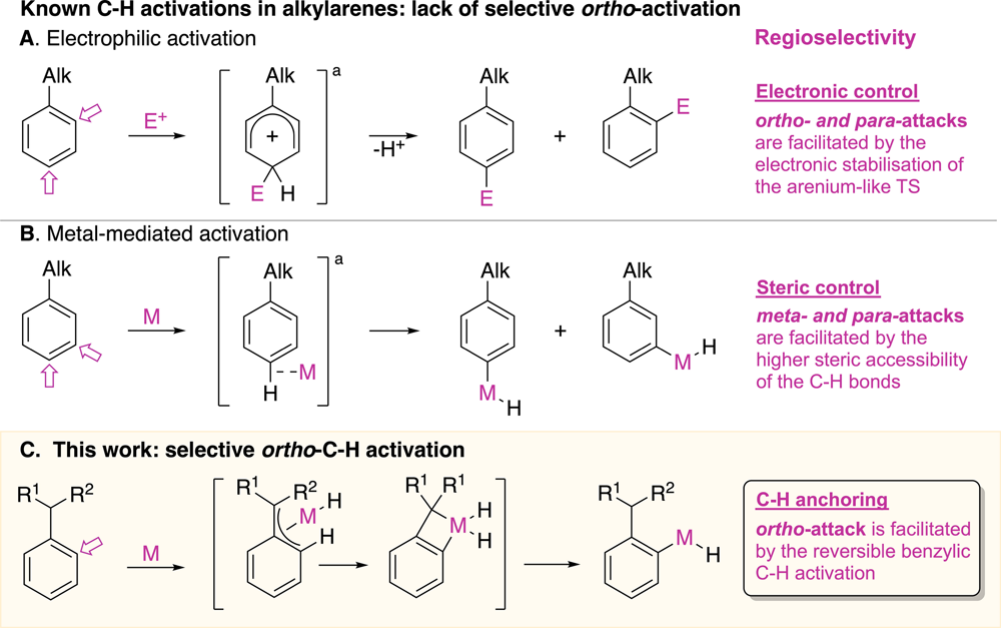
C–H activation of alkylarenes: (A) electrophilic aromatic substitution; (B) metal-mediated C–H activation; (C) suggested approach for selective iridium-mediated *ortho*-C–H activation. ^a^For brevity, only *para* isomers of key intermediates are shown.

We recently demonstrated that Cp*Ir(η^4^-methylarene) complexes promote selective benzylic C–H activation of the methylarene ligand in the presence of a phosphine ligand.^9^Our attempt to extend this reactivity to primary and secondary alkylarenes led to an unexpected switch in selectivity and formation of *ortho*-C–H activation products (Figure 2). The reaction of isopropylbenzene complex **1a** with PMe_3_ in *n*-hexane at 100 °C yielded the product of oxidative addition of an *ortho*-C–H bond, Ir aryl hydride complex **2a**, in 99% yield (Figure 2A). The use of a larger ligand, PPh_3_, decreased the yield of the *ortho*-C–H activation product **2a-ph** (67%), and the use of no ligand led to a complex mixture of products. Crystal structures of the C–H activation products **2a-ph** and **2a** as a hydride and bromide species are shown in Figures 2B and S7.

**Figure 2:**
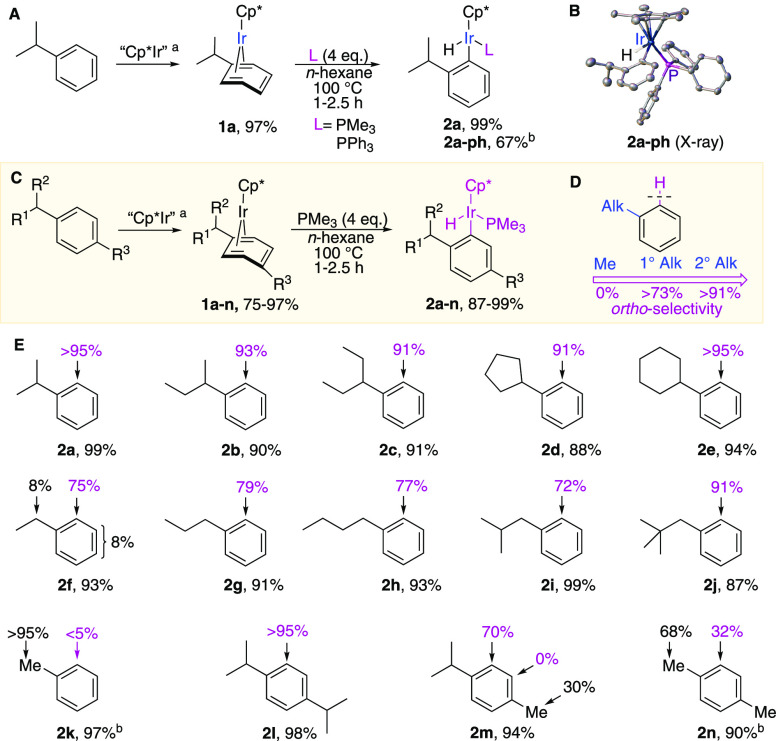
Scope and selectivity of iridium-mediated oxidative addition of *ortho*-C–H bonds in alkylarenes: (A) selective *ortho*-C–H activation in isopropylbenzene via initial η^4^-arene coordination to Cp*Ir and thermolysis of the resulting complex **1a** in the presence of PMe_3_ or PPh_3_; (B) crystal structure of **2a-ph**; (C) *ortho*-C–H activation of mono- and dialkylarenes in Cp*Ir complexes; (D) relative order of *ortho*-C–H selectivity; (E) scope of *ortho*-C–H activation of alkylarene ligands in complexes **1a**–**n**. Numbers under the arene structures are the total isolated yields of all C–H activation products. Numbers above the arene structures are the *ortho* selectivities determined by integration of the hydride signals in the ^1^H NMR spectra. ^a^Conditions: 8 equiv. of arene, 1 equiv. of [Cp*IrCl_2_]_2_, 4 equiv. of AgBF_4_, acetone, 24 °C, 16 h, then 2 equiv. of Cp_2_Co, benzene, 24 °C, 1 h. ^b^See ref (9).

We explored how the selectivity of *ortho*-C–H oxidative addition depends on the identity of the alkyl substituent on the arene ring by heating alkylarene iridium complexes **1a**–**n** with PMe_3_ as an added ligand (Figure 2C). These complexes are accessible from alkylarenes in 75–97% yield via a simple two-step procedure (see the Supporting Information). C–H activation of alkylarene ligands in **1a**–**n** led to high yields of iridium hydrides **2a**–**n** (87–99%) regardless of the identity of the alkyl substituent (Figure 2C). The *ortho* selectivity, however, was the highest with larger alkyl groups (Figure 2D). As shown in Figure 2E, arenes with secondary alkyls (*i*-Pr, *s*-Bu, 3-Pent, *c*-Pent, *c*-Hex) underwent *ortho*-C–H activation with ≥91% selectivity (**2a**–**e**), while arenes with primary alkyls (Et, *n*-Pr, *n*-Bu, *i*-Bu) gave lower *ortho* selectivities of 72–79% (**2f–i**). An exception was the bulkiest primary alkylarene in the test, neopentylbenzene, which yielded C–H activation product **2j** with 91% *ortho* selectivity. The arene with the smallest alkyl substituent, methylbenzene, gave no *ortho*-C–H activation product but instead gave benzyl hydride complex **2k**.^9^ The observed order of *ortho* regioselectivity, *sec*-alkyl > *n*-alkyl ≫ methyl (Figure 2D), is opposite to that of classical electrophilic substitution^7a,7b^ and contrasts with that of known oxidative additions of C–H bonds in alkylarenes, which favor *meta* and *para* but not *ortho* products.^10^ The same counterintuitive trend holds for the C–H activation of *para*-substituted dialkylarene ligands (Figure 2C): for example, in *p*-isopropylmethylbenzene, aromatic C–H activation occurs exclusively next to the isopropyl substituent and not next to the methyl substitutent (**2m**). Bulkier *p*-diisopropylbenzene gave *ortho* metalation product **2l** exclusively, while smaller *p*-dimethylbenzene yielded a 32:68 mixture of *ortho* and benzyl C–H metalation products **2n**.^9^

To rationalize the observed counterintuitive regioselectivity, we probed the reaction mechanism by monitoring the model C–H activation in *p*-diisopropylbenzene complex **1l** in the presence of PMe_3_ in cyclohexane-*d*_12_ (Figure 3A). Complex **1l** was chosen because it exists as a single isomer, which improves the accuracy of kinetic measurements by ^1^H NMR spectroscopy. The reaction is first-order in **1l** and zeroth-order in the phosphine (Figure 3A). The initial reaction rates for separate thermolyses of **1l** and its analogue with a fully deuterated arene ring, **1l**-**d**_**4**_, were within the experimental uncertainty (Figure 3B), suggesting that *ortho*-C–H bonds do not participate in the rate-determining step. Contrary to what was expected, the *ortho*-C–D activation in **1l**-**d**_**4**_ in *n*-hexane did not lead to deuterium incorporation in the hydride ligand of **2l**-**d**_**4**_. Instead, deuterium appeared in the methyl groups of the *o*-isopropyl group (Figure 3C). This may result from the intramolecular H/D redistribution between the deuteride ligand and the methyl groups. Intermolecular H/D scrambling between the hydride (deuteride) ligand and the solvent was excluded because heating **1l** in cyclohexane-*d*_12_ did not lead to incorporation of D into **2l** (Figure 3A). The observed H/D redistribution in **2l**-**d**_**4**_ must have resulted from H/D scrambling in reaction intermediates, not in the starting complex **1l**-**d**_**4**_, in which no H/D redistribution was observed over the course of the reaction.

**Figure 3:**
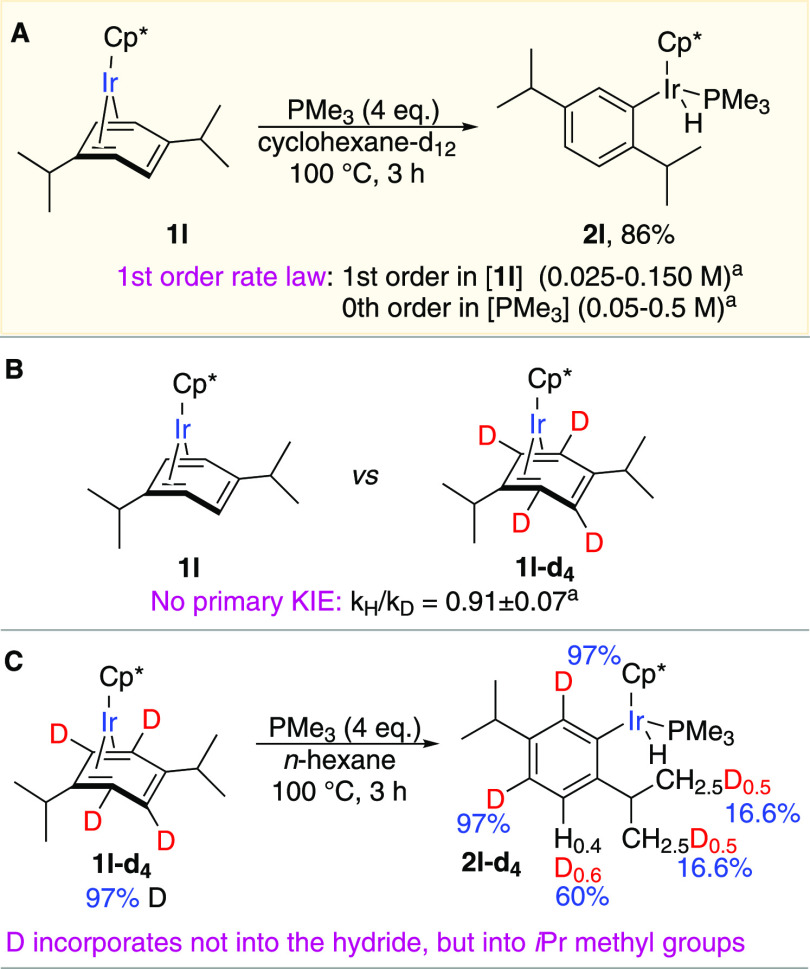
Mechanistic experiments using model *ortho*-C–H activation in **1l**: (A) model reaction and rate law measurement upon thermolysis of **1l** in cyclohexane-*d*_12_ in the presence of PMe_3_; (B) H/D kinetic isotope effect measured for separate thermolyses of **1l** and **1l**-**d**_**4**_ in cyclohexane-*d*_12_ at <15% conversion; (C) H/D scrambling upon thermolysis of **1l**-**d**_**4**_ in *n*-hexane. The values in blue show the D contents at the specified positions. ^a^Measurements were conducted at 75 °C.

We identified a mechanism that agrees with the experimental observations for the *ortho*-C–H activation in **1l** by computing a range of reaction paths using the M06-2X functional (Figures S8–S11). The lowest-energy path (Figure 4A, path 1) starts with sliding of the arene ligand to give η^2^-arene intermediate **3**, which then oxidatively adds the benzylic C–H bond of the adjacent isopropyl group. The resulting η^3^-benzyl hydride complex **4** isomerizes into metallacycle **5** by insertion of iridium into the adjacent *ortho*-C–H bond. Quick elimination of the benzylic C–H bond in **5** forms coordinatively unsaturated aryl hydride **6**, which binds PMe_3_ to afford the observed product **2l**. The similar energies of the four least-stable transition states of the main mechanism (21.2–23.3 kcal/mol) preclude unambiguous identification of the rate-determining step.^11^ However, the lack of a primary kinetic isotope effect (KIE) in **1l**-**d**_**4**_ vs **1l** suggests that oxidative addition of an aromatic C–H bond in **4** is not the rate-determining step.

**Figure 4:**
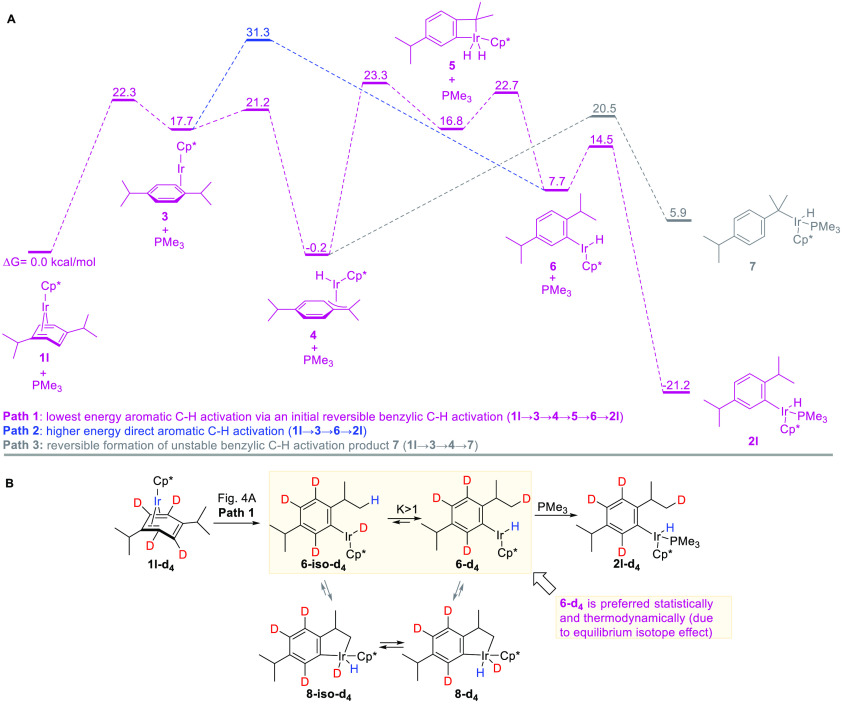
Mechanistic insight into C–H activation in **1l**. (A) Calculated mechanisms for aromatic and benzylic oxidative addition of *ortho*-C–H and benzylic C–H bonds in **1l**. All of the calculations were done with the M06-2X functional using the def2SVP basis set for geometry optimizations and frequency calculations and the def2TZVPP basis set for single-point energy calculations. All free energies are relative to 1 mol of **1l** and 1 mol of PMe_3_ at 75 °C in cyclohexane (represented in computations by the conductor-like polarizable continuum model). (B) Proposed mechanism for the observed intramolecular H/D scrambling in **1l**-**d**_**4**_.

This mechanism revealed that the observed selective *ortho*-C–H activation in iridium η^4^-arene complexes results from the favorable combination of the kinetic and thermodynamic factors that promote site-selective aromatic C–H activation and disfavor competing benzylic C–H activation. The *ortho*-C–H activation is kinetically favored because of the specific directing effect of an alkyl group (Figure 4A). The coordinated alkylarene substrate undergoes the initial benzylic C–H activation of the alkyl group that anchors the metal center next to an *ortho* position and thus promotes the oxidative addition of the *ortho*-C–H bond followed by the facile formation of the final product via fleeting iridacyclobutane dihydride intermediate **5**. This strained and bulky metallacycle has a high free energy (16.8 kcal/mol above the starting complex **1l**) and high reactivity (a barrier of 5.9 kcal/mol for the conversion to **6**), which precluded the detection of the intermediate. However, more stable analogues of **5** with less bulky ancillary and hydrocarbyl ligands^12^ and their proposed intermediacy in a related isomerization of *o*-methylaryl to benzyl complexes were reported.^13^ As can be seen in Figure 4A, the *ortho*-C–H activation via the sequential oxidative addition of two C–H bonds indeed requires traversing much lower barriers (≤23.3 kcal/mol, path 1) than the standard direct *ortho*-C–H oxidative addition in **3** (31.3 kcal/mol, path 2).

The *ortho*-C–H activation in **1l** (Figure 4, path 1) is thermodynamically preferable to the competing benzylic C–H activation (Figure 4, path 3) that occurs via the same intermediate **4** and gives exergonic benzyl complex **7**. In contrast, C–H activation in the less bulky iridium methylarene complexes selectively yields benzylic products, which are kinetically and thermodynamically more accessible than the corresponding *o*-methylaryl products as we reported previously.^14^ This comparison of the C–H activation in secondary alkylarene and methylarene iridium complexes suggests that the higher degree of substitution at the benzylic carbon destabilizes the benzyl complex versus the aryl complex and therefore promotes aromatic *ortho*-C–H activation at the expense of benzylic C–H activation (Figure 2C,D).

This reactivity contrasts with the established reactivity of metal complexes toward alkylarenes, which favors the activation of *meta*- and *para*-C–H bonds over the activation of benzylic and *ortho*-C–H bonds.^10a,10b,15^ The reported double C–H activation mechanism overcomes this limitation: the reversible benzylic C–H activation anchors the metal next to the *ortho* positions and lessens the barrier for the following *ortho*-C–H oxidative addition (Figure 4A).

Finally, the mechanism may explain the remarkable H/D redistribution upon the *ortho*-C–D oxidative addition in **1l**-**d**_**4**_ (Figure 3C) that yields hydride, not deuteride, product **2l**-**d**_**4**_.^16^ Complex **2l**-**d**_**4**_ may result from equilibration of the initial deuteride intermediate **6-iso-d**_**4**_ with hydride **6-d**_**4**_ followed by coordination of PMe_3_. Although the exact mechanism for this equilibration has yet to be identified, our preliminary calculations suggest that it may occur via five-membered metallacycles **8-iso-d**_**4**_ and **8-d**_**4**_ and that these metallacycles can be accessed from **1l**-**d**_**4**_ only via **6-iso-d**_**4**_ (Figures S8–S15). The equilibrium between **6-iso-d**_**4**_ and **6-d**_**4**_ lies toward hydride **6-d**_**4**_, which is favored entropically because of the 6:1 H:D ratio and also enthalpically because of the zero-point-energy effect, i.e., the preferred location of deuterium in the highest-frequency oscillator,^17^ which is the C(sp^3^)–D bond, not the metal–D bond. A similar explanation was proposed by Jones and Feher for the 2.7-fold higher stability of the related deuteride complex Cp*Rh(PMe_3_)(C_6_D_4_H)D versus its hydride isomer Cp*Rh(PMe_3_)(C_6_D_5_)H in an equilibrium mixture.^18^

In summary, we have presented a conceptually new method for controlling the site selectivity of C–H activation in arenes without directing groups. This method relies on the use of simple iridium(I) complexes that enable highly selective *ortho*-C–H activation in primary and secondary alkylarenes without any functional groups. Key to this selectivity is the transient reversible benzylic C–H activation that brings the metal center into close proximity to an *ortho*-C–H bond and enables smooth metal insertion into the most sterically hindered position of the aromatic ring. This C–H activation occurs in a highly reactive Cp*Ir(η^2^-alkylarene) intermediate generated by sliding of the arene ligand in a Cp*Ir(η^4^-alkylarene) precursor. Translation of this stoichiometric reactivity into catalytic *ortho*-C–H functionalizations may open new avenues for the selective synthesis of value-added chemicals from unactivated aromatic hydrocarbons. Enabling such synthetic applications will require further improvement of the scope and selectivity of the process and the design of a catalytic cycle that involves the straightforward formation of the key unsaturated η^2^-alkylarene iridium intermediate from the free arene and regeneration of this intermediate after C–H functionalization. Work on addressing these challenges is ongoing in our laboratory.

## Supporting Information Available

Figures S1–S15, Tables S1–S4, experimental procedures for synthetic and mechanistic experiments, NMR spectra for all new compounds, DFT data (*xyz* coordinates) for all calculated structures, and crystallographic data for **2a-ph** and **2a-br** (PDF)

## Abbreviations

Cp*: pentamethylcyclopentadienyl
TS: transition state
