# Supplementary material for: Selective ortho-C–H Activation in Arenes without Functional Groups
Source: J Am Chem Soc. 2022 Jun 21;144(26):11564–8. doi: 10.1021/jacs.2c04621 (PMC9348813; doi:10.1021/jacs.2c04621)
Supplement: Supplementary file 1 — ja2c04621_si_001.pdf [file ja2c04621_si_001.pdf]

Selective *ortho*-C-H activation in arenes without functional groups

Antony P. Y. Chan[1], Martin Jakoobi[1], Chenxu Wang[1], Robert T. O'Neill[1], Gülsevim S. S. Aydin[1], Nathan Halcovitch[2], Roman Boulatov[1,3], Alexey G. Sergeev[1*]

[1] Department of Chemistry, University of Liverpool; Crown Street, Liverpool, L69 7ZD, UK

[2] Department of Chemistry, Lancaster University; Bailrigg, Lancaster, LA1 4YW, UK

[3] State Key Laboratory of Supramolecular Structure and Materials, College of Chemistry, Jilin University, Changchun,130012, P. R. of China

Table of Contents:

1. General experimental details S2
2. Preparation of deuterated arenes S3
3. Preparation of η^6-arene complexes S5
4. Preparation of η^4-arene complexes S11
5. Procedure for *ortho*-C-H activation of η^4-arene complexes S22
6. Procedure for bromide-hydride exchange in **2a** S32
7. Procedures for kinetic and isotope-labelling studies S33
8. Crystallography data S43
9. DFT calculations data S45
10. NMR spectra S57
11. References S129

[1]Department of Chemistry, University of Liverpool; Crown Street, Liverpool, L69 7ZD, UK. [2]Department of Chemistry, Lancaster University; Bailrigg, Lancaster, LA1 4YW, UK. [3]State Key Laboratory of Supramolecular Structure and Materials, College of Chemistry, Jilin University, Changchun,130012, P. R. of China.
Email: sergeev@liverpool.ac.uk

1. General experimental details

All air-sensitive manipulations were conducted under an inert atmosphere in an argon-filled Innovative Technology glovebox or by standard Schlenk technique under argon. All glassware was heated in an oven at 120 °C and cooled under vacuum prior to use.

NMR spectra were acquired on a Bruker Avance I (400 MHz) and Bruker Avance III HD (500 MHz) instruments at ambient temperature. Chemical shifts (δ) are reported in ppm and ^{1}H NMR spectra are reported relative to the corresponding signals of residual protons in the deuterated solvents: C_6D_6 δ 7.16 ppm, $(CD_3)_2CO$ δ 2.05 ppm, CF_3CO_2D (d-TFA) δ 11.50 ppm, C_6D_{12} δ 1.38 ppm. ^{13}C NMR spectra are reported relative to the following signals of deuterated solvents: C_6D_6: δ 128.06 ppm, $(CD_3)_2CO$ δ 206.26 ppm, CF_3CO_2D (d-TFA) δ 116.60 (q) ppm.

The splitting patterns are designated as follows: s (singlet), br. s (broad singlet), d (doublet), dd (doublet of doublets), dt (doublet of triplets), dq (doublet of quartets), d sept (doublet of septets), ddd (doublet of doublet of doublets), t (triplet), app. t (apparent triplet), tt (triplet of triplets), q (quartet), app. quin (apparent quintet), app. sept (apparent septet) and m (multiplet). New compounds were assigned using HSQC, HMBC and COSY experiments where appropriate or by comparison with known analogues.

Elemental analyses were performed by the Microanalysis Laboratory of the Department of Chemistry, University of Liverpool on a Thermo Flash EA 112 Series instrument.

Mass spectrometry analyses were conducted by the EPSRC UK National Mass Spectrometry Facility at Swansea University and by the Microanalysis Laboratory of the Department of Chemistry, University of Liverpool. Samples containing [Cp*Ir(η^6-arene)][BF_4]$_2$ complexes were sent to Swansea University in vials as solids, whilst [Cp*Ir(η^4-arene)] and [Cp*Ir(PMe_3)H(Ar)] complexes were sealed under argon in vials with Teflon-lined screw caps. High resolution mass spectra (HRMS) were recorded in the positive mode. Electrospray (ESI) and nano-Electrospray (nanoESI) ionization spectra were recorded on the OrbitrapXL; Atmospheric Pressure Ionisation spectra (APCI) were recorded on the Xevo G2S using the Atmospheric Solids Analysis Probe (ASAP).

n-Hexane was distilled from sodium benzophenone ketyl still and stored under argon in the glovebox. Anhydrous benzene was purchased from Alfa Aesar and stored under argon in the glovebox. Acetone was purchased from Fisher Scientific and used without further purification.

[Cp*IrCl_2]$_2$[1], cyclopentylbenzene[2], **2k**[3] and **2n**[3] were prepared according to the literature. All other reagents were supplied commercially and used without further purification.

2. Preparation of deuterated arenes

1,4-Diisopropylbenzene-d$_4$

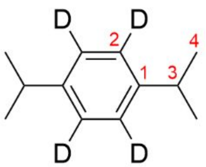

The product was prepared according to the literature procedure[4].

In a Schlenk bomb was added $MoCl_5$ (13.7 mg, 0.05 mmol), C_6D_6 (1.8 mL) and 1,4-diisopropylbenzene (0.81 g, 5.0 mmol) under argon. The vessel was sealed, and the suspension was stirred overnight at room temperature. The resultant solution was quenched with 2M HCl (8.0 mL) and the organic phase separated. The aqueous layer was extracted using Et_2O (3 × 5.0 mL) and the combined organic fractions were washed with H_2O (5.0 mL), sat. $NaHCO_3$ solution (5.0 mL) and brine (5.0 mL). $MgSO_4$ was used to dry the solution and the Et_2O was removed *in vacuo* to afford the crude product as a yellow oil. The deuteration process is then repeated two more times.

Following the third deuteration cycle, the crude product was then distilled (72 °C, 8-11 mbar) to afford the product (31 %, 0.26 g, 1.6 mmol) as a colourless oil with >97 % deuteration at the aromatic positions.

^{1}H NMR (500 MHz, $CDCl_3$): δ 2.89 (app. sept., J = 6.9 Hz, 2H, *H3*), 1.25 (d, J = 6.9 Hz, 12H, *H4*).

^{13}C NMR (126 MHz, $CDCl_3$): δ 146.13 (*C1*), 126.01 (t, *C2*), 33.69 (*C3*), 24.20 (*C4*).

HRMS (ESI+): *m/z* calculated for $[C_{12}H_{14}D_4]^+$ 166.1660, found 166.1646.

1,4-Diisopropylbenzene-d$_2$

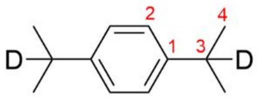

The product was prepared according to the literature procedure[5].

In a Schlenk bomb was added dry NaH (240.0 mg, 10.0 mmol), DMSO-d$_6$ (4.0 mL) and 1,4-diisopropylbenzene (3.26 g, 20.1 mmol) under argon. The vessel was sealed, and the mixture stirred for 48 h at 100 °C. Following cooling, D_2O (4.0 mL) was added, and the organic layer was separated. Diethyl ether (3 × 5.0 mL) was used to wash the red aqueous layer and the combined organic fractions were washed with deionised water (5.0 mL) and dried using K_2CO_3. Removal of Et_2O *in vacuo* affords the crude product as a pale-yellow oil. The deuteration process is then repeated two more times.

Following the third deuteration cycle, the crude product was distilled (78 °C, 6-8 mbar) to afford the product (60 %, 1.97 g, 12.0 mmol) as a colourless oil with 91 % deuteration at the benzylic positions.

N.B. Minor signals relating to non-deuterated arene present in ^{13}C NMR [δ 33.79 (*C3*) and 24.21 (*C4*)]

^{1}H NMR (500 MHz, $CDCl_3$): δ 7.16 (s, 4H, *H2*), 1.24 (br. s, 12H, *H4*).

^{13}C NMR (126 MHz, $CDCl_3$): δ 146.28 (*C1*), 126.42 (*C2*), 33.36 (t, *C3*), 24.10 (*C4*).

HRMS (ESI+): *m/z* calculated for $[C_{12}H_{16}D_2]^+$ 164.1534, found 164.1530.

3. Preparation of η^6-arene complexes

General procedure for the preparation of [Cp*Ir(η^6-arene)][BF_4]$_2$ (modified literature procedure)[6]

[Cp*$IrCl_2$]$_2$ (50.0 mg, 0.063 mmol) and $AgBF_4$ (49.0 mg, 0.252 mmol) was suspended in acetone (1 mL) and stirred for 1 h. The resultant yellow suspension was filtered and the precipitate washed with acetone until the washings became colourless. The combined filtrate and washings were reduced to *ca.* 1 mL and 8 eq. of the appropriate arene was added. The reaction mixture was stirred overnight at room temperature before evaporating to dryness. The residue was then re-dissolved in trifluoroacetic acid and passed through glass wool. Diethyl ether (*ca.* 10 mL) was added to precipitate the product and the solvents were decanted. The solid is then washed with additional amount of Et_2O and subsequently dried under vacuum at 50 °C overnight to afford the product as a white powder. Additional product can be collected from the decanted solution and washings.

[Cp*Ir(η^6-ethylbenzene)][BF_4]$_2$

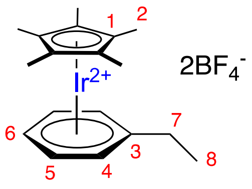

The complex was prepared according to the general procedure using ethylbenzene (0.07 mL, 0.571 mmol) to afford [Cp*Ir(η^6-ethylbenzene)][BF_4]$_2$ as a white solid (99%, 76.0 mg, 0.125 mmol). ^{1}H NMR spectrum of the product was similar to that of previously reported perchlorate salt [7].

^{1}H NMR (400 MHz, d-TFA): δ 7.55 (app. t, J = 6.0 Hz, 2H, *H5*), 7.51-7.47 (m, 3H, *H4*+*H6*), 2.99 (q, J = 7.5 Hz, 2H, *H7*), 2.56 (s, 15H, *H2*), 1.61 (t, J = 7.5 Hz, 3H, *H8*).

^{13}C NMR (100 MHz, d-TFA): δ 122.81 (*C3*), 108.71 (*C1*), 100.85 (*C4*/*C5*), 99.77 (*C4*/*C5*), 99.69 (*C6*), 28.04 (*C7*), 15.10 (*C8*), 10.79 (*C2*).

$C_{18}H_{25}B_2F_8Ir$ requires C 35.60, H 4.15. Found: C 35.23, H 4.02%.

HRMS (nanoESI+): *m/z* calculated for [$^{191}IrC_{18}H_{25}$]$^{2+}$ 216.0781, found 216.0773.

[Cp*Ir(η^6-isopropylbenzene)][BF_4]$_2$

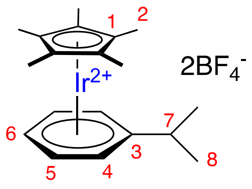

The complex was prepared according to the general procedure using isopropylbenzene (0.07 mL, 0.502 mmol) to afford [Cp*Ir(η^6-isopropylbenzene)][BF_4]$_2$ as a white solid (99%, 76.8 mg, 0.124 mmol).

^{1}H NMR (400 MHz, d-TFA): δ 7.56-7.53 (m, 5H, *H4-H6*), 3.24 (app. sept., J = 6.8 Hz, 1H, *H7*), 2.57 (s, 15H, *H2*), 1.62 (d, J = 6.8 Hz, 6H, *H8*).

^{13}C NMR (100 MHz, d-TFA): δ 128.51 (*C3*), 108.78 (*C1*), 100.51 (*C4/C5*), 100.03 (*C6*), 97.83 (*C4/C5*), 33.79 (*C7*), 22.85 (*C8*), 10.98 (*C2*).

$C_{19}H_{27}B_2F_8Ir$ requires C 36.73, H 4.38. Found: C 36.48, H 4.27%.

HRMS (nanoESI+): *m/z* calculated for $[^{191}IrC_{19}H_{27}]^{2+}$ 223.0859, found 223.0853.

[Cp* Ir(η^6-sec-butylbenzene)][BF$_4$]$_2$

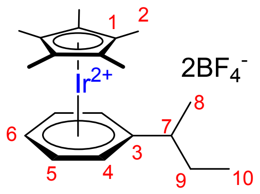

The complex was prepared according to the general procedure using $[Cp^*IrCl_2]_2$ (70.5 mg, 0.088 mmol), $AgBF_4$ (68.9 mg, 0.353 mmol) and sec-butylbenzene (0.110 mL, 0.703 mmol) to afford [Cp*Ir(η^6-*sec*-butylbenzene)][BF$_4$]$_2$ as a white solid (100%, 111.9 mg, 0.176 mmol).

^{1}H NMR [500 MHz, $(CD_3)_2CO$]: δ 7.72-7.56 (m, 5H, *H4-6*), 3.07 (m, 1H, *H7*), 2.48 (s, 15H, *H2*), 1.88-1.71 (m, 2H, *H9*), 1.45 (d, J = 6.9 Hz, 3H, *H8*), 1.02 (t, J = 7.4 Hz, 6H, *H10*).

^{13}C NMR [126 MHz, $(CD_3)_2CO$]: δ 125.56 (*C3*), 106.80 (*C1*), 99.27 (*C4*), 99.11 (*C5*), 99.08 (*C5*), 97.48 (*C6*), 96.39 (*C4*), 37.93 (*C7*), 31.16 (*C9*), 17.83 (*C8*), 11.46 (*C10*) 10.20 (*C2*).

HRMS (nanoESI+): *m/z* calculated for $[^{191}IrC_{20}H_{29}]^{2+}$ 230.0932, found 230.0934.

[Cp* Ir(η^6-3-pentylbenzene)][BF$_4$]$_2$

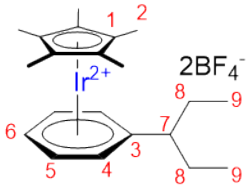

The complex was prepared according to the general procedure using $[Cp^*IrCl_2]_2$ (70.3 mg, 0.088 mmol), $AgBF_4$ (68.7 mg, 0.353 mmol) and 3-pentylbenzene (0.120 mL, 0.695 mmol) to afford [Cp*Ir(η^6-3-pentylbenzene)][BF$_4$]$_2$ as a white solid (96%, 109.6 mg, 0.169 mmol).

^{1}H NMR [500 MHz, $(CD_3)_2CO$]: δ 7.68-7.59 (m, 5H, *H4-6*), 2.91 (m, 1H, *H7*), 2.48 (s, 15H, *H2*), 1.99 (app. sept., J = 7.4 Hz, 2H, *H8*), 1.71 (app. d sept., J = 7.4 Hz, 2.5 Hz, 2H, *H8*), 0.99 (t, J = 7.4 Hz, 6H, *H9*).

^{13}C NMR [126 MHz, $(CD_3)_2CO$]: δ 125.96 (*C3*), 106.86 (*C1*), 99.11 (*C4/C5+C6*), 97.16 (*C4/C5*), 43.83 (*C7*), 25.95 (*C8*), 10.63 (*C9*) 10.21 (*C2*).

HRMS (nanoESI+): *m/z* calculated for $[^{191}IrC_{21}H_{31}]^{2+}$ 237.1010, found 237.1008.

[Cp*Ir(η^6-cyclopentylbenzene)][BF$_4$]$_2$

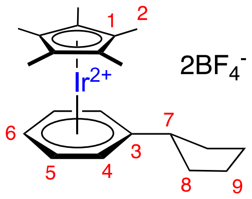

The complex was prepared according to the general procedure using cyclopentylbenzene (73.7 mg, 0.504 mmol) to afford [Cp*Ir(η^6-cyclopentylbenzene)][BF$_4$]$_2$ as a white solid (94%, 76.5 mg, 0.118 mmol).

^{1}H NMR [400 MHz, $(CD_3)_2CO$]: δ 7.72-7.71 (m, 2H, *H5*), 7.63-7.62 (m, 3H, *H4*+*H6*), 2.93 (m, 1H, *H7*), 2.50 (s, 15H, *H2*), 2.29-2.27 (m, 2H, *H8*), 1.90-1.79 (m, 6H, *H8*+*H9*).

^{13}C NMR [100 MHz, $(CD_3)_2CO$]: δ 124.23 (*C3*), 106.84 (*C1*), 99.40 (*C4*/*C5*), 98.94 (*C6*), 96.99 (*C4*/*C5*), 42.92 (*C7*), 33.71 (*C8*), 26.36 (*C9*), 10.35 (*C2*).

HRMS (ESI+): *m/z* calculated for $[^{191}IrC_{21}H_{29}]^{2+}$ 236.0938, found 236.0935.

[Cp*Ir(η^6-cyclohexylbenzene)][BF$_4$]$_2$

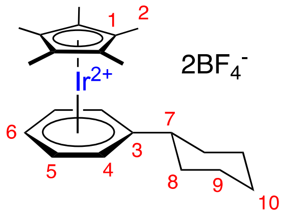

The complex was prepared according to the general procedure using cyclohexylbenzene (0.09 mL, 0.534 mmol) to afford [Cp*Ir(η^6-cyclohexylbenzene)][BF$_4$]$_2$ as a white solid (90%, 74.9 mg, 0.113 mmol).

^{1}H NMR [400 MHz, $(CD_3)_2CO$ + 20 μL TFA]: δ 7.70-7.68 (m, 2H, *H5*), 7.65-7.64 (m, 3H, *H4*+*H6*), 2.93 (tt, J = 11.7, 3.1 Hz, 1H, *H7*), 2.49 (s, 15H, *H2*), 2.11-1.26 (m, 10H, *H8*-*H10*).

^{13}C NMR [100 MHz, $(CD_3)_2CO$ + 20 μL TFA]: δ 124.56 (*C3*), 106.57 (*C1*), 99.10 (*C4*/*C5*), 98.88 (*C6*), 96.80 (*C4*/*C5*), 40.73 (*C7*), 33.16 (*C8*), 26.53 (*C9*), 25.75 (*C10*), 10.07 (*C2*).

HRMS (ESI+): *m/z* calculated for $[^{191}IrC_{22}H_{31}]^{2+}$ 243.1016, found 243.1015.

[Cp*Ir(η^6-*n*-propylbenzene)][BF$_4$]$_2$

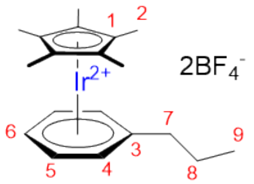

The complex was prepared according to the general procedure using [Cp*IrCl$_2$]$_2$ (50.9 mg, 0.064 mmol), AgBF$_4$ (49.3 mg, 0.253 mmol) and *n*-propylbenzene (0.070 mL, 0.502 mmol) to afford [Cp*Ir(η^6-*n*-propylbenzene)][BF$_4$]$_2$ as a white solid (85%, 64.4 mg, 0.104 mmol).

^{1}H NMR [500 MHz, $(CD_3)_2CO$/TFA (1:1)]: δ 7.37-7.32 (m, 5H, *H4-6*), 2.62 (app. t, J = 7.8 Hz, 2H, *H7*), 2.27 (s, 15H, *H2*), 1.64 (app. sext., J = 7.6 Hz, 2H, *H8*), 0.86 (t, J = 7.4 Hz, 3H, *H9*).

^{13}C NMR [126 MHz, $(CD_3)_2CO$/TFA]: δ 119.77 (*C3*), 107.45 (*C1*), 99.98 (*C4*/*C5*), 99.24 (*C4*/*C5*), 98.91 (*C6*), 35.10 (*C7*), 25.15 (*C8*), 13.36 (*C9*) 10.01 (*C2*).

HRMS (nanoESI+): *m/z* calculated for $[^{191}IrC_{19}H_{27}]^{2+}$ 223.0854, found 223.0853.

[Cp*Ir(η^6-*n*-butylbenzene)][BF$_4$]$_2$

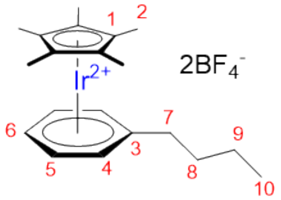

The complex was prepared according to the general procedure using [Cp*IrCl$_2$]$_2$ (50.9 mg, 0.064 mmol), AgBF$_4$ (50.4 mg, 0.259 mmol) and *n*-butylbenzene (0.080 mL, 0.511 mmol) to afford [Cp*Ir(η^6-*n*-butylbenzene)][BF$_4$]$_2$ as a white solid (94%, 76.1 mg, 0.120 mmol).

^{1}H NMR [400 MHz, (CD$_3$)$_2$CO]: δ 7.64-7.60 (m, 5H, *H4-H6*), 2.92 (m, *J* = 7.8 Hz, 2H, *H7),* 2.47 (s, 15H, *H2*), 1.78 (t of pent, *J* = 7.8 Hz, 2.1 Hz, 2H, *H8*), 1.48 (app. sextet, *J* = 7.4 Hz, 2H, *H9*), 0.94 (t, J = 7.5 Hz, 3H, *H10*).

^{13}C NMR [100 MHz, (CD$_3$)$_2$CO]: δ 119.06 (*C3*), 106.66 (*C1*), 99.55 (*C4/C5*), 98.90 (*C4/C5*), 98.53 (*C6*), 33.48 (*C8*), 33.43 (*C7*), 22.82 (*C9*), 13.92 (*C10*), 9.95 (*C2*).

HRMS (nanoESI+): *m/z* calculated for $[^{191}IrC_{20}H_{29}]^{2+}$ 230.0932, found 230.0932.

[Cp*Ir(η^6-isobutylbenzene)][BF$_4$]$_2$

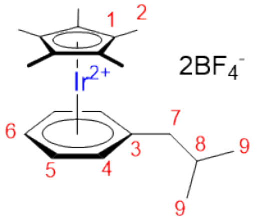

The complex was prepared according to the general procedure using [Cp*IrCl$_2$]$_2$ (50.5 mg, 0.063 mmol), AgBF$_4$ (49.9 mg, 0.256 mmol) and isobutylbenzene (0.080 mL, 0.511 mmol) to afford [Cp*Ir(η^6-isobutylbenzene)][BF$_4$]$_2$ as a white solid (100%, 80.5 mg, 0.127 mmol).

^{1}H NMR [400 MHz, (CD$_3$)$_2$CO/TFA, 1:1]: *δ* 7.35-7.28 (m, 5H, *H4-H6*), 2.48 (d, *J* = 7.1 Hz, 2H, *H7*), 2.26 (s, 15H, *H2*), 1.87 (app. nonet, *J* = 6.8 Hz, 1H, *H8*), 0.82 (d, *J* = 6.8 Hz, 6H, *H9*).

^{13}C NMR [100 MHz, (CD$_3$)$_2$CO/TFA]: *δ* 118.98 (*C3*), 107.52 (*C1*), 100.04 (*C4/C5*), 99.56 (*C4/C5*), 99.07 (*C6*), 41.73 (*C7*), 31.92 (*C8*), 21.70 (*C9*), 10.0 (*C2*).

HRMS (nanoESI+): *m/z* calculated for $[^{191}IrC_{20}H_{29}]^{2+}$ 230.0932, found 230.0932.

[Cp*Ir(η^6-neopentylbenzene)][BF$_4$]$_2$

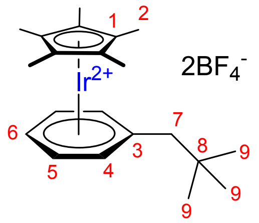

The complex was prepared according to the general procedure using [Cp*IrCl$_2$]$_2$ (70.6 mg, 0.089 mmol), AgBF$_4$ (69.1 mg, 0.355 mmol) and neopentylbenzene (0.120 mL, 0.795 mmol) to afford [Cp*Ir(η^6-neopentylbenzene)][BF$_4$]$_2$ as a white solid (90%, 109.8 mg, 0.169 mmol).

^{1}H NMR [500 MHz, (CD$_3$)$_2$CO]: *δ* 7.67-7.59 (m, 5H, *H4-H6*), 2.82 (s, 2H, *H7*), 2.48 (s, 15H, *H2*), 10.05 (s, 9H, *H9*).

^{13}C NMR [126 MHz, $(CD_3)_2CO$]: δ 116.97 (*C3*), 106.76 (*C1*), 9960 (*C4/C5*), 99.44 (*C4/C5*), 98.96 (*C6*), 415.44 (*C7*), 33.71 (*C8*), 28.86 (*C9*), 9.98 (*C2*).

HRMS (nanoESI+): *m/z* calculated for $[^{191}IrC_{21}H_{31}]^{2+}$ 237.1010, found 237.1003.

[Cp*Ir(η^6-1,4-diisopropylbenzene)][BF$_4$]$_2$

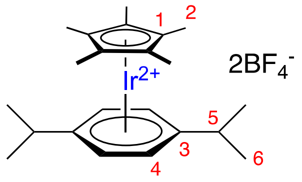

The complex was prepared according to the general procedure using and 1,4-diisopropylbenzene (0.10 mL, 0.528 mmol) to afford [Cp*Ir(η^6-1,4-diisopropylbenzene)][BF$_4$]$_2$ as a light grey-white solid (98%, 81.5 mg, 0.123 mmol).

^{1}H NMR [400 MHz, $(CD)_3CO$]: δ 7.61 (s, 4H, *H4*), 3.27 (app. sept., J = 6.9 Hz, 2H, *H5*), 2.44 (s, 15H, *H2*), 1.47 (d, J = 6.9 Hz, 12H, *H6*).

^{13}C NMR [100 MHz, $(CD)_3CO$]: δ 124.80 (*C3*), 106.26 (*C1*), 96.38 (*C4*), 31.26 (*C5*), 22.23 (*C6*), 10.10 (*C2*).

HRMS (ESI+): *m/z* calculated for $[^{191}IrC_{22}H_{33}]^{2+}$ 244.1091, found 244.1094.

[Cp*Ir(η^6-1,4-diisopropylbenzene-d$_2$)][BF$_4$]$_2$

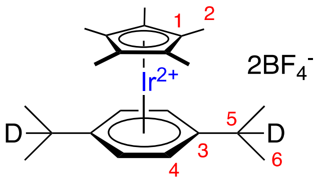

The complex was prepared according to the general procedure using and d$_2$-1,4-diisopropylbenzene (0.10 mL, 0.528 mmol) to afford [Cp*Ir(η^6 -1,4-diisopropylbenzene-d$_2$)][BF$_4$]$_2$ as a pale-yellow solid (78%, 65.1 mg, 0.098 mmol) with 91% deuteration at the benzylic positions.

N.B. Reaction mixture turns **red** upon overnight stirring as opposed to typical pale yellow. No detectable impurities, however, were found by NMR.

^{1}H NMR [500 MHz, $(CD)_3CO$]: δ 7.61 (s, 4H, *H4*), 2.44 (s, 15H, *H2*), 1.46 (s, 12H, *H6*).

^{13}C NMR [126 MHz, $(CD)_3CO$]: δ 124.75 (*C3*), 106.27 (*C1*), 96.40 (*C4*), 22.24 (*C6*), 10.12 (*C2*).

HRMS (ESI+): *m/z* calculated for $[^{191}IrC_{22}H_{31}D_2]^{2+}$ 245.1157, found 245.1149.

[Cp*Ir(η^6-1,4-diisopropylbenzene-d$_4$)][BF$_4$]$_2$

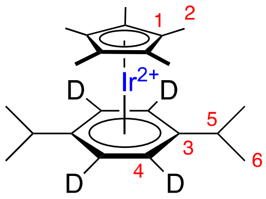

The complex was prepared according to the general procedure using and 1,4-diisopropylbenzene-d$_4$ (0.10 mL, 0.528 mmol) to afford [Cp*Ir(η^6-1,4-diisopropylbenzene-d$_4$)][BF$_4$]$_2$ as a white solid (70%, 58.7 mg, 0.088 mmol) with 97% deuteration at the aromatic positions.

^{1}H NMR [500 MHz, (CD)$_3$CO]: δ 3.26 (app. sept., J = 6.9 Hz, 2H, *H5*), 2.43 (s, 15H, *H2*), 1.46 (d, J = 6.9 Hz, 12H, *H6*).

^{13}C NMR [126 MHz, (CD)$_3$CO]: δ 124.62 (*C3*), 106.22 (*C1*), 96.05 (t, *C4*), 31.16 (*C5*), 22.21 (*C6*), 10.07 (*C2*).

HRMS (ESI+): *m/z* calculated for $[^{191}IrC_{22}H_{29}D_4]^{2+}$ 246.1220, found 246.1212.

[Cp*Ir(η^6-4-isopropyltoluene)][BF$_4$]$_2$

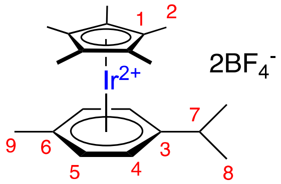

The complex was prepared according to the general procedure using 4-isopropyltoluene (0.08 mL, 0.511 mmol) to afford [Cp*Ir(η^6-4-isopropyltoluene)][BF$_4$]$_2$ as a cream solid (96%, 76.6 mg, 0.121 mmol).

^{1}H NMR [400 MHz, (CD$_3$)$_2$CO + 20 μL TFA]: δ 7.65-76.54 (m, 4H, *H4+H5*), 3.25 (app. sept., J = 6.9 Hz, 1H, *H7*), 2.75 (s, 3H, *H9*), 2.44 (s, 15H, *H2*), 1.46 (d, J = 6.9 Hz, 6H, *H8*).

^{13}C NMR [100 MHz, (CD$_3$)$_2$CO + 20 μL TFA]: δ 124.02 (*C3*), 115.83 (*C6*), 105.86 (*C1*), 98.83 (*C4*/*C5*), 96.19 (*C4*/*C5*), 31.27 (*C7*), 21.96 (*C8*), 17.60 (*C9*), 9.62 (*C2*).

HRMS (ESI+): *m/z* calculated for $[^{191}IrC_{20}H_{29}]^{2+}$ 230.0938, found 230.0937.

4. Preparation of η^4-arene complexes

General procedure for the preparation of [Cp*Ir(η^4-arene)] (modified literature procedure)[1]

In a glovebox, [Cp*Ir(η^6-arene)][BF_4]$_2$ (0.043-0.082 mmol) and two eq. of $CoCp_2$ (0.085-0.164 mmol) were suspended in benzene (1 mL). The resultant mixture was stirred vigorously for 2 h. Hexane (8 mL) was then added, and the yellow/orange suspension was filtered through glass wool. The precipitate was washed with additional hexane until the washings remained colourless. The combined filtrate and washings were evaporated to dryness to afford the product as a residue/oil. Product ratios calculated from relative integrals in their respective ^{1}H NMR spectrum.

[Cp*Ir(η^4-ethylbenzene)] (1f)

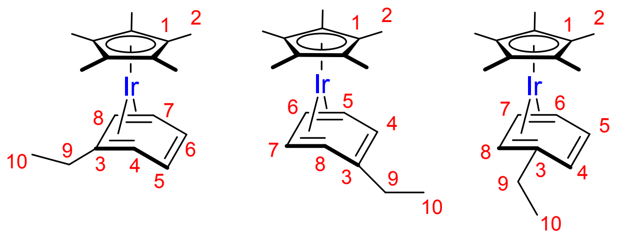

Complex **1f** was prepared according to the general procedure using [Cp*Ir(η^6-ethyl-benzene)][BF_4]$_2$ (50.0 mg, 0.082 mmol) and $CoCp_2$ (31.1 mg, 0.164 mmol) to afford [Cp*Ir(η^4-ethyl-benzene)] as a light brown oil (90%, 32.1 mg, 0.074 mmol). The product was obtained as a mixture of three regioisomers.

Regioisomer **1f_a**: ^{1}H NMR (400 MHz, C_6D_6): δ 5.74 (d, J = 4.6 Hz, 1H, *H8*), 5.51 (m, 2H, *H5*+*H6*), 3.12 (m, 1H, *H7*), 3.02 (m, 1H, *H4*), 2.26 (m, 2H, *H9*), 1.82 (s, 15H, *H2*), 1.21 (t, J = 7.5 Hz, 3H, *H10*).

^{13}C NMR (100 MHz, C_6D_6): δ 131.64 (*C6*), 130.72 (*C5*), 88.83 (*C1*), 85.92 (*C3*), 67.01 (*C8*), 50.03 (*C4*), 48.60 (*C7*), 27.25 (*C9*), 15.01 (*C10*), 10.48 (*C2*).

Regioisomer **1f_b**: ^{1}H NMR (400 MHz, C_6D_6): δ 5.79 (m, 2H, *H6*+*H7*), 5.10 (m, 1H, *H4*), 3.17 (app. t, J = 4.2 Hz, 1H, *H5*), 3.02 (m, 1H, *H8*), 1.89 [m, 2H, *H9* (merged with *H2*)], 1.86 (s, 15H, *H2*), 1.02 (t, J = 7.4 Hz, 3H, *H10*).

^{13}C NMR (100 MHz, C_6D_6): δ 146.09 (*C3*), 122.79 (*C4*), 89.17 (*C1*), 66.48 (*C6*), 65.69 (*C7*), 49.67 (*C8*), 45.92 (*C5*), 25.80 (*C9*), 13.11 (*C10*), 10.59 (*C2*).

Regioisomer **1f** (partial assignment): ^{1}H NMR (400 MHz, C_6D_6): δ 5.70 (m, 1H, *H7*/*H8*), 5.69 (m, 1H, *H7*/*H8*), 3.13 (m, 1H, *H6*), 1.09 (t, J = 7.4 Hz, 3H, *H10*).

^{13}C NMR (100 MHz, C_6D_6): δ 68.90 (*C7*/*C8*), 67.69 (*C7*/*C8*), 47.83 (*C6*), 30.16 (*C9*), 14.31 (*C10*), 10.26 (*C2*).

HRMS (ASAP): *m/z* calculated for $[^{191}IrC_{18}H_{26}]^+$ 433.1640, found 433.1616.

[Cp*Ir(η^4-isopropylbenzene)] (1a)

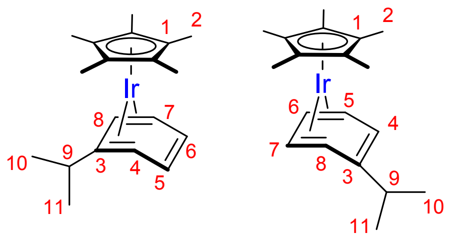

1a_a **1a_b**

1.78 : 1

Complex **1a** was prepared according to the general procedure using [Cp*Ir(η^6-isopropyl-benzene)][BF_4]$_2$ (50.0 mg, 0.080 mmol) and $CoCp_2$ (30.9 mg, 0.163 mmol) to afford [Cp*Ir(η^4-isopropylbenzene)] as a light brown oil (98%, 35.4 mg, 0.079 mmol). The product was obtained as a mixture of two regioisomers.

Regioisomer **1a_a**: ^{1}H NMR (400 MHz, C_6D_6): δ 5.83-5.77 (m, 1H, *H8*), 5.56-5.52 (m, 2H, *H5*+*H6*), 3.11-3.09 (m, 2H, *H4*+*H7*), 2.24 (app. sept., J = 6.8 Hz, 1H, *H9*), 1.83 (s, 15H, *H2*), 1.30 (d, J = 6.9 Hz, 3H, *H10*), 1.26 (d, J = 6.8 Hz, 3H, *H11*).

^{13}C NMR (100 MHz, C_6D_6): δ 131.66 (*C5*), 130.76 (*C6*), 91.57 (*C3*), 88.89 (*C1*), 65.32 (*C8*), 48.78 (*C7*), 48.46 (*C4*), 32.70 (*C9*), 24.57 (*C10*), 22.26 (*C11*), 10.58 (*C2*).

Regioisomer **1a_b**: ^{1}H NMR (400 MHz, C_6D_6): δ 5.83-5.77 (m, 2H, *H6*+*H7*), 5.09-5.08 (m, 1H, *H4*), 3.16 (app. t, J = 4.5 Hz, 1H, *H5*), 3.06 (dd, J = 4.6, 2.0 Hz, 1H, *H8*), 2.12 (app. sept., J = 6.8 Hz, 1H, *H9*), 1.87 (s, 15H, *H2*), 1.06 (d, 3H, J = 6.8 Hz, *H10*), 1.05 (d, 3H, J = 6.7 Hz, *H11*).

^{13}C NMR (100 MHz, C_6D_6): δ 150.77 (*C3*), 121.17 (*C4*), 89.13 (*C1*), 66.50 (*C6*), 65.66 (*C7*), 48.35 (*C8*), 45.75 (*C5*), 30.94 (*C9*), 21.74 (*C10*), 21.53 (*C11*), 10.58 (*C2*).

HRMS (ASAP): *m/z* calculated for $[^{191}IrC_{19}H_{28}]^+$ 447.1797, found 447.1790.

[Cp*Ir(η^4-*sec*-butylbenzene)] (1b)

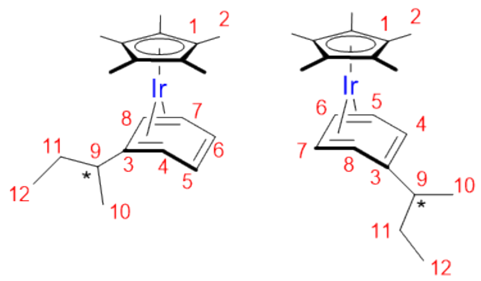

1b_aa **1b_ba**
1b_ab **1b_bb**
37 : 63

Complex **1b** was prepared according to the general procedure using [Cp*Ir(η^6-*sec*-butylbenzene)][BF$_4$]$_2$ (88.6 mg, 0.139 mmol) and CoCp$_2$ (52.8 mg, 0.279 mmol) in benzene (1.8 ml) to afford [Cp*Ir(η^4- *sec*-butylbenzene)] as a dark brown oil (98%, 63.2 mg, 0.137 mmol). The product was obtained as a mixture of two regioisomers each existing as two diastereomers.

Regioisomer **1b_a** (2 diastereomers **1b_aa** and **1b_ab**): ^{1}H NMR (500 MHz, C$_6$D$_6$): δ 5.81 (dd, J = 4.3, 2.2 Hz, 1H, ***aa/ab**-H8*), 5.80 (dd, merged with ***ba**-H6+**bb**-H6+**ba**-H7+**bb**-H7,* J = 4.6, 2.1 Hz, 1H, ***aa/ab**-H8*), 5.53-5.48 (m, 2H+2H, ***aa**-H5+**ab**-H5+**aa**-H6+**ab**-H6*), 3.12-3.08 (m, 1H+1H, ***aa**-H4+**ab**-H4*), 3.01 (dt, J = 2.9, 0.6 Hz, 1H+1H, ***aa**-H7+**ab**-H7*), 2.18-2.13 (m, 1H, ***aa/ab**-H9*), 1.84 (s, 15H, ***aa/ab**-H2*), 1.83 (s, 15H, ***aa/ab**-H2*), 1.84-1.83 (m, merged with ***ba**-H2+**bb**-H2* and ***aa**-H2+**ab**-H2,* 1H, ***aa/ab**-H9),* 1.82-1.57 (m, 2H+2H, ***aa**-H11+**ab**-H11*), 1.28 (d, J = 6.9 Hz, 3H, ***aa/ab**-H10*), 1.17 (d, J = 6.8 Hz, 3H, ***aa/ab**-H10*), 1.03 (t, merged with ***ba/bb**-H10* and ***ba**-H12+**bb**-H12,* J = 7.4 Hz, 3H, ***aa/ab**-H12*), 1.02 (t, merged with ***ba/bb**-H10* and ***ba**-H12+**bb**-H12,* J = 7.4 Hz, 3H, ***aa/bb**-H12*).

^{13}C NMR (126 MHz, C$_6$D$_6$): δ 131.56 (***aa/ab**-C5*), 131.51 (***aa/ab**-C5*), 130.99 (***aa/ab**-C6*), 130.72 (***aa/ab**-C6*), 91.09 (***aa/ab**-C3*), 90.54 (***aa/ab**-C3*), 88.98 (***aa/ab**-C1*), 88.85 (***aa/ab**-C1*), 66.82 (***aa/ab**-C8*), 64.92 (***aa/ab**-C8*), 50.88 (***aa/ab**-C7*), 48.82 (***aa/ab**-C7*), 48.70 (***aa/ab**-C4*), 47.01 (***aa/ab**-C4*) 39.86 (***aa/ab**-C9*), 39.46 (***aa/ab**-C9*), 32.52 (***aa/ab**-C11*), 30.64 (***aa/ab**-C11*), 20.41 (***aa/ab**-C10*), 19.14 (***aa/ab**-C10*), 12.59 (***aa/ab**-C12*), 12.32 (***aa/ab**-C12*), 10.58 (***aa**-C2+**ab**-C2*).

Regioisomer **1b_b** (2 diastereomers **1b_ba** and **1b_bb**): ^{1}H NMR (500 MHz, C$_6$D$_6$): δ 5.78-5.75 (m, 2H+2H, ***ba**-H6+**bb**-H6+**ba**-H7+**bb**-H7*), 5.10 (ddd, J = 4.3, 2.1, 0.6 Hz, 1H, ***ba/bb**-H4*), 5.07 (ddd, J = 4.3, 2.1, 0.9 Hz, 1H, ***ba/bb**-H4*), 3.15 (ddt, J = 6.0, 2.0, 0.5 Hz, 1H+1H, ***ba**-H5+**bb**-H5*), 3.05-3.02 (*m*, 1H+1H, ***ba**-H8+**bb**-H8*), 2.03 (m, 1H, ***ba/bb**-H9*), 1.94 (m, 1H, ***ba/bb**-H9),* 1.87 (s, 15H, ***ba/bb**-H2*), 1.86 (s, 15H, ***ba/bb**-H2*), 1.56-1.48 (m, 1H, ***ba/bb**-H11)**,* 1.48-1.40 (m, 1H, ***ba/bb**-H11*), 1.40-1.29 (m, 2H, ***ba/bb**-H11*), 1.06 (d, J = 6.8 Hz, 3H, ***ba/bb**-H10*), 1.00 (d, J = 6.8 Hz, 3H, ***ba/bb**-H10*), 0.99 (t, J = 7.4 Hz, 3H, ***ba/bb**-H12*), 0.91 (t, J = 7.4 Hz, 3H, ***ba/bb**-H12*).

^{13}C NMR (126 MHz, C$_6$D$_6$): δ 148.73 (***ba/bb**-C3*), 148.59 (***ba/bb**-C3*), 123.77 (***ba/bb**-C4*), 123.11 (***ba/bb**-C4*), 89.10 (***ba/bb**-C1*), 89.09 (***ba/bb**-C1*), 66.73 (***ba/bb**-C6*), 66.44 (***ba/bb**-C6*), 65.83

(***ba/bb***-*C7*), 65.72 (***ba/bb***-*C7*), 47.62 (***ba/bb***-*C8*), 47.02 (***ba/bb***-*C8*), 45.72 (***ba/bb***-*C5*), 45.66 (***ba/bb***-*C5*), 38.26 (***ba/bb***-*C9*), 37.86 (***ba/bb***-*C9*), 28.19 (***ba/bb***-*C11*), 27.99 (***ba/bb***-*C11*), 19.50 (***ba/bb***-*C10*), 19.49 (***ba/bb***-*C10*), 12.49 (***ba/bb***-*C12*), 11.91 (***ba/bb***-*C12*), 10.66 (***ba/bb***-*C2*), 10.53 (***ba/bb***-*C2*).

HRMS (nanoESI+): *m/z* calculated for $[^{193}IrC_{20}H_{30}]^+$ 463.1971, found 463.1980.

[Cp*Ir(η^4-3-pentylbenzene)] (1c)

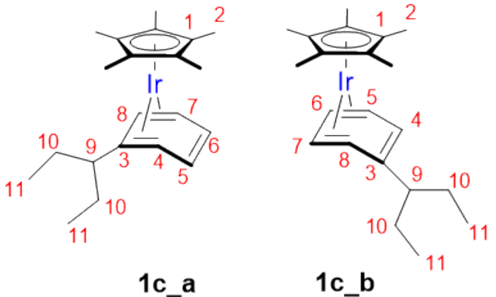

Complex **1c** was prepared according to the general procedure using [Cp*Ir(η^6-3-pentylbenzene)][BF_4]$_2$ (92.8 mg, 0.143 mmol) and $CoCp_2$ (54.1 mg, 0.286 mmol) in benzene (1.9 ml) to afford [Cp*Ir(η^4- 3-pentylbenzene)] as a dark brown oil (91%, 61.6 mg, 0.129 mmol). The product was obtained as a mixture of two regioisomers.

Regioisomer **1c_a**: ^{1}H NMR (500 MHz, C_6D_6): δ 5.72 (dd, J = 4.8, 0.7 Hz, 1H, *H8*), 5.52 (m, 2H, *H5*+*H6*), 3.12-3.08 (m, 1H, *H4*), 3.03-2.98 (m, merged with ***b***-*H5,* 1H, *H7*), 1.84 (s, 15H, *H2*), 1.82-1.75 (m, overlaps with ***b***-*H9*, 1H, *H9*), 1.56-1.28 (m, overlaps with ***b***-*H10*, 4H, *H10*), 1.02 (t, merged with ***b***-*H11*, J = 7.4 Hz, 3H, *H11*), 0.76 (t, J = 7.4 Hz, 3H, *H11*).

^{13}C NMR (126 MHz, C_6D_6): δ 131.23 (*C5*), 130.72 (*C6*), 88.69 (*C1*), 66.03 (*C8*), 49.95 (*C7*), 48.52 (*C4*), 46.05 (*C9*), 29.64 (*C10*), 27.42 (*C10*), 12.48 (*C11*), 11.58 (*C11*), 10.51 (*C2*). *C3* not observed on the spectrum.

Regioisomer **1c_b**: ^{1}H NMR (500 MHz, C_6D_6): δ 5.80-5.73 (m, 2H, *H6*+*H7*), 5.11 (dd, J = 4.3, 2.0 Hz, 1H, *H4*), 3.15 (dt, J = 4.4, 0.4 Hz, 1H, *H5*), 3.00 (dd, merged with ***a***-*H4,* J = 4.7 Hz, 2.0 Hz, 1H, *H8*), 1.86 (s, 15H, *H2*), 1.82-1.75 (m, 1H, *H9*), 1.56-1.45 (m, 1H, *H10*), 1.44-1.28 (m, overlaps with ***a***-*H10*, 2H, *H10*), 1.44-1.28 (m, *overlaps with* ***a***-*H10*, 2H, *H10*), 1.05 (t, J = 7.4 Hz, 3H, *H11*), 0.88 (t, J = 7.4 Hz, 3H, *H11*).

^{13}C NMR (126 MHz, C_6D_6): δ 145.68 (*C3*), 125.82 (*C4*), 88.79 (*C1*), 66.55 (*C6*), 65.78 (*C7*), 46.08 (*C8*), 45.84 (*C9*), 45.47 *(C5)*, 26.49 (*C10*), 26.44 (*C10*), 12.69 (*C11*), 12.36 (*C11*), 10.70 (*C2*).

HRMS (nanoESI+): *m/z* calculated for $[^{193}IrC_{21}H_{32}]^+$ 477.2128, found 477.2155.

[Cp*Ir(η^4-cyclopentylbenzene)] (1d)

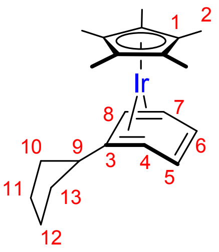

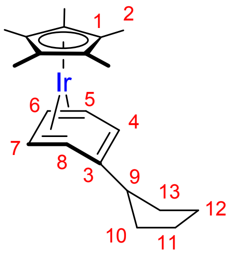

1d_a **1d_b**

1 : 2

Complex **1d** was prepared according to the general procedure using [Cp*Ir(η^6-cyclopentyl-benzene)][BF_4]$_2$ (30.0 mg, 0.046 mmol) and $CoCp_2$ (17.5 mg, 0.093 mmol) to afford [Cp*Ir(η^4-cyclopentylbenzene)] as an orange solid (99%, 21.9 mg, 0.046 mmol). The product was obtained as a mixture of two regioisomers.

Regioisomer **1d_a**: ^{1}H NMR (400 MHz, C_6D_6): δ 5.76 (d, J = 4.7 Hz, 1H, *H8*), 5.54-5.53 (m, 2H, *H5*+*H6*), 3.11-3.09 (m, 2H, *H4*+*H7*), 2.63 (app. quin, J = 7.7 Hz, 1H, *H9*), 1.92-1.50 (m, 8H, *H10-H13*), 1.85 (s, 15H, *H2*).

^{13}C NMR (100 MHz, C_6D_6): δ 131.68 (*C5*), 130.72 (*C6*), 89.53 (*C3*), 88.99 (*C1*), 66.29 (*C8*), 48.87 (*C7*), 48.61 (*C4*), 43.89 (*C9*), 33.71 (*C10*/*C13*), 32.98 (*C10*/*C13*), 26.14 (*C11*/*C12*), 25.93 (*C11*/*C12*), 10.72 (*C2*).

Regioisomer **1d_b**: ^{1}H NMR (400 MHz, C_6D_6): δ 5.83-5.42 (m, 1H, *H6*), 5.80-5.78 (m, 1H, *H7*), 5.13-5.11 (m, 1H, *H4*), 3.17 (app. t, J = 4.5 Hz, 1H, *H5*), 3.07 (dd, J = 4.7, 2.0 Hz, 1H, *H8*), 2.32 (app. quin, J = 7.9 Hz, 1H, *H9*), 1.92-1.50 (m, 8H, *H10-H13*), 1.87 (s, 15H, *H2*).

^{13}C NMR (100 MHz, C_6D_6): δ 148.01 (*C3*), 122.09 (*C4*), 89.12 (*C1*), 66.53 (*C6*), 65.70 (*C7*), 48.63 (*C8*), 45.81 (*C5*), 42.80 (*C9*), 31.66 (*C10*/*C13*), 31.46 (*C10*/*C13*), 25.72 (*C11*/*C12*), 25.58 (*C11*/*C12*), 10.60 (*C2*).

HRMS (ESI+): *m/z* calculated for [$^{191}IrC_{21}H_{30}$]$^+$ 473.1954, found 473.1897.

[Cp*Ir(η^4-cyclohexylbenzene)] (1e)

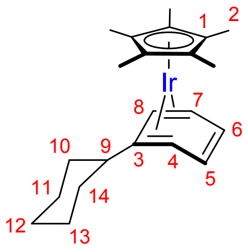

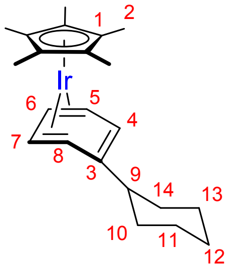

1e_a **1e_b**

1.5 : 1

Complex **1e** was prepared according to the general procedure using [Cp*Ir(η^6-cyclohexyl-benzene)][BF_4]$_2$ (50.0 mg, 0.076 mmol) and $CoCp_2$ (29.0 mg, 0.153 mmol) to afford [Cp*Ir(η^4-cyclohexylbenzene)] as a brown oil (99%, 36.6 mg, 0.075 mmol). The product was obtained as a mixture of two regioisomers.

Regioisomer **1e_a**: ^{1}H NMR (400 MHz, C_6D_6): δ 5.84-5.83 (m, 1H, *H8*), 5.56-5.51 (m, 2H, *H5*+*H6*), 3.13-3.11 (m, 2H, *H4*+*H7*), 2.02-1.11 (m, 11H, *H9-H14*), 1.85 (s, 15H, *H2*).

^{13}C NMR (100 MHz, C_6D_6): δ 131.58 (*C5*), 130.72 (*C6*), 90.15 (*C3*), 88.80 (*C1*), 65.50 (*C8*), 48.68 (*C7*), 48.63 (*C4*), 42.80 (*C9*), 35.93 (*C10*/*C14*), 33.14 (*C10*/*C14*), 27.46 (*C11*/*C13*), 27.31 (*C11*/*C13*), 27.07 (*C12*), 10.70 (*C2*).

Regioisomer **1e_b**: ^{1}H NMR (400 MHz, C_6D_6): δ 5.84-5.43 (m, 1H, *H6*/*H7*), 5.80-5.78 (m, 1H, *H6*/*H7*), 5.09-5.08 (m, 1H, *H4*), 3.18 (app. t, J = 4.5 Hz, 1H, *H5*), 3.09 (m, 1H, *H8*), 2.02-1.11 (m, 11H, *H9-H14*), 1.88 (s, 15H, *H2*).

^{13}C NMR (100 MHz, C_6D_6): δ 149.79 (*C3*), 121.49 (*C4*), 89.03 (*C1*), 66.43 (*C6*), 65.62 (*C7*), 48.76 (*C8*), 45.89 (*C5*), 41.18 (*C9*), 32.37 (*C10*/*C14*), 32.26 (*C10*/*C14*), 27.07 (*C11*/*C13*), 26.94 (*C11*/*C13*), 26.89 (*C12*), 10.70 (*C2*).

HRMS (ESI+): *m/z* calculated for $[^{191}IrC_{22}H_{30}]^+$ 485.1954, found 485.1935.

[Cp*Ir(η^4-*n*-propylbenzene)] (1g)

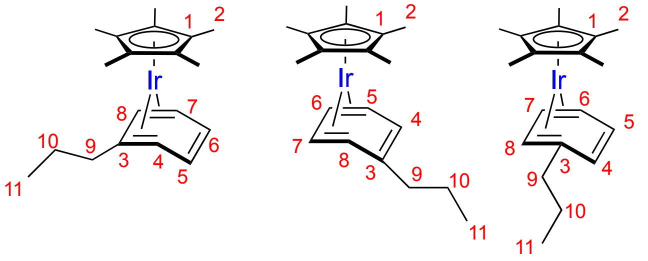

1g_a 57 : **1g_b** 41 : **1g_c** 2

Complex **1g** was prepared according to the general procedure using [Cp*Ir(η^6-*n*-propylbenzene)][BF_4]$_2$ (82.7 mg, 0.133 mmol) and $CoCp_2$ (50.4 mg, 0.266 mmol) in benzene (1.7 ml) to afford [Cp*Ir(η^4-*n*-propylbenzene)] as a dark brown oil (90%, 53.8 mg, 0.120 mmol). The product was obtained as a mixture of three regioisomers.

Regioisomer **1g_a**: ^{1}H NMR (500 MHz, C_6D_6): δ 5.73 (d, J = 4. Hz, 1H, *H8*), 5.54 (m, 2H, *H5*+*H6*), 3.19-3.15 (m, merged with ***b***-*H8,* 1H, *H4*), 3.00 (app dt, J = 2.9, 0.5 Hz, 1H, *H7*), 2.37 (m, 1H, *H9*), 2.05 (m, merged with ***b***-H9, 1H, *H9*), 1.83 (s, 15H, *H2*), 1.68 (m, 2H, *H10*), 1.04 (t, J = 7.4 Hz, 3H, *H10*).

^{13}C NMR (126 MHz, C_6D_6): δ 131.53 (*C6*), 130.76 (*C5*), 88.45 (*C1*), 83.80 (*C3*), 68.01 (*C8*), 50.68 (*C7*), 48.66 (*C4*), 36.85 (*C9*), 25.05 (*C10*), 14.41 (*C11*), 10.47 (*C2*).

Regioisomer **1g_b**: ^{1}H NMR (500 MHz, C_6D_6): δ 5.79 (m, 2H, *H6*+*H7*), 5.13 (m, 1H, *H4*), 3.19-3.15 (m, merged with ***a***-*H4,* 1H, *H8*), 3.03 (dd, J = 4.7 Hz, 2.0 Hz, 1H, *H5*), 2.02 (m, merged with

a-H9, 1H, *H9*), 1.87 (s, 15H, *H2*), 1.49 (d sext., *J* = 7.4 Hz, 1.4 Hz, 2H, *H10*), 0.94 (t, *J* = 7.4 Hz, 3H, *H11*).

^{13}C NMR (126 MHz, C_6D_6): δ 144.06(*C3*), 124.40 (*C4*), 89.15 (*C1*), 66.47 (*C6*), 65.74 (*C7*), 49.57 (*C5*), 45.89 (*C8*), 34.72 (*C9*), 21.38 (*C10*), 13.95 (*C11*), 10.59 (*C2*).

Regioisomer **1g_c** (partial assignment): ^{1}H NMR (500 MHz, C_6D_6): δ 5.71 (m, 1H, *H7*/*H8*), 5.67 (m, 1H, *H7*/*H8*).

^{13}C NMR (126 MHz, C_6D_6): δ 10.26 (*C2*).

HRMS (ESI+): *m/z* calculated for $[^{193}IrC_{19}H_{28}]^+$ 449.1815, found 449.1840.

[Cp*Ir(η^4-*n*-butylbenzene)] (1h)

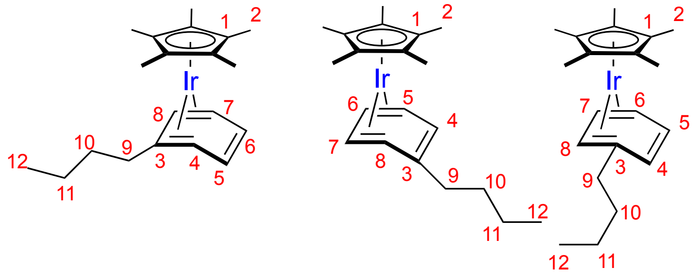

Complex **1h** was prepared according to the general procedure using [Cp*Ir(η^6-*sec*-butylbenzene)][BF_4]$_2$ (90.2 mg, 0.142 mmol) and $CoCp_2$ (53.7 mg, 0.284 mmol) in benzene (1.8 ml) to afford [Cp*Ir(η^4-*n*-butylbenzene)] as a dark brown oil (94%, 61.7 mg, 0.134 mmol). The product was obtained as a mixture of three regioisomers.

Regioisomer **1h_a**: ^{1}H NMR (400 MHz, C_6D_6): δ 5.74 (d, *J* = 4.7 Hz, 1H, *H8*), 5.55-5.50 (m, 2H, *H5*+*H6*), 3.16-3.13 (m, merged with ***b***-*H5,* 1H, *H7*), 3.02-3.00 (m, 1H, *H4),* 2.38 (ddd, *J* = 13.9, 8.9, 6.3 Hz, 1H, *H9*) 2.12 (ddd, *J* = 13.9, 8.7, 6.2 Hz, 1H, *H9*), 1.83 (s, 15H, *H2*), 1.70-1.60 (m, 2H, *H10*), 1.52-1.27 (m, overlaps with ***b***-*H10* and ***b***-*H11*, 1H, *H11*), 0.96 (d, *J* = 7.3 Hz, 3H, *H12).*

^{13}C NMR (100 MHz, C_6D_6): δ 131.53 (*C5*), 130.70 (*C6*), 88.81 (*C3*), 84.03 (*C1*), 67.87 (*C8*), 50.58 (*C4*), 48.63 (*C7*), 34.44 (*C9*), 34.00 (*C10*), 23.19 (*C11*), 14.46 (*C12*), 10.53 (*C2*).

Regioisomer **1h_b**: ^{1}H NMR (400 MHz, C_6D_6): δ 5.80-5.77 (m, 2H, *H6*+*H7*), 5.12-5.10 (m, 1H, *H4*), 3.17-3.13 (m, merged with **a**-*H7, J* = 4.4, 1.1 Hz, 1H, *H5*), 3.04-3.02 (m, 1H, *H8*), 2.07-2.01 (m, 1H, *H9)*, 1.87 (s, 15H, *H2*), 1.87-1.83 (m, overlaps with the ***a***-*H2* and ***b***-, 1H, *H9),* 1.52-1.27 (m, overlaps with ***a***-*H10* and ***b***-*H11*, 1H, *H10*), 1.52-1.27 (m, overlaps with ***a***-*H11* and ***b***-*H10*, 2H, *H11*), 0.93 (d, *J* = 7.1 Hz, 3H, *H12*),.

^{13}C NMR (100 MHz, C_6D_6): δ 144.19 (*C3*), 124.18 (*C4*), 89.12 (*C1*), 66.47 (*C6*), 65.75 (*C7*), 49.55 (*C8*), 45.89 (*C5*), 32.25 (*C9*), 30.46 (*C10*), 22.51 (*C11*), 14.35 (*C12*), 10.63 (*C2*).

Regioisomer **1h_c** (partial assignment): ^{1}H NMR (400 MHz, C_6D_6): δ 5.70 (m, 1H, *H7*/*H8*), 5.65 (m, 1H, *H7*/*H8*), 1.09 (t, J = 7.4 Hz, 3H, *H10*).

^{13}C NMR (100 MHz, C_6D_6): δ 69.50 (*C7*/*C8*), 67.63 (*C7*/*C8*), 47.75 (*C6*), 37.44 (*C9*), 32.18 (*C10*), 23.53 (*C11*), 10.31 (*C2*).

HRMS (ASAP): *m/z* calculated for $[^{193}IrC_{20}H_{30}]^{+}$ 463.1971, found 463.1979.

[Cp*Ir(η^4-isobutylbenzene)] (2i)

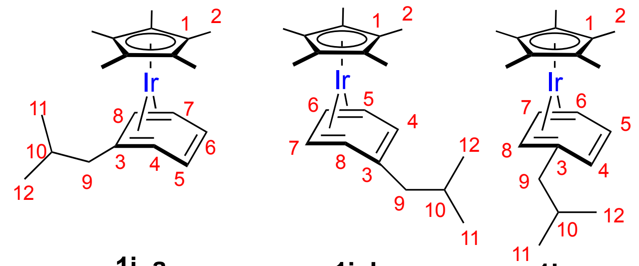

1i_a : **1i_b** : **1i_c**

40 : 59 : 1

Complex **1i** was prepared according to the general procedure using [Cp*Ir(η^6-*sec*-butylbenzene)][BF_4]$_2$ (89.6 mg, 0.141 mmol) and $CoCp_2$ (53.4 mg, 0.282 mmol) in benzene (1.8 ml) to afford [Cp*Ir(η^4- isobutylbenzene)] as a dark brown oil (92%, 59.9 mg, 0.130 mmol). The product was obtained as a mixture of three regioisomers.

Regioisomer **1i_a**: ^{1}H NMR (400 MHz, C_6D_6): δ 5.69 (dd, J = 4.6 Hz, 0.5 Hz, 1H, *H8*), 5.53-5.49 (m, 2H, *H5*+*H6*), 3.18-3.14 (m, merged with ***b***-*H8,* 1H, *H4*), 2.95 (dt, J = 3.0 Hz, 0.7 Hz, 1H, *H7),* 2.50 (dd, J = 12.9 Hz, 6.8 Hz, 1H, *H9*) 1.95-1.90 (m, 1H, *H10*), 1.83 (s, 15H, *H2*), 1.73 (dd, J = 13.0 Hz, 7.3 Hz, 1H, *H9*), 1.08 (d, J = 6.7 Hz, 3H, *H11*/*H12*), 1.07 (d, J = 6.7 Hz, 3H, *H11/H12).*

^{13}C NMR (100 MHz, C_6D_6): δ 131.30 (*C5*), 130.80 (*C6*), 88.75 (*C1*), 81.91 (*C3*), 69.11 (*C8*), 51.42 (*C4*), 48.78 (*C7*), 44.61 (*C9*), 31.61 (*C10*), 22.98 (*C11*/*C12*), 22.96 (*C11*/*C12*), 10.55 (*C2*).

Regioisomer **1i_b**: ^{1}H NMR (400 MHz, C_6D_6): δ 5.79-5.76 (m, 2H, *H6*+*H7*), 5.11-5.09 (m, 1H, *H4*), 3.18-3.14 (dt, merged with ***a***-*H4,* J = 4.4 Hz, 1.0 Hz, 1H, *H5*), 3.02-3.00 (m, 1H, *H8*), 2.02-1.95 (m , 1H, *H9),* 1.86 (s, 15H, *H2*), 1.86-1.83 (overlaps with the **a**-*H2* and **b**-, m, 1H, *H10),* 1.68 (ddd, J = 13.3 Hz, 6.3 Hz, 1.3 Hz, 1H, *H9*), 1.01 (d, 3H, J = 6.6 Hz, *H11*/*H12*), 0.94 (d, 3H, J = 6.6 Hz, *H11*/*H12*),.

^{13}C NMR (100 MHz, C_6D_6): δ 143.25 (*C3*), 125.48 (*C4*), 89.05 (*C1*), 66.46 (*C6*), 65.78 (*C7*), 49.67 (*C8*), 45.78 (*C5*), 42.28 (*C9*), 26.93 (*C10*), 23.04 (*C11*/*C12*), 22.58 (*C11/C12),* 10.69 (*C2*).

Regioisomer **1i_c** (partial assignment): ^{1}H NMR (400 MHz, C_6D_6): δ 5.70 (m, 1H, *H7*/*H8*), 5.60 (m, 1H), 5.41 (dd, J = 6.4, 1.6 Hz, 1H).

^{13}C NMR (100 MHz, C_6D_6): δ 71.02 (*C7*/*C8*), 67.57 (*C7*/*C8*), 47.58 (*C6*/*C9*), 47.45 (*C6*/*C9*), 31.95 (*C10*), 14.31 (*C10*).

HRMS (ASAP): *m/z* calculated for $[^{193}IrC_{20}H_{30}]^+$ 463.1971, found 463.1979.

[Cp*Ir(η^4-neopentylbenzene)] (1j)

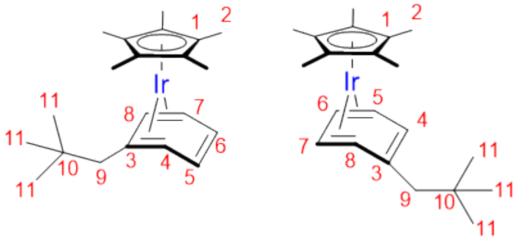

1j_a : 1j_b

71 : 29

Complex **1j** was prepared according to the general procedure using [Cp*Ir(η^6-neopentylbenzene)][BF_4]$_2$ (90.0 mg, 0.143 mmol) and $CoCp_2$ (52.5 mg, 0.278 mmol) in benzene (1.8 ml) to afford [Cp*Ir(η^4-neopentylbenzene)] as a dark brown oil (83%, 54.6 mg, 0.115 mmol). The product was obtained as a mixture of two regioisomers.

Regioisomer **1j_a**: ^{1}H NMR (500 MHz, C_6D_6): δ 5.70 (d, J = 4.6 Hz, 1H, *H8*), 5.56-5.49 (m, 2H, *H5*+*H6*), 3.19 (dt, J = 4.4, 1.9 Hz, 1H, *H7*), 2.96-2.92 (m, 1H, *H4),* 2.56 (d, J = 12.9 Hz, 1H, *H9*), 1.84 (s, 15H, *H2*), 1.70 (d, J = 13.1 Hz, 1H, *H9*), 1.10 (s, 9H, *H11*).

^{13}C NMR (126 MHz, C_6D_6): δ 131.11 (*C5*), 131.05 (*C6*), 88.81 (*C1*), 80.23 (*C3*), 70.06 (*C8*), 52.58 (*C4*), 50.23 (*C9*), 48.90 (*C7*), 32.42 (*C10*), 30.10 (*C11*), 10.50 (*C2*).

Regioisomer **1j_b**: ^{1}H NMR (500 MHz, C_6D_6): δ 5.77-5.74 (m, 2H, *H6*+*H7*), 5.10-5.08 (m, 1H, *H4*), 3.16 (app. t, J = 4.4 Hz, 1.1 Hz, 1H, *H5*), 3.08-3.06 (m, 1H, *H8*), 2.27 (d, J = 12.9 Hz, 1H, *H9)*, 1.86 (s, 15H, *H2*), 1.63 (d, J = 13.1 Hz, 1H, *H9),* 1.03 (s, 9H, *H11*).

^{13}C NMR (126 MHz, C_6D_6): δ 142.39 (*C3*), 127.33 (*C4*), 89.11 (*C1*), 66.25 (*C6*), 65.63 (*C7*), 52.22 (*C8*), 47.06 (*C9*), 46.00 (*C5*), 31.52 (*C10*), 30.23 (*C11*), 10.67 (*C2*).

HRMS (nanoESI+): *m/z* calculated for $[^{193}IrC_{21}H_{32}]^+$ 477.2128, found 477.2161.

[Cp*Ir(η^4-1,4-diisopropylbenzene)] (1l)

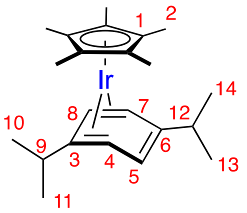

Complex **1l** was prepared according to the general procedure using [Cp*Ir(η^4-1,4-diisopropylbenzene)][BF_4]$_2$ (41.6 mg, 0.063 mmol) and $CoCp_2$ (23.7 mg, 0.125 mmol) to afford [Cp*Ir(η^4-1,4-diisopropylbenzene)] as an orange solid (97%, 29.7 mg, 0.061 mmol).

^{1}H NMR (400 MHz, C_6D_6): δ 5.76-5.75 (m, 1H, *H8*), 5.11-5.09 (m, 1H, *H5*), 3.02 (dd, J = 4.5, 0.7 Hz, 1H, *H4*), 2.93 (dd, J = 4.8, 2.3 Hz, 1H, *H7*), 2.26 (app. sept., J = 6.8 Hz, 1H, *H9*), 2.08 (d sept., J = 6.8, 1.3 Hz, 1H, *H12*), 1.86 (s, 15H, *H2*), 1.29 (d, J = 6.8 Hz, 3H, *H10*/*H11*), 1.25 (d, J = 6.8 Hz, 3H, *H10*/*H11*), 1.06 (d, J = 6.8 Hz, 3H, *H13*/*H14*), 1.04 (d, J = 6.8 Hz, 3H, *H13*/*H14*).

^{13}C NMR (100 MHz, C_6D_6): δ 150.84 (*C6*), 121.58 (*C5*), 91.97 (*C3*), 88.66 (*C1*), 64.67 (*C8*), 48.45 (*C7*), 45.56 (*C4*), 32.50 (*C9*), 30.76 (*C12*), 24.55 (*C10*/*C11*), 22.14 (*C10*/*C11*), 21.78 (*C13*/*C14*), 21.59 (*C13*/*C14*), 10.65 (*C2*).

HRMS (ESI+): *m/z* calculated for $[^{191}IrC_{22}H_{32}]^+$ 487.2110, found 487.2107.

[Cp*Ir(η^4-1,4-diisopropylbenzene)] (1l-d$_4$)

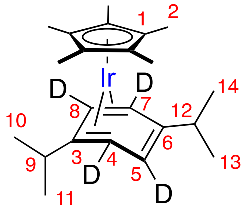

Complex **1l-d$_4$** was prepared according to the general procedure using [Cp*Ir(η^6-1,4-diisopropylbenzene-d$_4$)][BF_4]$_2$ (27.5 mg, 0.041 mmol) and $CoCp_2$ (15.6 mg, 0.082 mmol) to afford [Cp*Ir(η^4-1,4-diisopropyl-benzene-d$_4$)] as a brown solid (98%, 19.8 mg, 0.040 mmol). ^{1}H NMR analysis of the product showed 97% deuteration at the arene ring positions (*C4, C5, C7* and *C8*).

^{1}H NMR (500 MHz, C_6D_6): δ 2.26 (app. sept., J = 6.8 Hz, 1H, *H9*), 2.08 (app. sept., J = 6.8 Hz, 1H, *H12*), 1.86 (s, 15H, *H2*), 1.29 (d, J = 6.8 Hz, 3H, *H10*/*H11*), 1.25 (d, J = 6.8 Hz, 3H, *H10*/*H11*), 1.07 (d, J = 6.8 Hz, 3H, *H13*/*H14*), 1.05 (d, J = 6.8 Hz, 3H, *H13*/*H14*).

^{13}C NMR (126 MHz, C_6D_6): δ 150.61 (*C6*), 121.43 (t, *C5*), 91.80 (*C3*), 88.58 (*C1*), 64.33 (t, *C8*), 47.97 (t, *C7*), 45.06 (t, *C4*), 32.39 (*C9*), 30.67 (*C12*), 24.56 (*C10*/*C11*), 22.13 (*C10*/*C11*), 21.78 (*C13*/*C14*), 21.61 (*C13*/*C14*), 10.70 (*C2*).

HRMS (ESI+): *m/z* calculated for $[^{191}IrC_{22}H_{28}D_4]^+$ 491.2361, found 491.2347.

[Cp*Ir(η^4-1,4-diisopropylbenzene)] (1l-d$_2$)

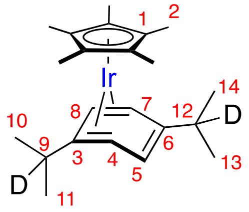

Complex **1l-d$_2$** was prepared according to the general procedure using [Cp*Ir(η^6-1,4-diisopropylbenzene-d$_2$)][BF_4]$_2$ (29.5 mg, 0.044 mmol) and $CoCp_2$ (16.8 mg, 0.089 mmol) to afford [Cp*Ir(η^4-1,4-diisopropyl-benzene-d$_2$)] as an orange solid (86%, 18.8 mg, 0.038 mmol). ^{1}H NMR analysis of the product showed 91% deuteration at the benzylic positions.

^{1}H NMR (500 MHz, C_6D_6): δ 5.76 (dd, J = 4.8, 1.0 Hz, 1H, *H8*), 5.11 (dd, J = 4.5, 2.3 Hz, 1H, *H5*), 3.02 (dd, J = 4.5, 1.0 Hz, 1H, *H4*), 2.93 (dd, J = 4.8, 2.3 Hz, 1H, *H7*), 1.86 (s, 15H, *H2*), 1.29 (s, 3H, *H10*/*H11*), 1.24 (s, 3H, *H10*/*H11*), 1.06 (s, 3H, *H13*/*H14*), 1.04 (s, 3H, *H13*/*H14*).

^{13}C NMR (126 MHz, C_6D_6): δ 150.83 (*C6*), 121.87 (*C5*), 91.93 (*C3*), 88.68 (*C1*), 64.67 (*C8*), 48.43 (*C7*), 45.58 (*C4*), 32.05 (t, *C9*), 30.31 (t, *C12*), 24.44 (*C10*/*C11*), 22.02 (*C10*/*C11*), 21.65 (*C13*/*C14*), 21.47 (*C13*/*C14*), 10.63 (*C2*).

HRMS (ESI+): *m/z* calculated for $[^{191}IrC_{22}H_{32}D_2]^+$ 491.2392, found 491.2347.

[Cp*Ir(η^4-4-isopropyltoluene)] (1m)

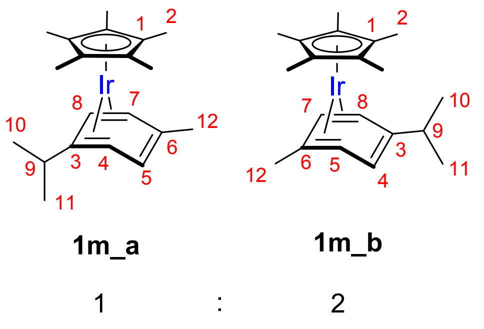

Complex **1m** was prepared according to the general procedure using [Cp*Ir(η^6-4-isopropyltoluene)]$[BF_4]_2$ (50.0 mg, 0.079 mmol) and $CoCp_2$ (29.8 mg, 0.158 mmol) to afford [Cp*Ir(η^4-isopropyltoluene)] as a yellow oil (87%, 31.7 mg, 0.069 mmol). The product was obtained as a mixture of two regioisomers.

Regioisomer **1m_a**: ^{1}H NMR (500 MHz, C_6D_6): δ 5.78 (d, *J* = 4.7 Hz, 1H, *H8*), 5.17-5.16 (m, 1H, *H5*), 3.02 (d, *J* = 4.4 Hz 1H, *H4*), 2.86 (dd, *J* = 4.7, 2.2 Hz, 1H, *H7*), 2.24 (app. sept., *J* = 6.8 Hz, 1H, *H9*), 1.85 (s, 15H, *H2*), 1.60 (d, *J* = 1.6 Hz, 3H, *H12*), 1.30 (d, *J* = 6.9 Hz, 3H, *H10*/*H11*), 1.26 (d, *J* = 6.8 Hz, 3H, *H10*/*H11*).

^{13}C NMR (126 MHz, C_6D_6): δ 139.41 (*C6*), 125.44 (*C5*), 91.75 (*C3*), 88.78 (*C1*), 64.72 (*C8*), 51.27 (*C7*), 45.81 (*C4*), 32.47 (*C9*), 24.65 (*C10*/*C11*), 22.22 (*C10*/*C11*), 18.09 (*C12*), 10.70 (*C2*).

Regioisomer **1m_b**: ^{1}H NMR (500 MHz, C_6D_6): δ 5.66 (d, *J* = 4.6 Hz, 1H, *H7*), 5.13-5.12 (m, 1H, *H4*), 2.98 (dd, *J* = 4.7, 2.2 Hz, 1H, *H5*), 2.91 (d, *J* = 4.4 Hz 1H, *H8*), 2.10 (br. s, 4H, *H9*+*H12*), 1.83 (s, 15H, *H2*), 1.06 (d, *J* = 6.7 Hz, 3H, *H10*/*H11*), 1.05 (d, *J* = 6.7 Hz, 3H, *H10*/*H11*).

^{13}C NMR (126 MHz, C_6D_6): δ 150.52 (*C3*), 121.86 (*C4*), 88.61 (*C1*), 79.17 (*C6*), 68.41 (*C7*), 48.65 (*C5*), 48.37 (*C8*), 30.87 (*C9*), 21.72 (*C10*/*C11*), 21.48 (*C10*/*C11*), 19.43 (*C12*), 10.45 (*C2*).

HRMS (ESI+): *m/z* calculated for $[^{191}IrC_{20}H_{28}]^+$ 459.1797, found 459.1789.

5. Procedure for *ortho*-C-H activation of η^4-arene complexes

In a glovebox, a 4 mL vial equipped with a Teflon-lined screw cap was charged with a hexane (2 mL) solution of [Cp*Ir(η^4-arene)] (0.044-0.119 mmol). Four eq. of trimethylphosphine (0.177-0.502 mmol) was then added to the reaction and the mixture was heated at 100 °C on a heating block. Upon cooling, the mixture was passed through glass wool and the filtrate was dried under vacuum to afford the product as a yellow/brown residue. Product ratios were calculated from relative integrals in their respective ^{1}H NMR spectrum.

Thermolysis of 1a with PMe_3 to form [Cp*Ir(PMe_3)(H)(2-isopropylphenyl)] (2a)

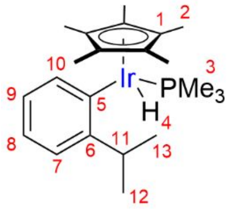

Complex **2a** was prepared according to the general procedure using **1a** (31.5 mg, 0.070 mmol) and PMe_3 (28.6 µL, 0.281 mmol) in hexane (2 mL) with a reaction time of 1 h. The product [Cp*Ir(PMe_3)(H)(2-isopropyl-phenyl)] was isolated as an orange-brown solid (99%, 36.8 mg, 0.070 mmol).

^{1}H NMR (500 MHz, C_6D_6): δ 7.59 (d, J = 7.6 Hz, 1H, *H10*), 7.35 (d, J = 7.5 Hz, 1H, *H7*), 7.20 (t, J = 7.3 Hz, 1H, *H8*), 6.92 (t, J = 7.2 Hz, 1H, *H9*), 4.02 (app. sept, J = 6.9 Hz, 1H, *H11*), 1.78 (s, 15H, *H2*), 1.49 (d, J = 7.0 Hz, 3H, *H12*), 1.40 (d, J = 6.9 Hz, 3H, *H13*), 1.06 (d, $J_{P\text{-}H}$ = 10.0 Hz, 9H, *H3*), -16.68 (d, $J_{P\text{-}H}$ = 40.4 Hz, 1H, *H4*).

^{13}C NMR (126 MHz, C_6D_6): δ 156.41 (*C6*), 143.72 (d, $J_{P\text{-}C}$ = 7.3 Hz, *C10*), 137.70 (d, $J_{P\text{-}C}$ = 11.1 Hz, *C5*), 124.37 (*C9*), 123.96 (*C7*), 122.38 (*C8*), 92.46 (d, $J_{P\text{-}C}$ = 3.3 Hz, *C1*), 38.89 (*C11*), 25.20 (*C12*), 25.01 (*C13*), 19.11 (d, $J_{P\text{-}C}$ = 38.2 Hz, *C3*), 10.45 (*C2*).

^{31}P NMR (202 MHz, C_6D_6): δ -41.40 (s).

HRMS (ASAP): *m/z* calculated for $[^{191}IrC_{22}H_{35}P]^+$ 521.2082, found 521.2076.

Thermolysis of 1a with PPh_3 to form [Cp*Ir(PPh_3)(H)(2-isopropylphenyl)] (2a-ph)

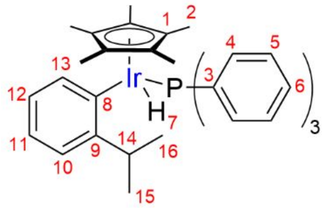

Thermolysis of **1a** was conducted according to the general procedure using one equivalent of triphenylphosphine instead of trimethylphosphine. A mixture of **1a** (25.1 mg, 0.056 mmol) and PPh_3 (14.9 mg, 0.057 mmol) in hexane (2 mL) were heated at 100 °C for 1 h. The crude product mixture was isolated as a dark brown residue (39.3 mg). The yield of **2a-ph** (67%) was determined by ^{1}H NMR spectroscopy, using HMDSO (5.0 µL) as an internal standard. Suitable crystals were grown in cold pentane for single crystal XRD analysis. ^{31}P NMR

spectroscopy revealed the presence of residual PPh_3 as well as two unidentified phosphine species, also noted in the ^1H and ^{13}C NMR spectra.

PPh_3: ^1H NMR (500 MHz, C_6D_6): 7.40-7.37 (m), 7.09-6.96 (m).

^{13}C NMR (126 MHz, C_6D_6): 138.05 (d), 134.19 (d), 128.87 (d), 128.80 (s).

^{31}P NMR (202 MHz, C_6D_6): -5.31 (s).

^1H NMR (500 MHz, C_6D_6): δ 7.64 (dd, J = 7.6, 1.1 Hz, 1H, *H13*), 7.28-7.22 (m, 7H, *H4*+*H10*), 7.09-6.96 (overlapped with PPh_3, 10H, *H5*+*H6*+*H11*), 6.61 (dt, J = 7.3, 1.6 Hz, 1H, *H12*), 3.85 (app. sept, J = 6.8 Hz, 1H, *H14*), 1.64 (d, J = 1.5 Hz, 15H, *H2*), 1.42 (d, J = 7.0 Hz, 3H, *H15*), 0.81 (d, J = 6.8 Hz, 3H, *H16*), -15.95 (d, $J_{P\text{-}H}$ = 41.7 Hz, 1H, *H7*).

^{13}C NMR (126 MHz, C_6D_6): δ 156.12 (*C9*), 144.03 (d, $J_{P\text{-}C}$ = 6.2 Hz, *C13*), 136.35 (d, $J_{P\text{-}C}$ = 53.5 Hz, *C3*), 135.08 (d, $J_{P\text{-}C}$ = 10.2 Hz, *C8*), 134.23 (d, $J_{P\text{-}C}$ = 10.4 Hz, *C4*), 129.44 (d, $J_{P\text{-}C}$ = 1.8 Hz, *C6*), 127.66 (*C5*), 124.90 (*C10*), 124.04 (*C12*), 122.49 (*C11*), 93.61 (d, $J_{P\text{-}C}$ = 2.8 Hz, *C1*), 38.40 (*C14*), 25.65 (*C15*), 23.95 (*C13*), 10.10 (*C2*).

^{31}P NMR (202 MHz, C_6D_6): δ 11.48 (s).

HRMS (ESI+): *m/z* calculated for $[^{191}IrC_{28}H_{30}P]^+$ (M – C_9H_{12}) 588.1691, found 588.1629.

Thermolysis of 1a without added ligand.

Thermolysis of **1a** was conducted according to the general procedure without using an added ligand. A mixture of **1a** (29.0 mg, 0.0648 mmol) in *n*-hexane (0.50 mL) was heated at 100 °C for 48 h. Evaporation of the resulting solution in vacuum gave dark brown viscous oil, which was found to be a mixture of several diiridium species according to NMR and MS data.

Partial ^1H NMR (400 MHz, C_6D_6): δ 7.98 (d, J 7.2 Hz, 1H), 7.92 (d, J 7.3 Hz, 1H), 5.43 (m, 1H), 5.09 (t, J 6.9 Hz, 1H), 4.87 (d, J 6.7 Hz, 1H).

HRMS (ASAP) m/z: calculated for $[^{191}Ir_2C_{29}H_{39}]^+$ (e.g. $[(Cp^*Ir)_2(C_9H_{12})\text{-}3H]$) 769.2264. found 769.2267.

HRMS (nanoESI) m/z: calculated for $[^{191}Ir_2C_{29}H_{37}]^+$ (e.g. $[(Cp^*Ir)\ _2(C_9H_{12})\text{-}5H]\ ^+$) 767.2102, found 767.2104.

Thermolysis of 1b with PMe_3 to form [Cp*Ir(PMe_3)(H)(2-*sec*-butylphenyl)] (2b)

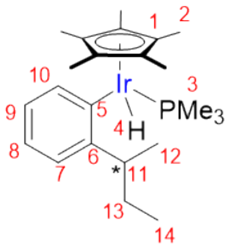

Complex **2b** was prepared according to the general procedure using **1b** (55.4 mg, 0.120 mmol) and PMe_3 (49.5 µL, 0.480 mmol) in hexane (2.4 ml) with a reaction time of 2 h. The product was isolated as dark brown oil (90%, 58.3 mg, 0.108 mmol). [Cp*Ir(PMe_3)(H)(2-sec-butylphenyl)] (**2b**) was identified as the major product (93%) obtained as two diastereomers. The remaining products are presumably comprised of [Cp*Ir(PMe_3)(H)(1-methyl-1-ethyl-benzyl)] (four isomers) and a number of other hydride-containing complexes.

^{1}H NMR (500 MHz, C_6D_6): δ 7.60 (d, J = 7.6 Hz, 1H+1H, *H10**a*** + *H10**b***), 7.25 (d, J = 7.7 Hz, 1H+1H, *H7**a*** + *H7**b***), 7.17 (t, merged with the residual protons of C_6D_6, J = 7.2 Hz, 1H+1H, *H9**a*** + *H9**a***), 6.90 (dt and dt, J = 7.2, 1.7 Hz, 1H+1H, *H8**a*** + *H8**b***), 3.71 (m, 1H, *H11**a**/H11**b***), 3.63 (m, 1H, *H11**a**/H11**b***), 1.91-1.82 (m, 2H+1H, *H13**a**+H13**b***), 1.78 (d, J = 1.4 Hz, 15H, *H2**a**/H2**b***), 1.77 (d, J = 1.4 Hz, 15H, *H2**a**/H2**b***), 1.61-1.54 (m, 1H, *H13**a**+H13**b***), 1.43 (d, J = 7.0 Hz, 3H, *H12**a**/H12**b***), 1.35 (d, J = 6.8 Hz, 3H, *H12**a**/H12**b***), 1.18 (t, J = 7.4 Hz, 3H, *H14**a**/H14**b***), 1.16 (t, J = 7.5 Hz, 3H, *H14**a**/H14**b***), 1.07 (d, $J_{P\text{-}H}$ = 9.8 Hz, 9H, *H3**a**/H3**b***), 1.06 (d, $J_{P\text{-}H}$ = 9.7 Hz, 9H, *H3**a**/H3**b***), -16.69 (d, $J_{P\text{-}H}$ = 40.0 Hz, 1H, *H4**a**/H4**b***), -16.74 (d, $J_{P\text{-}H}$ = 40.1 Hz, 1H, *H4**a**/H4**b***).

^{13}C NMR (126 MHz, C_6D_6): δ 156.17 (*C6**a**/C6**b***), 155.82 (*C6**a**/C6**b***), 144.86 (d, $J_{P\text{-}C}$ = 7.1 Hz, *C10**a**+C10**b***), 138.16 (d, $J_{P\text{-}C}$ = 11.3 Hz, *C5**a**/C5**b***), 137.87 (d, $J_{P\text{-}C}$ = 11.3 Hz, *C5**a**/C5**b***), 124.63 (*C9**a**/C9**b***), 124.39 (*C9**a**/C9**b***), 124.34 (*C8**a**/C8**b***), 124.30 (*C8**a**/C8**b***), 122.32 (*C9**a**/C9**b***), 122.20 (*C9**a**/C9**b***), 92.50 (d, $J_{P\text{-}C}$ = 3.4 Hz, *C1**a**/C1**b***), 92.43 (d, $J_{P\text{-}C}$ = 3.4 Hz, *C1**a**/C1**b***), 46.15 (*C11**a**/C11**b***), 45.21 (*C11**a**/C11**b***), 31.81 (*C13**a**/C13**b***), 31.64 (*C13**a**/C13**b***), 21.60 (*C12**a**/C12**b***), 21.50 (*C12**a**/C12**b***), 19.16 (d, $J_{P\text{-}C}$ = 38.2 Hz, *C3**a**/C3**b***), 19.01 (d, $J_{P\text{-}C}$ = 38.3 Hz, *C3**a**/C3**b***), 13.50 (*C14**a**/C14**b***), 13.21 (*C14**a**/C14**b***), 10.44 (*C2**a**+C2**b***).

^{31}P NMR (202 MHz, C_6D_6): δ -41.48 (d, $J_{P\text{-}H}$ = 9.5 Hz), -41.73 (d, $J_{P\text{-}H}$ = 7.0 Hz).

HRMS (ASAP): *m/z* calculated for $[^{191}IrC_{23}H_{37}P]^+$ 535.2239, found 535.2224.

Thermolysis of 1c with PMe_3 to form [Cp*Ir(PMe_3)(H)(2-(3-pentyl)phenyl)] (2c)

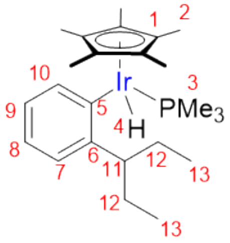

Complex **2c** was prepared according to the general procedure using **1c** (52.8 mg, 0.111 mmol) and PMe_3 (45.8 µL, 0.444 mmol) in hexane (2.2 ml) with a reaction time of 2 h. The product was isolated as a dark brown oil (91%, 56.0 mg, 0.070 mmol). The complex [Cp*Ir(PMe_3)(H)(2-(3-pentyl)phenyl)] (**2c**) was identified as the major product (91%). The remaining products are tentatively identified as [Cp*Ir(PMe_3)(H)(1,1-diethylbenzyl)] (two diastereomers) and number of other hydride-containing complexes.

^{1}H NMR (500 MHz, C_6D_6): δ 7.64 (d, J = 7.4 Hz, 1H, *H10*), 7.20-7.13 (m, merged with the residual protons of C_6D_6 and *H9*, 1H, *H7*), 7.20-7.13 (m, merged with the residual protons of C_6D_6 and *H7*, 1H, *H9*), 6.90 (t, J = 6.5 Hz, 1H, *H8*), 3.50 (pent., J = 6.3 Hz, 1H, *H11*), 1.91-1.78 (m, merged with *H2*, 4H, *H12*), 1.78 (s, 15H, *H2*), 1.13 (t, J = 7.4 Hz, 3H, *H13*), 1.08 (t, merged with *H3,* J = 7.9 Hz, 9H, *H3*), 1.06 (d, $J_{P\text{-}H}$ = 9.9 Hz, 9H, *H3*), -16.69 (d, $J_{P\text{-}H}$ = 39.2 Hz, 1H, *H4*).

^{13}C NMR (126 MHz, C_6D_6): δ 152.88 (*C6*), 144.17 (d, $J_{P\text{-}C}$ = 7.3 Hz, *C10*), 138.86 (d, $J_{P\text{-}C}$ = 11.7 Hz, *C5*), 125.50 (*C7*), 124.21 (*C8*), 121.75 (*C9*), 92.44 (d, $J_{P\text{-}C}$ = 2.9 Hz, *C1*), 51.06 (*C11*), 28.05 (*C12*), 27.18 (*C12*), 19.03 (d, $J_{P\text{-}C}$ = 38.3 Hz, *C3*), 13.01 (*C13*), 11.72 (*C13*), 10.51 (*C2*).

^{31}P NMR (202 MHz, C_6D_6): δ -41.29 (s).

HRMS (nanoESI+): *m/z* calculated for $[^{191}IrC_{24}H_{39}P]^+$ 549.2390, found 549.2384.

Thermolysis of 1d with PMe_3 to form [Cp*Ir(PMe_3)(H)(2-cyclopentylphenyl)] (2d)

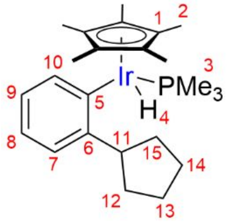

Complex **2d** was prepared according to the general procedure using **1d** (21.0 mg, 0.044 mmol) and PMe_3 (18.0 µL, 0.177 mmol) in hexane (2 mL) with a reaction time of 1 h. The product [Cp*Ir(PMe_3)(H)(2-cyclopentyl-phenyl)] was isolated as a light brown solid (88%, 21.4 mg, 0.039 mmol).

^{1}H NMR (500 MHz, C_6D_6): δ 7.59 (dd, J = 7.5, 1.0 Hz, 1H, *H10*), 7.35 (dd, J = 7.7, 1.1 Hz, 1H, *H7*), 7.20 (t, J = 7.4 Hz, 1H, *H8*), 6.92 (dt, J = 7.2, 1.5 Hz, 1H, *H9*), 4.07 (app. quin, J = 8.7 Hz, 1H, *H11*), 2.37-2.32 (m, 1H, *H12/H15*), 2.18-2.13 (m, 1H, *H12/H15*), 1.96-1.84 (m, 5H, *H12-H15*), 1.78 (d, J = 1.4 Hz, 15H, *H2*), 1.59-1.51 (m, 1H, *H12/H15*), 1.06 (d, $J_{P\text{-}H}$ = 9.9 Hz, 9H, *H3*), -16.67 (d, $J_{P\text{-}H}$ = 40.0 Hz, 1H, *H4*).

^{13}C NMR (126 MHz, C_6D_6): δ 154.21 (*C6*), 143.59 (d, $J_{P\text{-}C}$ = 7.4 Hz, *C10*), 139.03 (d, $J_{P\text{-}C}$ = 11.2 Hz, *C5*), 124.64 (*C9*), 124.26 (*C7*), 122.43 (*C8*), 92.49 (d, $J_{P\text{-}C}$ = 3.3 Hz, *C1*), 52.19 (*C11*), 36.23 (*C12+C15*), 27.09 (*C13/C14*), 26.49 (*C13/C14*), 19.08 (d, $J_{P\text{-}C}$ = 38.3 Hz, *C3*), 10.43 (*C2*).

^{31}P NMR (202 MHz, C_6D_6): δ -41.77 (s).

HRMS (ESI+): *m/z* calculated for $[^{191}IrC_{24}H_{37}P]^+$ 547.2239, found 547.2227.

Thermolysis of 1e with PMe₃ to form [Cp*Ir(PMe₃)(H)(2-cyclohexylphenyl)] (2e)

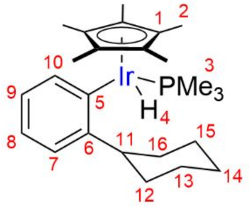

Complex **2e** was prepared according to the general procedure using **1e** (37.0 mg, 0.076 mmol) and PMe_3 (31.4 µL, 0.309 mmol) in hexane (2 mL) with a reaction time of 1 h. The product [Cp*Ir(PMe_3)(H)(2-cyclohexylphenyl)] was isolated as a brown solid (94%, 40.3 mg, 0.071 mmol).

^{1}H NMR (400 MHz, C_6D_6): δ 7.59 (d, J = 6.7 Hz, 1H, *H10*), 7.30 (d, J = 6.8 Hz, 1H, *H7*), 7.19 (t, J = 6.9 Hz, 1H, *H8*), 6.91 (t, J = 6.9 Hz, 1H, *H9*), 3.55 (app. t, J = 11.1 Hz, 1H, *H11*), 2.12-1.24 (m, 10H, *H12-16*), 1.79 (s, 15H, *H2*), 1.05 (d, $J_{P\text{-}H}$ = 9.9 Hz, 9H, *H3*), -16.74 (d, $J_{P\text{-}H}$ = 40.5 Hz, 1H, *H4*).

^{13}C NMR (100 MHz, C_6D_6): δ 155.30 (*C6*), 143.78 (d, $J_{P\text{-}C}$ = 7.3 Hz, *C10*), 138.22 (d, $J_{P\text{-}C}$ = 11.0 Hz, *C5*), 124.96 (*C9*), 124.39 (*C7*), 122.23 (*C8*), 92.44 (d, $J_{P\text{-}C}$ = 2.9 Hz, *C1*), 50.10 (*C11*), 35.45 (*C12/C16*), 35.59 (*C12/C16*), 28.49 (*C13/C15*), 28.33 (*C13/C15*), 27.33 (*C14*), 19.09 (d, $J_{P\text{-}C}$ = 38.1 Hz, *C3*), 10.47 (*C2*).

^{31}P NMR (162 MHz, C_6D_6): δ -41.78 (s).

HRMS (ESI+): *m/z* calculated for $[^{191}IrC_{13}H_{25}P]^+$ (M – $C_{12}H_{15}$) 403.1300, found 403.1292.

Thermolysis of 1f with PMe₃ to form [Cp*Ir(PMe₃)(H)(2-ethylphenyl)] (2f)

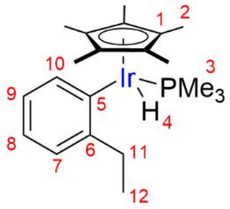

Thermolysis of **1f** (48.0 mg, 0.111 mmol) was conducted according to the general procedure using PMe_3 (45.8 µL, 0.451 mmol) in hexane (2 mL) with a reaction time of 1 h to yield a brown residue (93%, 50.9 mg, 0.103 mmol). The complex [Cp*Ir(PMe_3)(H)(2-ethylphenyl)] (**2f**) was identified as the major product (75%). The remaining products are tentatively identified as the *meta* and *para* isomers (8%), [Cp*Ir(PMe_3)(H)(1-methylbenzyl)] (two diastereomers, 8%) and a number of trace hydride-containing complexes.

^{1}H NMR (500 MHz, C_6D_6): δ 7.58 (dd, *J* = 7.5, 1.4 Hz, 1H, *H10*), 7.36 (dd, *J* = 7.5, 1.6 Hz, 1H, *H7*), 7.16 (m, merged with the residual protons of C_6D_6, 1H, *H9*), 6.93 (dt, *J* = 7.3, 1.6 Hz, 1H, *H8*), 3.16 (dq, *J* = 13.5, 7.5 Hz, 1H, *H11*), 2.96 (dq, *J* = 13.5, 7.5 Hz, 1H, *H11*), 1.77 (dd, *J* = 1.9, 0.7 Hz, 15H, *H2*), 1.43 (t, *J* = 7.5 Hz, 3H, *H12*), 1.07 (d, $J_{P\text{-}H}$ = 9.9 Hz, 9H, *H3*), -16.74 (d, $J_{P\text{-}H}$ = 39.7 Hz, 1H, *H4*).

^{13}C NMR (126 MHz, C_6D_6): δ 152.22 (*C6*), 143.86 (d, $J_{P\text{-}C}$ = 6.9 Hz, *C10*), 137.34 (d, $J_{P\text{-}C}$ = 11.7 Hz, *C5*), 126.66 (*C7*), 124.41 (*C9*), 122.16 (*C8*), 92.49 (d, $J_{P\text{-}C}$ = 3.1 Hz, *C1*), 36.49 (*C11*), 19.01 (d, $J_{P\text{-}C}$ = 38.3 Hz, *C3*), 16.55 (*C12*), 10.41 (*C2*).

^{31}P NMR (202 MHz, C_6D_6): δ -42.16 (s).

HRMS (ASAP) m/z: calculated for $[^{191}IrC_{21}H_{33}P]^+$ 507.1926, found 507.1938.

Thermolysis of 1g with PMe_3 to form [Cp*Ir(PMe_3)(H)(2-*n*-propylphenyl)] (2g)

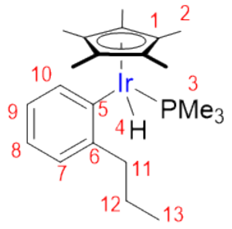

Complex **2g** was prepared according to the general procedure using **1g** (46.3 mg, 0.103 mmol) and PMe_3 (42.7 µL, 0.414 mmol) in hexane (2.1 ml) with a reaction time of 2 h. The product was isolated as dark brown oil (91%, 49.5 mg, 0.095 mmol). The complex [Cp*Ir(PMe_3)(H)(2-*n*-propylphenyl)] (**2g**) was identified as the major product (79%). The remaining products were tentatively identified as [Cp*Ir(PMe_3)(H)(1-ethylbenzyl)] (two diastereomers) and a number of other hydride-containing complexes.

^{1}H NMR (500 MHz, C_6D_6): δ 7.59 (dd, *J* = 7.5, 1.4 Hz, 1H, *H10*), 7.33 (dd, *J* = 7.5, 1.6 Hz, 1H, *H7*), 7.13 (dt, *J* = 7.3, 1.3 Hz, 1H, *H9*), 6.92 (dt, *J* = 7.3, 1.6 Hz, 1H, *H8*), 3.07 (ddd, *J* = 12.5, 10.8, 5.5 Hz, 1H, *H11*), 2.89 (ddd, *J* = 12.6, 10.5, 5.6 Hz, 1H, *H11*), 1.92 (m, 1H, *H12*), 1.79 (*overlaps with H2,* m, 1H, *H12*), 1.78 (dd, *J* = 1.9, 0.6 Hz, 15H, *H2*), 1.19 (t, *J* = 7.3 Hz, 3H, *H13*), 1.07 (d, $J_{P\text{-}H}$ = 10.0 Hz, 9H, *H3*), -16.76 (d, $J_{P\text{-}H}$ = 39.5 Hz, 1H, *H4*).

^{13}C NMR (126 MHz, C_6D_6): δ 150.58 (*C6*), 143.94 (d, $J_{P\text{-}C}$ = 6.9 Hz, *C10*), 137.51 (d, $J_{P\text{-}C}$ = 11.5 Hz, *C5*), 127.46 (*C7*), 124.41 (*C8*), 121.91 (*C9*), 92.48 (d, $J_{P\text{-}C}$ = 3.3 Hz, *C1*), 46.08 (*C11*), 25.06 (s, *C12*), 18.99 (d, $J_{P\text{-}C}$ = 38.2 Hz, *C3*), 15.21 (*C13*), 10.43 (*C2*).

^{31}P NMR (202 MHz, C_6D_6): δ -42.04 (s).

HRMS (nanoESI+): *m/z* calculated for $[^{193}IrC_{22}H_{35}P]^+$ 523.2100, found 523.2079.

Thermolysis of 1h with PMe_3 to form [Cp*Ir(PMe_3)(H)(2-*n*-butylphenyl)] (2h)

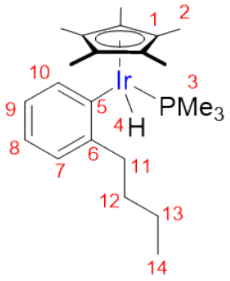

Complex **2h** was prepared according to the general procedure using **1h** (53.8 mg, 0.117 mmol) and PMe_3 (48.1 µL, 0.447 mmol) in hexane (2.3 ml) with a reaction time of 2 h. The product was isolated as dark brown oil (93%, 58.3 mg, 0.108 mmol). The complex [Cp*Ir(PMe_3)(H)(2-*n*-butylphenyl)] (**2h**) was identified as the major product (77%). The remaining products were tentatively identified as [Cp*Ir(PMe_3)(H)(1-propylbenzyl)] (two diastereomers) and a number of other hydride-containing complexes.

^{1}H NMR (500 MHz, C_6D_6): δ 7.59 (dd, J = 7.5, 1.1 Hz, 1H, *H10*), 7.34 (dd, J = 7.5, 1.2 Hz, 1H, *H7*), 7.14 (dt, merged with the residual protons of C_6D_6, J = 7.2, 1.0 Hz, 1H, *H9*), 6.92 (dt, J = 7.4, 1.4 Hz, 1H, *H8*), 3.10 (app dt, J = 12.1, 5.3 Hz, 1H, *H11*), 2.89 (ddd, J = 12.1, 11.0, 5.5 Hz, 1H, *H11*), 1.84 (m, 1H, *H12*), 1.78 (d, J = 1.4 Hz, 15H, *H2*), 1.77 (m, overlaps with *H2,* 1H, *H12*), 1.64-1.56 (m, 2H, *H13*), 1.09 (t, J = 7.4 Hz, 3H, *H14*), 1.08 (d, $J_{P\text{-}H}$ = 9.9 Hz, 9H, *H3*), -16.77 (d, $J_{P\text{-}H}$ = 39.4 Hz, 1H, *H4*).

^{13}C NMR (126 MHz, C_6D_6): δ 150.77 (*C6*), 143.98 (d, $J_{P\text{-}C}$ = 6.6 Hz, *C10*), 137.47 (d, $J_{P\text{-}C}$ = 11.5 Hz, *C5*), 127.43 (*C7*), 124.38 (*C8*), 121.94 (*C9*), 92.50 (d, $J_{P\text{-}C}$ = 3.1 Hz, *C1*), 43.54 (*C11*), 34.41 (*C12*), 23.90 (*C13*), 19.00 (d, $J_{P\text{-}C}$ = 38.1 Hz, *C3*), 14.68 (*C14*), 10.43 (*C2*).

^{31}P NMR (202 MHz, C_6D_6): δ -42.13 (d, J = 5.6 Hz).

HRMS (nanoESI+): *m/z* calculated for $[^{191}IrC_{23}H_{37}P]^+$ 535.2233, found 535.2221.

Thermolysis of 1i with PMe_3 to form [Cp*Ir(PMe_3)(H)(2-*iso*-butylphenyl)] (2i)

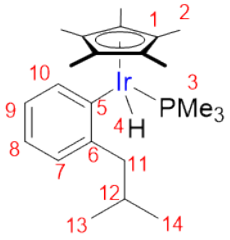

Complex **2i** was prepared according to the general procedure using **1i** (51.6 mg, 0.112 mmol) and PMe_3 (46.1 µL, 0.447 mmol) in hexane (2.2 ml) with a reaction time of 2 h. The product was isolated as dark brown oil (99%, 59.4 mg, 0.110 mmol). The complex [Cp*Ir(PMe_3)(H)(2-iso-butylphenyl)] (**2i**) was identified as the major product (72%). The remaining products were tentatively identified as [Cp*Ir(PMe_3)(H)(1-isopropylbenzyl)] (diastereomers) and a number of other hydride-containing complexes.

^{1}H NMR (500 MHz, C_6D_6): δ 7.60 (dd, J = 7.5, 1.3 Hz, 1H, *H10*), 7.25 (dd, J = 7.5, 1.5 Hz, 1H, *H7*), 7.11 (dt, J = 7.2, 1.2 Hz, 1H, *H9*), 6.91 (dt, J = 7.2, 1.5 Hz, 1H, *H8*), 2.93 (dd, J = 12.5, 6.5 Hz, 1H, *H11*), 2.71 (dd, J = 12.5, 7.9 Hz, 1H, *H11*), 2.31 (sept, J = 6.7 Hz, 1H, *H12*), 1.77 (d, J =

1.4 Hz, 15H, *H2*), 1.17 (d, J = 6.6 Hz, 3H, *H13/H14*), 1.17 (d, J = 6.6 Hz, 3H, *H13/H14*), 1.06 (d, $J_{P\text{-}H}$ = 9.9 Hz, 9H, *H3*), -16.78 (d, $J_{P\text{-}H}$ = 39.2 Hz, 1H, *H4*).

^{13}C NMR (126 MHz, C_6D_6): δ 149.21 (*C6*), 144.18 (d, $J_{P\text{-}C}$ = 6.4 Hz, *C10*), 137.84 (d, $J_{P\text{-}C}$ = 11.9 Hz, *C5*), 128.89 (*C7*), 124.50 (*C8*), 121.52 (*C9*), 92.55 (d, $J_{P\text{-}C}$ = 3.0 Hz, *C1*), 52.95 (*C11*), 28.61 (*C12*), 23.65 (*C13/C14*), 22.69 (*C13/C14*), 18.89 (d, $J_{P\text{-}C}$ = 38.2 Hz, *C3*), 10.43 (*C2*).

^{31}P NMR (202 MHz, C_6D_6): δ -41.83 (s).

HRMS (ASAP): *m/z* calculated for $[^{191}IrC_{23}H_{37}P]^+$ 535.2233, found 535.2216.

Thermolysis of 1j with PMe$_3$ to form [Cp*Ir(PMe$_3$)(H)(2-*neo*-pentylphenyl)] (2j)

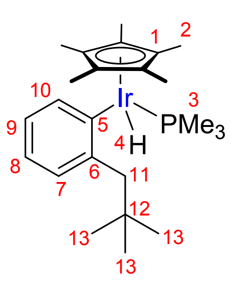

Complex **2j** was prepared according to the general procedure using **1j** (43.6 mg, 0.092 mmol) and PMe$_3$ (37.8 µL, 0.444 mmol) in hexane (1.8 ml) with a reaction time of 4 h. The product was isolated as dark brown oil (87%, 44.0 mg, 0.080 mmol). The complex [Cp*Ir(PMe$_3$)(H)(2-neopentylphenyl)] (**2j**) was identified as the major product (91%). The remaining products were tentatively identified as [Cp*Ir(PMe$_3$)(H)(1-*t*-butylbenzyl)] (two diastereomers) and a number of other hydride-containing complexes.

^{1}H NMR (500 MHz, C_6D_6): δ 7.64 (dd, J = 7.6, 0.9 Hz, 1H, *H10*), 7.42 (d, J = 7.6, 1.5 Hz, 1H, *H7*), 7.10 (dt, J = 7.3, 1.3 Hz, 1H, *H9*), 6.91 (dt, J = 7.3, 1.6 Hz, 1H, *H8*), 3.61 (dd, J = 12.9, 1.1 Hz, 1H, *H11*), 2.77 (d, J = 12.9 Hz, 1H, *H11*), 1.72 (dd, J = 1.8, 0.5 Hz, 15H, *H2*), 1.25 (s, 9H, *H13*), 1.15 (d, $J_{P\text{-}H}$ = 9.9 Hz, 9H, *H3*), -16.64 (d, $J_{P\text{-}H}$ = 39.3 Hz, 1H, *H4*).

^{13}C NMR (126 MHz, C_6D_6): δ 48.62 (*C6*), 144.60 (d, $J_{P\text{-}C}$ = 7.5 Hz, *C10*), 140.15 (d, $J_{P\text{-}C}$ = 10.8 Hz, *C5*), 128.61 (*C7*), 124.59 (*C8*), 121.26 (*C9*), 92.73 (d, $J_{P\text{-}C}$ = 3.1 Hz, *C1*), 54.16 (*C11*), 33.32 (*C12*), 31.16 (*C13*), 19.36 (d, $J_{P\text{-}C}$ = 37.8 Hz, *C3*), 10.28 (*C2*).

^{31}P NMR (202 MHz, C_6D_6): δ -42.45 (d, $J_{P\text{-}H}$ = 9.2 Hz).
HRMS (nanoESI+) *m/z* calculated for $[^{191}IrC_{24}H_{39}P]^+$ 549.2390, found 551.2413.

Thermolysis of 1l with PMe$_3$ to form [Cp*Ir(PMe$_3$)(H)(2,5-diisopropylphenyl)] (2l)

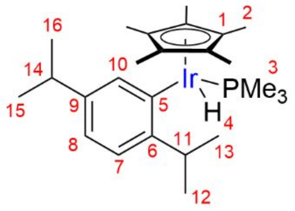

Complex **2l** was prepared according to the general procedure using **1l** (26.0 mg, 0.053 mmol) and PMe$_3$ (21.6 µL, 0.206 mmol) in hexane (2 mL) with a reaction time of 3 h. The product [Cp*Ir(PMe$_3$)(H)(2,5-diisopropylphenyl)] was isolated as a brown solid (98%, 29.3 mg, 0.052 mmol).

^{1}H NMR (500 MHz, C_6D_6): δ 7.48 (d, J = 1.9 Hz, 1H, *H10*), 7.27 (d, J = 7.8 Hz, 1H, *H7*), 7.00 (dd, J = 7.8, 2.2 Hz, 1H, *H8*), 3.98 (app. sept, J = 6.9. Hz, 1H, *H11*), 2.89 (app. sept, J = 6.7 Hz, 1H, *H14*), 1.80 (d, J = 1.5 Hz, 15H, *H2*), 1.48 (d, J = 7.0 Hz, 3H, *H13*), 1.40-1.37 (m, 9H, *H12+H15+H16*), 1.10 (d, $J_{P\text{-}H}$ = 9.9 Hz, 9H, *H3*), -16.69 (d, $J_{P\text{-}H}$ = 40.3 Hz, 1H, *H4*).

^{13}C NMR (126 MHz, C_6D_6): δ 153.84 (*C6*), 142.95 (*C9*), 141.72 (d, $J_{P\text{-}C}$ = 7.4 Hz, *C10*), 137.26 (d, $J_{P\text{-}C}$ = 11.3Hz, *C5*), 123.60 (*C7*), 120.71 (*C8*), 92.53 (d, $J_{P\text{-}C}$ = 3.4 Hz, *C1*), 38.47 (*C11*), 34.15 (*C14*), 25.33 (*C13*), 25.11 (*C12/C15/C16*), 25.05 (*C12/C15/C16*), 24.89 (*C12/C15/C16*), 19.21 (d, $J_{P\text{-}C}$ = 38.2 Hz, *C3*), 10.53 (*C2*).

^{31}P NMR (162 MHz, C_6D_6): δ -41.21 (s).

HRMS (ESI+): *m/z* calculated for $[^{191}IrC_{25}H_{41}P]^+$ 563.2552, found 563.2535.

Thermolysis of 1m with PMe_3 to form [Cp*Ir(PMe_3)(H)(2-^{i}propyl-5-methylphenyl)] (2m-1) and [Cp*Ir(PMe_3)(H)(4-^{i}propyl-benzyl)] (2m-2)

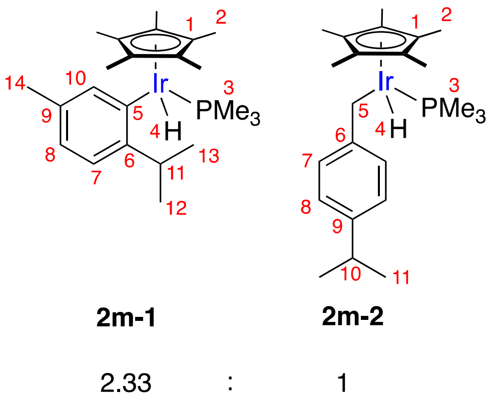

A mixture of complexes **2m-1** and **2m-2** was obtained according to the general procedure using **1m** (31.6 mg, 0.068 mmol) and PMe_3 (28.4 µL, 0.279 mmol) in hexane (2 mL) with a reaction time of 1 h. Removal of the solvent affords a yellow-brown oil, which was subsequently identified as a mixture of [Cp*Ir(PMe_3)(H)(2-^{i}propyl-5-methylphenyl)] and [Cp*Ir(PMe_3)(H)(4-^{i}propylbenzyl)] in a 70:30 ratio (92%, 33.8 mg, 0.063 mmol).

Compound **2m-1**: ^{1}H NMR (500 MHz, C_6D_6): δ 7.44 (s, 1H, *H10*), 7.25 (d, J = 7.7 Hz, 1H, *H7*), 7.00 (d, J = 7.3 Hz, 1H, *H8*), 3.97 (app. sept, J = 6.8 Hz, 1H, *H11*), 2.36 (s, 3H, *H14*), 1.79 (d, J = 0.7 Hz, 15H, *H2*), 1.48 (d, J = 6.9 Hz, 3H, *H12*), 1.40 (d, J = 6.9 Hz, 3H, *H13*), 1.08 (d, $J_{P\text{-}H}$ = 9.9 Hz, 9H, *H3*), -16.69 (d, $J_{P\text{-}H}$ = 40.3 Hz, 1H, *H4*).

^{13}C NMR (126 MHz, C_6D_6): δ 153.44 (*C6*), 144.72 (d, $J_{P\text{-}C}$ = 7.3 Hz, *C10*), 137.52 (d, $J_{P\text{-}C}$ = 11.4 Hz, *C5*), 131.87 (*C9*), 123.63 (*C7*), 123.19 (*C8*), 92.44 (d, $J_{P\text{-}C}$ = 3.4 Hz, *C1*), 38.47 (*C11*), 25.36 (*C12*), 25.10 (*C13*), 21.06 (*C14*), 19.07 (d, $J_{P\text{-}C}$ = 38.3 Hz, *C3*), 10.46 (*C2*).

^{31}P NMR (202 MHz, C_6D_6): δ -41.71 (s).

Compound **2m-2**: ^{1}H NMR (500 MHz, C_6D_6): δ 7.49 (d, J = 7.9 Hz, 2H, *H7*), 7.12 (d, J = 7.9 Hz, 2H, *H8*), 3.12 (app. t, J = 9.8 Hz, 1H, *H5*), 3.00 (dd, J = 11.0, 2.9 Hz, 1H, *H5*), 2.83 (app. sept, J = 6.9 Hz, 1H, *H10*), 1.75 (s, 15H, *H2*), 1.26 (d, J = 6.9 Hz, 6H, *H11*), 1.17 (d, $J_{P\text{-}H}$ = 9.7 Hz, 9H, *H3*), -17.30 (d, $J_{P\text{-}H}$ = 36.6 Hz, 1H, *H4*).

^{13}C NMR (126 MHz, C_6D_6): δ 152.94 (d, $J_{P\text{-}C}$ = 3.3 Hz, *C6*), 142.52 (*C9*), 129.66 (*C7*), 125.37 (*C8*), 91.81 (d, $J_{P\text{-}C}$ = 3.4 Hz, *C1*), 34.10 (*C10*), 24.66 (*C11*), 19.18 (d, $J_{P\text{-}C}$ = 36.4 Hz, *C3*), 10.15 (*C2*), -7.69 (d, $J_{P\text{-}C}$ = 9.3 Hz, *C5*).

^{31}P NMR (202 MHz, C_6D_6): δ -45.15 (s).

HRMS (ESI+): *m/z* calculated for $[^{191}IrC_{23}H_{37}P]^+$ 535.2239, found 535.2234.

6. Procedure for bromide-hydride exchange in 2a

[Cp*Ir(PMe$_3$)(Br)(2-isopropylphenyl)] (2a-br)

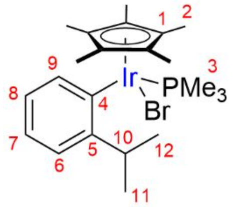

In a glovebox, a Schlenk flask was charged with a bromoform (2.0 mL) solution of **1a** (36.5 mg, 0.070 mmol). The flask was then removed from the glovebox and stirred at room temperature for 3 h. Following this, the solvent was removed *in vacuo* at 30 °C and resultant residue was extracted using hexane (3 × 5 mL) to remove unreacted **1a**. Benzene (3 × 5 mL) was then used to extract the residue and the resultant orange solution was then dried *in vacuo* to afford [Cp*Ir(PMe$_3$)(Br)(2-isopropylphenyl)] (68%, 28.7 mg, 0.048 mmol) as a yellow powder.

Suitable crystals were grown from a hexane solution at -20 °C for XRD analysis.

^{1}H NMR (500 MHz, C_6D_6): δ 7.38 (dd, J = 7.7, 1.7 Hz, 1H, *H6*), 7.35 (dt, J = 7.7, 1.4 Hz, 1H, *H7*), 7.14 (dd, J = 7.7, 1.7 Hz, 1H, *H9*), 6.86 (m, 1H, *H8*), 4.00 (app. sept, J = 6.8 Hz, 1H, *H10*), 1.69 (d, J = 6.6 Hz, 3H, *H12*), 1.43 (d, J = 7.1 Hz, 3H, *H11*), 1.38 (d, J = 2.3 Hz, 15H, *H2*), 1.24 (d, $J_{P\text{-}H}$ = 10.1 Hz, 9H, *H3*).

^{13}C NMR (126 MHz, C_6D_6): δ 158.79 (*C5*), 142.49 (d, $J_{P\text{-}C}$ = 11.1 Hz, *C7*), 137.43 (d, $J_{P\text{-}C}$ = 13.7 Hz, *C4*), 127.46 (*C9*), 123.97 (*C6*), 123.48 (*C7*), 92.47 (d, $J_{P\text{-}C}$ = 2.9 Hz, *C1*), 36.02 (*C10*), 29.48 (*C12*), 25.75 (*C11*), 15.04 (d, $J_{P\text{-}C}$ = 39.2 Hz, *C3*), 8.93 (*C2*).

^{31}P NMR (202 MHz, C_6D_6): δ -38.07 (s).

7. Procedures for kinetic and isotope-labelling studies

Determination of reaction order on [Cp*Ir(η^4-1,4-diisopropylbenzene)] (1l)

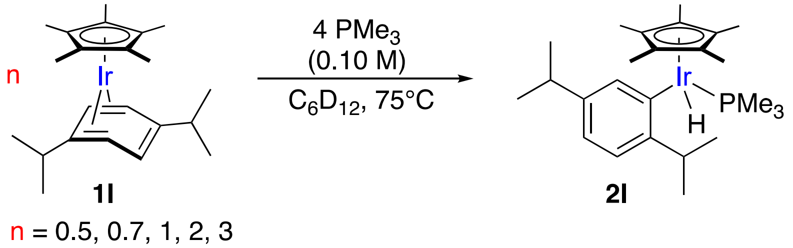

In a glovebox, a J. Young NMR tube was charged with a 0.025, 0.035, 0.050, 0.100 or 0.150 M solution of **1l** in C_6D_{12} (0.5 mL). HMDSO (5.3 µL) was then added as an internal standard followed by PMe_3 (10.2 µL). The J. Young NMR tube was sealed, removed from the glovebox and heated in an oil bath at 75 °C (oil bath temperature). After designated time intervals, the NMR tube was removed from the oil bath, cooled with liquid nitrogen, washed with DCM to remove external oil, after which the ^{1}H NMR spectrum was recorded, and the NMR tube was put back in the oil bath until the next measurement. The reaction order on **1l** was measured using the initial rate of the accumulation of **2l** at the conversion of **1l** less than 15%. Concentrations of **1l** and **2l** were quantified by integrating the signals indicated below against the internal standard (HMDS). Experiments were carried out in triplicate.

Underlined signals were used to determine concentrations.

Starting complex **1l**:

^{1}H NMR (500 MHz, C_6D_{12}): δ 5.61 (1H), 4.71 (1H), 2.79 (1H), 2.74 (1H), 2.20 (1H), 1.89 (15H), 1.85 (1H), 1.20 (6H), 0.85 (6H).

Product **2l**:

^{1}H NMR (500 MHz, C_6D_{12}): δ 7.26 (1H), 6.85 (1H), 6.21 (1H), 3.61 (1H), 2.66 (1H), 1.84 (15H), 1.27 (9H), 1.19 (6H), 1.15 (3H), 1.03 (3H), -16.89 (1H).

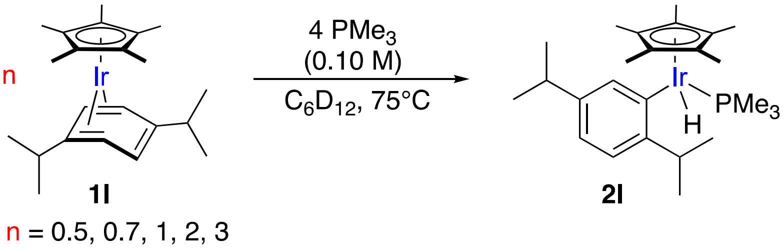

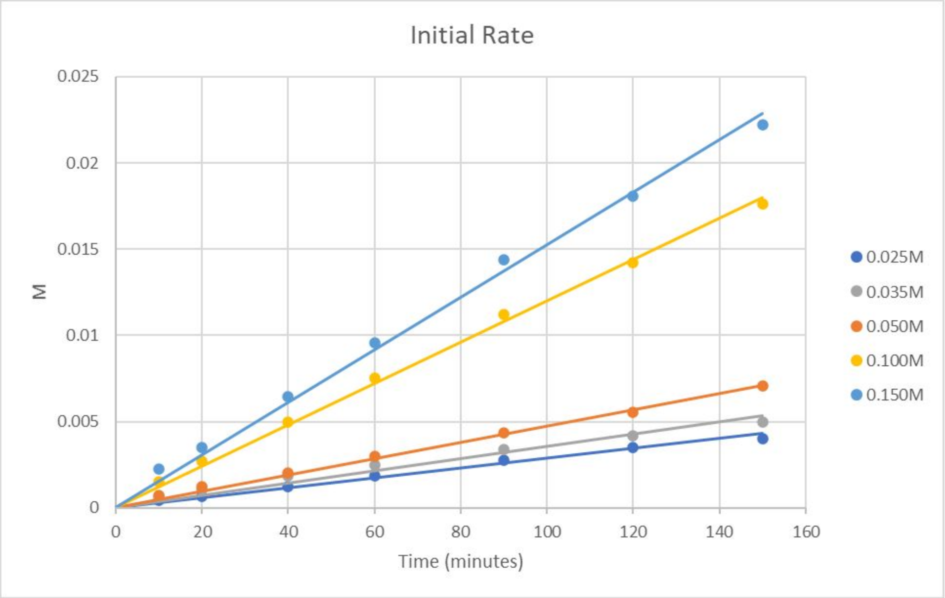

Figure S1. Plot of concentration of product **2l** (moles) against time (minutes) for varying starting concentrations of **1l**.

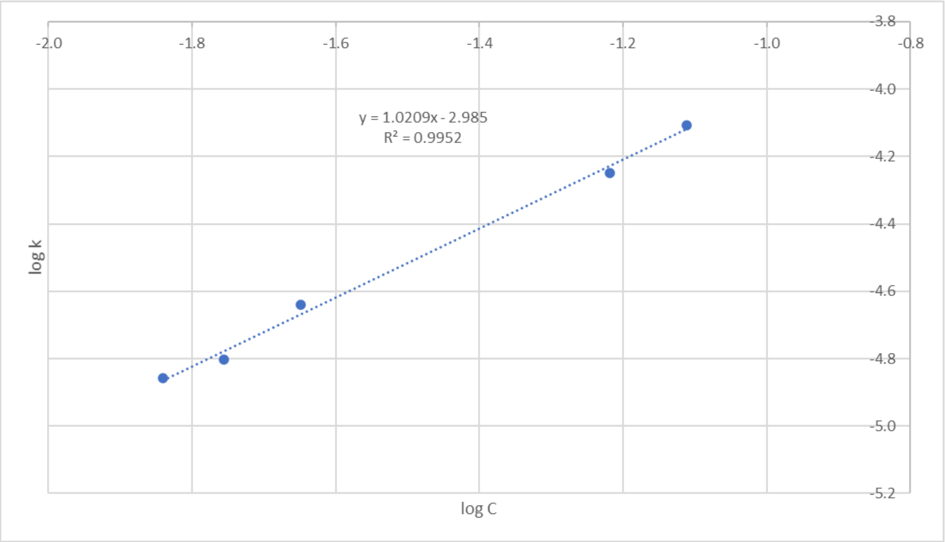

Figure S2. Plot of log(k), where k = observed rate determined from data points at 10 to 150 minutes, against log(c), where c = starting concentration of **1l**.
Gradient from line of best fit = ~1 and thus the reaction is first order with respect to [**1l**].

Determination of reaction order on PMe_3

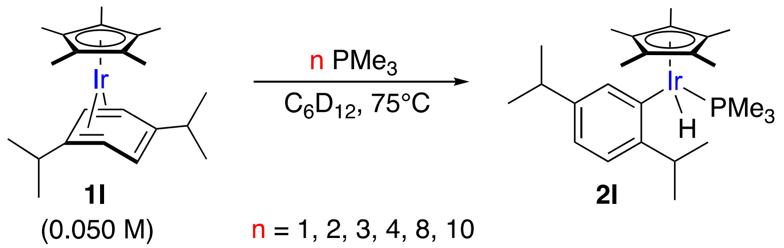

In a glovebox, J. Young NMR tube was charged with a 0.050 M solution of **1l** in C_6D_{12} (0.5 mL). HMDSO (5.3 μL) was then added as an internal standard followed by 2.5, 5.1, 10.2, 20.3 or 25.2 μL of PMe_3 (1, 2, 4, 8 or 10 eq., respectively). The J. Young NMR tube was sealed, removed from the glovebox and heated in an oil bath at 75 °C (oil bath temperature). After designated time intervals, the NMR tube was removed from the oil bath, cooled with liquid nitrogen, washed with DCM to remove external oil, an ^{1}H NMR spectrum was recorded, and the NMR tube was put back in the oil bath until the next measurement. The reaction order on PMe_3 was measured using the initial rate of the accumulation of **2l** at the conversion of **1l** less than 15%. Concentrations of **1l** and **2l** were quantified by integrating the signals indicated below against the signal of internal standard (HMDS). Experiments were carried out in triplicate.

Underlined signals were used to determine concentrations.

Starting complex **1l**:

^{1}H NMR (500 MHz, C_6D_{12}): *δ* 5.61 (1H), 4.71 (1H), 2.79 (1H), 2.74 (1H), 2.20 (1H), 1.89 (15H), 1.85 (1H), 1.20 (6H), 0.85 (6H).

Product **2l**:

^{1}H NMR (500 MHz, C_6D_{12}): *δ* 7.26 (1H), 6.85 (1H), 6.21 (1H), 3.61 (1H), 2.66 (1H), 1.84 (15H), 1.27 (9H), 1.19 (6H), 1.15 (3H), 1.03 (3H), -16.89 (1H).

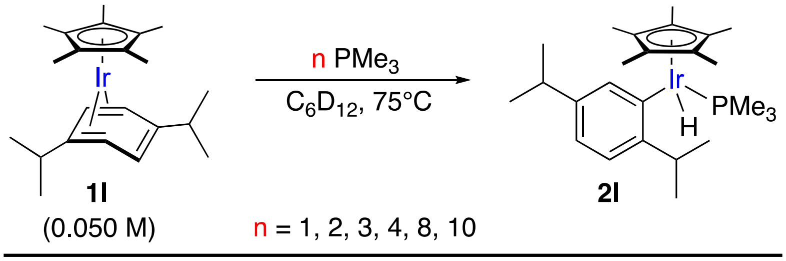

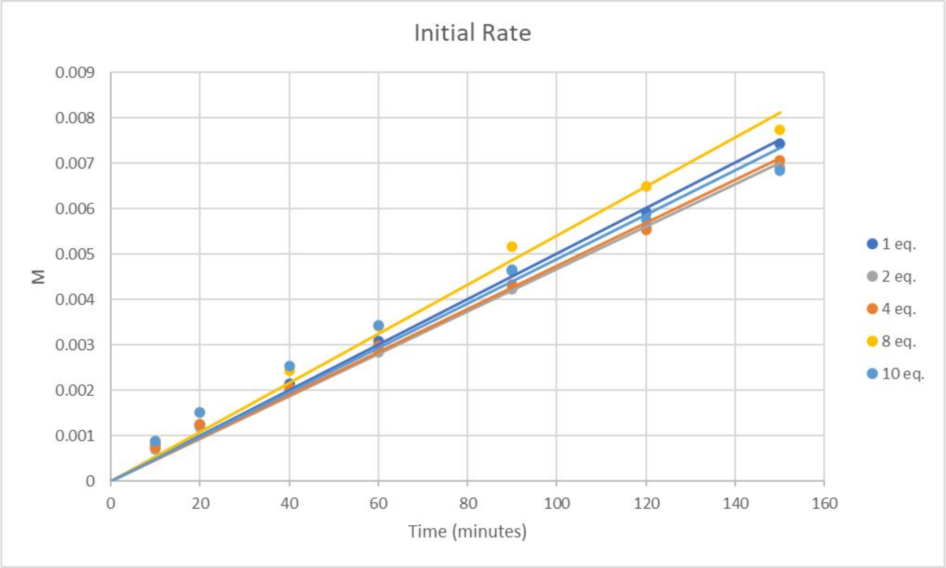

Figure S3. Plot of concentration of product **2l** (moles) against time (minutes) for varying concentrations of PMe_3.

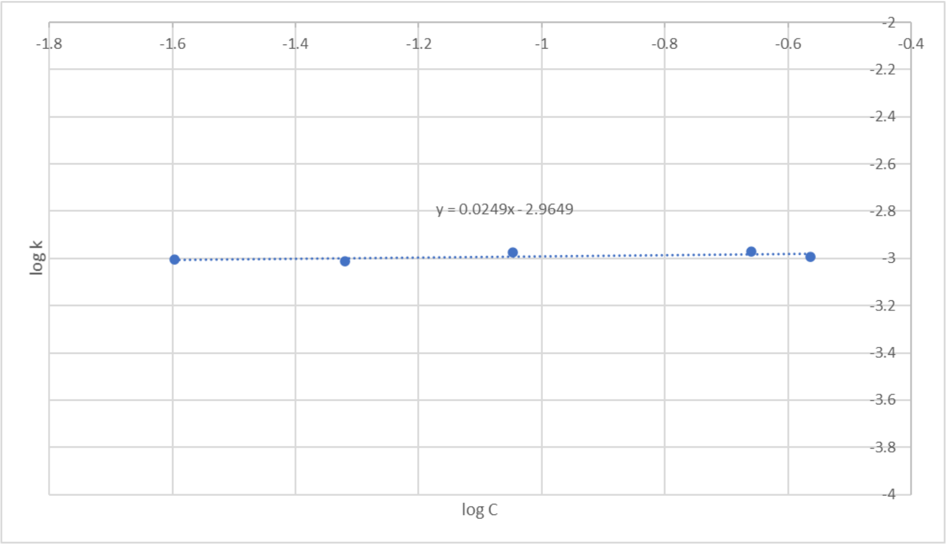

Figure S4. Plot of log(k), where k = observed rate determined from data points at 10 to 150 minutes, against log(c), where c = starting concentration of PMe_3.
Gradient from line of best fit = ~0 and thus the reaction is zero order with respect to [PMe_3].

Thermolysis of 1l-d$_4$ with PMe$_3$ to form 2l-d$_4$ (Fig 3C) in *n*-hexane

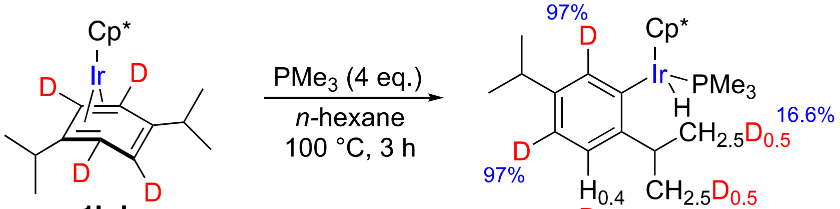

1l-d$_4$
97% D

2l-d$_4$
87%

Thermolysis of **1l-d$_4$** (25.9 mg, 0.052 mmol) was conducted according to the general procedure using PMe$_3$ (21.3 µL, 0.209 mmol) in hexane (2 mL) with a reaction time of 3 h. The product was isolated as a brown solid (87%, 26.0 mg, 0.046 mmol).

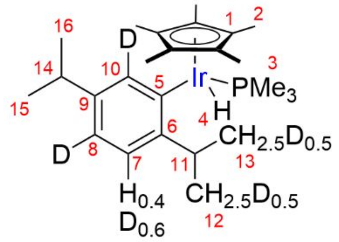

^{1}H NMR (500 MHz, C_6D_6): δ 7.27 (s, 0.41H, *H7*), 3.99-3.95 (m 1H, *H11*), 2.89 (app. sept, J = 6.9 Hz, 1H, *H14*), 1.80 (d, J = 1.6 Hz, 15H, *H2*), 1.49-1.46 (m, 2.48H, *H13*), 1.40-1.37 (m, 8.48H, *H12+H15+H16*), 1.10 (d, $J_{P\text{-}H}$ = 9.9 Hz, 9H, *H3*), -16.68 (d, $J_{P\text{-}H}$ = 40.2 Hz, 1H, *H4*).

^{13}C NMR (126 MHz, C_6D_6): δ 153.79 (d, *C6*), 142.78 (*C9*), 141.42-141.31 (m, *C10*), 137.12-137.02 (m, *C5*), 123.49 (*C7*), 120.48-120.22 (m, *C8*), 92.53 (*C1*), 38.38-38.32 (m, *C11*), 34.06 (*C14*), 25.30-24.74 (m, *C12+C13*), 25.05 (*C15*), 24.89 (*C16*), 19.20 (d, $J_{P\text{-}C}$ = 38.2 Hz, *C3*), 10.52 (*C2*).

^{31}P NMR (162 MHz, C_6D_6): δ -41.18 (s).

HRMS (ESI+): *m/z* calculated for $[^{191}IrC_{13}H_{25}P]^+$ (M – $C_{12}H_{13}D_4$) 403.1300, found 403.1291.

Determination of H/D Kinetic Isotope Effects for thermolysis of 1l and 1l-d$_4$ in C_6D_{12}

In a glovebox, a J. Young NMR tube was charged with a 0.050 M solution of **1l** or **1l-d$_4$** in C_6D_{12} (0.5 mL). HMDSO (5.3 µL) was then added as an internal standard followed by PMe$_3$ (10.2 µL). The J. Young NMR tube was subsequently sealed, removed from the glovebox and heated in an oil bath at 75 °C. After designated time interval, the NMR tube was removed from the oil bath, cooled with liquid nitrogen, washed with DCM to remove external oil, an ^{1}H NMR spectrum was recorded, and the NMR tube was put back in the oil bath until next measurement. Thermolyzes of **1l** and **1l-d$_4$** were conducted in separate J. Young NMR tubes. Concentrations of **1l** and **1l-d$_4$** were

measured using relative integrals of the H3 and H5 protons of isopropyl groups and methyl protons of HMDSO (Fig. S5). KIEs were calculated as the ratio of initial rates for the disappearance of **1l** and **1l-d$_4$** at conversions less than 15% (Fig. S6, Tables S1 and S2). Thermolysis of each complex was conducted four times.

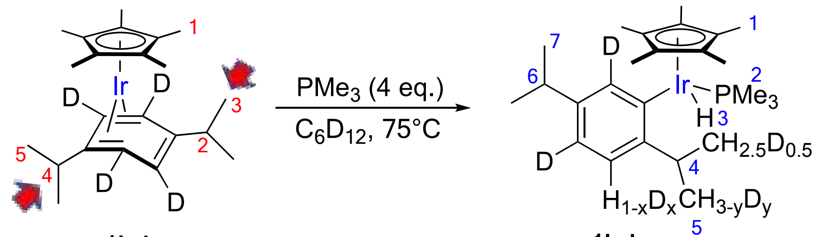

1l-d$_4$ **1l-d$_4$**

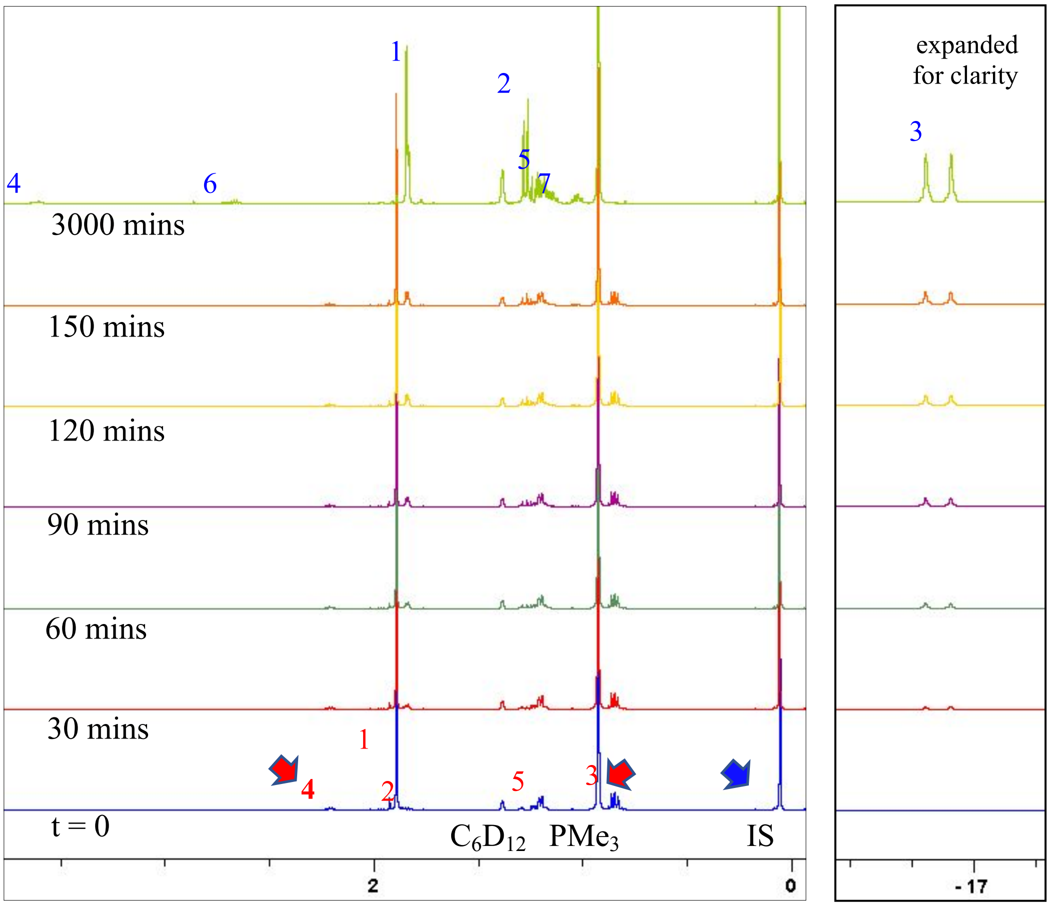

Figure S5. ^{1}H NMR (500 MHz, 25 °C, C_6D_{12}) spectra of a reaction mixture of thermolysis of **1l-d$_4$** at 75 °C. The selected hydrogen atoms corresponding to the starting complex **1l-d$_4$** and the product **2l-d$_4$** are labelled with the corresponding numbers in red and blue colours, respectively. Red and blue arrows point at the signals of the starting complex and internal standard used for integration.

Complex **1l-d$_4$**:

^{1}H NMR (500 MHz, C_6D_{12}): δ 2.21 (1H, H4), 1.88 (15H, H1), 1.85 (1H, H2), 1.20 (6H, H5), 0.85 (6H, H3).

Complex **2l-d$_4$**:

^{1}H NMR (500 MHz, C_6D_{12}): δ 3.60 (1H, H4), 2.66 (1H, H6), 1.84 (15H, H1), 1.27 (9H, H2), 1.22-1.18 (H5), 1.14, 1.03 (H7), -16.89 (1H, H3).

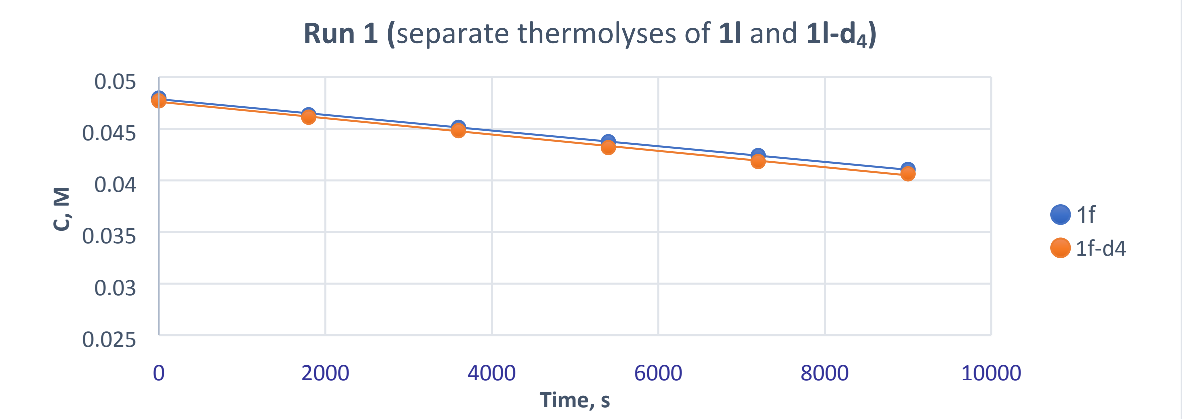

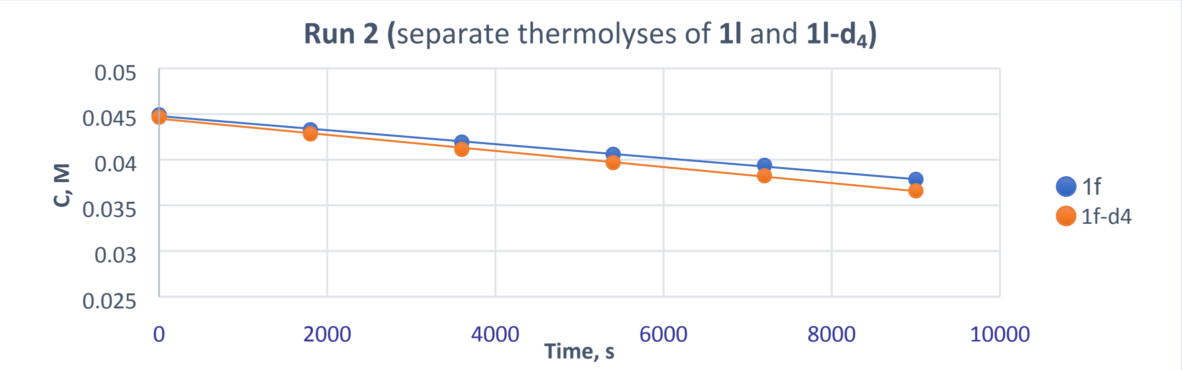

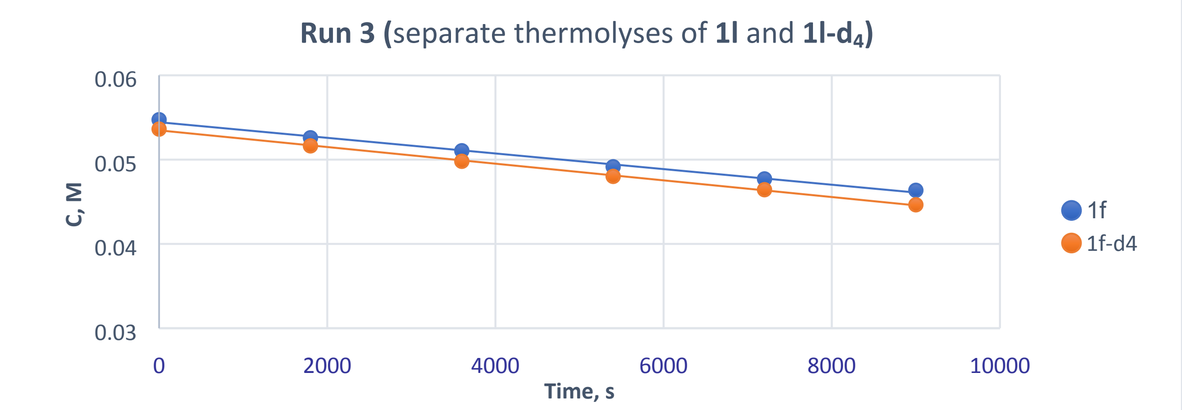

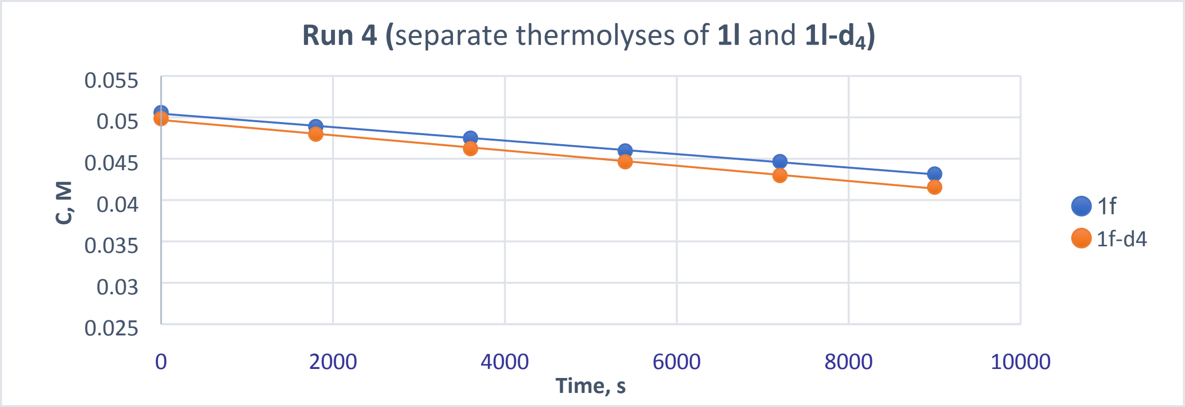

Figure S6. Concentration-time plots for the thermolysis of **1** and **1l-d$_4$** to determine the initial reaction rate. Shown are results for four separate pairs of experiments.

Table S1. Concentration-time data for the thermolysis of **1l** and **1l-d$_4$** at 75 °C.

**Time** (mins)	**Run 1**		**Run 2**		**Run 3**		**Run 4**	
	**1l** (M)	**1l-d$_4$** (M)	**1l** (M)	**1l-d$_4$** (M)	**1** (M)	**1l-d$_4$** (M)	**1l** (M)	**1l-d$_4$** (M)
0	0.04798	0.04771	0.04492	0.04467	0.05473	0.05362	0.05057	0.04985
1800	0.04636	0.04612	0.04328	0.04283	0.05259	0.05163	0.04888	0.04797
3600	0.04514	0.04482	0.04193	0.04114	0.05105	0.04980	0.04748	0.04622
5400	0.04375	0.04318	0.04059	0.03969	0.04915	0.04801	0.04594	0.04463
7200	0.04241	0.04183	0.03939	0.03825	0.04771	0.04643	0.04465	0.04297
9000	0.04106	0.04064	0.03785	0.03660	0.04636	0.04464	0.04315	0.04156

Table S2. Initial reaction rates for the thermolysis of **1l** and **1l-d$_4$**, and KIE(**1l/1l-d$_4$**).

	**Run 1**	**Run 2**	**Run 3**	**Run 4**	**Average**
k_{obs}(**1l**) (s^{-1})	$-7.593\cdot10^{-7}$	$-7.676\cdot10^{-7}$	$-9.268\cdot10^{-7}$	$-8.147\cdot10^{-7}$	$-8.171\cdot10^{-7}$
k_{obs}(**1l-d$_4$**) (s^{-1})	$-7.914\cdot10^{-7}$	$-8.815\cdot10^{-7}$	$-9.887\cdot10^{-7}$	$-9.212\cdot10^{-7}$	$-8.957\cdot10^{-7}$
KIE(**1l/1l-d$_4$**)	0.95(9)	0.87(0)	0.93(7)	0.88(4)	0.91

KIE(**1l/1l-d$_4$**) = 0.91 ± 0.09 (confidence interval 95%, standard deviation 0.042, n 4, degrees of freedom 4, Student's t 3.18,

Thermolysis of 1l-d$_2$ in the presence of PMe$_3$ to give 2l-d$_2$

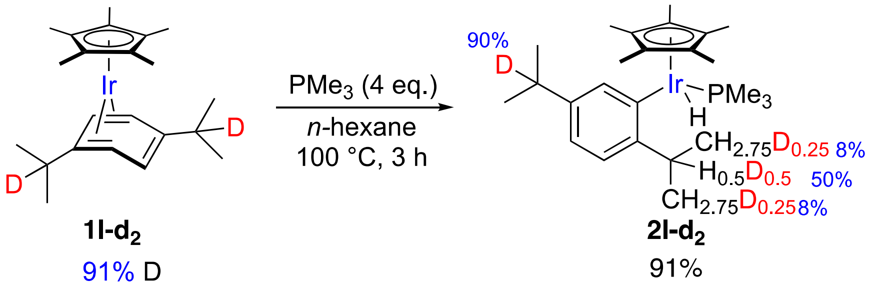

1l-d$_2$
91% D

2l-d$_2$
91%

Thermolysis of **1l-d$_2$** (33.1 mg, 0.067 mmol) was conducted according to the general procedure using PMe$_3$ (27.3 μL, 0.268 mmol) in hexane (2 mL) with a reaction time of 3 h. The product was isolated as a brown solid (91%, 34.7 mg, 0.061 mmol).

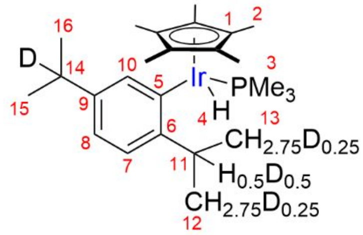

^{1}H NMR (500 MHz, C_6D_6): δ 7.48 (d, J = 1.8 Hz, 1H, *H10*), 7.28 (d, J = 7.8 Hz, 1H, *H7*), 7.00 (dd, J = 7.8, 2.0 Hz, 1H, *H8*), 4.02-3.95 (m, 0.5H, *H11*), 1.80 (d, J = 1.6 Hz, 15H, *H2*), 1.49 (d, J = 7.4 Hz, 2.75H, *H13*), 1.41-1.38 (m, 8.75H, *H12+H15+H16*), 1.09 (d, $J_{P\text{-}H}$ = 9.9 Hz, 9H, *H3*), -16.69 (dd, J = 40.1, 5.7 Hz, 1H, *H4*).

^{13}C NMR (126 MHz, C_6D_6): δ 153.32 (d, *C6*), 142.42 (*C9*), 141.20 (d, $J_{P\text{-}C}$ = 7.6 Hz, *C10*), 136.74 (t, *C5*), 123.32 (*C7*), 120.46 (*C8*), 92.10 (d, $J_{P\text{-}C}$ = 3.4 Hz, *C1*), 37.95 (*C11*-H), 37.61 (t, *C11*-D), 33.42 (t, *C14*), 25.36-24.88 (m, *C12+C13+C15+C16*), 19.48 (d, $J_{P\text{-}C}$ = 38.2 Hz, *C3*), 10.77 (*C2*).

^{31}P NMR (162 MHz, C_6D_6): δ -40.82 (s).

HRMS (ESI+): *m/z* calculated for $[^{191}IrC_{13}H_{25}P]^+$ (M – $C_{12}H_{15}D_2$) 403.1300, found 403.1292.

8. Crystallography data

Single crystals of **2a** and **2a-br** were mounted on a Mitegen loop using Paratone-N oil on a SuperNova, Dual, AtlasS2 diffractometer. During data collection, the crystals were kept at 100(1) and 140(1) K, respectively. The structure was solved using Olex2[8] with the SHELXT[9] structure solution program using Intrinsic Phasing and refined with the SHELXL[10] refinement package using Least Squares minimisation. **2a-ph** was refined as a two component twin with 56% / 44% occupancy ratio, and twin law involving a rotation by 4.5141% around the [0.61 0.02 -0.79] (reciprocal) or [0.95 0.02 -0.30] (direct) axis.

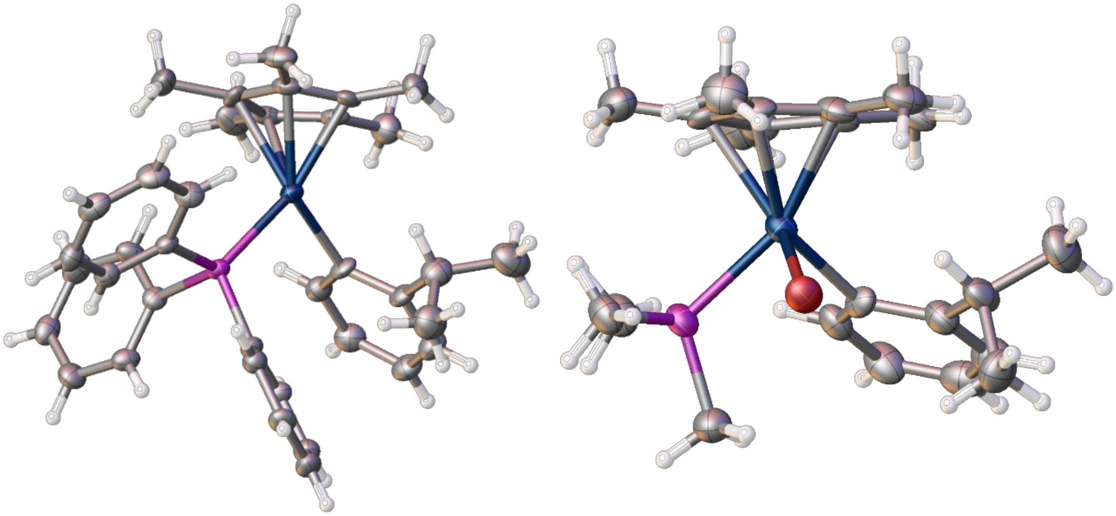

Figure S7. Refined structures of **2a-ph** (left) and **2a-br** (right).

Table S3. Crystallographic data and structure refinement for **2a-ph** and **2a-br**.

Compound	**2a-ph**	**2a-br**
CCDC	2128609	2128610
Formula	$C_{37}H_{41}IrP$	$C_{22}H_{35}BrIrP$
M	708.87	602.58
Crystal system	Monoclinic	Monoclinic
Space group	*Pn*	$P2_1/n$
a/Å	8.4870(2)	8.7529(3)
b/Å	10.9813(2)	29.6008(11)
c/Å	16.3630(3)	8.7647(4)
α/°	90	90
β/°	95.909(2)	100.087(4)
γ/°	90	90
U/Å^3	1516.90(5)	2235.77(15)
Z, *Z'*	2, 1	4, 1
F(000)/e	710	1176
D_{calc}/Mg m^{-3}	1.552	1.790
μ/mm^{-1}	9.181	14.327
θ_{max}/°	73.79	76.41
Data measured	10403	21382
Unique data	10403	4565
R_{int}	-	0.086
R_1, wR_2 (obs. data)	0.041, 0.109	0.078, 0.180
S	1.033	1.189
Variables	360	236
E_{max}, E_{min}/e Å^{-3}	1.45, -0.98	4.40, -1.83
Flack parameter	-0.014(10)	-

Crystallographic files can be obtained free-of-charge from: https://www.ccdc.cam.ac.uk

9. DFT calculations data

All DFT calculations were performed with Gaussian 16; geometry optimizations, analytical frequency calculations and intrinsic reaction path calculations were at the M06-2x/def2SVP level in the gas phase. Stabilities of converged wavefunctions were confirmed by running the "stable" test. Analytical frequency calculations confirmed that all converged minima contained 0 and all converged transition-state geometries contained exactly 1 imaginary frequency. IRC calculations established all minima connected to each transition-state geometry. Single point energies of all converged geometries were calculated with the def2TZVPP basis set in hexane-parameterized CPCM. Basis-set superposition error for energies of association reactions was calculated by the counterpoise method.

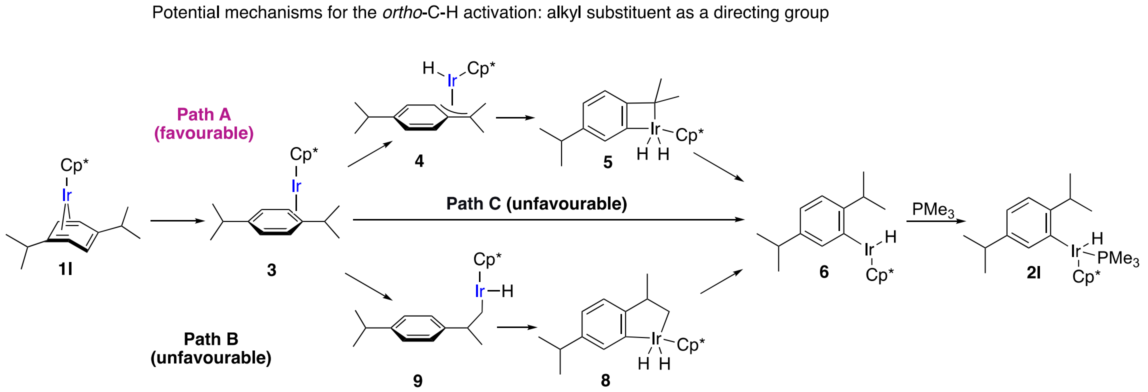

Figure S8. Overview of calculated mechanisms for *ortho*-C-H oxidative addition upon thermolysis of **1l** in the presence of PMe_3: Paths A-C. Path A is the same as Path 1 in Fig. 4A in the main text.

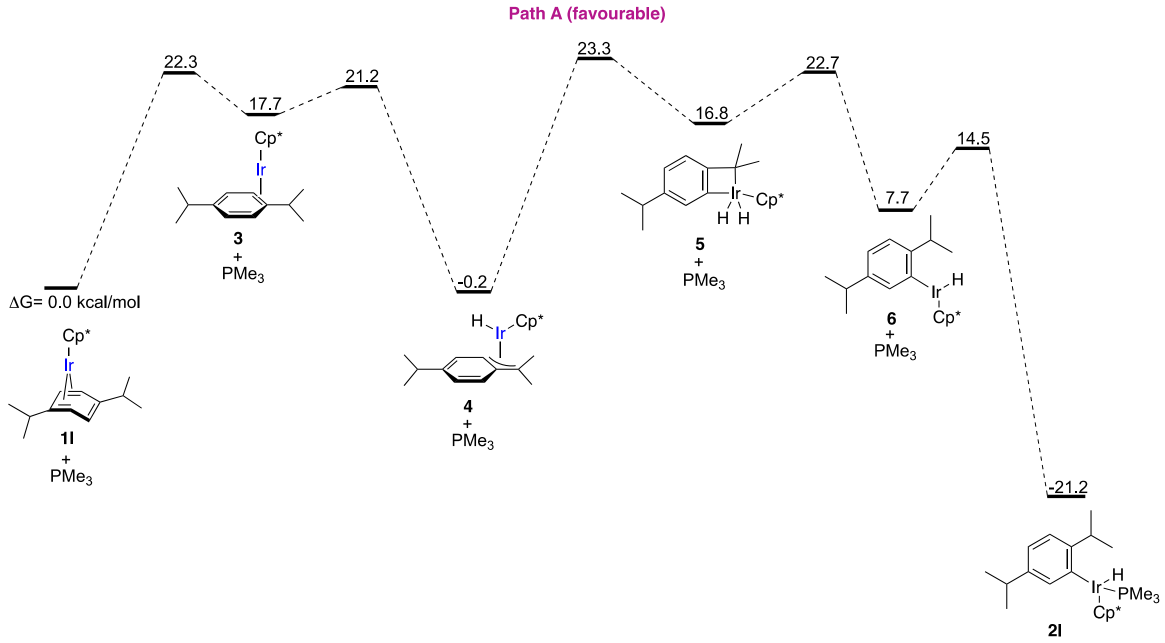

Figure S9. Energy diagram of the lowest energy mechanism of aromatic C-H activation involving an initial benzylic C-H activation (Path A). Same is Path 1 in Fig. 4A in the main text.

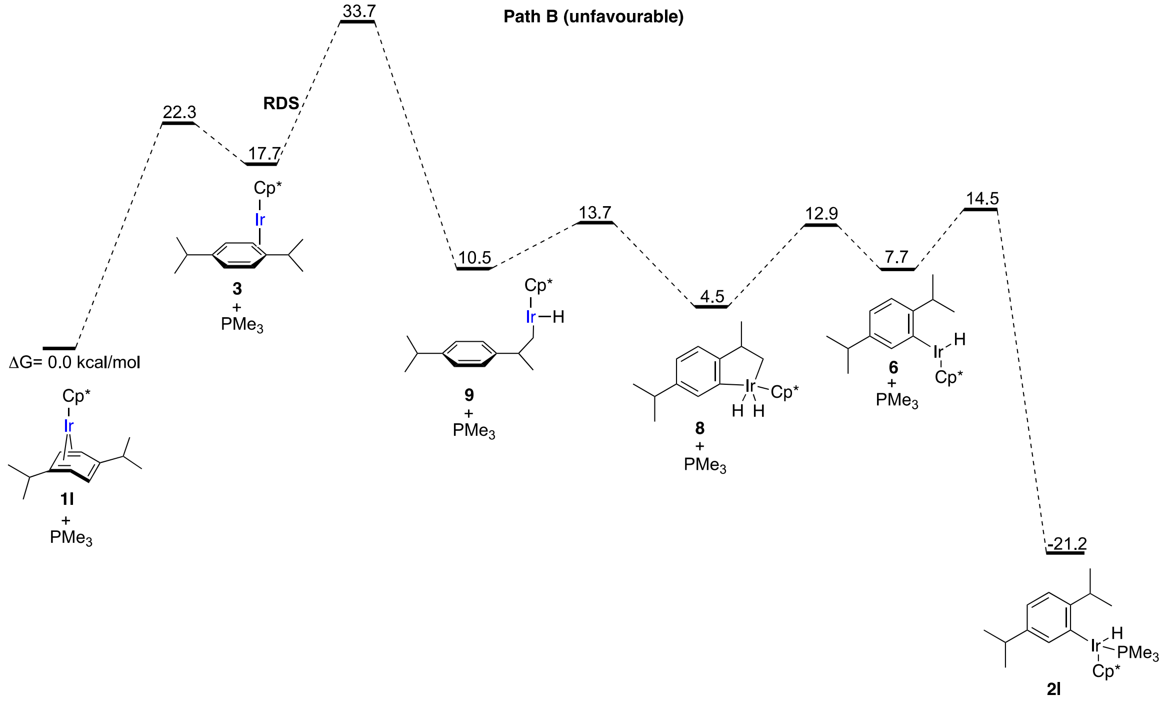

Figure S10. Energy diagram of a higher energy mechanism of aromatic C-H activation involving an initial β-C-H activation (Path B).

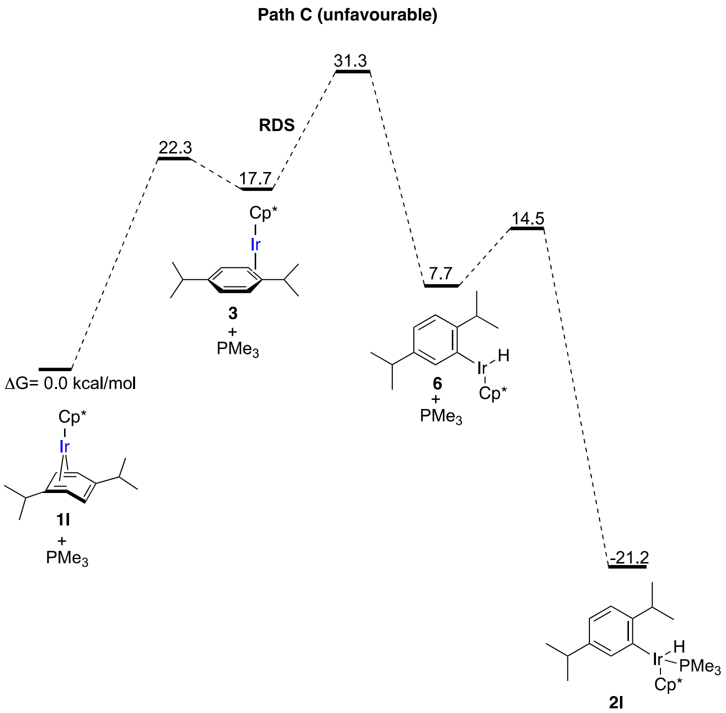

Figure S11. Energy diagram of a higher energy mechanism involving direct aromatic C-H activation (Path C).

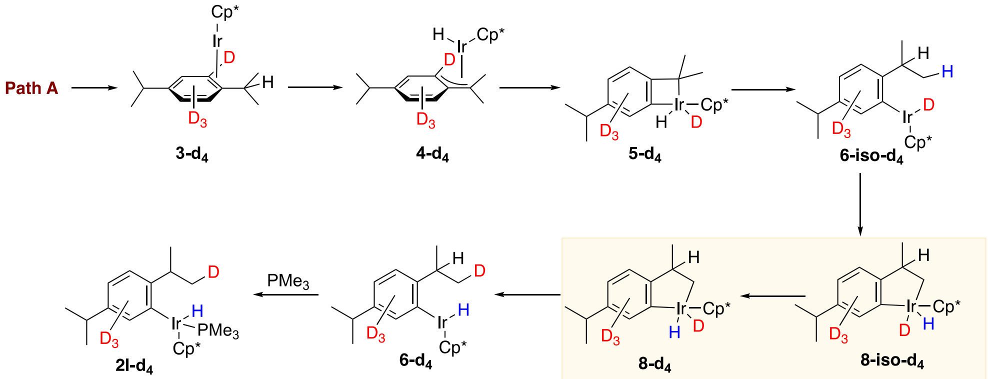

Figure S12. The lowest energy pathway for the H/D scrambling upon thermolysis of **1l-d$_4$** **(**Path D**).**

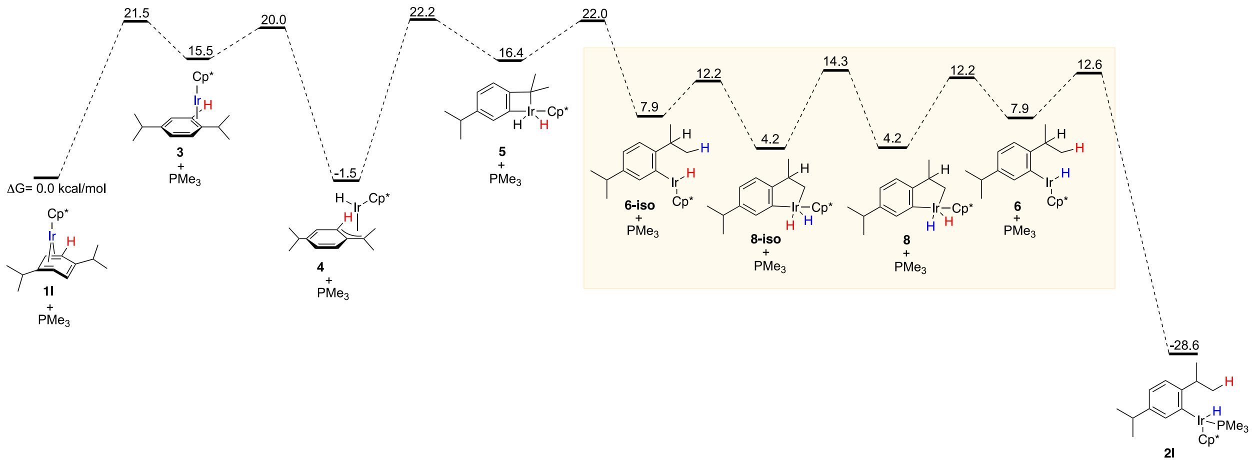

Figure S13. Energy diagram of the lowest energy pathway (Path D).

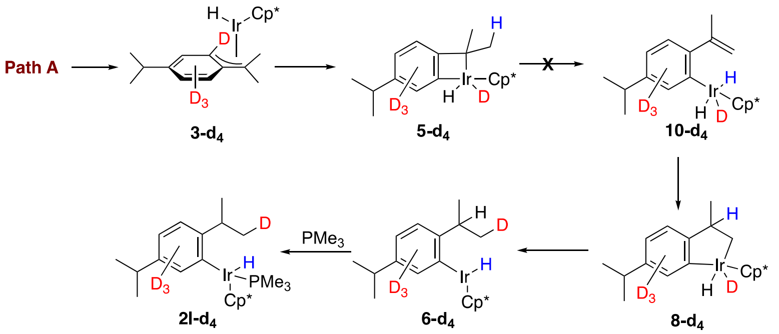

Figure S14. A higher energy pathway for the H/D scrambling upon thermolysis of **1l-d$_4$ (**Path E**).**

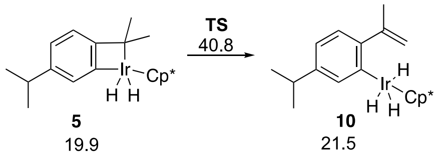

Figure S15. Part of Path E with prohibitively high free energy barrier for the conversion of conversion of intermediate **5** into **10**.

Table S4. Cartesian coordinates for all calculated structures.

1l

6 6.726385 6.656945 1.088732
6 6.341519 7.366993 -0.105408
6 4.957019 7.738864 0.009079
6 4.495295 7.321100 1.288636
6 5.585901 6.673327 1.966450
6 8.035884 5.972372 1.337773
1 8.858720 6.505546 0.843139
1 8.027731 4.935130 0.965357
1 8.266922 5.946452 2.411106
6 7.206376 7.565371 -1.314213
1 6.975653 8.513262 -1.819851
1 7.063612 6.750014 -2.040740
1 8.268064 7.589121 -1.035782
6 4.119219 8.408069 -1.039036
1 3.595244 9.290042 -0.641644
1 3.357231 7.715288 -1.429880
1 4.731089 8.735994 -1.889403
6 3.102730 7.494513 1.812751
1 3.094725 7.578962 2.907687
1 2.464871 6.638203 1.539659
1 2.637194 8.401493 1.403090
6 5.510450 6.021192 3.314049
1 6.494765 6.005843 3.800278
1 5.156551 4.981683 3.228830
1 4.818410 6.558395 3.976902
6 7.826156 10.210403 1.310249
6 6.547487 10.853184 1.616346
6 6.047500 10.431226 2.888682
6 6.913372 9.464420 3.551221
1 6.494623 8.959466 4.428752
6 8.380092 9.757605 3.581652
6 8.851717 10.147463 2.389384
1 9.877729 10.472250 2.207176
77 6.308598 8.764403 1.644210
1 5.068985 10.744299 3.258979
1 8.189583 10.328182 0.285618
6 5.808118 11.833333 0.734487
1 4.730122 11.677494 0.914330
6 6.165753 13.264936 1.147678
1 7.243976 13.439893 1.010484
1 5.618264 13.999414 0.538410
1 5.927657 13.445228 2.205527
6 6.079368 11.610068 -0.750550
1 5.900065 10.560501 -1.025348
1 5.425984 12.248098 -1.362876
1 7.120607 11.860628 -1.003936
6 9.122827 9.697926 4.891305
1 8.918498 8.702299 5.328689
6 10.631269 9.843822 4.722587
1 11.145098 9.720826 5.686954
1 11.027909 9.097526 4.019706
1 10.881316 10.843337 4.334489
6 8.579527 10.748592 5.868230
1 8.767412 11.759087 5.473789
1 7.495289 10.641734 6.015007
1 9.068693 10.663286 6.850152

1t3

77 -0.665309 2.719901 4.821898
6 -2.416451 2.862222 5.989140
6 -1.248604 2.592735 6.852792
6 0.692464 3.781815 3.566215
6 -0.329211 3.270082 2.641738
6 -0.321395 1.876662 2.764396
6 0.731880 1.517432 3.735995
6 1.463775 2.691361 4.087535
6 2.684283 2.787615 4.947323
6 1.080684 0.109313 4.101403
6 -1.173200 0.879087 2.046161
6 -1.236510 4.125340 1.815463
6 0.983530 5.234030 3.778392
1 -1.081245 1.564988 7.189594
6 -0.738895 3.618049 7.756460
6 -1.139764 4.913823 7.669290
6 -2.131137 5.240486 6.665082
6 -2.741588 4.287564 5.906783
1 -3.540406 4.587548 5.223480
6 -3.563235 1.876393 5.745893
1 -2.028474 1.361416 1.555917
1 -1.565376 0.120657 2.738117
1 -0.588612 0.354855 1.272962
1 -2.081546 3.543630 1.424854
1 -0.701439 4.568920 0.960662
1 -1.650553 4.948222 2.415657
1 0.061509 5.827554 3.721916
1 1.681522 5.607462 3.012084
1 1.431881 5.401445 4.766888
1 2.786638 1.899954 5.585552
1 2.632674 3.663157 5.609411
1 3.600354 2.874660 4.341058
1 0.174347 -0.502993 4.203087
1 1.622661 0.072592 5.055147
1 1.714342 -0.349550 3.325592
1 -4.368897 2.143275 6.454829
6 -4.126635 1.994932 4.329165
1 -3.363482 1.684206 3.599491
1 -5.012081 1.353309 4.207053
1 -4.423403 3.024134 4.082979
6 -3.167430 0.428811 6.026392
1 -2.864720 0.279014 7.071929
1 -4.010797 -0.246440 5.821044
1 -2.324026 0.130202 5.380587
1 -2.432595 6.285556 6.549085
1 -0.015251 3.306711 8.512268
6 -0.652757 6.024533 8.579415
6 -1.776122 6.489370 9.514839
1 -1.454874 7.348027 10.123652
1 -2.673829 6.783573 8.952821
1 -2.060397 5.670235 10.193075
1 -0.392031 6.876693 7.925150
6 0.590354 5.657029 9.383261
1 0.368593 4.854330 10.103409
1 1.405211 5.311878 8.730577
1 0.948425 6.524590 9.955980

3

77 -0.879293 2.208634 4.956532
6 -2.611288 2.907269 5.922234
6 -1.558153 2.596301 6.904794
6 0.509204 3.581161 4.235183
6 -0.282382 3.218932 3.052237
6 -0.079867 1.846212 2.837264
6 0.920201 1.364088 3.800222
6 1.352556 2.439250 4.591846
6 2.407406 2.457930 5.652149
6 1.406319 -0.051523 3.849565
6 -0.711117 0.986412 1.785799
6 -1.142145 4.173239 2.286303
6 0.714323 4.981186 4.717008
1 -1.621863 1.661241 7.472753
6 -0.907031 3.689170 7.615888
6 -1.198248 4.994187 7.368522
6 -2.238108 5.296415 6.404091
6 -2.916816 4.322967 5.744945
1 -3.720303 4.607015 5.061648
6 -3.719609 1.877284 5.672934
1 -1.608268 1.462086 1.368527
1 -1.008950 0.010591 2.195039
1 -0.010663 0.795433 0.956841
1 -1.865064 3.641166 1.653822
1 -0.531939 4.820577 1.635879
1 -1.705627 4.821065 2.974067
1 -0.225491 5.548982 4.686379
1 1.454778 5.492649 4.080624
1 1.076050 4.993913 5.753484
1 2.663308 1.441679 5.979056
1 2.060898 3.016434 6.533891
1 3.328858 2.940471 5.288222
1 0.568584 -0.760363 3.787225
1 1.948277 -0.254470 4.781922
1 2.082735 -0.264926 3.006575
1 -4.452135 1.940067 6.499262
6 -4.463317 2.128205 4.360094
1 -3.753992 2.124769 3.516565
1 -5.213656 1.342878 4.186452
1 -4.992453 3.090309 4.358807
6 -3.137462 0.457162 5.669941
1 -2.760526 0.144788 6.651625
1 -3.890260 -0.277125 5.348600
1 -2.301296 0.369459 4.935916
1 -2.487316 6.345372 6.218556
1 -0.172070 3.422464 8.379115
6 -0.528897 6.160144 8.072070
6 -1.521876 6.881456 8.992259
1 -1.066806 7.778092 9.439233
1 -2.426488 7.190507 8.450058
1 -1.833583 6.208632 9.805721
1 -0.229801 6.873333 7.280981
6 0.726503 5.774761 8.848120
1 0.479252 5.113628 9.692829
1 1.454602 5.250519 8.211044
1 1.213152 6.670320 9.260223

3t4

77 -0.792566 2.081032 4.239155
6 -2.160373 2.912172 5.562525
6 -1.132076 2.263247 6.369751
6 0.445852 3.572564 2.905351

6 -0.211482 2.670385 2.042703
6 0.217534 1.324353 2.395834
6 1.196046 1.407132 3.431878
6 1.255384 2.793959 3.821845
6 2.189431 3.383853 4.832823
6 2.032540 0.293072 3.981542
6 -0.172854 0.084229 1.648556
6 -1.164972 2.996168 0.933923
6 0.307864 5.060711 2.966650
1 -1.304040 1.275743 6.803145
6 -0.252209 3.134311 7.127633
6 -0.356334 4.494293 7.101157
6 -1.419665 5.096739 6.326347
6 -2.296452 4.348309 5.603659
1 -3.102595 4.828020 5.044649
6 -2.992333 1.977361 4.769996
1 -1.216443 0.136950 1.309787
1 -0.070274 -0.807398 2.280624
1 0.461624 -0.052959 0.758311
1 -2.017627 2.301961 0.922930
1 -0.673267 2.928677 -0.049971
1 -1.566069 4.013235 1.038029
1 -0.467151 5.419239 2.276468
1 1.253363 5.560453 2.703419
1 0.027134 5.386668 3.981928
1 2.394047 2.674029 5.646102
1 1.758689 4.289085 5.283788
1 3.148734 3.655482 4.363421
1 1.519270 -0.672626 3.884352
1 2.244013 0.447924 5.048041
1 2.997348 0.219127 3.453058
1 -2.134196 1.062187 4.280776
6 -3.760009 0.907927 5.555425
1 -4.596545 1.389922 6.084672
1 -4.173009 0.144357 4.880057
1 -3.127331 0.406540 6.297321
6 -3.866264 2.596161 3.692203
1 -3.345674 3.402299 3.159966
1 -4.162011 1.833955 2.956375
1 -4.788985 3.000199 4.140263
1 -1.522700 6.185158 6.335075
1 0.502163 2.658051 7.757701
6 0.546333 5.422746 7.896218
6 -0.247648 6.137499 8.997365
1 0.383376 6.867672 9.525704
1 -1.119993 6.669769 8.593348
1 -0.614179 5.402526 9.730256
1 0.905159 6.194786 7.190172
6 1.769261 4.734568 8.494463
1 1.471741 4.003118 9.261571
1 2.354220 4.205742 7.728563
1 2.423849 5.473229 8.978540

4
77 11.964544 15.168001 20.420698
1 10.646360 14.537101 20.955636
6 13.009584 14.072919 16.867079
6 12.081851 13.500493 17.692547
6 11.191693 14.275312 18.519680
1 10.266740 13.789280 18.823711
6 14.035415 14.229801 20.923194
6 12.868520 15.623848 22.364243
6 14.320015 15.595176 20.554539
6 13.622595 16.447571 21.449919
6 11.284000 15.717048 18.467572
6 13.172253 14.236883 22.053160
6 12.239232 16.293787 17.547239
1 12.278078 17.379510 17.444846
6 13.050042 15.509610 16.784040
6 10.558258 16.461975 19.494104
6 15.230104 15.994223 19.436597
1 15.081362 15.345487 18.560407
1 16.287477 15.915766 19.736928
1 15.044314 17.027384 19.113011
6 14.608720 13.033570 20.230290
1 14.134746 12.106248 20.576748
1 15.691771 12.953310 20.415071
1 14.455603 13.104308 19.142329
6 12.129218 16.117015 23.571381
1 11.633936 17.075160 23.363363
1 12.811868 16.264267 24.424119
1 11.352580 15.403845 23.876150
6 13.706444 17.940480 21.517940
1 14.030072 18.371105 20.560719
1 14.434144 18.249481 22.285362
1 12.736994 18.385372 21.779289
6 12.728258 13.046156 22.848810
1 11.753457 13.228507 23.318585
1 13.452982 12.815803 23.645734
1 12.626715 12.157785 22.212053
6 9.087205 16.165619 19.725878
1 8.804643 16.425390 20.756485
1 8.465159 16.769829 19.042106
1 8.838158 15.109301 19.567789
6 10.835506 17.947260 19.593591
1 10.273324 18.510380 18.826956
1 10.509309 18.321385 20.576449
1 11.901327 18.184630 19.482774
1 11.982770 12.411419 17.712063
1 13.738839 15.983435 16.079659
6 13.909983 13.240503 15.974290
1 13.741116 12.184824 16.243440
6 15.393928 13.550563 16.193061
1 15.619096 14.601412 15.952708
1 15.694524 13.370949 17.235303
1 16.021252 12.921055 15.544666
6 13.531453 13.417580 14.499802
1 14.152801 12.776877 13.856405
1 13.681592 14.460475 14.180037
1 12.475974 13.162846 14.330949

3t9
77 -0.820750 2.389472 4.595231
6 -2.422244 3.141591 5.847458
6 -1.290001 2.789843 6.675758
6 0.812404 3.843802 3.995865
6 0.130459 3.410702 2.814647
6 0.308762 1.989058 2.731277
6 1.232073 1.573333 3.777439
6 1.536184 2.709396 4.549530
6 2.455040 2.784228 5.727726
6 1.761799 0.182648 3.946683
6 -0.173033 1.093921 1.628027
6 -0.596213 4.304389 1.857562
6 0.952906 5.272102 4.423831
1 -1.297359 1.833147 7.207215
6 -0.543997 3.860419 7.311776
6 -0.853626 5.175450 7.120053
6 -1.985020 5.505409 6.284869
6 -2.751609 4.541068 5.705755
1 -3.626060 4.820555 5.114038
6 -3.411661 1.997221 5.625138
1 -1.061661 1.511537 1.136446
1 -0.437674 0.098836 2.012063
1 0.607549 0.959886 0.862499
1 -1.332996 3.743626 1.267326
1 0.102685 4.787631 1.154932
1 -1.133844 5.096785 2.397002
1 -0.025181 5.774757 4.461855
1 1.596528 5.825205 3.720926
1 1.398728 5.346261 5.424249
1 2.638711 1.791244 6.158542
1 2.029608 3.421774 6.515286
1 3.427503 3.215722 5.440903
1 0.978325 -0.565776 3.764298
1 2.143917 0.022248 4.963300
1 2.584425 -0.019501 3.241521
1 -3.851503 1.685806 6.588605
6 -4.542103 2.339322 4.663647
1 -4.139422 2.685146 3.697845
1 -5.171565 1.456693 4.479720
1 -5.188692 3.131578 5.066701
6 -2.477678 0.919127 5.052677
1 -1.958097 0.302560 5.795895
1 -2.988667 0.232382 4.359227
1 -2.075887 1.650740 3.921736
1 -2.235668 6.559161 6.134865
1 0.257722 3.581069 7.998442
6 -0.111585 6.321316 7.784448
6 -1.013994 7.028012 8.804380
1 -0.510601 7.908782 9.230065
1 -1.958687 7.359258 8.351448
1 -1.260566 6.339170 9.626694
1 0.121166 7.048252 6.984265
6 1.203470 5.913505 8.440922
1 1.027030 5.234331 9.289070
1 1.874970 5.404754 7.733292
1 1.726871 6.798545 8.829602

9
77 11.942180 15.254792 20.545092
1 10.590050 14.594720 20.945444
6 13.066576 14.207776 16.981115
6 12.073449 13.641069 17.745085
6 11.131207 14.433285 18.471114
1 10.211379 13.955465 18.804653
6 13.995475 14.098365 20.924723
6 12.884116 15.437410 22.454789
6 14.341294 15.483216 20.654991
6 13.700473 16.297345 21.609986
6 11.202861 15.848438 18.401632
6 13.146591 14.061510 22.052410
6 12.241180 16.419662 17.599821
1 12.298903 17.505805 17.507156
6 13.132554 15.632652 16.916181
6 15.281355 15.914151 19.575933

1 15.045000 15.417093 18.623725
1 16.321103 15.657051 19.835880
1 15.232282 16.998021 19.406557
6 14.539969 12.936076 20.156536
1 14.049855 11.997979 20.447803
1 15.622626 12.821545 20.327825
1 14.385802 13.075255 19.075757
6 12.179169 15.867677 23.705989
1 11.712941 16.853596 23.573542
1 12.875918 15.930957 24.557336
1 11.381698 15.160140 23.968364
6 13.819162 17.781775 21.758737
1 14.060428 18.262582 20.800755
1 14.610177 18.043860 22.479090
1 12.879365 18.218332 22.123810
6 12.636022 12.842542 22.759126
1 11.645785 13.026158 23.194898
1 13.317457 12.551429 23.574315
1 12.544114 11.991771 22.071616
6 9.743694 17.977102 18.277595
1 8.905426 18.484106 18.777056
1 10.587448 18.683385 18.264189
1 9.451922 17.770272 17.236395
1 11.968137 12.552987 17.769097
1 13.894220 16.108244 16.293327
6 13.997918 13.356325 16.136532
1 13.840726 12.308845 16.443165
6 15.477097 13.690925 16.348260
1 15.695389 14.731781 16.063682
1 15.779977 13.556419 17.396249
1 16.109240 13.040325 15.726268
6 13.626228 13.473830 14.653765
1 14.262220 12.821145 14.037317
1 12.576036 13.196620 14.487603
1 13.761123 14.508354 14.301578
6 10.099613 16.702042 19.030885
1 9.203886 16.062211 19.085256
6 10.662934 16.888526 20.436106
1 11.267168 17.812764 20.480046
1 9.896900 16.935902 21.223568

4t5
77 -1.236445 2.876014 4.548855
6 -3.022795 1.530270 4.374690
6 -1.848108 0.685599 4.461467
6 -1.067074 0.903817 3.295754
6 -1.777297 1.835898 2.447544
6 -2.991844 2.181901 3.084662
6 -4.115296 2.974478 2.493138
6 -1.298403 2.301037 1.106235
6 0.216320 0.219957 2.930616
6 -1.556005 -0.294758 5.557584
6 -4.197206 1.504579 5.307751
1 0.761283 -0.113580 3.823354
1 0.876458 0.891858 2.364813
1 0.027321 -0.664284 2.300842
1 -0.475512 -0.444740 5.680694
1 -2.011728 -1.272449 5.334666
1 -1.955633 0.053458 6.518523
1 -3.883458 1.255445 6.329802
1 -4.944163 0.759115 4.990164
1 -4.694599 2.483354 5.345743
1 -3.754724 3.734312 1.786869
1 -4.703292 3.483183 3.267072
1 -4.797579 2.305158 1.944617
1 -0.241867 2.605451 1.145325
1 -1.876562 3.165197 0.754881
1 -1.389343 1.501670 0.353914
6 0.902876 6.777987 2.929855
6 -0.217326 6.681620 3.764359
1 -0.810578 7.569421 3.995908
6 -0.559178 5.432060 4.269250
6 0.179721 4.306587 3.908327
6 1.286244 4.393207 3.067331
6 1.660556 5.653289 2.573869
6 -1.713770 4.895264 5.080241
1 -1.021246 2.828467 6.084608
1 0.305689 3.268057 4.854211
6 -1.578845 5.231529 6.564466
1 -1.711221 6.317232 6.724247
1 -2.344946 4.704490 7.153757
1 -0.591373 4.951115 6.956538
6 -3.058552 5.379944 4.558755
1 -3.888275 4.844821 5.048420
1 -3.192440 6.456030 4.773825
1 -3.144571 5.243772 3.472103
6 2.866018 5.791876 1.659942
1 3.291990 4.782774 1.535889
6 2.462269 6.302128 0.273620
1 2.039734 7.316527 0.338618
1 3.333857 6.343691 -0.396572
1 1.703381 5.650807 -0.182670
6 3.946051 6.681917 2.281099
1 4.251313 6.304959 3.267237
1 4.835147 6.725706 1.634488
1 3.576279 7.710252 2.413496
1 1.863591 3.506061 2.791216
1 1.189828 7.757172 2.537899

5
77 -0.830360 2.591952 4.260388
6 -2.672500 3.671651 5.859139
6 -1.360112 3.297081 6.092043
6 0.264482 4.490624 3.441875
6 -0.118777 3.710231 2.305632
6 0.576109 2.455685 2.379146
6 1.419522 2.473436 3.536570
6 1.216472 3.719157 4.201205
6 1.960615 4.226260 5.397475
6 2.390366 1.402415 3.933628
6 0.542899 1.380277 1.334488
6 -0.956083 4.153594 1.144255
6 -0.132552 5.900075 3.769860
1 -0.816815 1.378398 5.229220
6 -0.748594 3.548555 7.316385
6 -1.481871 4.223956 8.307620
6 -2.801791 4.620724 8.042698
6 -3.420842 4.346974 6.817989
1 -4.453583 4.655363 6.637511
6 -2.909174 3.132899 4.472338
1 -0.429034 1.348471 0.825488
1 0.725033 0.390533 1.773028
1 1.317288 1.557830 0.571109
1 -1.620899 3.351164 0.795815
1 -0.311204 4.443640 0.299526
1 -1.581430 5.016916 1.400893
1 -0.829584 6.302141 3.023395
1 0.749131 6.558880 3.788058
1 -0.619847 5.961415 4.756224
1 2.278283 3.404771 6.053871
1 1.338534 4.911122 5.989062
1 2.863928 4.772873 5.082165
1 2.017245 0.405811 3.663390
1 2.568798 1.409081 5.016732
1 3.360028 1.545997 3.431182
1 -1.631101 1.336780 3.810756
6 -3.891596 1.965199 4.494987
1 -4.894634 2.328200 4.782778
1 -3.973271 1.492778 3.503916
1 -3.587856 1.201814 5.224218
6 -3.361169 4.181230 3.467624
1 -3.405979 3.767160 2.449013
1 -4.376752 4.530155 3.728334
1 -2.698901 5.057825 3.467393
6 -0.846465 4.524778 9.654890
1 0.171411 4.102097 9.630275
6 -1.603775 3.850384 10.802107
1 -1.678824 2.765639 10.642462
1 -1.096765 4.027072 11.762638
1 -2.626249 4.249808 10.884057
6 -0.719939 6.032521 9.891698
1 -1.713240 6.505223 9.933898
1 -0.208581 6.238547 10.844076
1 -0.153594 6.514130 9.081830
1 0.277433 3.235011 7.528093
1 -3.363521 5.150215 8.816690

9t8
77 -1.399553 2.891881 4.744550
6 -2.848170 1.243498 4.467472
6 -1.538548 0.672026 4.222053
6 -1.008005 1.296011 3.066401
6 -2.012663 2.197413 2.530646
6 -3.150198 2.131724 3.365348
6 -4.479820 2.795506 3.179169
6 -1.822699 3.023343 1.293936
6 0.302814 1.001671 2.401276
6 -0.899042 -0.421698 5.022357
6 -3.844247 0.782757 5.489771
1 1.062483 0.683418 3.128306
1 0.685385 1.888215 1.876287
1 0.193247 0.197579 1.655655
1 0.195914 -0.373624 4.958689
1 -1.217324 -1.409784 4.653654
1 -1.175948 -0.351515 6.082152
1 -3.342274 0.407196 6.390919
1 -4.479187 -0.025537 5.091606
1 -4.501425 1.607784 5.796814
1 -4.484369 3.461503 2.307446
1 -4.761503 3.391388 4.059804
1 -5.261908 2.035059 3.028271
1 -0.947734 3.686862 1.389883
1 -2.698233 3.654861 1.095188
1 -1.662660 2.382572 0.412898
6 1.059785 6.691010 2.865216
6 -0.139926 6.838639 3.558627

1 -0.639852 7.810754 3.570878
6 -0.728278 5.758798 4.221487
6 -0.079754 4.512975 4.174492
6 1.131718 4.375552 3.482920
6 1.711314 5.451836 2.807316
1 -1.031866 2.637996 6.236378
1 -0.033072 3.695255 5.194574
6 -3.136788 5.966757 3.716358
1 -3.055125 5.121683 3.016079
1 -4.159397 5.985254 4.122974
1 -2.973950 6.893007 3.146148
6 3.003718 5.276772 2.031749
1 3.350001 4.246287 2.214541
6 2.764001 5.430563 0.526525
1 2.414189 6.447590 0.290915
1 3.690997 5.252656 -0.038728
1 2.000274 4.722749 0.173219
6 4.097497 6.231728 2.516083
1 4.276923 6.115623 3.594044
1 5.041551 6.042549 1.984060
1 3.815702 7.279613 2.331451
1 1.637895 3.406095 3.487847
1 1.492548 7.555992 2.356577
6 -2.340677 4.519245 5.654012
1 -3.422547 4.324249 5.748021
1 -1.964499 4.665985 6.676876
6 -2.105846 5.807311 4.839840
1 -2.182524 6.692866 5.495588

8
77 -0.458109 2.557049 4.343996
6 -2.946732 3.327238 5.792286
6 -1.545632 3.320370 5.902679
6 0.992956 4.410494 3.968520
6 0.506623 3.974420 2.701042
6 0.938403 2.618847 2.489340
6 1.700112 2.220461 3.634205
6 1.724049 3.324689 4.557365
6 2.534519 3.393383 5.817902
6 2.454589 0.934698 3.797055
6 0.721878 1.824132 1.236444
6 -0.273332 4.788806 1.717016
6 0.809942 5.767008 4.577013
1 -0.437311 1.553892 5.527643
6 -0.959325 3.874319 7.044788
6 -1.724967 4.468372 8.056824
6 -3.115756 4.485625 7.914165
6 -3.719748 3.914807 6.793754
1 -4.808083 3.935071 6.700925
1 -0.283270 1.992486 0.825901
1 0.828211 0.748350 1.426381
1 1.451269 2.104040 0.460028
1 -0.998411 4.168456 1.171783
1 0.398233 5.249274 0.975565
1 -0.829243 5.595715 2.212654
1 -0.030669 6.299355 4.113799
1 1.714079 6.382080 4.447596
1 0.596324 5.694510 5.652971
1 2.461610 2.466005 6.401803
1 2.202247 4.222297 6.456703
1 3.597832 3.563869 5.585620
1 1.947150 0.105078 3.287920
1 2.553989 0.666229 4.856849
1 3.468080 1.022148 3.374697
1 -1.124297 1.172226 4.136489
6 -4.820079 3.212246 4.055844
1 -4.774060 4.302997 3.912422
1 -5.099834 2.750661 3.097292
1 -5.625183 2.998333 4.774157
6 -1.052394 5.089727 9.267843
1 0.027670 4.887128 9.174459
6 -1.534691 4.458853 10.576507
1 -1.385455 3.370042 10.569412
1 -0.992225 4.879578 11.436514
1 -2.607452 4.650405 10.731696
6 -1.243075 6.609470 9.288077
1 -2.309869 6.864899 9.381698
1 -0.711092 7.062043 10.138589
1 -0.870020 7.066934 8.360488
1 0.127583 3.840571 7.167218
1 -3.739141 4.946534 8.684417
6 -3.474676 2.678221 4.538070
1 -3.581455 1.595579 4.739436
6 -2.362766 2.851591 3.506385
1 -2.522409 2.216135 2.621633
1 -2.356503 3.904626 3.174268

5t6
77 -0.393676 2.680918 4.493663
6 -2.553756 3.357498 5.924201
6 -1.194473 3.402694 6.194283
6 0.978819 4.511544 3.956269
6 0.373102 4.150551 2.726643
6 0.748725 2.790901 2.419925
6 1.614202 2.326262 3.449294
6 1.730382 3.373009 4.437207
6 2.659884 3.375555 5.614171
6 2.346994 1.018772 3.479423
6 0.388212 2.065264 1.157780
6 -0.426615 5.035267 1.821093
6 0.896575 5.836914 4.650101
1 -0.318036 1.436476 5.416640
6 -0.739185 3.921970 7.404263
6 -1.671151 4.408766 8.336521
6 -3.040882 4.351629 8.038223
6 -3.503172 3.817717 6.830735
1 -4.574898 3.776702 6.619616
6 -2.676119 2.740080 4.548423
1 -0.643887 2.284548 0.851373
1 0.476356 0.978406 1.283221
1 1.050674 2.363568 0.329403
1 -1.262896 4.493449 1.358371
1 0.205548 5.422027 1.005621
1 -0.840432 5.898824 2.357927
1 0.055644 6.433126 4.272896
1 1.820129 6.417686 4.499659
1 0.747333 5.707876 5.731751
1 2.706988 2.387061 6.090041
1 2.327753 4.095989 6.373593
1 3.681099 3.657959 5.311459
1 1.785304 0.232776 2.958146
1 2.512781 0.682663 4.510992
1 3.330262 1.113770 2.992001
1 -1.564427 1.699394 4.098933
6 -3.569016 1.494397 4.571930
1 -4.591435 1.796204 4.854682
1 -3.620389 1.006029 3.586624
1 -3.214561 0.764758 5.312972
6 -3.137324 3.708346 3.471322
1 -3.001358 3.280780 2.465155
1 -4.213337 3.919694 3.601392
1 -2.592404 4.659845 3.529220
1 -3.763596 4.728105 8.766482
1 0.327085 3.951091 7.650446
6 -1.198633 4.989825 9.658563
1 -0.098909 4.914055 9.666762
6 -1.730341 4.189259 10.850577
1 -1.450709 3.129376 10.771156
1 -1.331449 4.585078 11.796711
1 -2.828635 4.244661 10.900024
6 -1.565957 6.471216 9.784026
1 -1.169311 6.895653 10.718644
1 -1.164751 7.051086 8.940746
1 -2.658924 6.602515 9.792039

8t6
77 -0.430374 2.690345 4.311794
6 -2.916829 3.364819 5.818312
6 -1.510283 3.339351 5.904453
6 1.055257 4.513253 4.085525
6 0.626986 4.188244 2.777969
6 1.032591 2.826561 2.489388
6 1.718278 2.321672 3.623959
6 1.688184 3.340768 4.651260
6 2.471586 3.300607 5.930655
6 2.426331 1.006062 3.741580
6 0.840610 2.136881 1.171793
6 -0.101026 5.077813 1.817592
6 0.900518 5.824613 4.791314
1 -0.550588 1.411682 5.189271
6 -0.918829 3.818419 7.080785
6 -1.664441 4.359639 8.134703
6 -3.055817 4.405871 8.007317
6 -3.671446 3.904184 6.862180
1 -4.760765 3.935516 6.786778
1 -0.156016 2.338661 0.754021
1 0.946397 1.049128 1.273742
1 1.583212 2.480594 0.434380
1 -0.854162 4.515886 1.245597
1 0.592441 5.527809 1.089789
1 -0.617496 5.894956 2.338351
1 0.206662 6.486113 4.257425
1 1.869721 6.341531 4.868069
1 0.507488 5.683510 5.808998
1 2.303037 2.365655 6.482213
1 2.194154 4.134572 6.588930
1 3.551165 3.387610 5.728402
1 1.965714 0.242354 3.101707
1 2.400688 0.637815 4.775248
1 3.482297 1.107215 3.444335
1 -1.560424 1.742070 3.693123
6 -4.844621 3.385619 4.134268
1 -4.716809 4.467414 3.975710
1 -5.196265 2.936472 3.193492
1 -5.636744 3.244538 4.882731
6 -2.487307 2.802576 3.465521

1 -2.808357 2.198030 2.601028
1 -2.361379 3.830238 3.092621
1 -3.668661 4.828290 8.807359
1 0.166953 3.763641 7.195403
6 -0.970350 4.897534 9.372899
1 0.104137 4.676075 9.260016
6 -1.458429 4.207563 10.649078
1 -1.334759 3.117644 10.581349
1 -0.900090 4.567987 11.526174
1 -2.525220 4.415195 10.823699
6 -1.126546 6.417871 9.476580
1 -0.579006 6.812297 10.346012
1 -0.749132 6.916164 8.571996
1 -2.186830 6.691237 9.591646
6 -3.529096 2.750600 4.580101
1 -3.721246 1.683568 4.800595

6
77 -0.248638 1.465234 5.387495
6 -2.368539 3.513971 5.796294
6 -1.187744 2.971362 6.357003
6 1.254201 2.711793 4.193697
6 0.725799 1.813410 3.260824
6 1.007994 0.443482 3.706709
6 1.721119 0.503792 4.912475
6 1.774239 1.915006 5.313378
6 2.587183 2.466043 6.442798
6 2.317110 -0.629012 5.687527
6 0.643389 -0.779394 2.922701
6 0.000529 2.129878 1.989784
6 1.228844 4.207454 4.168481
1 -0.535024 0.694559 6.728753
6 -0.683759 3.561888 7.524893
6 -1.284739 4.666153 8.139592
6 -2.447869 5.185684 7.566425
6 -2.980335 4.609714 6.414463
1 -3.892106 5.035868 5.992845
6 -2.924561 2.890863 4.521411
1 -0.381696 -0.710223 2.532389
1 0.706703 -1.682986 3.541855
1 1.317684 -0.908838 2.061108
1 -0.940666 1.564519 1.918176
1 0.612583 1.859187 1.114994
1 -0.239644 3.198035 1.917887
1 0.570008 4.583163 3.375206
1 2.238034 4.612598 3.996117
1 0.857326 4.603948 5.125376
1 2.523915 1.816222 7.325954
1 2.225825 3.462631 6.729320
1 3.646517 2.554196 6.152240
1 1.810444 -1.576346 5.463749
1 2.232973 -0.450345 6.767016
1 3.384793 -0.743892 5.442580
1 -2.164629 3.046014 3.732962
6 -4.229090 3.513017 4.030939
1 -4.111433 4.585317 3.822467
1 -4.562891 3.024141 3.104250
1 -5.027215 3.397597 4.780447
6 -3.113076 1.377891 4.681625
1 -2.221038 0.804063 5.034239
1 -3.887485 1.165110 5.434406
1 -3.405251 0.907989 3.730576
6 -0.690039 5.275585 9.395879
1 0.200375 4.678164 9.652443
6 -1.662634 5.197946 10.575864
1 -2.567293 5.792311 10.375269
1 -1.976222 4.161098 10.761271
1 -1.198734 5.591491 11.492961
6 -0.234578 6.717845 9.155282
1 0.483056 6.772450 8.324180
1 -1.092428 7.359030 8.900232
1 0.242328 7.135341 10.054991
1 -2.951030 6.043904 8.018810
1 0.212083 3.137560 7.988457

6t2l
77 -0.282778 1.543640 4.659321
6 -2.675013 3.126301 5.834367
6 -1.305063 2.774450 5.947828
6 1.505903 2.754298 4.008088
6 0.830672 2.132205 2.902800
6 0.956605 0.682982 2.999086
6 1.544280 0.407904 4.247977
6 1.846784 1.692855 4.897174
6 2.551218 1.805743 6.211046
6 1.860457 -0.920337 4.855415
6 0.485803 -0.294753 1.969316
6 0.222586 2.833068 1.730692
6 1.680959 4.218644 4.261128
6 -0.601918 3.271825 7.054910
6 -1.185765 4.052660 8.061259
6 -2.534164 4.379537 7.927403
6 -3.257035 3.925593 6.821309
1 -4.309972 4.202309 6.741209
6 -3.480490 2.673180 4.627782
1 -0.471987 0.021241 1.534663
1 0.338795 -1.288806 2.410020
1 1.216043 -0.387260 1.150182
1 -0.709279 2.340097 1.421867
1 0.918562 2.811935 0.877272
1 -0.004850 3.880714 1.962495
1 0.955991 4.805476 3.682255
1 2.691719 4.558471 3.987729
1 1.509683 4.454874 5.320944
1 2.021386 1.226036 6.982250
1 2.615964 2.850638 6.539562
1 3.575741 1.412487 6.119143
1 1.238552 -1.715228 4.424350
1 1.676641 -0.891661 5.938764
1 2.918611 -1.178827 4.691834
1 -3.125661 1.654667 4.388684
6 -3.173285 3.562044 3.416591
1 -2.091511 3.590473 3.220158
1 -3.683106 3.194139 2.512343
1 -3.508315 4.593842 3.606904
6 -4.987394 2.599394 4.866247
1 -5.223986 2.015110 5.767331
1 -5.427634 3.601568 4.983773
1 -5.484855 2.125542 4.007412
6 -0.344293 4.442297 9.265193
1 0.635288 4.770162 8.875570
6 -0.098534 3.214343 10.151293
1 -1.051921 2.856680 10.572985
1 0.344618 2.390788 9.569828
1 0.576785 3.454484 10.986816
6 -0.929904 5.589091 10.083959
1 -1.141159 6.466856 9.456509
1 -1.868912 5.286056 10.572144
1 -0.230125 5.891124 10.876528
1 -3.037173 4.992284 8.677135
1 0.451963 3.004390 7.178957
1 -1.390694 0.408667 4.975944
15 -0.376789 -0.209055 8.183142
6 -0.667922 -1.854058 7.377372
1 0.240264 -2.471120 7.445335
1 -1.503215 -2.397044 7.846233
1 -0.897237 -1.689179 6.314131
6 -0.226282 -0.772811 9.945695
1 -0.219803 0.104233 10.608941
1 -1.057197 -1.433216 10.238610
1 0.723142 -1.309471 10.084817
6 -2.118174 0.410883 8.212537
1 -2.783289 -0.290833 8.739299
1 -2.158011 1.398667 8.695689
1 -2.461914 0.542194 7.176873

2l
77 -0.268400 1.226664 5.118645
6 -2.314816 3.467387 5.714044
6 -1.224964 2.691768 6.180731
6 0.900755 2.650825 3.736431
6 0.710895 1.431390 3.014759
6 1.414984 0.389618 3.696611
6 2.077042 0.984839 4.833069
6 1.770622 2.370588 4.858151
6 2.290831 3.408068 5.804787
6 2.996743 0.259032 5.768910
6 1.587303 -1.020333 3.213783
6 -0.050699 1.273477 1.732727
6 0.410692 4.016544 3.358821
6 -0.689559 3.042723 7.437147
6 -1.198940 4.053236 8.256136
6 -2.307879 4.765858 7.792280
6 -2.836914 4.476151 6.539280
1 -3.687958 5.063299 6.186524
6 -2.958383 3.242457 4.351177
1 0.666676 -1.404545 2.752980
1 1.852434 -1.696204 4.038742
1 2.391633 -1.093093 2.463263
1 -0.477472 0.265515 1.647300
1 0.607215 1.437725 0.864587
1 -0.878417 1.992540 1.668246
1 -0.365097 3.960815 2.583021
1 1.232236 4.637234 2.967038
1 -0.023614 4.535081 4.227497
1 2.648728 2.958474 6.741455
1 1.504904 4.132049 6.064103
1 3.130486 3.964491 5.357610
1 2.635242 -0.758270 5.981479
1 3.095138 0.790385 6.725382
1 4.005202 0.162788 5.336012
1 -2.207916 2.736596 3.726982
6 -3.351230 4.539273 3.638550
1 -2.517017 5.255820 3.619465
1 -3.646593 4.325425 2.600037
1 -4.208887 5.032070 4.121214

6 -4.169063 2.311656 4.469535
1 -3.879858 1.349861 4.915781
1 -4.938220 2.766498 5.113790
1 -4.618726 2.114634 3.483906
6 -0.556874 4.369034 9.594044
1 0.267307 3.648965 9.733269
6 -1.541764 4.184823 10.751695
1 -2.382502 4.890055 10.664723
1 -1.958135 3.167214 10.759002
1 -1.050123 4.366773 11.719293
6 0.045254 5.777099 9.604829
1 0.772546 5.900795 8.789767
1 -0.740966 6.536015 9.471097
1 0.553450 5.981520 10.559415
1 -2.751943 5.560758 8.396905
1 0.195783 2.506202 7.794387
1 -1.690232 0.844512 4.603225
15 -0.866528 -0.248240 6.655766
6 -2.578653 -0.121987 7.285620
1 -3.276018 -0.307077 6.457683
1 -2.755655 -0.852812 8.087228
1 -2.743866 0.898251 7.659641
6 -0.769481 -1.990879 6.090077
1 0.270603 -2.220366 5.818487
1 -1.106088 -2.690510 6.868996
1 -1.391584 -2.104740 5.191979
6 0.112970 -0.308804 8.209464
1 -0.162208 -1.187031 8.811350
1 1.183192 -0.351914 7.964619
1 -0.080292 0.601732 8.791558

8isoTS
77 -0.667872 2.752216 4.143331
6 -2.927195 3.706376 5.808926
6 -1.555673 3.392287 5.869081
6 0.530031 4.467770 3.400546
6 0.448327 3.545821 2.283158
6 1.122272 2.361356 2.655389
6 1.714274 2.561778 3.972223
6 1.387599 3.866468 4.398384
6 1.887937 4.579190 5.614470
6 2.611280 1.583774 4.669767
6 1.323308 1.137258 1.812355
6 -0.233712 3.829691 0.981203
6 0.056181 5.889907 3.410923
1 -1.671904 1.519574 4.664875
6 -0.884918 3.565079 7.084167
6 -1.519399 4.096014 8.215030
6 -2.869314 4.448092 8.116055
6 -3.568034 4.243154 6.926590
1 -4.628038 4.503025 6.875523
1 0.545704 1.046378 1.043357
1 1.301386 0.223251 2.420957
1 2.299660 1.171912 1.303427
1 -0.429568 2.905148 0.422968
1 0.391606 4.480926 0.350843
1 -1.195683 4.337831 1.136053
1 -0.777547 6.033089 2.710102
1 0.861591 6.581151 3.115712
1 -0.296933 6.180208 4.410539
1 2.265153 3.880152 6.372310
1 1.099590 5.189772 6.074560
1 2.716175 5.249574 5.334243
1 2.276394 0.549636 4.512372
1 2.631724 1.763953 5.752497
1 3.645186 1.657060 4.295584
1 -0.946589 1.110251 4.301302
6 -4.806590 4.225724 4.148200
1 -4.517698 5.287992 4.124158
1 -5.199865 3.954457 3.157168
1 -5.626116 4.113066 4.873191
6 -0.749911 4.295864 9.508514
1 0.263088 3.891713 9.344945
6 -1.379019 3.525534 10.672285
1 -1.465127 2.455332 10.437421
1 -0.775371 3.634460 11.585943
1 -2.389167 3.904027 10.891430
6 -0.610488 5.783197 9.846974
1 -1.599057 6.235875 10.019854
1 -0.009528 5.925621 10.757927
1 -0.130560 6.332158 9.024081
1 0.165145 3.271736 7.169718
1 -3.388351 4.875631 8.977672
6 -3.603758 3.356218 4.503621
1 -3.959875 2.310513 4.600052
6 -2.517268 3.373493 3.423590
1 -2.825687 2.794232 2.537644
1 -2.370772 4.420038 3.101459

7
77 13.040045 19.440886 19.264001
6 12.937356 14.175596 17.917859
1 13.475426 13.231639 17.980647
6 10.732866 12.886203 17.789289
6 11.542535 14.175620 17.812414
6 10.914362 15.427052 17.725615
1 9.829736 15.471923 17.636711
6 12.613772 21.418166 18.125942
6 11.513237 21.036585 20.138261
6 11.417618 20.654438 17.853476
6 10.764879 20.377889 19.082985
6 11.645275 16.612648 17.754120
6 12.637063 21.689273 19.550368
6 13.049122 16.628062 17.865133
6 13.670006 15.366697 17.934353
6 10.830536 20.391222 16.495682
1 11.572773 20.480943 15.700222
1 10.040814 21.127964 16.288254
1 10.372155 19.400050 16.418892
6 13.468986 22.120108 17.106885
1 14.493652 22.253234 17.466897
1 13.068900 23.118167 16.873567
1 13.523384 21.564412 16.167024
6 11.014100 21.222263 21.543684
1 10.582905 20.305683 21.960493
1 10.222488 21.985092 21.569414
1 11.806760 21.559632 22.217167
6 9.416831 19.732428 19.240345
1 9.240078 18.966616 18.478604
1 8.606908 20.472339 19.150262
1 9.304372 19.253472 20.217985
6 13.586244 22.631058 20.238717
1 13.703259 22.387102 21.298716
1 13.225400 23.667897 20.173886
1 14.580180 22.598345 19.783959
1 9.675391 13.171174 17.696275
15 13.078944 17.987750 21.021144
6 14.310205 16.613992 21.163606
1 14.192777 15.902231 20.345474
1 15.324219 17.022682 21.140202
1 14.161851 16.093394 22.116467
6 13.430264 18.829020 22.637734
1 14.410781 19.311295 22.576705
1 12.683211 19.596729 22.846025
1 13.436631 18.106873 23.462574
6 11.504171 17.089285 21.398974
1 10.690538 17.809133 21.528728
1 11.254629 16.437533 20.557110
1 11.596869 16.489074 22.311586
1 14.593377 19.600180 19.548290
1 14.751128 15.299344 17.998249
6 13.821845 17.946248 17.822325
6 15.339010 17.736471 17.994826
1 15.741759 17.058851 17.224285
1 15.857426 18.693349 17.881658
1 15.617612 17.329804 18.969870
6 13.658906 18.497741 16.385300
1 12.614997 18.608225 16.090786
1 14.161151 19.464110 16.274046
1 14.119992 17.801781 15.664932
6 11.080982 12.009133 16.571257
1 12.125306 11.676468 16.607412
1 10.447165 11.114208 16.541778
1 10.939817 12.559822 15.634826
6 10.880131 12.089301 19.099807
1 10.593381 12.696125 19.966037
1 10.246893 11.193606 19.085159
1 11.915988 11.762146 19.250502
1 11.119157 17.561354 17.708524

3t6
77 -0.744965 2.438597 3.859943
6 -2.223862 0.983486 3.188432
6 -0.973519 0.384545 3.595967
6 -0.035771 0.870267 2.579534
6 -0.818308 1.252430 1.360819
6 -2.128404 1.331755 1.729070
6 -3.301241 1.804404 0.927002
6 -0.180068 1.622114 0.057563
6 1.415881 0.479070 2.507387
6 -0.733758 -0.656860 4.651112
6 -3.548856 0.763444 3.870668
1 1.848249 0.363207 3.505666
1 2.006198 1.229593 1.969689
1 1.538879 -0.476312 1.974504
1 0.284173 -0.598396 5.048691
1 -0.875621 -1.668391 4.238672
1 -1.423567 -0.539287 5.492342
1 -3.421176 0.615643 4.947199
1 -4.064061 -0.122584 3.468839
1 -4.219059 1.619458 3.731074
1 -3.025458 2.024386 -0.108739
1 -3.732755 2.720040 1.355289
1 -4.107006 1.057857 0.906895
1 0.499382 2.478233 0.175590
1 -0.924423 1.896731 -0.696209

1 0.418665 0.797268 -0.352850
6 0.654973 6.791690 2.790158
6 -0.390441 6.492629 3.664796
1 -1.138656 7.252682 3.872455
6 -0.477144 5.236084 4.274093
6 0.500297 4.269262 3.936449
6 1.559938 4.591240 3.070160
6 1.654200 5.853946 2.481376
6 -1.570520 4.878680 5.281971
1 -1.645603 3.762071 5.348754
1 0.667586 3.386868 4.687261
1 2.304864 3.828617 2.858845
1 0.702576 7.781702 2.341214
6 2.790083 6.211647 1.531018
1 2.632697 7.253407 1.219284
6 4.163438 6.140631 2.225334
1 4.959447 6.457648 1.540954
1 4.195070 6.787171 3.109175
1 4.392303 5.118750 2.550730
6 2.768120 5.342574 0.258995
1 1.804388 5.418724 -0.256447
1 3.554806 5.655917 -0.437863
1 2.936990 4.285549 0.497543
6 -1.179093 5.342584 6.699050
1 -1.077552 6.434626 6.727081
1 -1.942586 5.050206 7.429414
1 -0.223895 4.905829 7.008366
6 -2.962582 5.393793 4.887485
1 -3.233738 5.068222 3.878174
1 -3.716937 5.015237 5.586396
1 -3.009709 6.488891 4.918689

4t7
77 13.712668 20.058168 18.949548
6 11.776345 18.469845 15.080852
1 10.853387 17.887399 15.153385
6 11.074528 19.220549 12.753213
6 12.051306 19.198176 13.912867
6 13.247703 19.912561 13.872880
1 13.498671 20.482871 12.974539
6 13.243784 22.068942 18.982643
6 11.844819 20.700301 20.191460
6 12.205343 21.574960 18.067264
6 11.409775 20.684994 18.790269
6 14.132193 19.919191 14.955307
6 12.895393 21.617112 20.333391
6 13.852225 19.217506 16.135169
6 12.651783 18.477298 16.158996
6 12.087179 21.950922 16.626813
1 13.074444 22.152670 16.190872
1 11.475979 22.861407 16.518405
1 11.629072 21.147727 16.032675
6 14.221205 23.156029 18.661321
1 15.083085 23.117982 19.339971
1 13.748912 24.147713 18.752586
1 14.597476 23.048526 17.635161
6 11.152091 19.939832 21.277186
1 10.901830 18.924041 20.939245
1 10.208845 20.437837 21.554386
1 11.773363 19.867170 22.179556
6 10.271301 19.856213 18.284429
1 10.218413 19.882907 17.188418
1 9.314025 20.224844 18.686798
1 10.388612 18.806864 18.596916
6 13.577186 22.043141 21.595749
1 13.494582 21.271356 22.372127
1 13.131766 22.972063 21.985855
1 14.645309 22.225233 21.421262
1 11.538330 19.820541 11.953159
15 11.986864 16.519995 19.997105
6 13.249627 16.857060 21.309613
1 14.202379 17.148133 20.844395
1 12.914738 17.703029 21.926111
1 13.412603 15.979780 21.954404
6 10.637532 15.816309 21.055250
1 10.202150 16.611451 21.677628
1 9.839983 15.414459 20.414549
1 11.008112 15.014325 21.711987
6 12.671493 14.944925 19.303794
1 11.942679 14.505009 18.608483
1 13.589252 15.160252 18.738167
1 12.900668 14.214011 20.094311
1 15.102440 19.919545 19.679105
1 12.385610 17.911426 17.057107
6 14.757488 19.242607 17.344906
6 14.890555 17.824461 17.941723
1 14.936580 17.053419 17.153430
1 15.783340 17.747979 18.575972
1 14.017875 17.554049 18.571243
6 16.129314 19.832684 17.043255
1 16.059986 20.898215 16.776860
1 16.775255 19.762748 17.927932
1 16.624609 19.307444 16.205736
6 10.814538 17.817750 12.197804
1 10.334416 17.179119 12.955150
1 10.146168 17.860851 11.324884
1 11.752464 17.332219 11.894064
6 9.763208 19.904905 13.152648
1 9.946549 20.923625 13.523769
1 9.073221 19.965592 12.297571
1 9.258697 19.340323 13.952570
1 15.054358 20.494528 14.869905

10
77 -0.582022 2.438799 4.373823
6 -3.188107 3.425029 5.875345
6 -1.781579 3.272530 5.890044
6 0.595573 4.469185 3.855958
6 0.012867 3.948326 2.652414
6 0.581564 2.643340 2.408247
6 1.515153 2.360599 3.470787
6 1.516399 3.493159 4.369973
6 2.455902 3.699505 5.524445
6 2.457139 1.190528 3.533456
6 0.370300 1.814675 1.171634
6 -0.898974 4.690244 1.716535
6 0.345488 5.823945 4.450778
1 -0.335894 1.570093 5.685731
6 -1.100877 3.764550 7.023059
6 -1.724662 4.418685 8.091936
6 -3.114084 4.575827 8.037170
6 -3.821198 4.080306 6.947361
1 -4.902966 4.192839 6.919510
6 -4.089250 2.953476 4.771527
1 -0.651963 1.908128 0.794628
1 0.554694 0.754352 1.363512
1 1.051644 2.132017 0.369861
1 -1.552148 4.008147 1.165673
1 -0.315087 5.257598 0.978081
1 -1.535211 5.402689 2.248538
1 -0.563709 6.277226 4.047637
1 1.181869 6.502419 4.234444
1 0.228535 5.768901 5.537396
1 2.723296 2.753341 6.003110
1 2.020348 4.349735 6.288328
1 3.387491 4.173642 5.184368
1 2.030069 0.305593 3.054151
1 2.696026 0.922705 4.566411
1 3.401562 1.423985 3.022251
1 -0.990183 0.911926 4.171607
6 -4.660806 1.743772 4.804446
1 -5.347064 1.417239 4.025595
1 -4.451425 1.037699 5.603154
1 -2.069093 2.237639 3.845911
6 -4.393249 3.954970 3.681199
1 -5.079397 3.544388 2.932412
1 -4.844423 4.862620 4.104661
1 -3.472917 4.268901 3.173943
6 -0.907780 4.918986 9.276570
1 0.144658 4.678914 9.068807
6 -1.293343 4.199960 10.583475
1 -1.186286 3.114404 10.482494
1 -0.657116 4.531904 11.413394
1 -2.334526 4.407737 10.857773
6 -1.007800 6.447635 9.440573
1 -2.035837 6.756667 9.664314
1 -0.369764 6.794094 10.263038
1 -0.697585 6.965521 8.525871
1 -0.025903 3.613002 7.088191
1 -3.648721 5.069062 8.845692

5t10
77 -0.392615 2.407521 4.616226
6 -3.150713 3.463974 5.812703
6 -1.723954 3.235121 5.964984
6 0.850464 4.363698 3.905009
6 0.002981 3.850483 2.847488
6 0.473300 2.528982 2.499141
6 1.563098 2.206900 3.378204
6 1.803382 3.361905 4.225681
6 2.922991 3.482495 5.219815
6 2.445326 0.992961 3.287761
6 0.012953 1.703359 1.330061
6 -1.040979 4.647977 2.118983
6 0.748948 5.737676 4.501769
1 0.113198 1.724343 5.971082
6 -1.158556 3.793621 7.139730
6 -1.857819 4.496490 8.119741
6 -3.243402 4.684015 7.944631
6 -3.855933 4.195839 6.811327
1 -4.928794 4.329808 6.697839
6 -3.861725 3.115020 4.613468
1 -1.038123 1.890049 1.091723
1 0.119596 0.633410 1.529721
1 0.602696 1.934874 0.431546
1 -1.722393 4.001913 1.558315

1 -0.578502 5.341392 1.401553
1 -1.644881 5.246784 2.808858
1 -0.294477 6.048244 4.615005
1 1.249832 6.484805 3.869759
1 1.211077 5.782367 5.492338
1 3.156217 2.518664 5.681080
1 2.679794 4.185632 6.021728
1 3.839477 3.844281 4.732248
1 1.899395 0.132523 2.890874
1 2.836835 0.706772 4.268553
1 3.306119 1.173600 2.627396
1 -1.018650 0.949940 4.688456
6 -3.405949 2.035103 3.815835
1 -3.859031 1.930889 2.827424
1 -3.285062 1.073884 4.314824
1 -2.154527 2.244530 3.790511
6 -4.986380 3.983313 4.115462
1 -5.120192 3.860627 3.035331
1 -5.938624 3.685763 4.582229
1 -4.835475 5.043682 4.342875
6 -1.138130 5.036387 9.346417
1 -0.078292 4.766410 9.244323
6 -1.657022 4.386759 10.644126
1 -1.566760 3.295884 10.602636
1 -1.087154 4.745125 11.510142
1 -2.712511 4.628822 10.816996
6 -1.218997 6.573211 9.428513
1 -2.255037 6.912480 9.546965
1 -0.646972 6.945221 10.287286
1 -0.816867 7.039561 8.522213
1 -0.092102 3.653379 7.296162
1 -3.831451 5.204690 8.696171

10. NMR Spectra

1,4-Diisopropylbenzene-d_2

^{1}H NMR (500 MHz, $CDCl_3$)

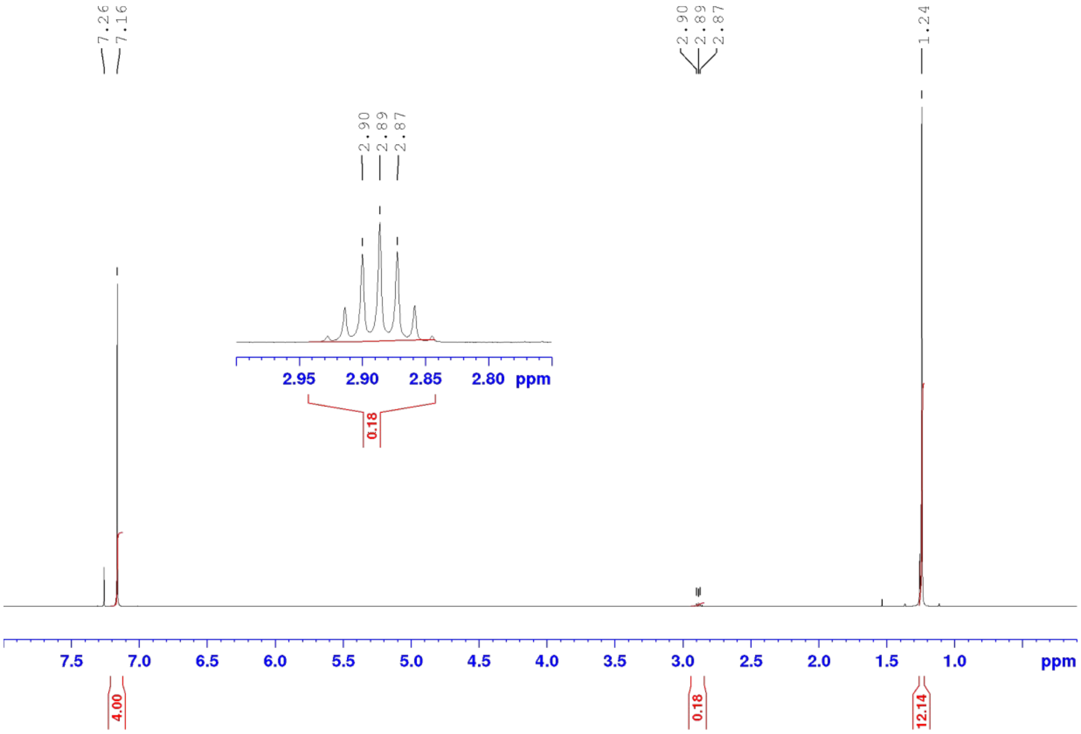

$^{13}C\{^1H\}$ NMR (126 MHz, $CDCl_3$)

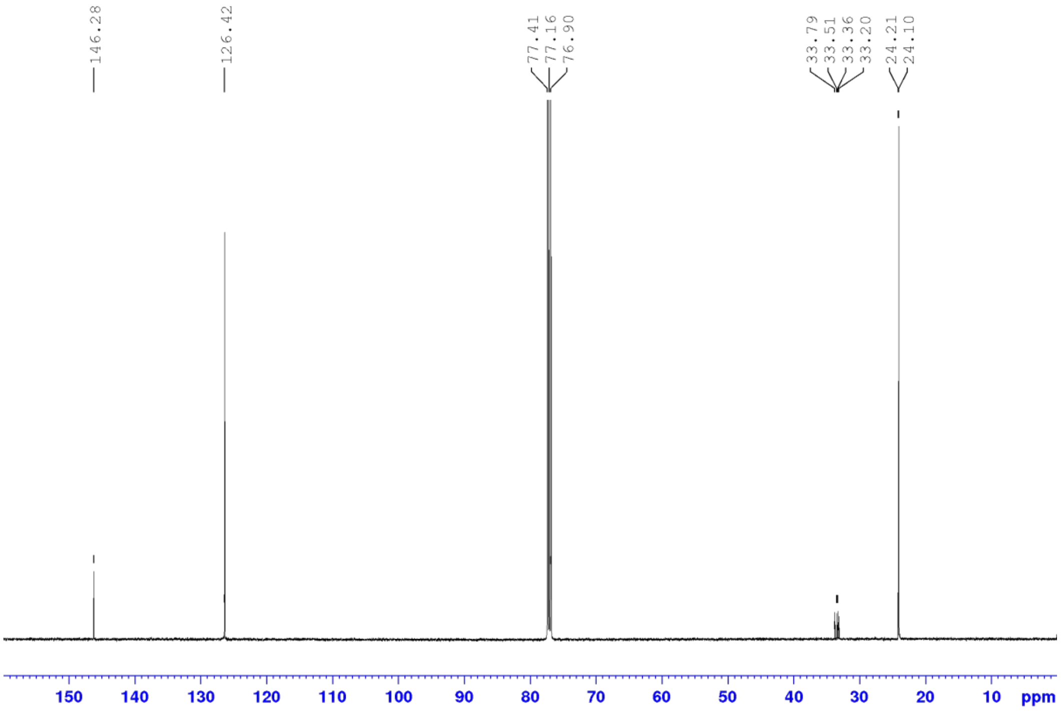

HSQC (500 MHz, $CDCl_3$)

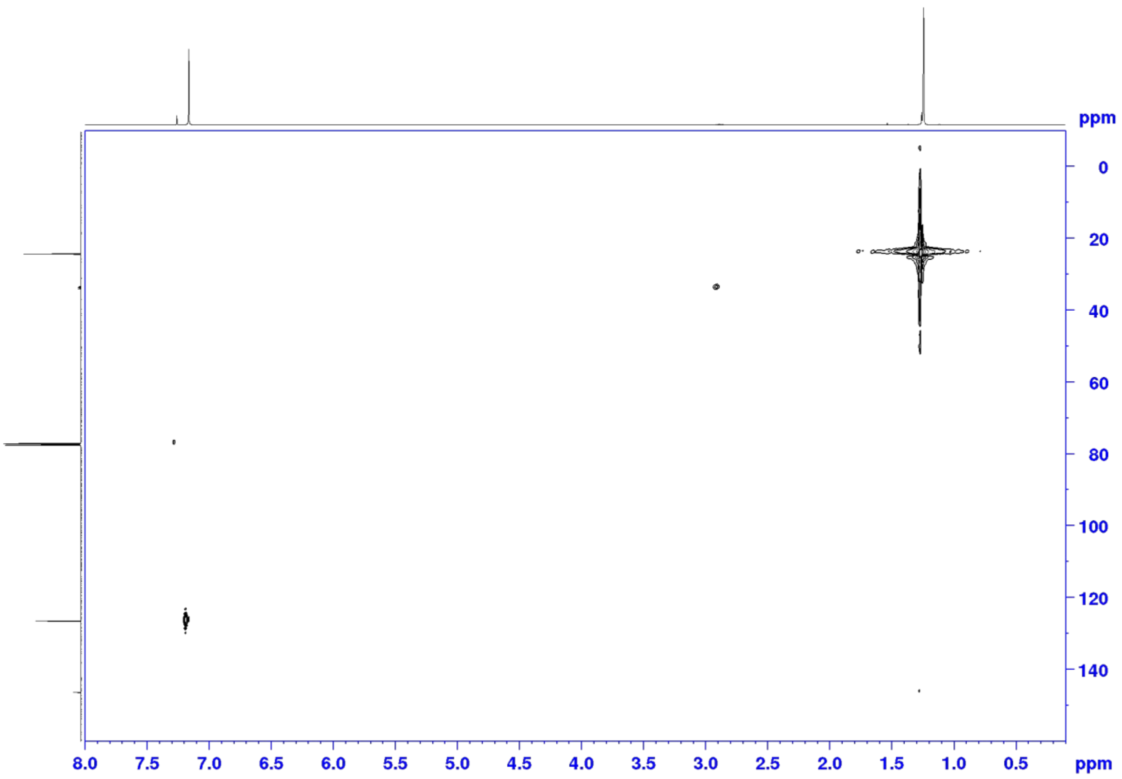

1,4-Diisopropylbenzene-d_4

^1H NMR (500 MHz, $CDCl_3$)

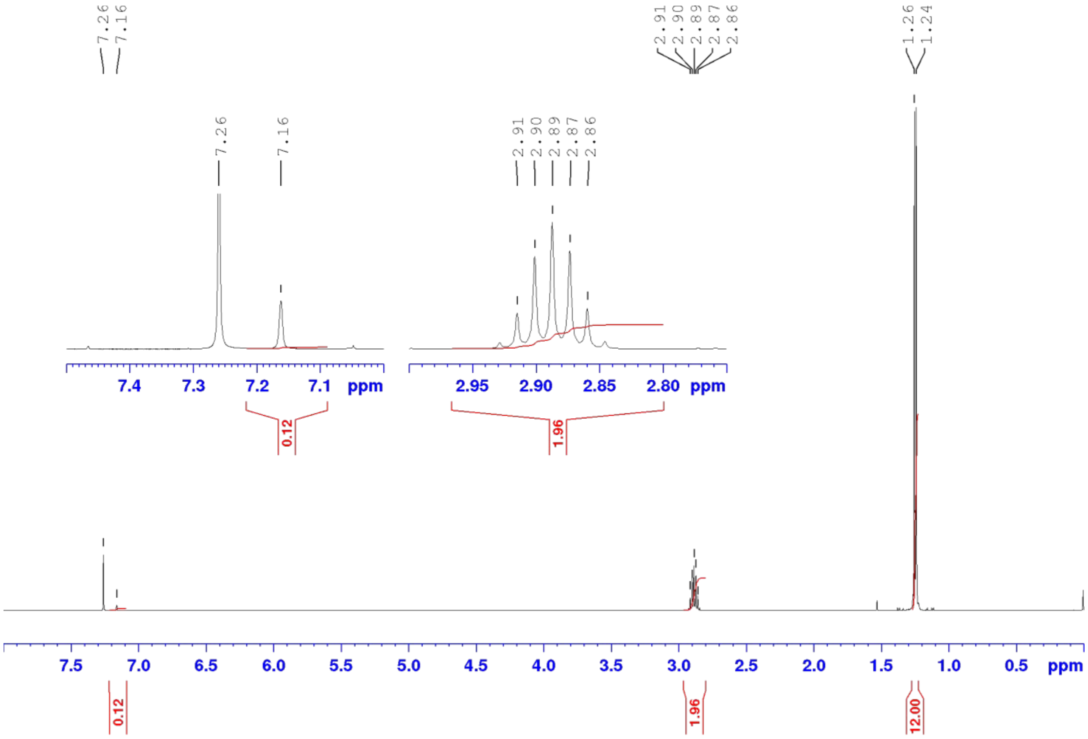

$^{13}C\{^1H\}$ NMR (126 MHz, $CDCl_3$)

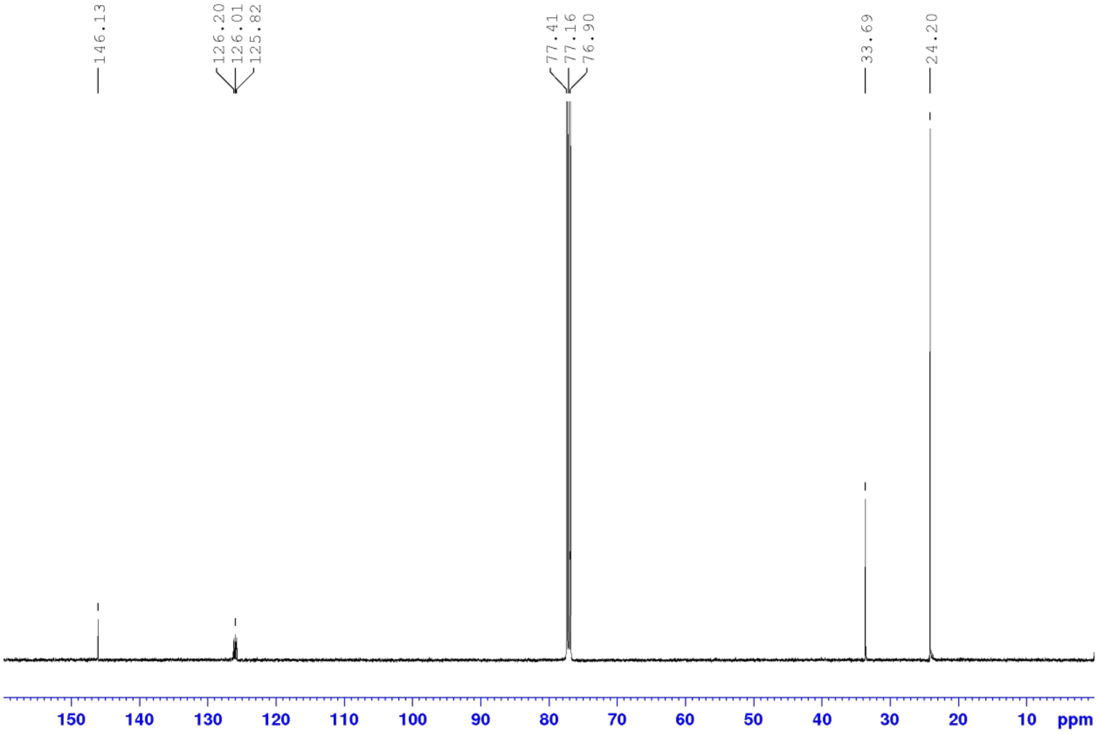

HSQC (500 MHz, $CDCl_3$)

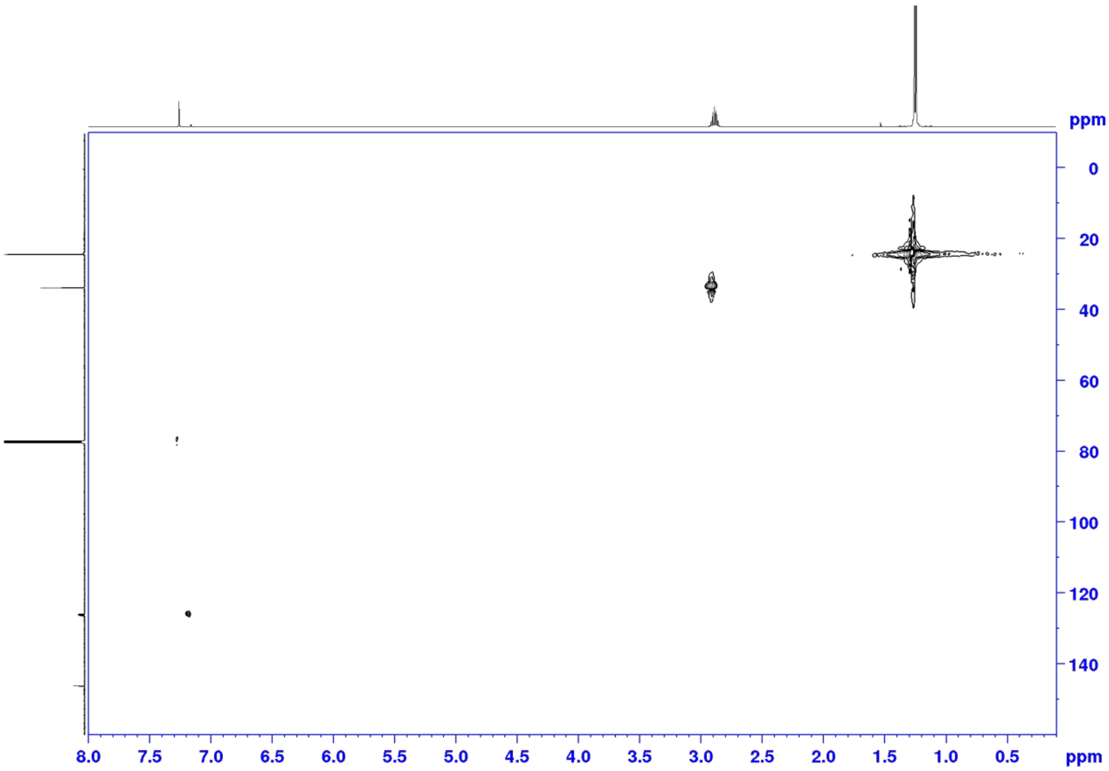

[Cp*Ir(η^6-ethylbenzene)][BF_4]$_2$

^1H NMR (400 MHz, d-TFA)

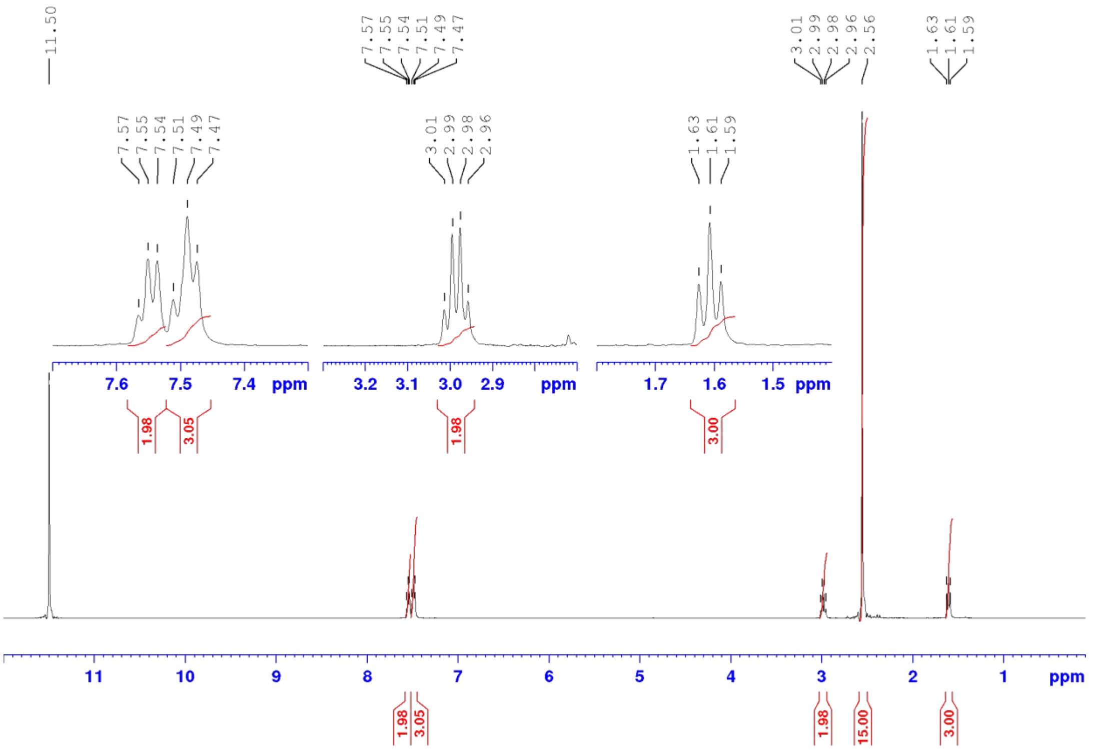

$^{13}C\{^1H\}$ NMR (100 MHz, d-TFA)

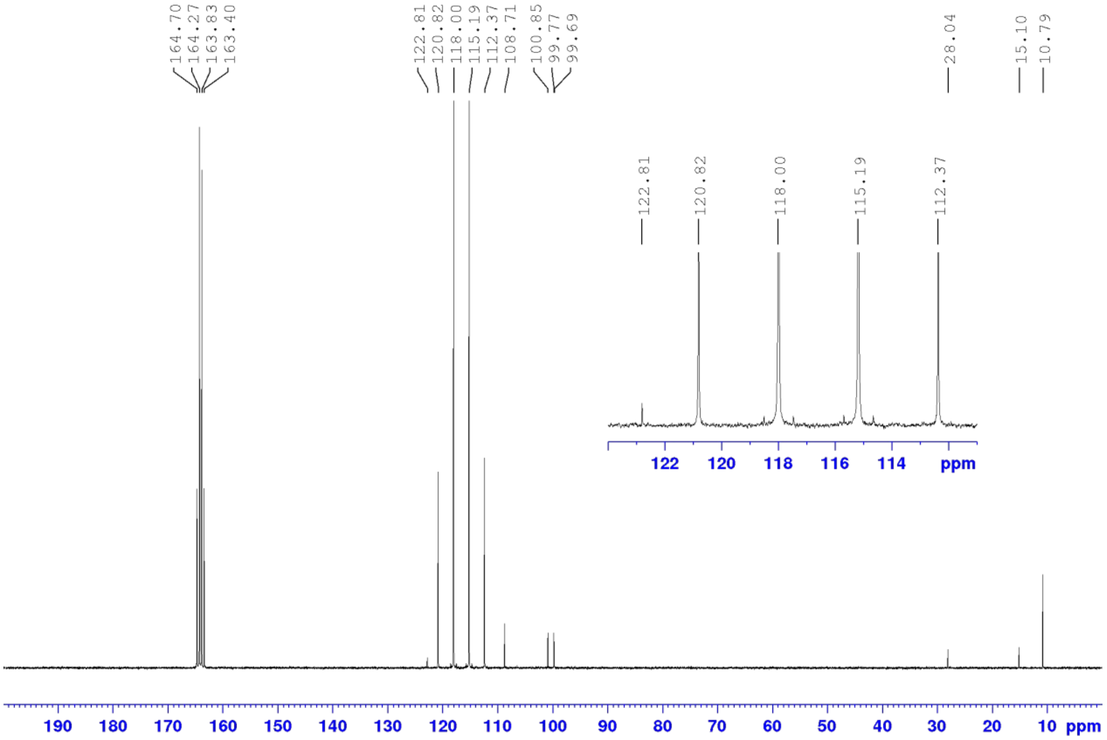

[Cp*Ir(η^6-isopropylbenzene)][BF_4]$_2$

^{1}H NMR (400 MHz, d-TFA)

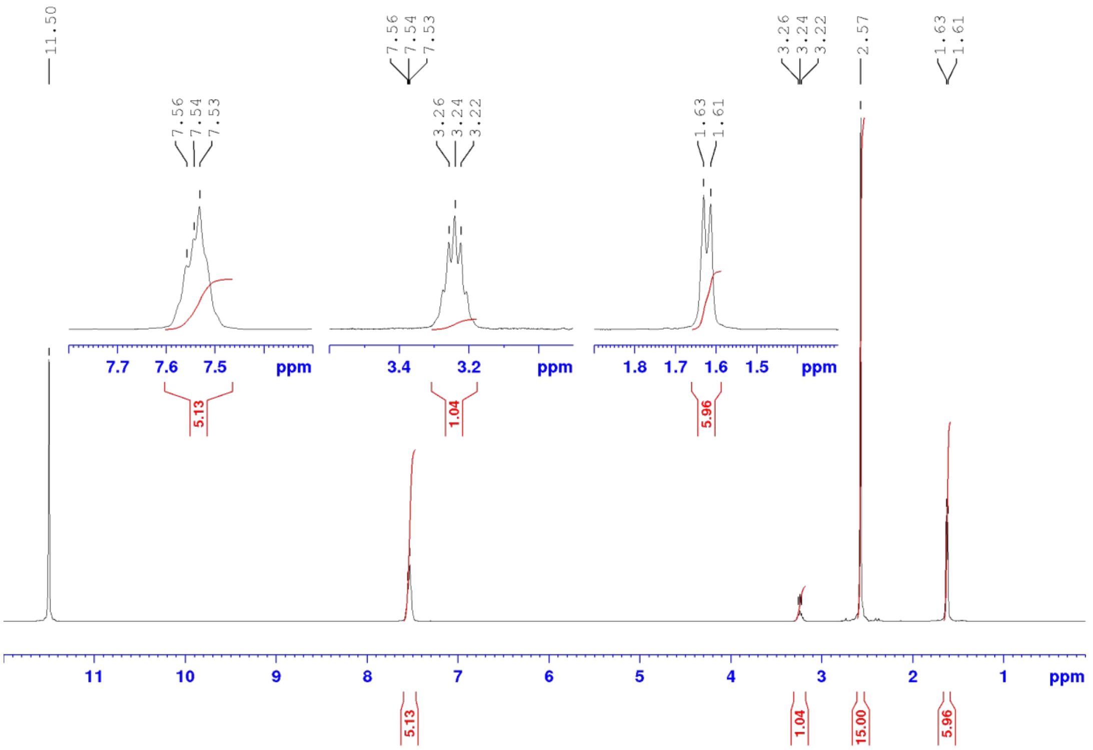

^{13}C{^{1}H} NMR (100 MHz, d-TFA)

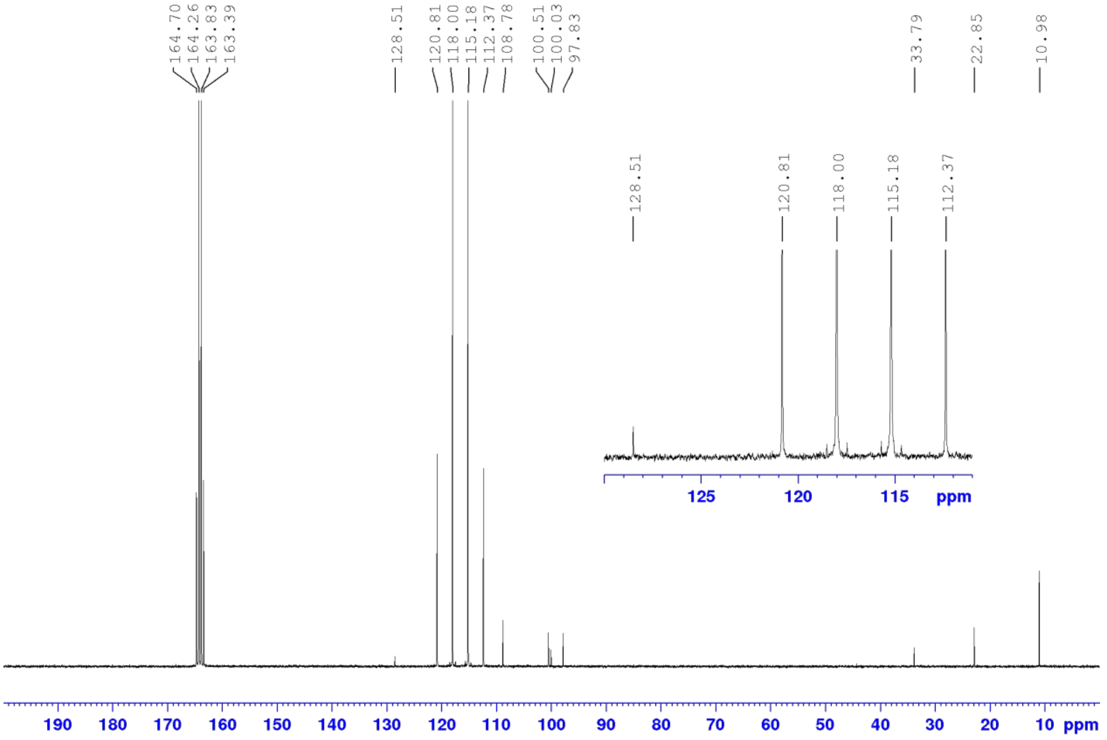

[Cp*Ir(η^6-*sec*-butylbenzene)][BF_4]$_2$

^1H NMR [500 MHz, $(CD_3)_2CO$]

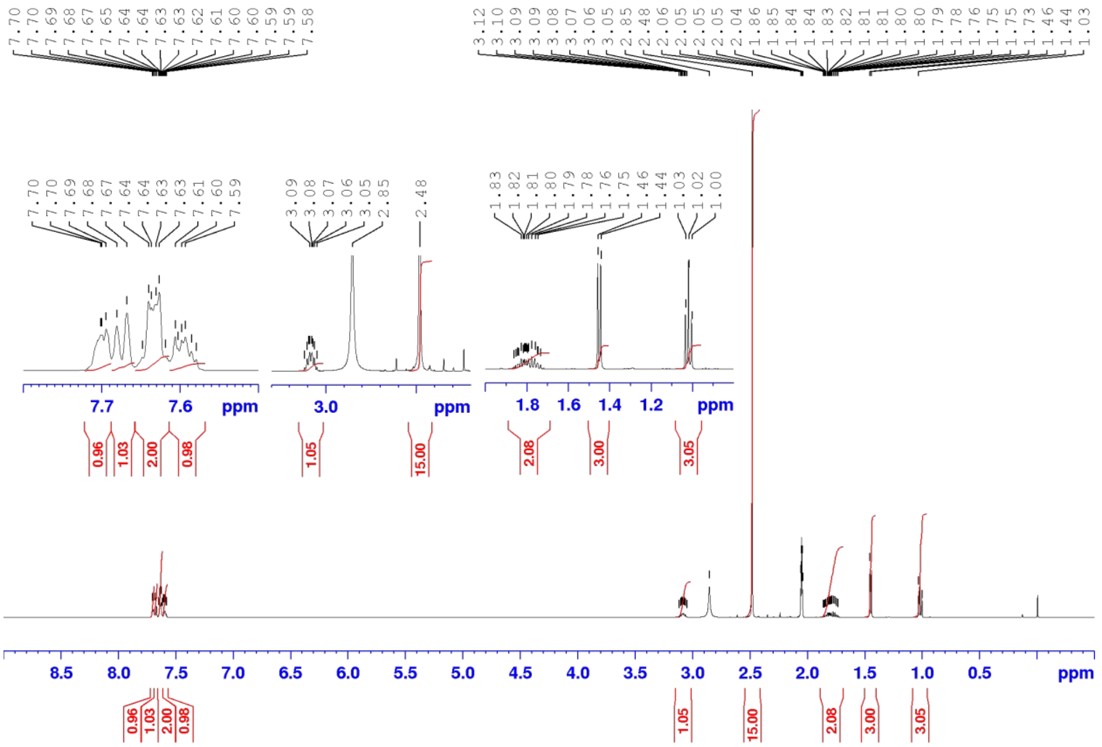

$^{13}C\{^1H\}$ NMR [126 MHz, $(CD_3)_2CO$]

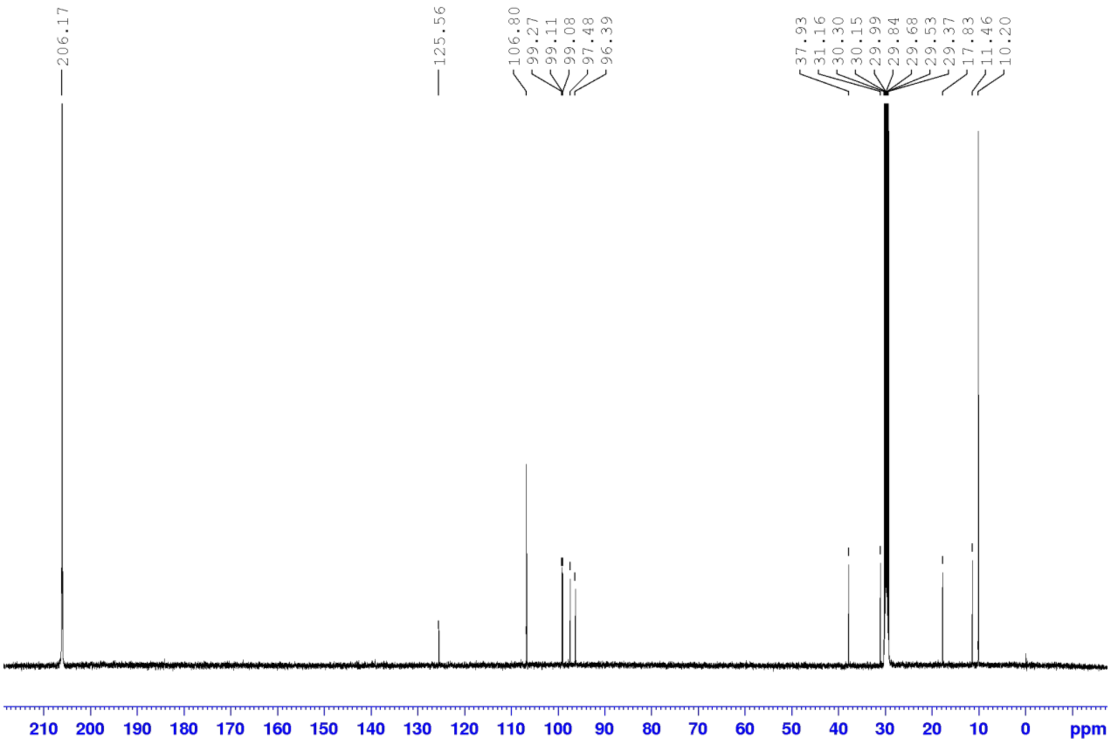

[Cp*Ir(η^6-3-pentylbenzene)][BF_4]$_2$

^{1}H NMR [500 MHz, $(CD_3)_2CO$]

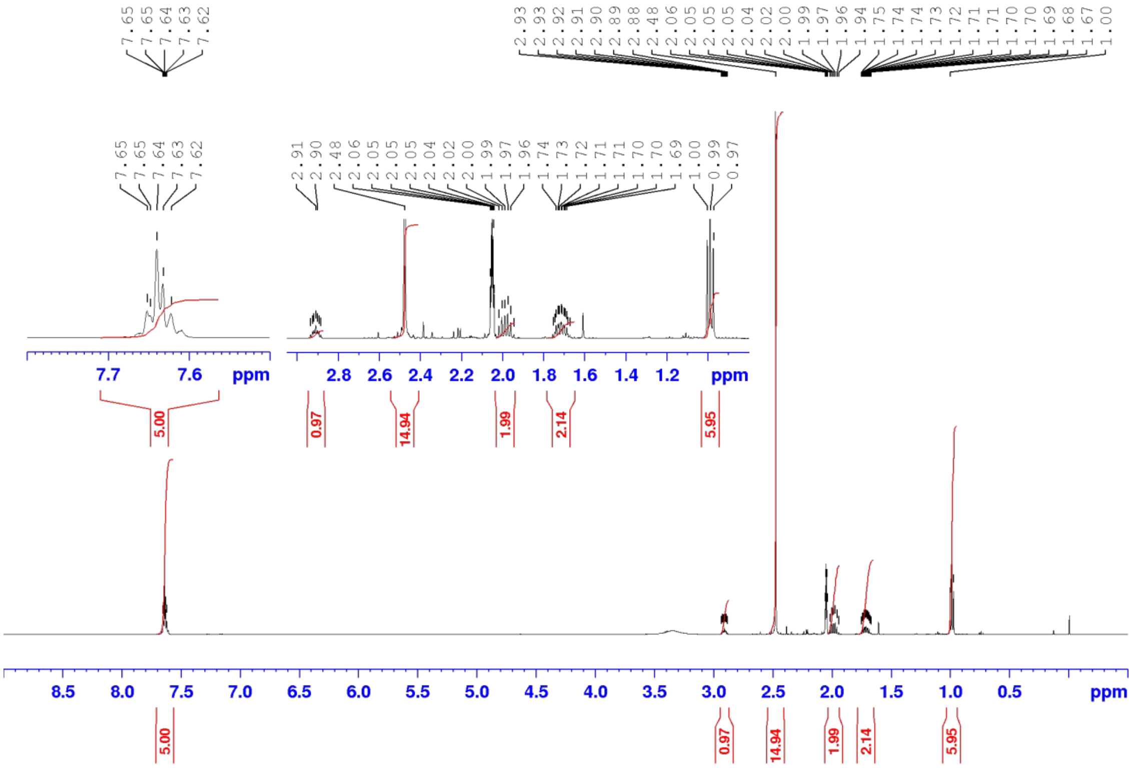

^{13}C{^{1}H} NMR [126 MHz, $(CD_3)_2CO$]

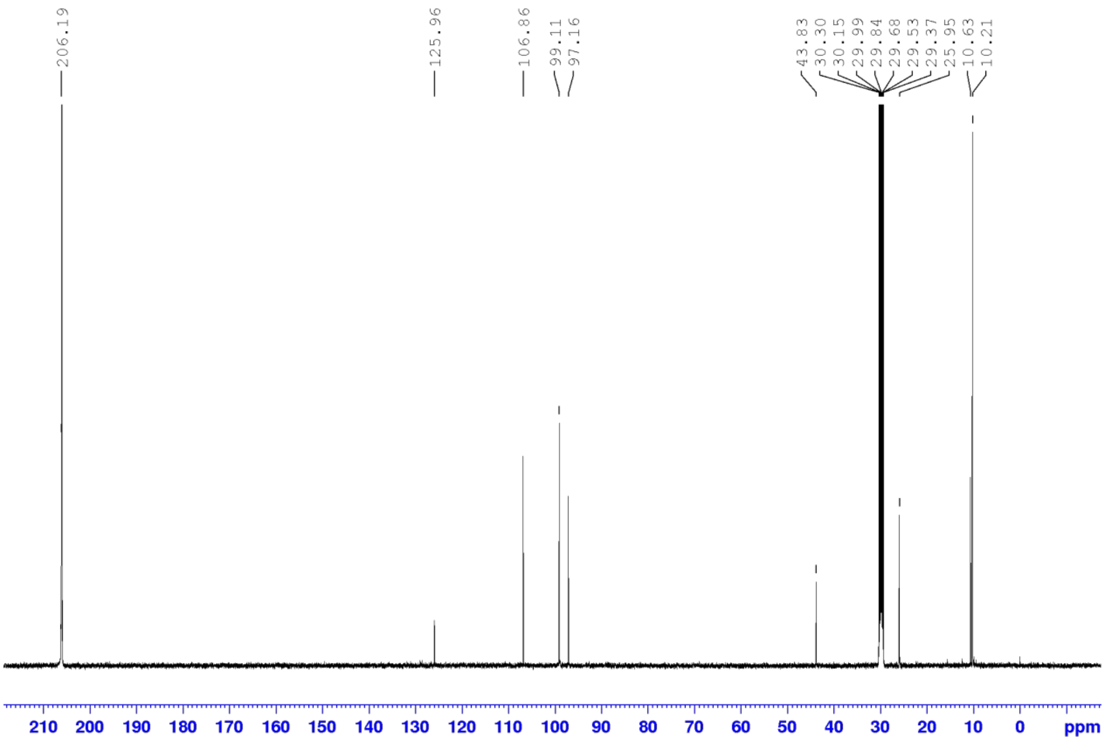

[Cp*Ir(η^6-cyclopentylbenzene)][BF_4]$_2$

^1H NMR [400 MHz, $(CD_3)_2CO$]

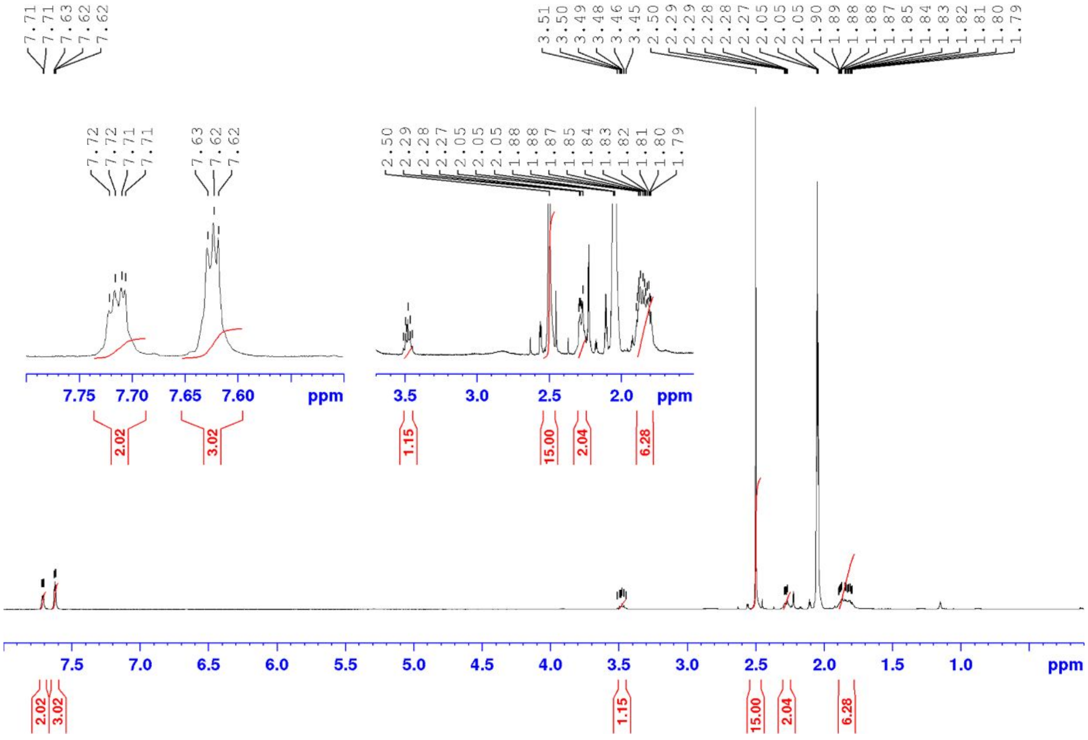

$^{13}C\{^1H\}$ NMR [100 MHz, $(CD_3)_2CO$]

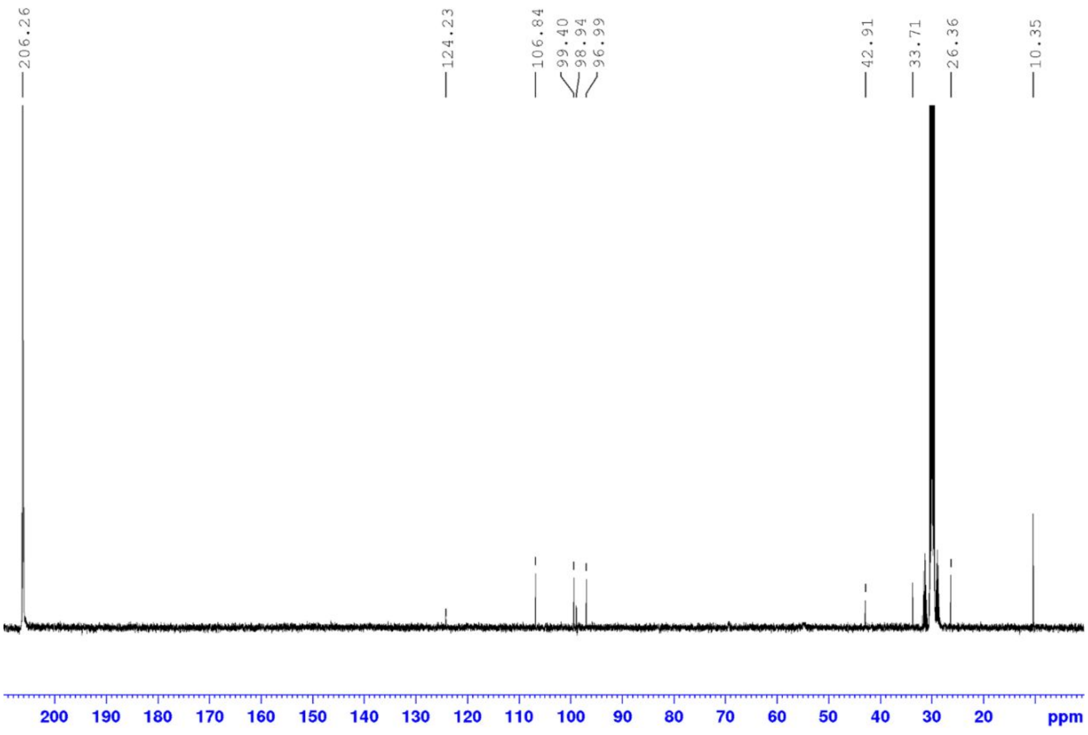

[Cp*Ir(η^6-cyclohexylbenzene)][BF$_4$]$_2$

^{1}H NMR [400 MHz, $(CD_3)_2CO$ + 20 μL TFA]

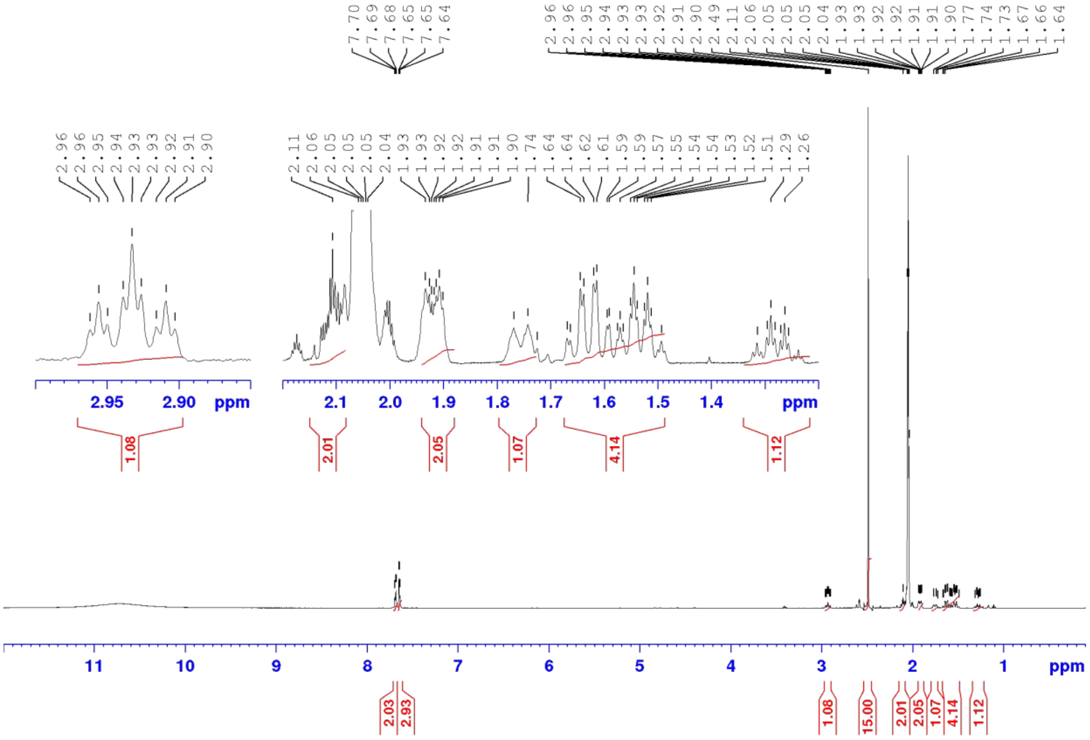

$^{13}C\{^1H\}$ NMR [100 MHz, $(CD_3)_2CO$ + 20 μL TFA]

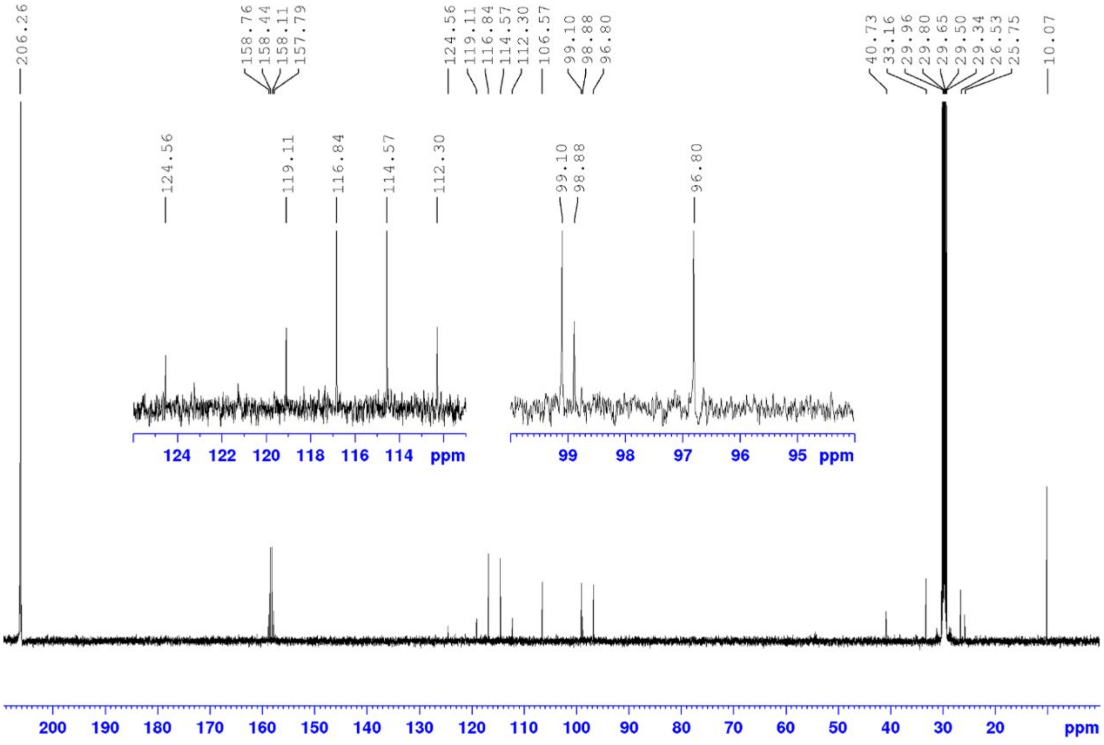

[Cp*Ir(η^6-*n*-propylbenzene)][BF$_4$]$_2$

^{1}H NMR [400 MHz, $(CD_3)_2CO$/TFA (1:1)]

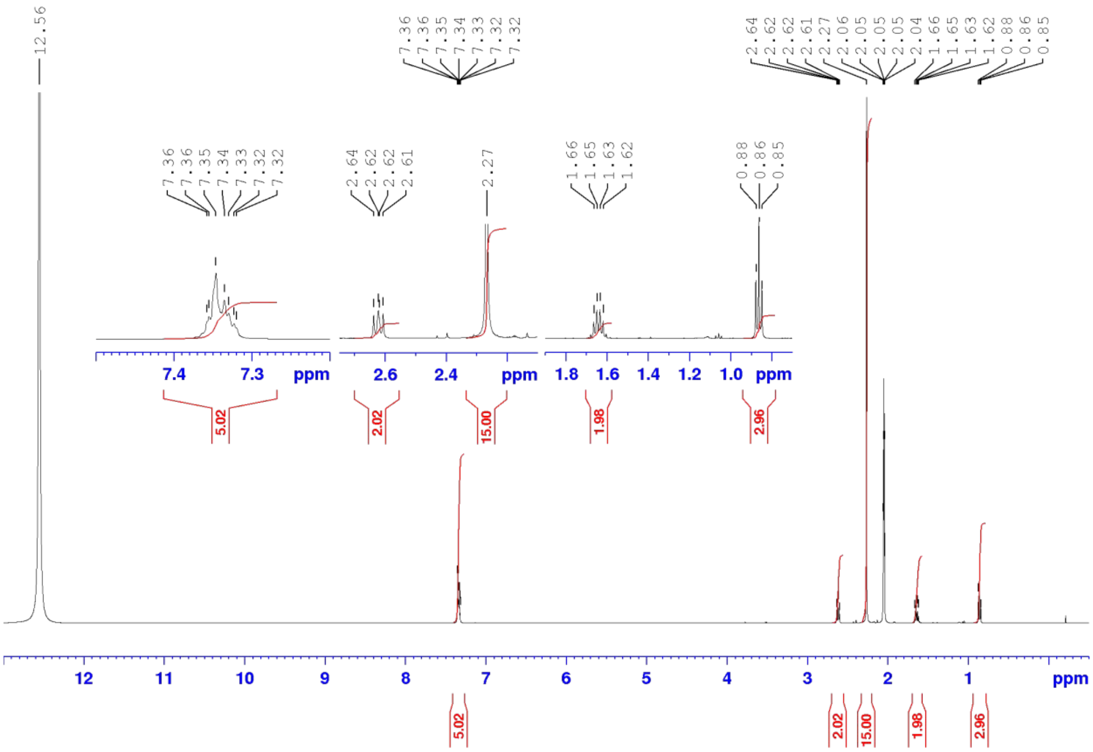

^{13}C{^{1}H} NMR [100 MHz, $(CD_3)_2CO$/TFA (1 : 1)]

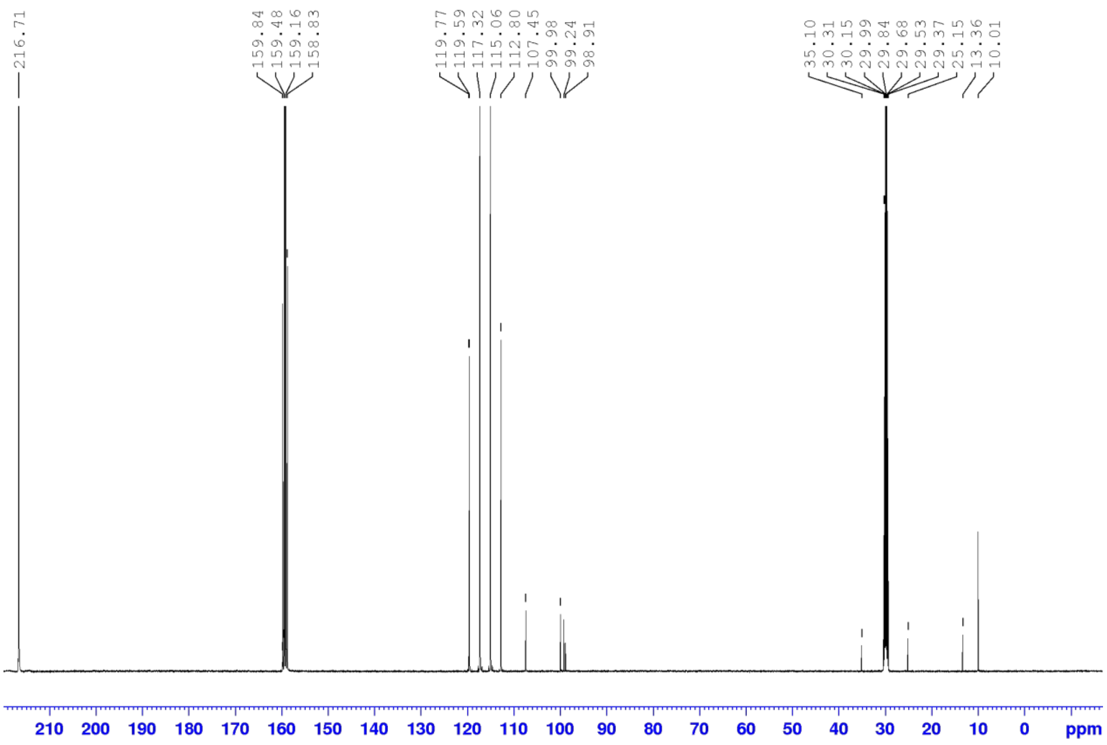

[Cp*Ir(η^6-*n*-butylbenzene)][BF_4]$_2$

^1H NMR [400 MHz, $(CD_3)_2CO$]

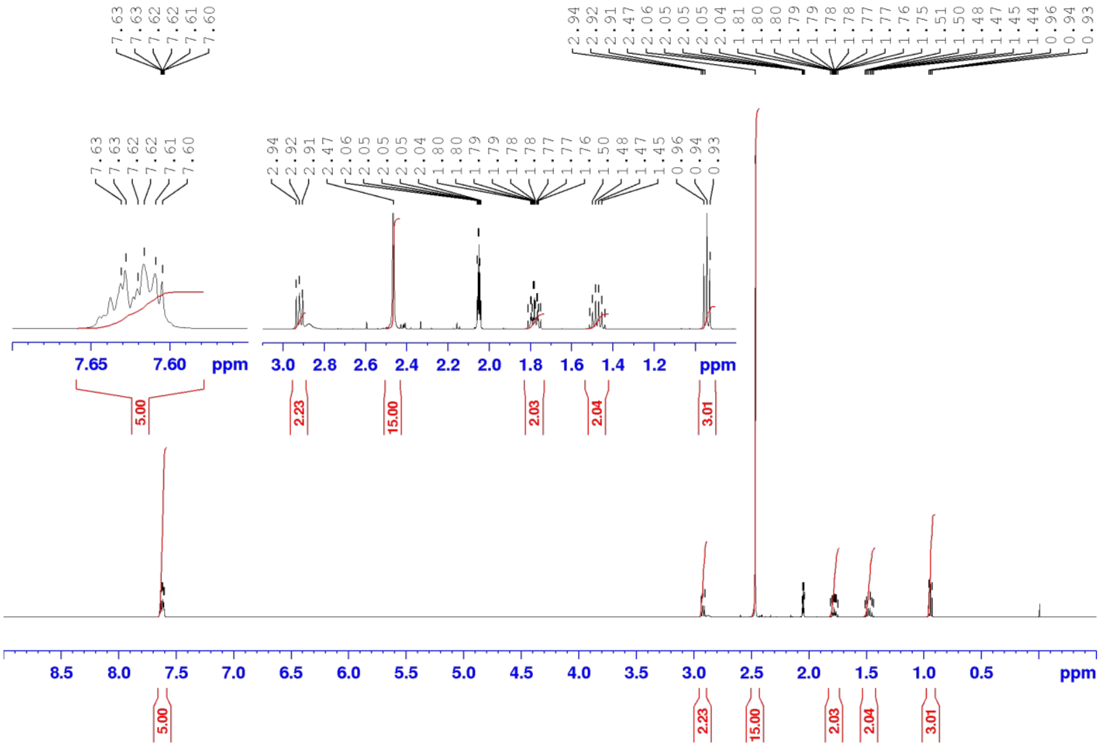

$^{13}C\{^1H\}$ NMR [100 MHz, $(CD_3)_2CO$]

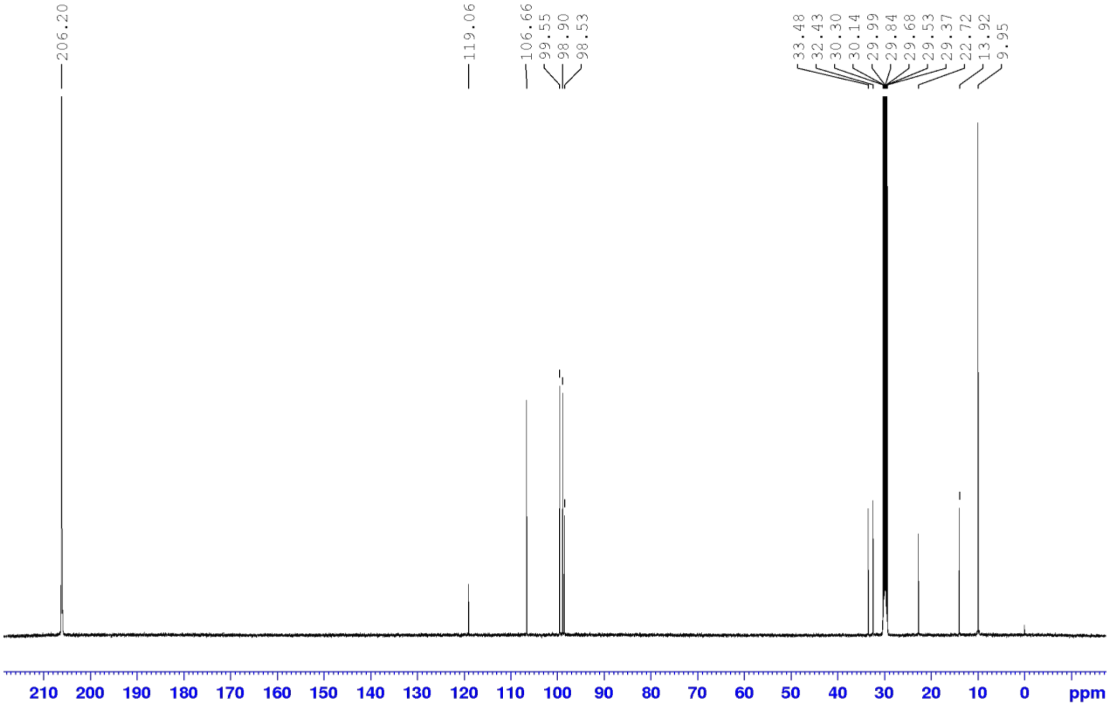

[Cp*Ir(η^6-*iso*-butylbenzene)][BF_4]$_2$

^1H NMR [400 MHz, $(CD_3)_2CO$/TFA (1:1)]

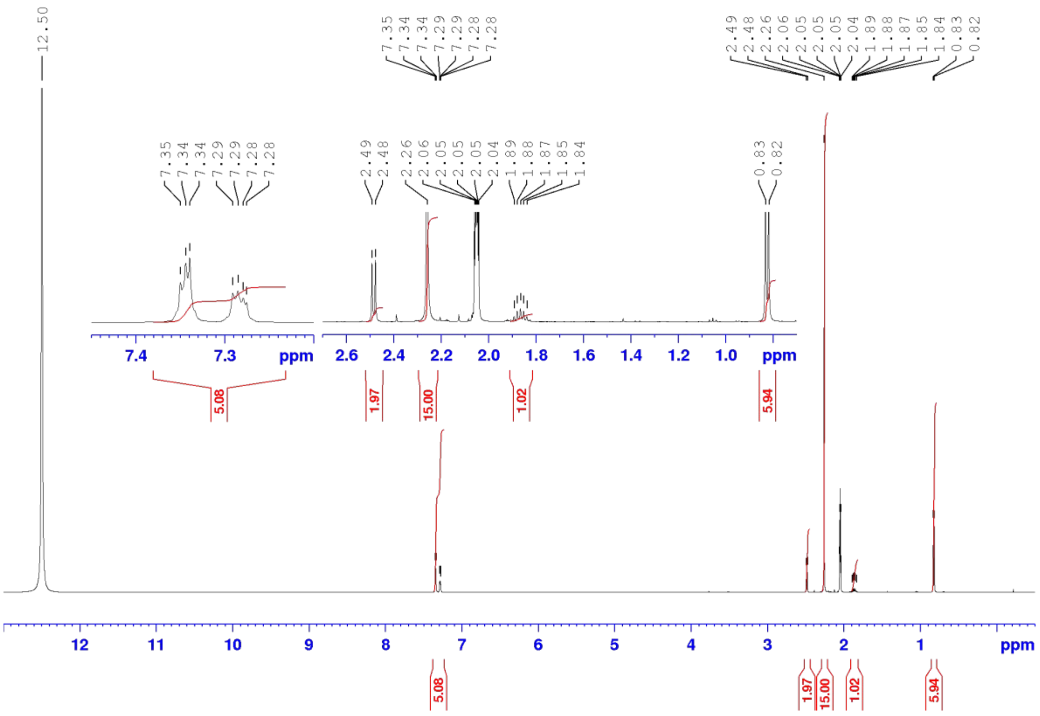

$^{13}C\{^1H\}$ NMR [100 MHz, $(CD_3)_2CO$/TFA (1 : 1)]

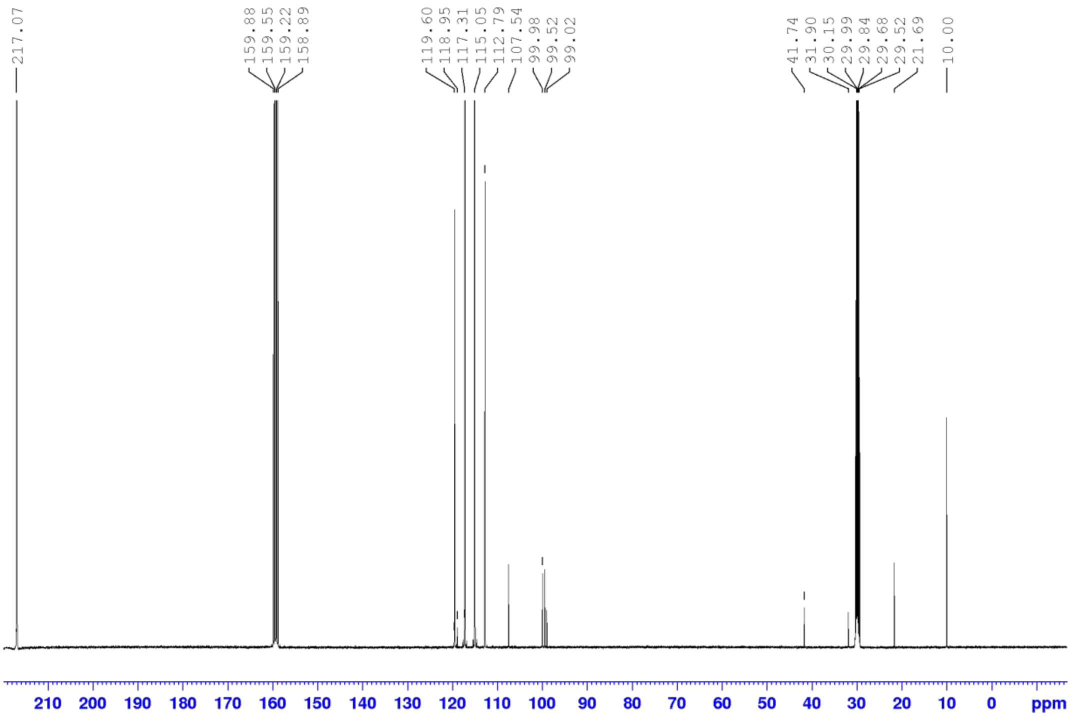

[Cp*Ir(η^6-neopentylbenzene)][BF_4]$_2$

^1H NMR [500 MHz, $(CD_3)_2CO$]

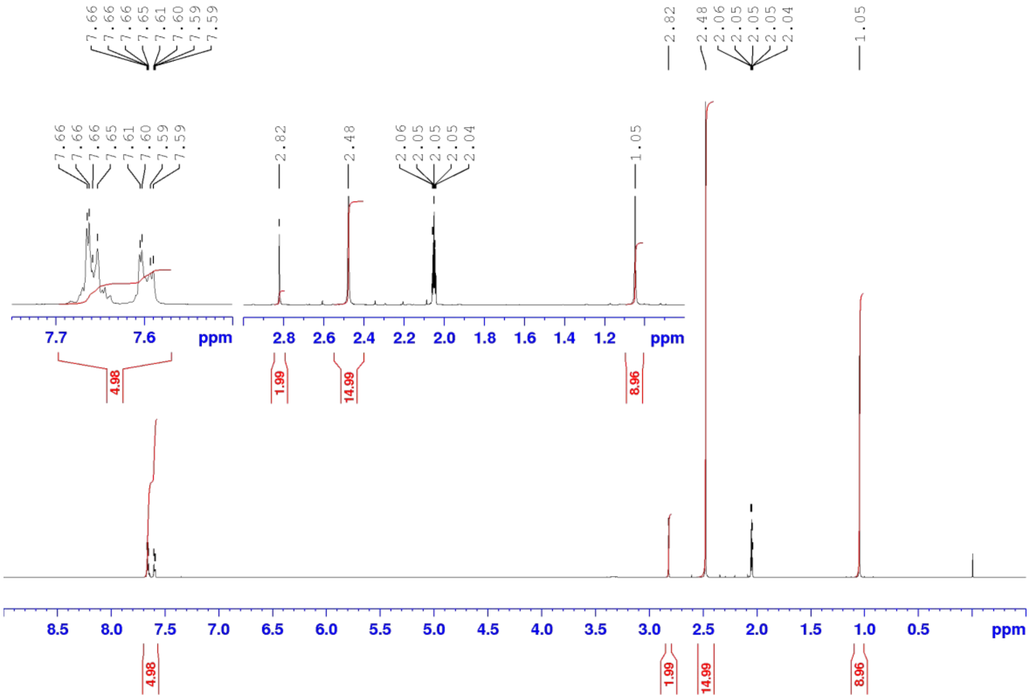

$^{13}C\{^1H\}$ NMR [126 MHz, $(CD_3)_2CO$]

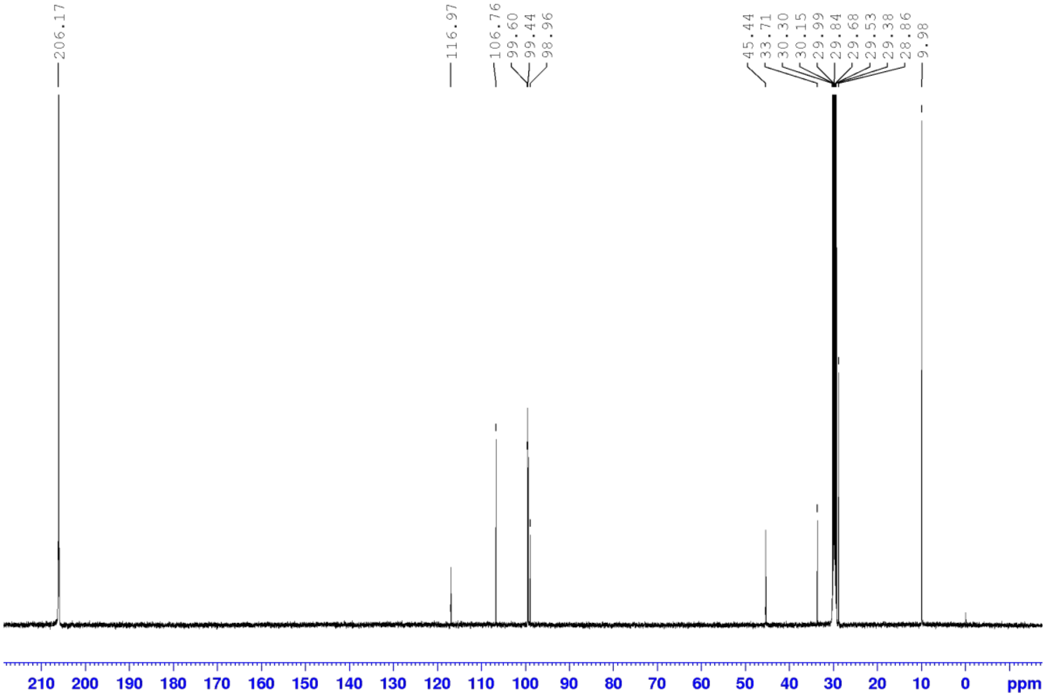

[Cp*Ir(η^6-1,4-diisopropylbenzene)][BF_4]$_2$

^{1}H NMR [400 MHz, $(CD_3)_2CO$]

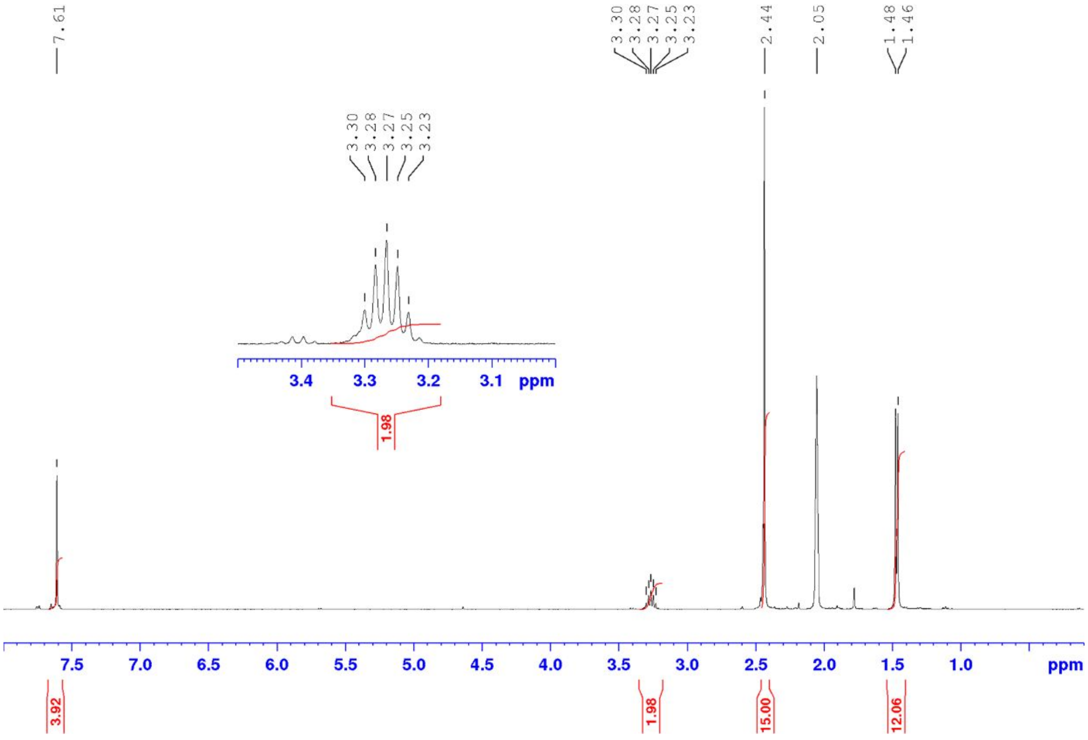

^{13}C{^{1}H} NMR [100 MHz, $(CD_3)_2CO$]

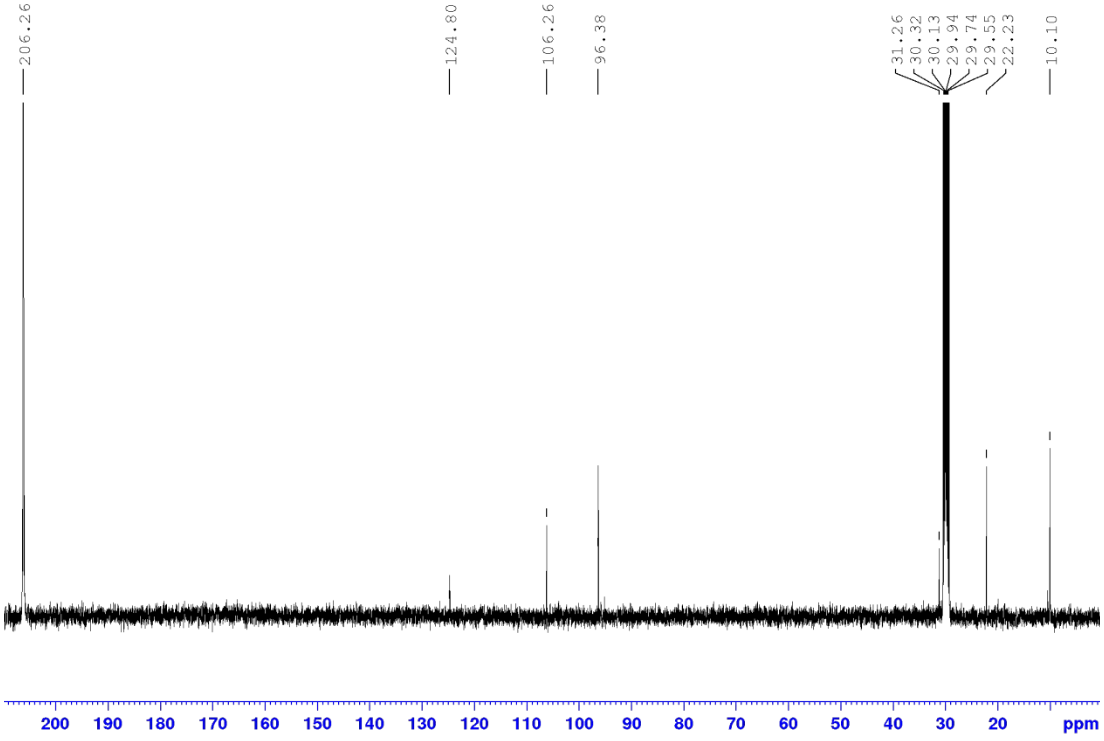

[Cp*Ir(η^6-1,4-diisopropylbenzene-d_2)][BF_4]$_2$

^{1}H NMR [500 MHz, $(CD_3)_2CO$]

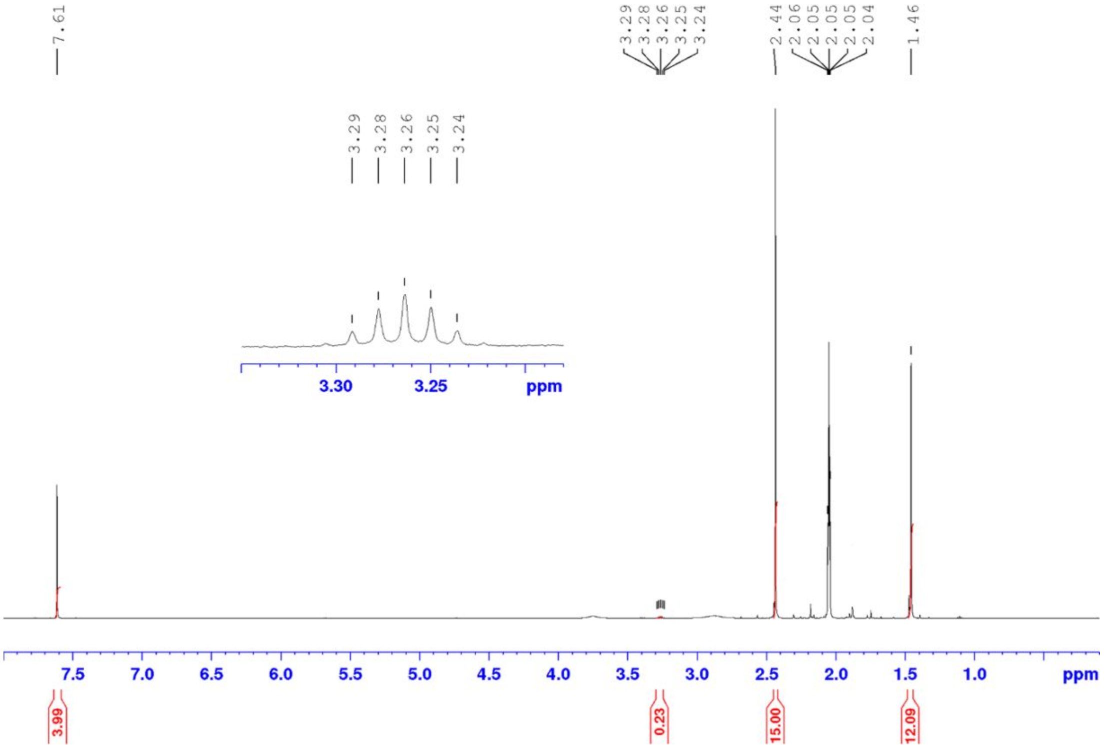

^{13}C{^{1}H} NMR [126 MHz, $(CD_3)_2CO$]

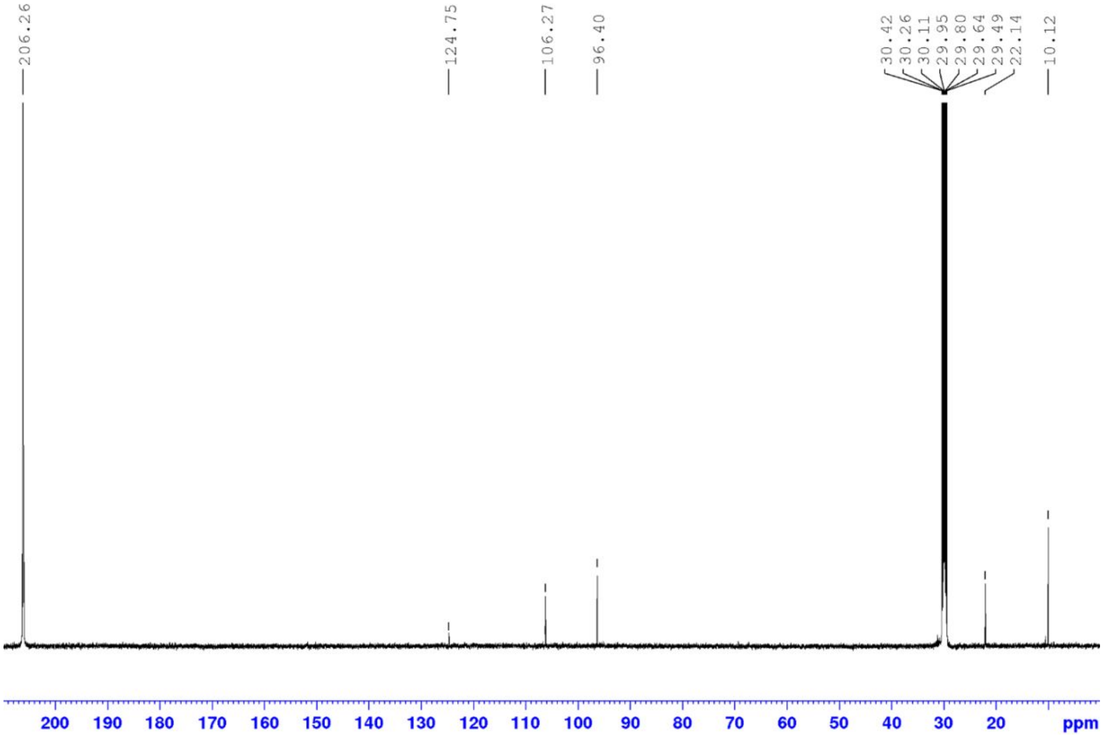

HSQC [500 MHz, $(CD_3)_2CO$]

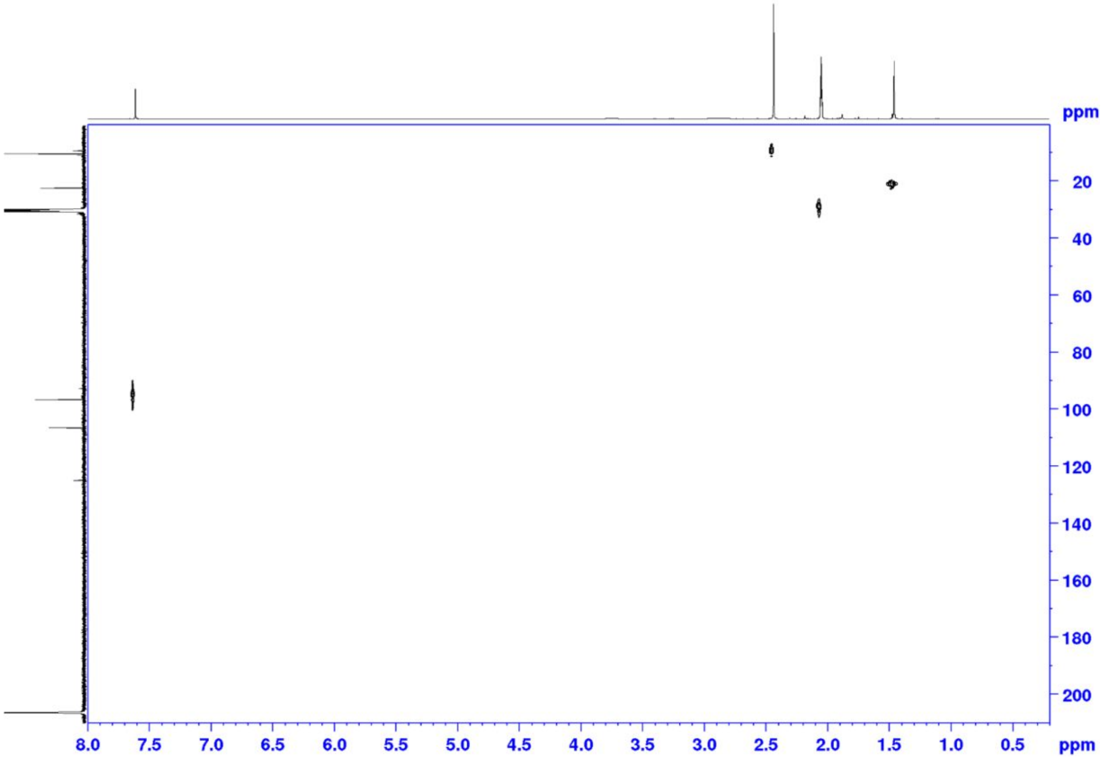

[Cp*Ir(η^6-1,4-diisopropylbenzene-d$_4$)][BF$_4$]$_2$

^{1}H NMR [500 MHz, $(CD_3)_2CO$]

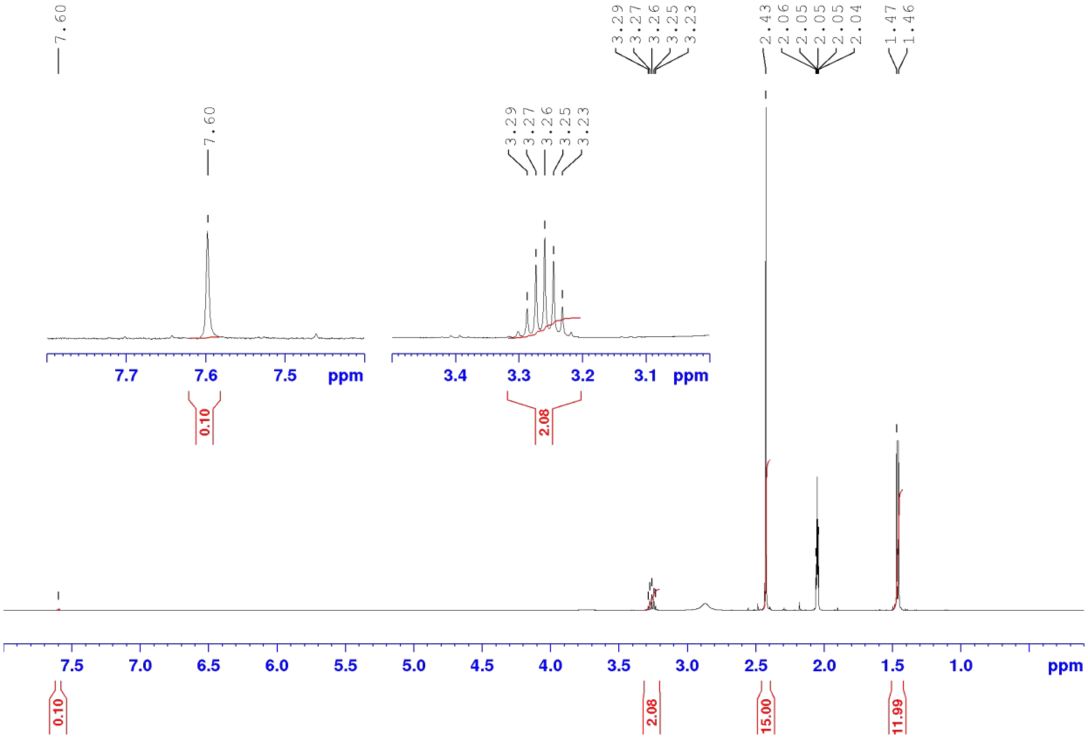

^{13}C{^{1}H} NMR [126 MHz, $(CD_3)_2CO$]

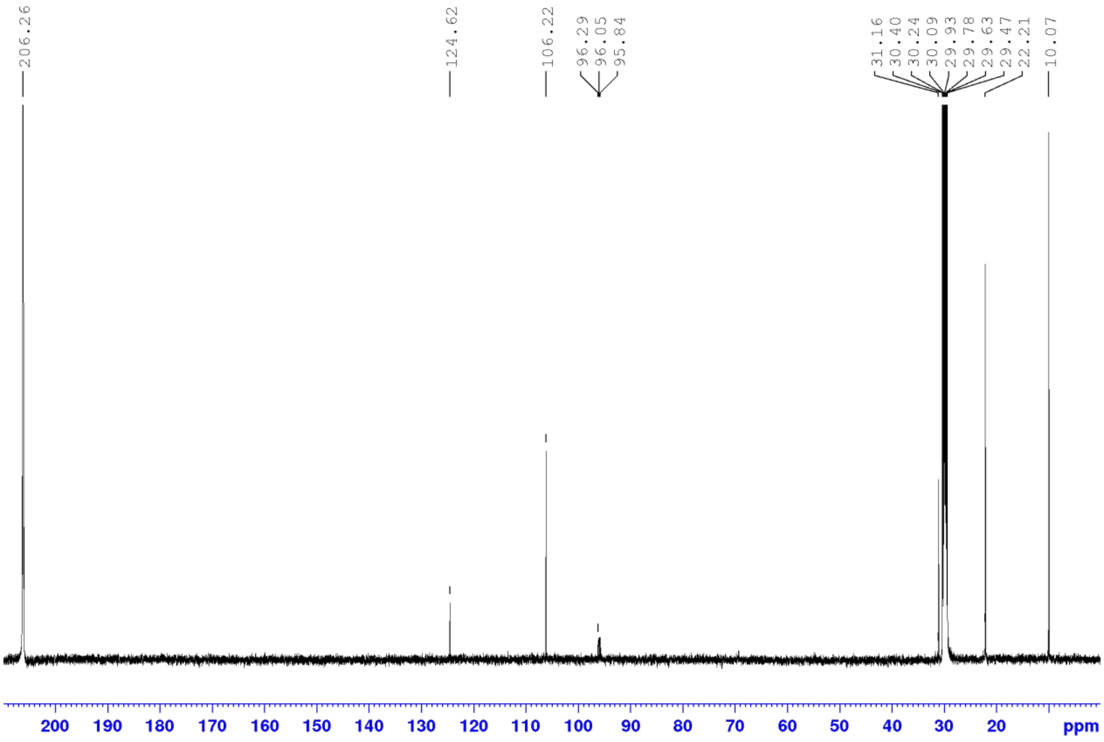

HSQC [500 MHz, $(CD_3)_2CO$]

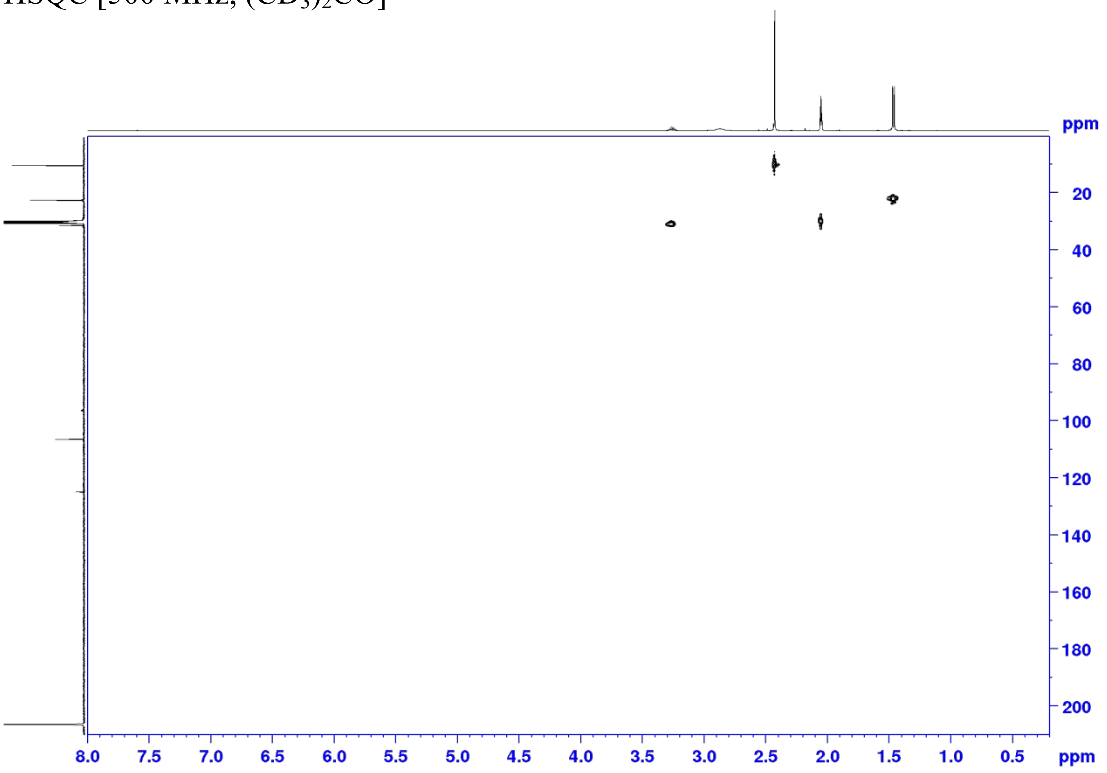

[Cp*Ir(η^6-4-isopropyltoluene)][BF_4]$_2$

^{1}H NMR [400 MHz, $(CD_3)_2CO$ + 20 μL TFA]

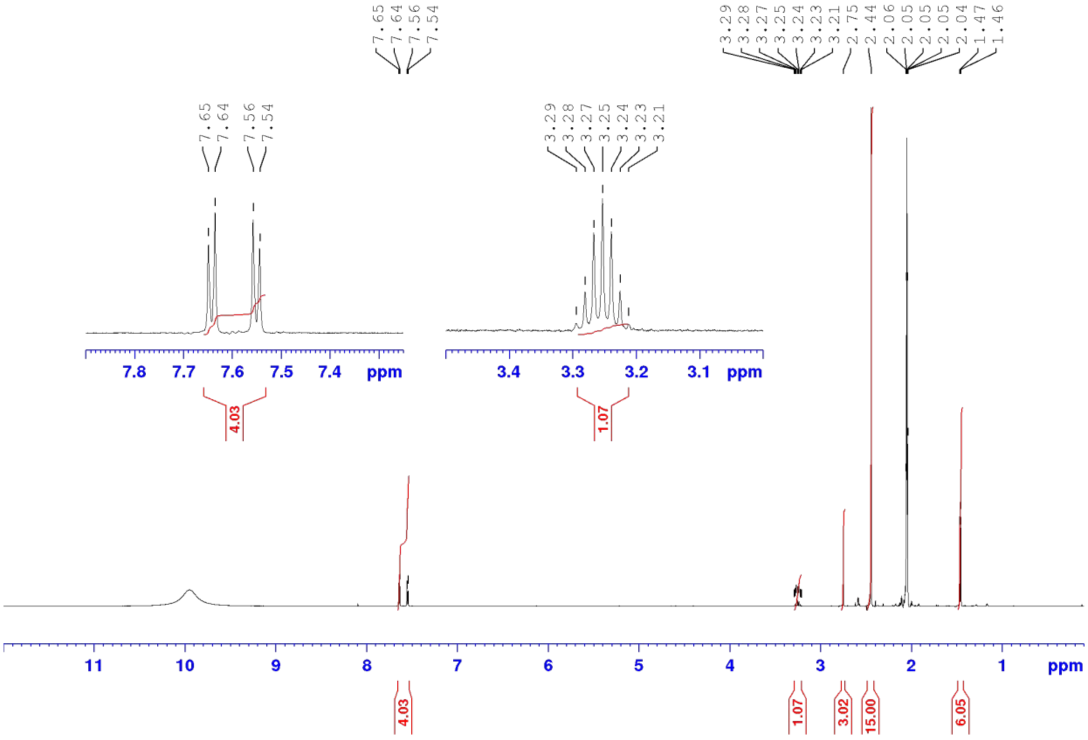

^{13}C{^{1}H} NMR [100 MHz, $(CD_3)_2CO$ + 20 μL TFA]

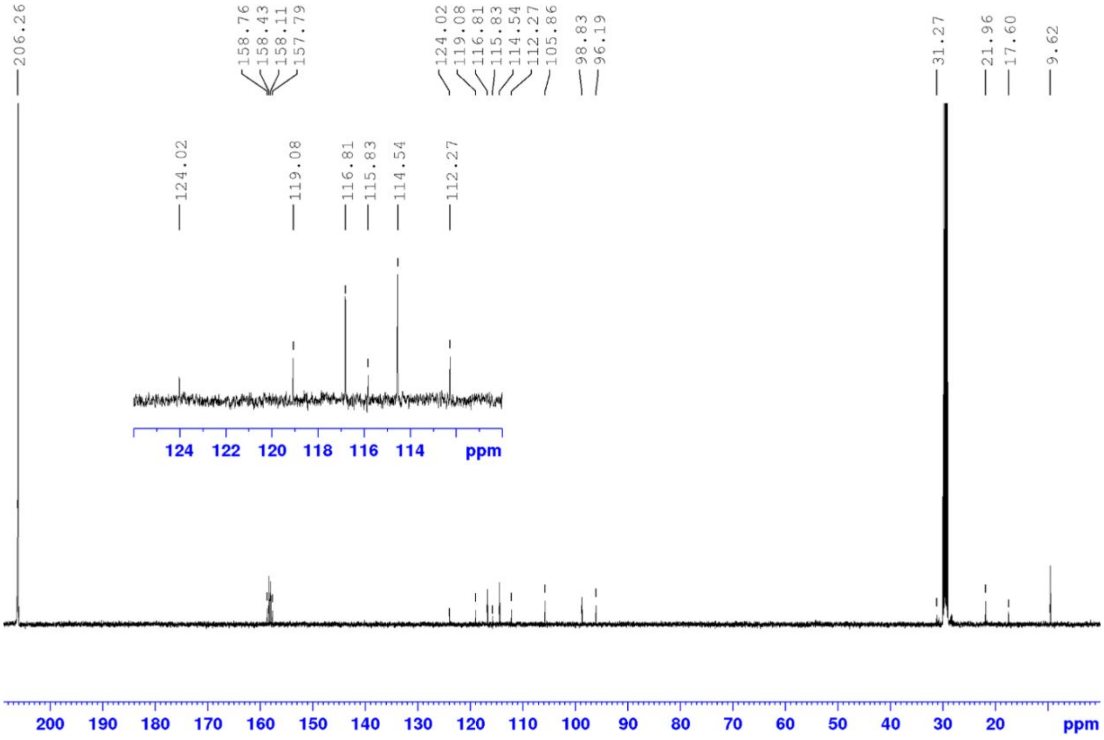

Complex **1f** - [Cp*Ir(η^4-ethylbenzene)]

^{1}H NMR (400 MHz, C_6D_6)

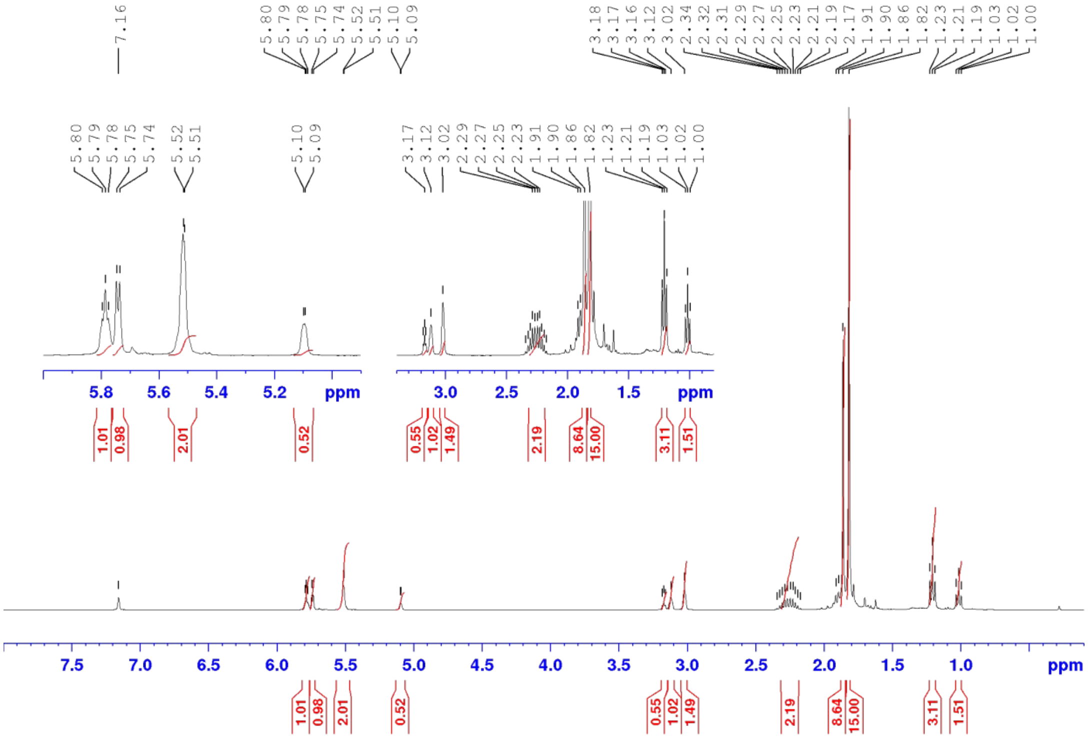

^{13}C{^{1}H} NMR (100 MHz, C_6D_6)

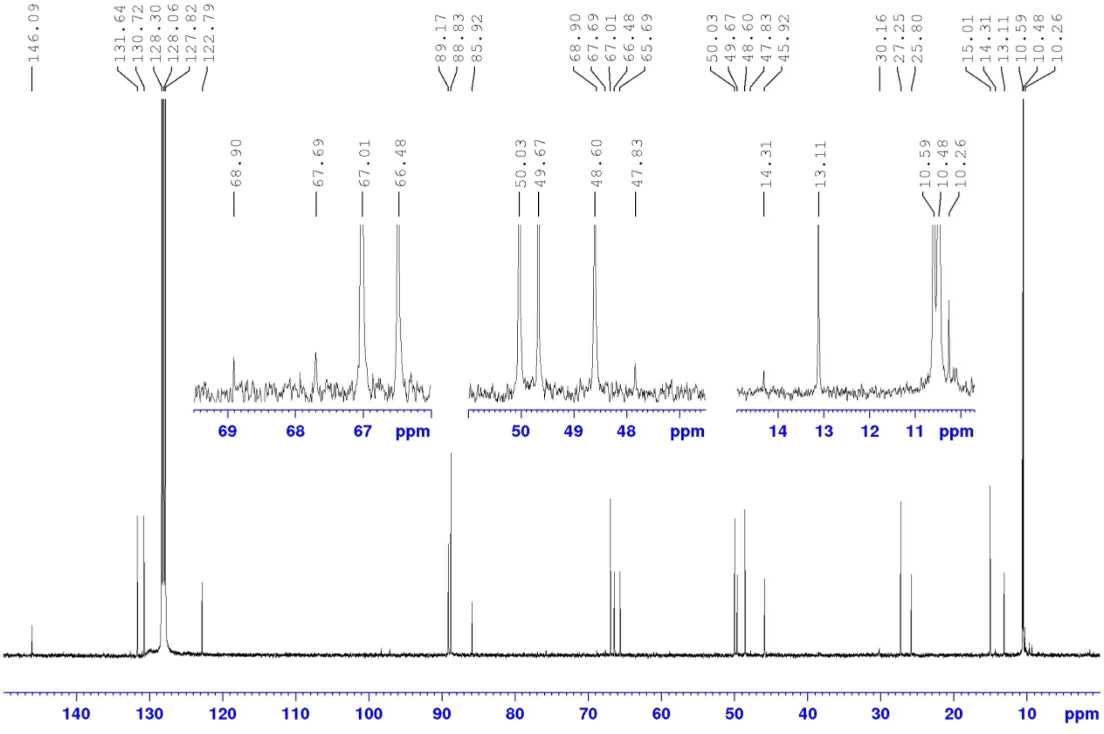

Compound **1a** - [Cp*Ir(η^4-isopropylbenzene)]

^{1}H NMR (400 MHz, C_6D_6)

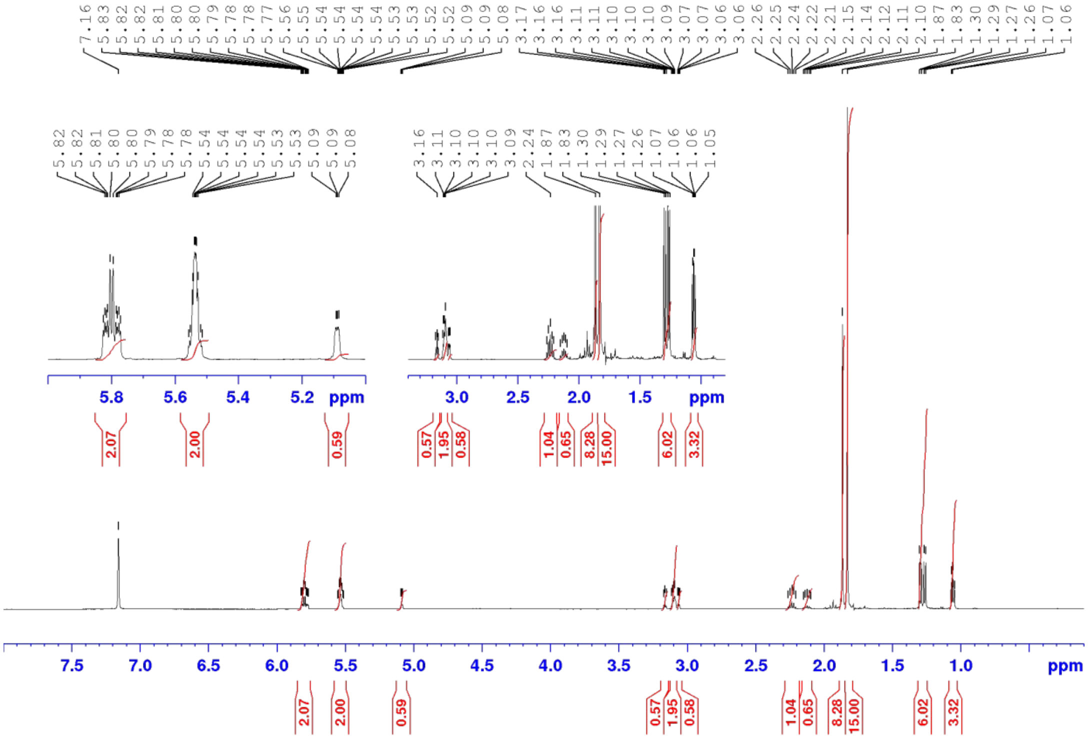

$^{13}C\{^1H\}$ NMR (100 MHz, C_6D_6)

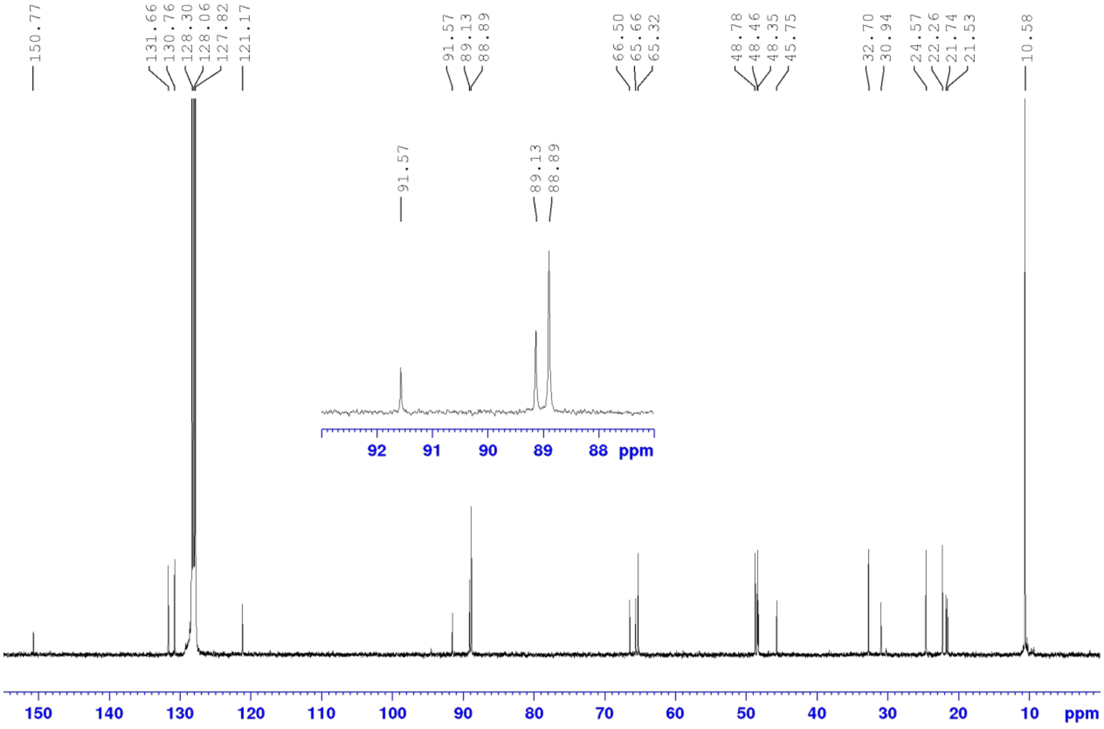

Complex **1b** - [Cp*Ir(η^4-*sec*-butylbenzene)]

^{1}H NMR (500 MHz, C_6D_6)

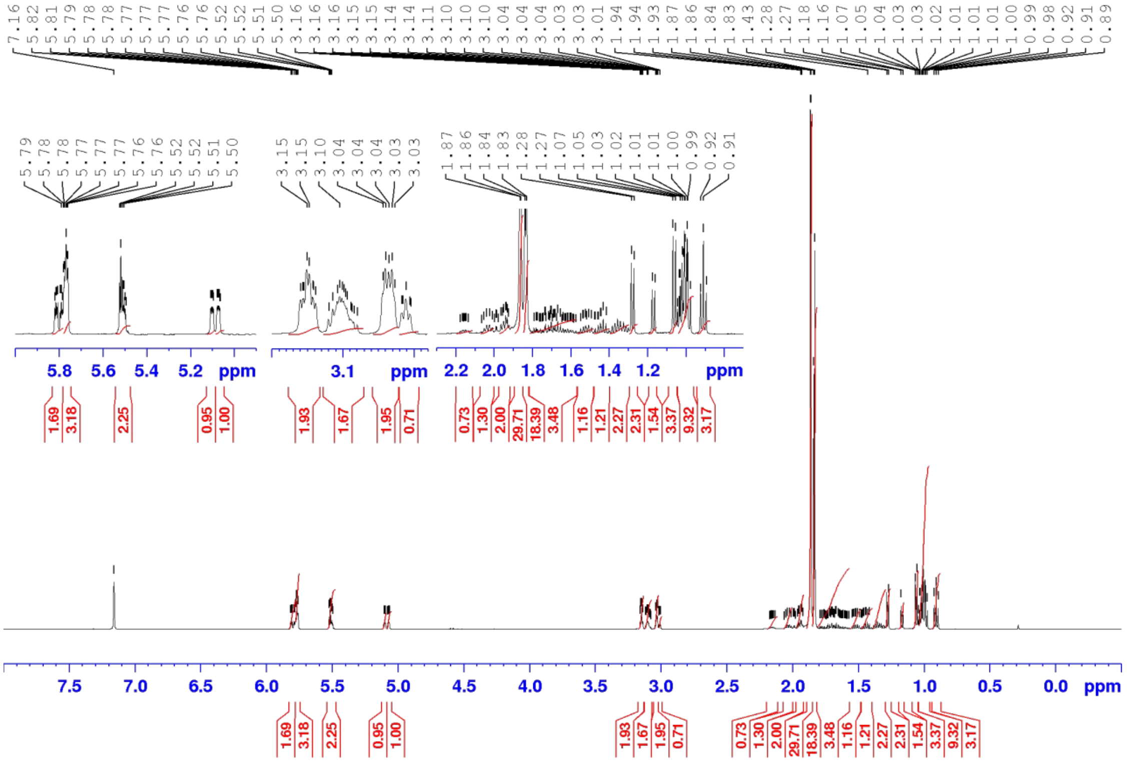

^{13}C{^{1}H} NMR (126 MHz, C_6D_6)

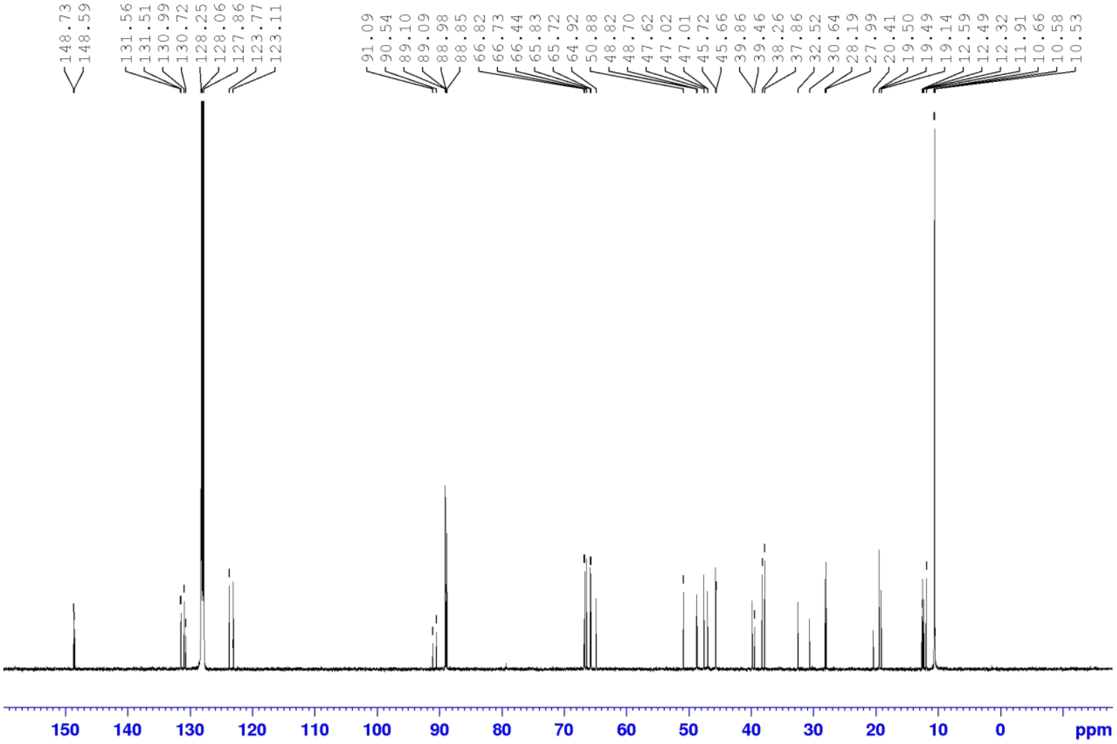

Complex **1c** - [Cp*Ir(η^4-3-pentylbenzene)]

^{1}H NMR (500 MHz, C_6D_6)

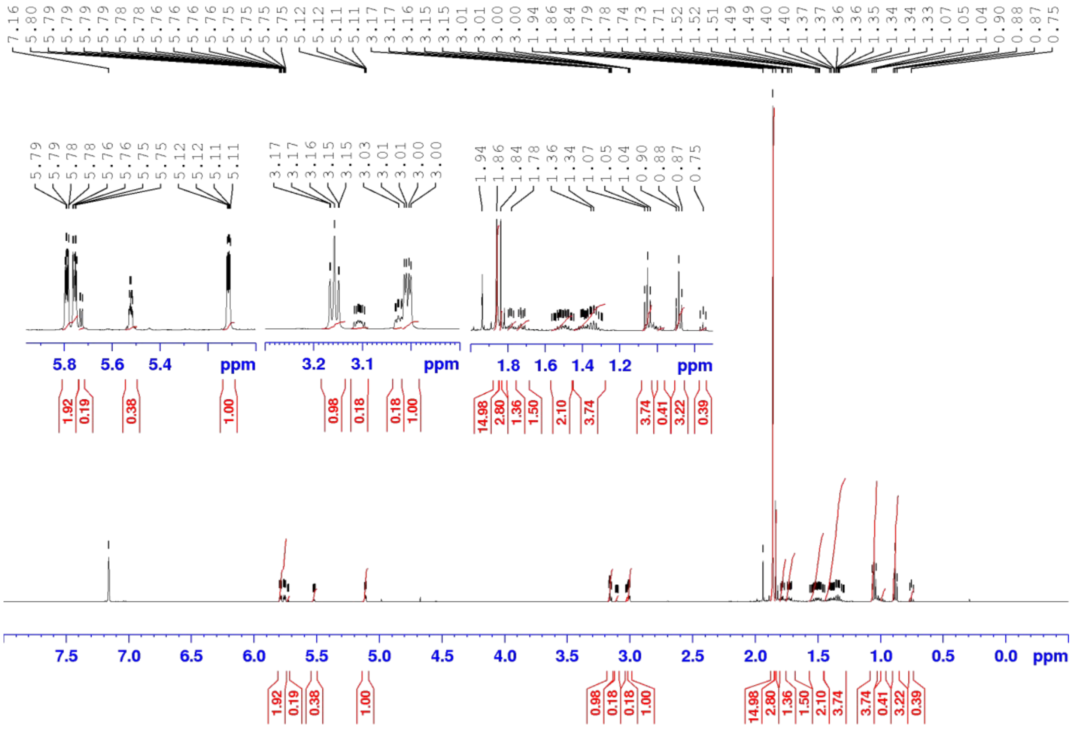

^{13}C{^{1}H} NMR (126 MHz, C_6D_6)

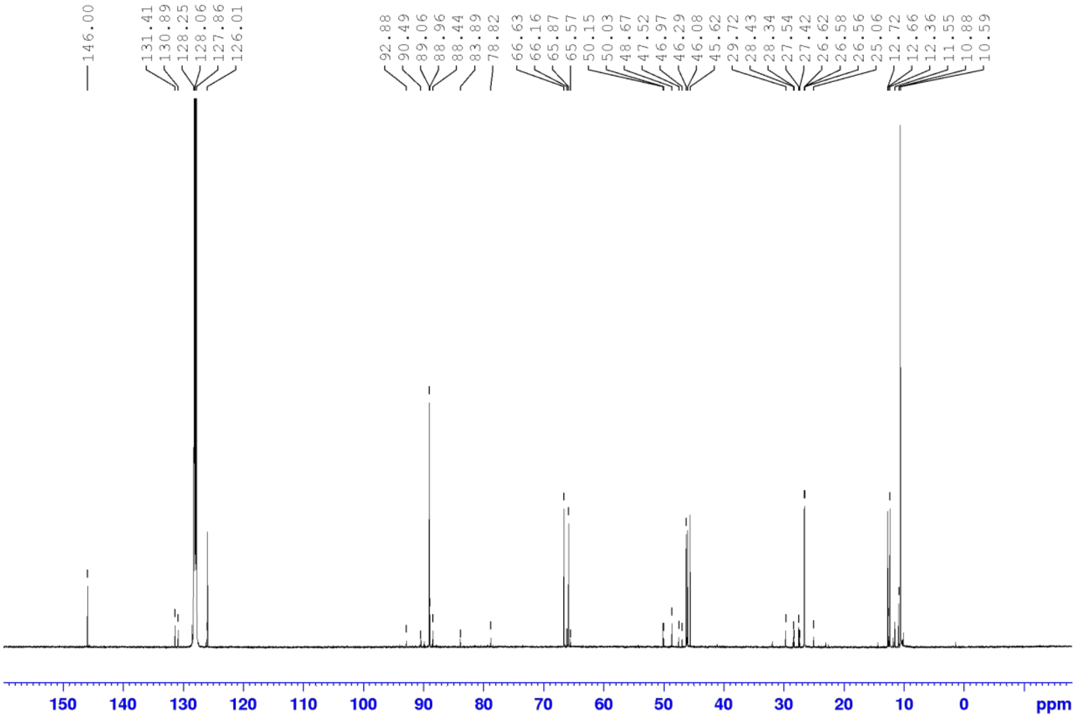

Complex **1d** - [Cp*Ir(η^4-cyclopentylbenzene)]

^{1}H NMR (400 MHz, C_6D_6)

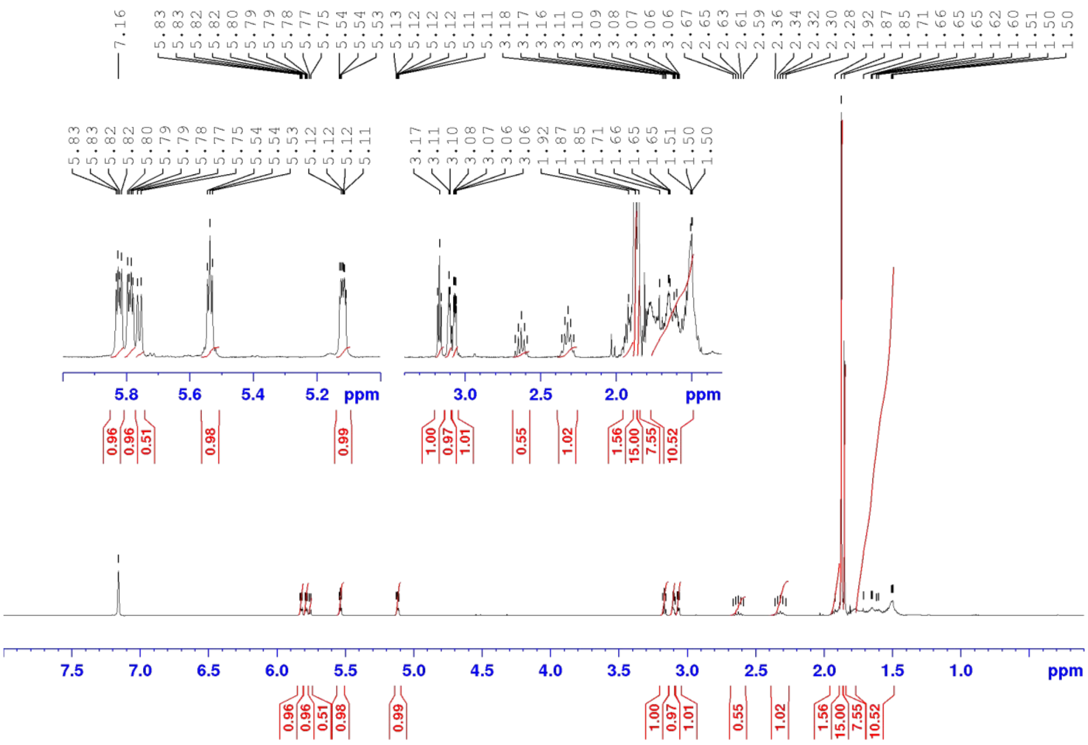

^{13}C{^{1}H} NMR (100 MHz, C_6D_6)

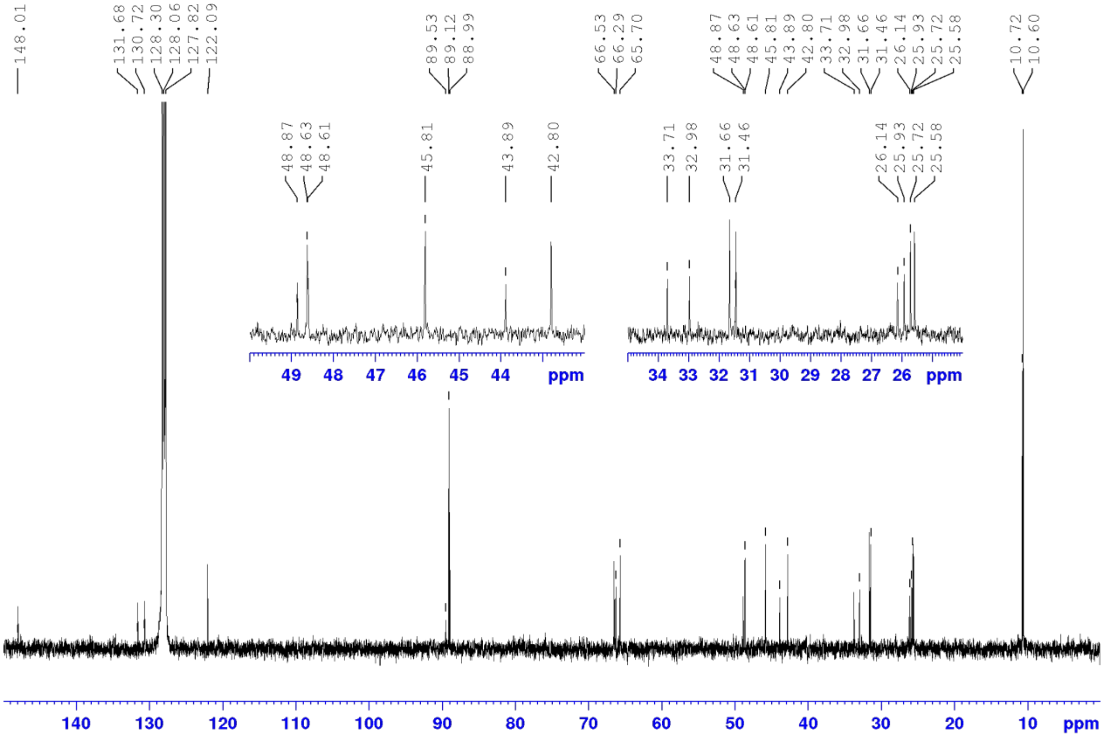

Complex **1e** - [Cp*Ir(η^4-cyclohexylbenzene)]

^{1}H NMR (400 MHz, C_6D_6)

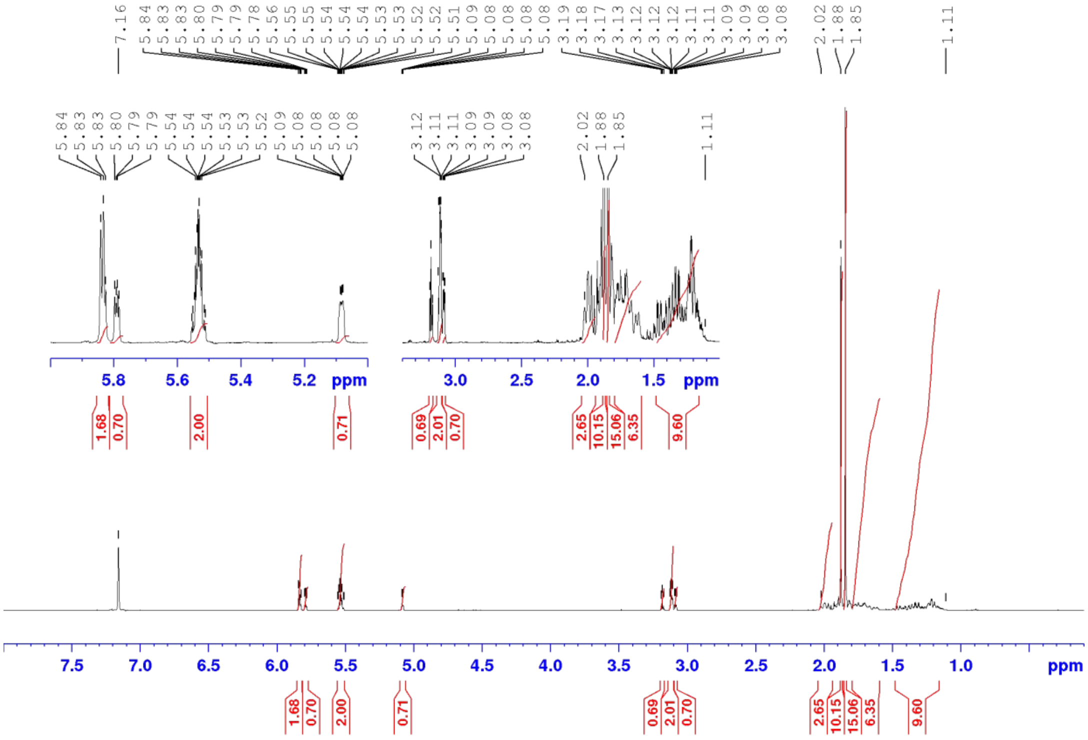

^{13}C{^{1}H} NMR (100 MHz, C_6D_6)

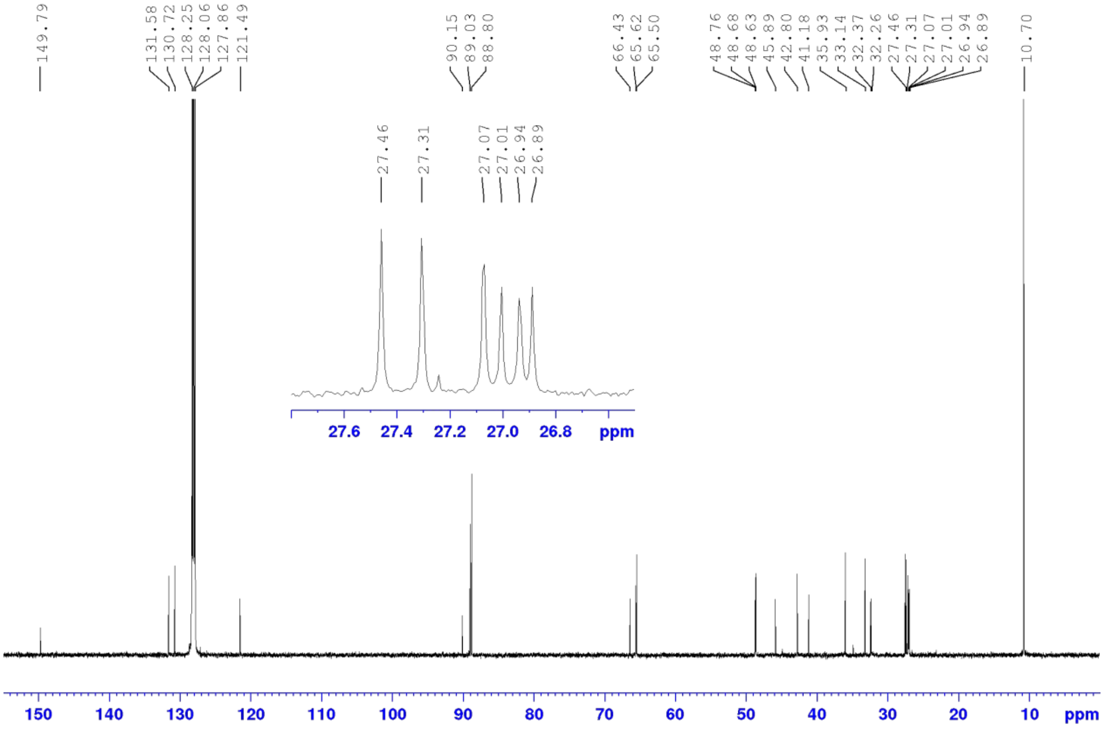

Complex **1g**- [Cp*Ir(η^4-*n*-propylbenzene)]

^{1}H NMR (500 MHz, C_6D_6)

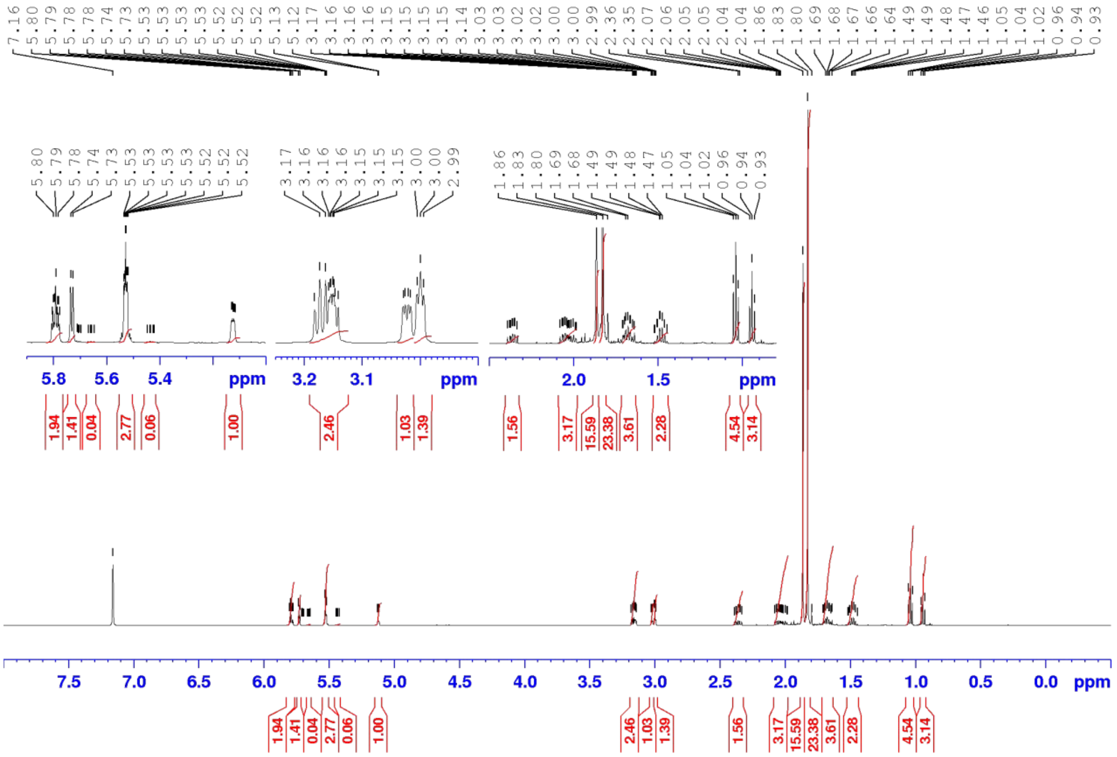

^{13}C{^{1}H} NMR (126 MHz, C_6D_6)

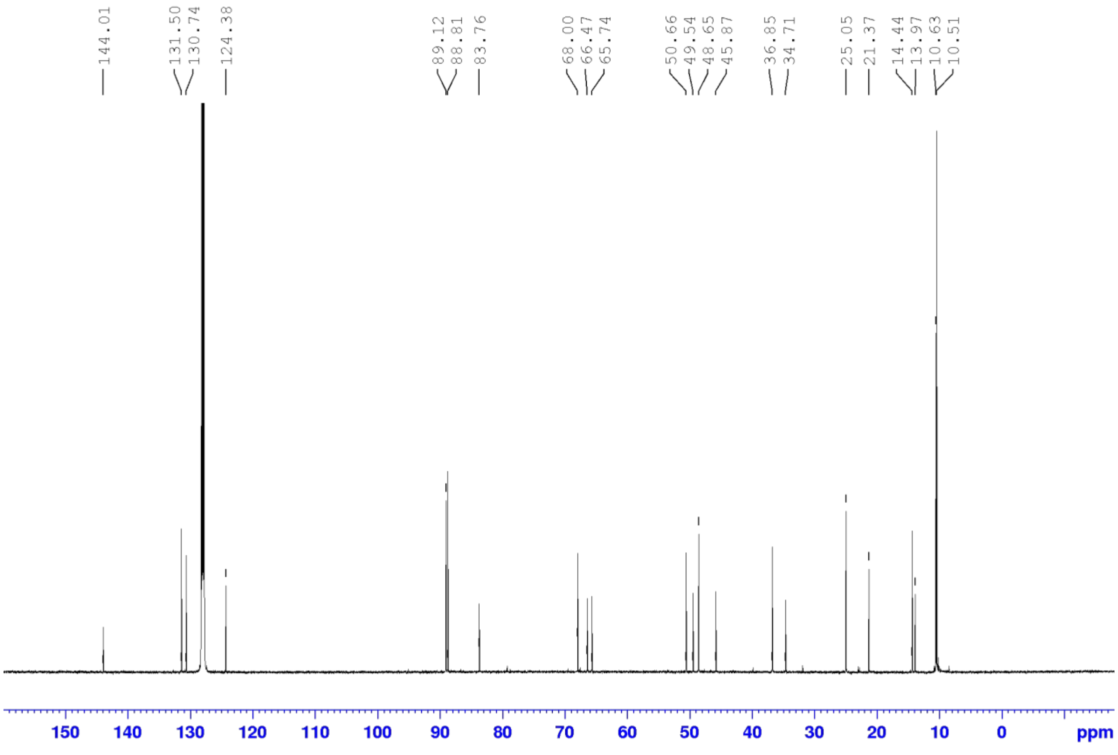

Complex **1h**- [Cp*Ir(η^4-*n*-butylbenzene)]

^{1}H NMR (500 MHz, C_6D_6)

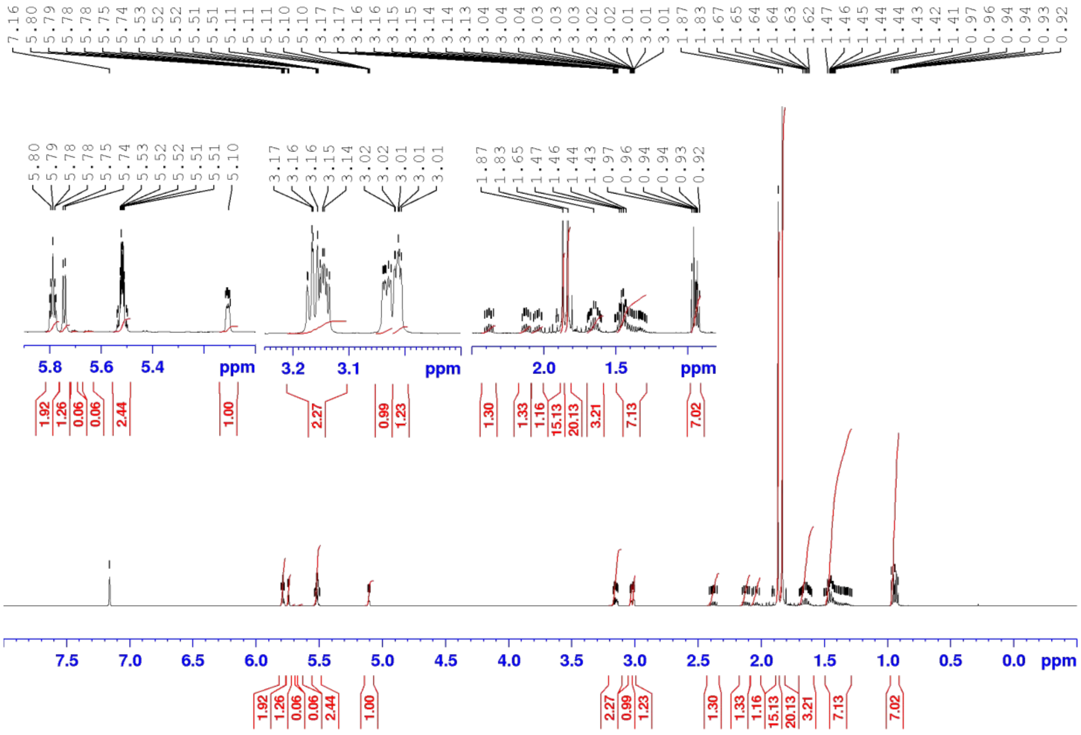

^{13}C{^{1}H} NMR (126 MHz, C_6D_6)

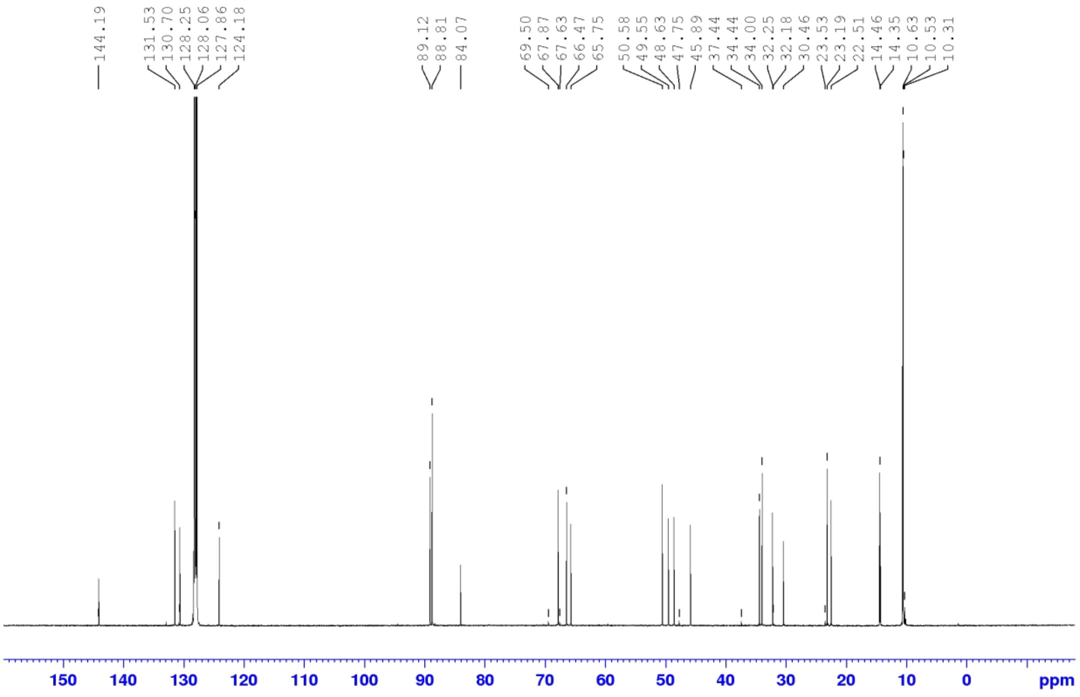

Complex **1i**- [Cp*Ir(η^4-*iso*-butylbenzene)]

^{1}H NMR (500 MHz, C_6D_6)

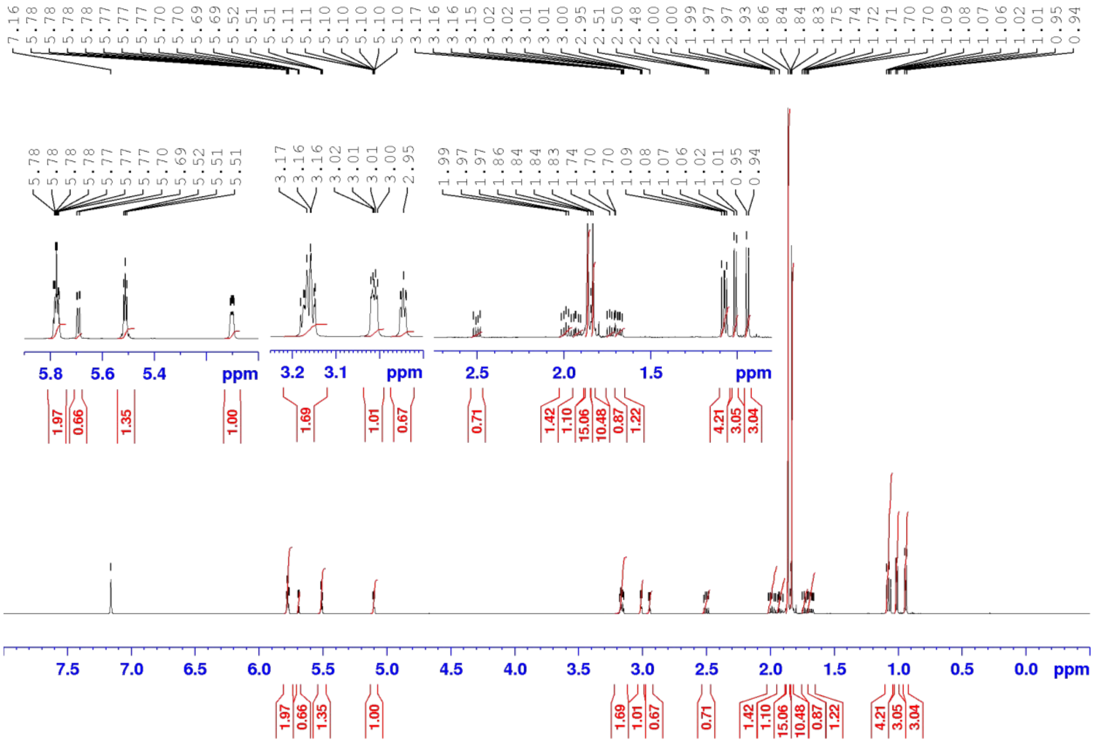

^{13}C{^{1}H} NMR (126 MHz, C_6D_6)

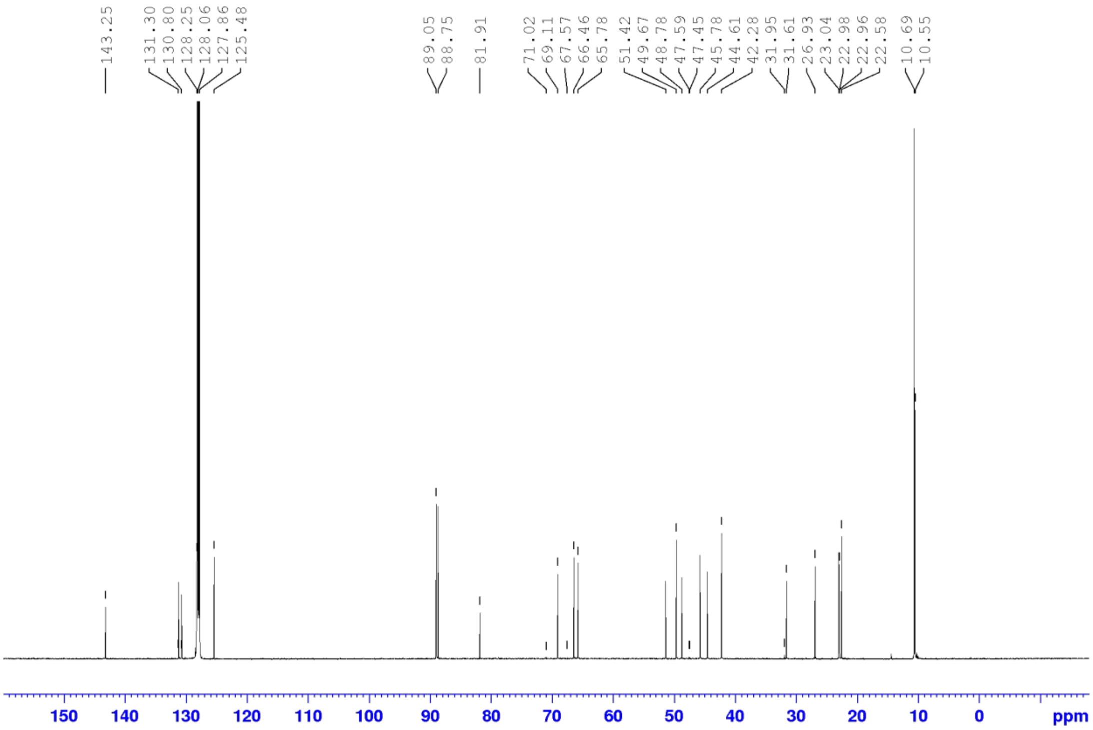

Complex **1j**- [Cp*Ir(η^4-neopentylbenzene)]

^{1}H NMR (500 MHz, C_6D_6)

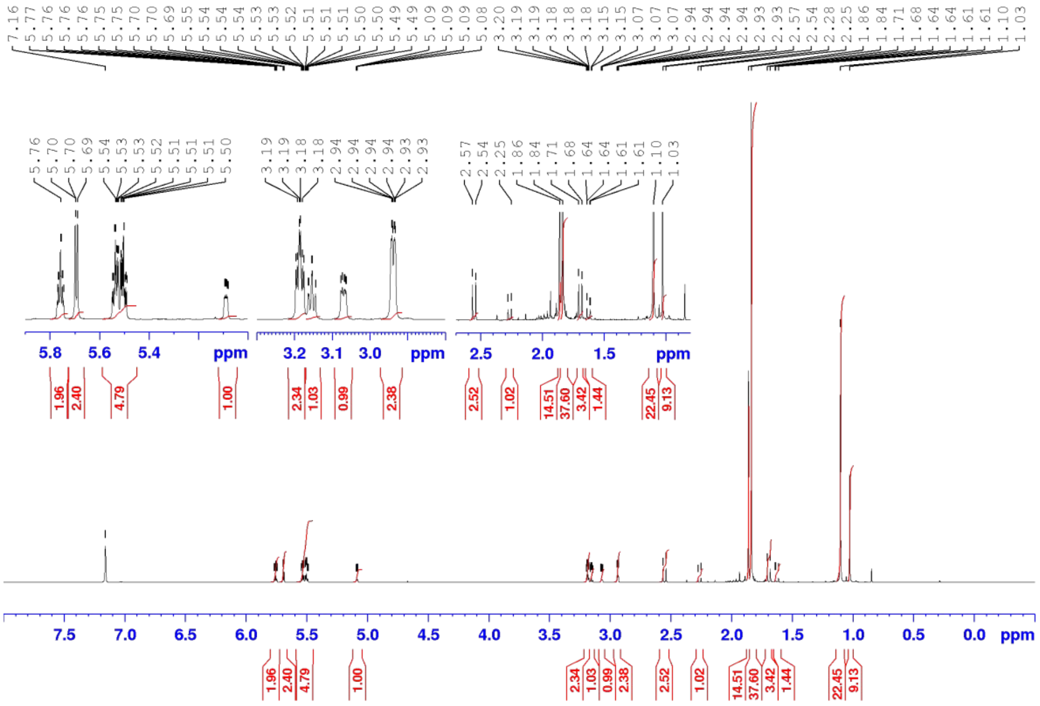

^{13}C{^{1}H} NMR (126 MHz, C_6D_6)

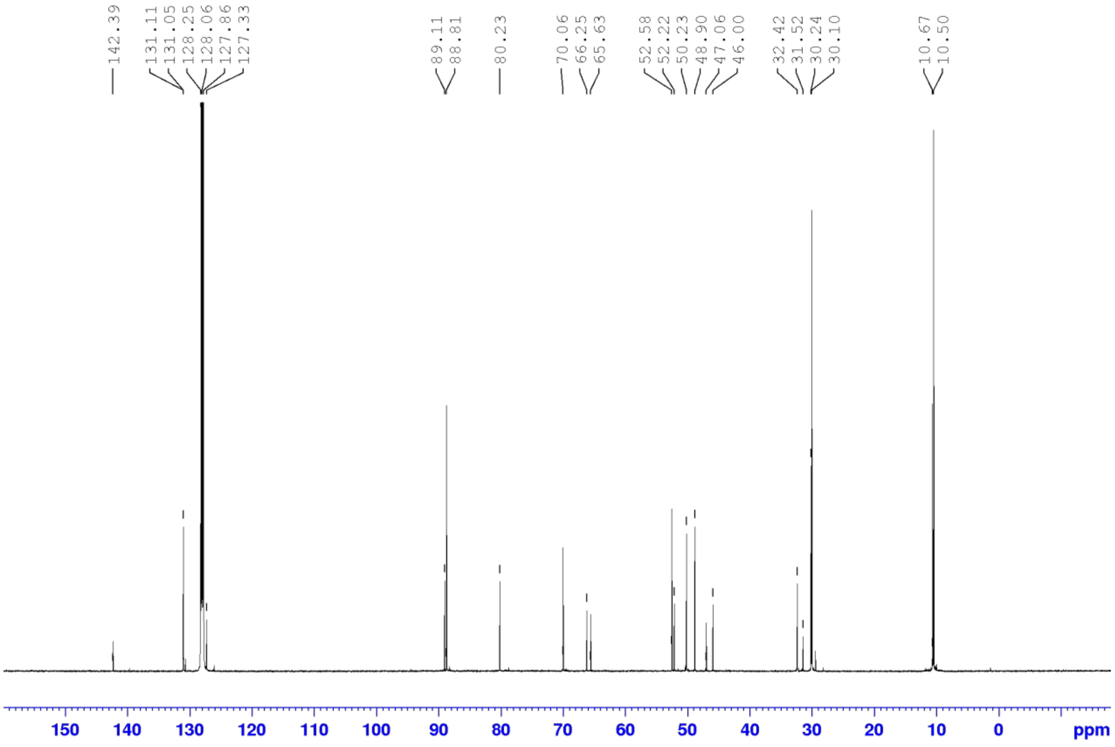

Complex **1l** - [Cp*Ir(η^4-1,4-diisopropylbenzene)]

^{1}H NMR (400 MHz, C_6D_6)

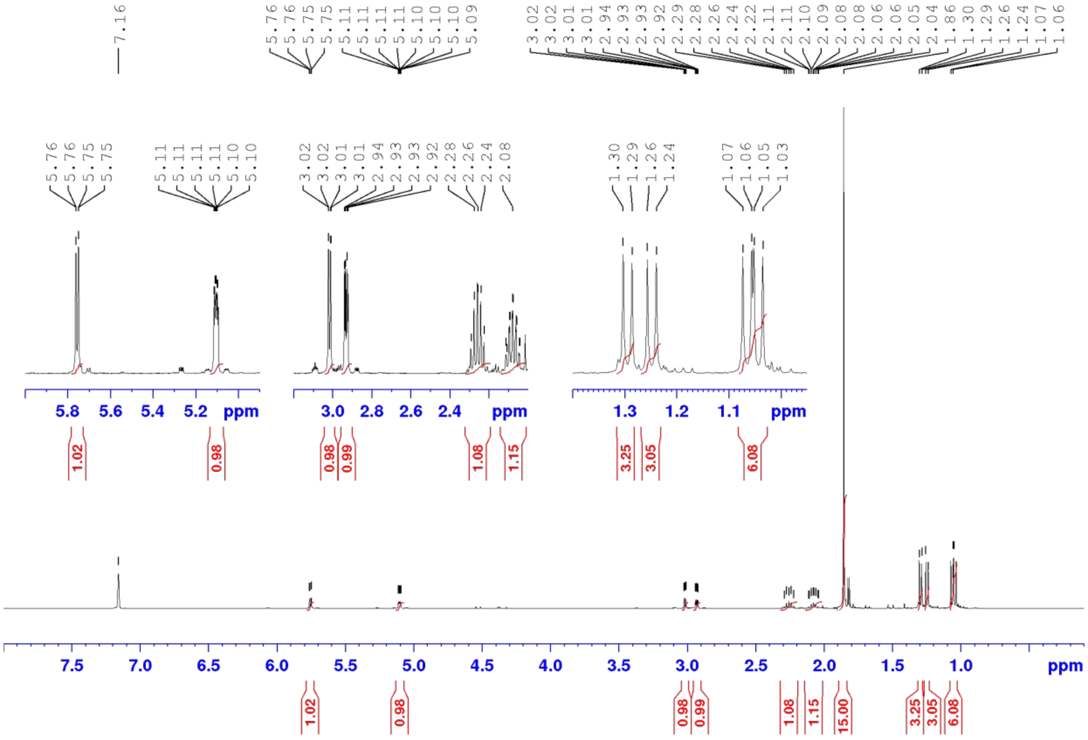

^{13}C{^{1}H} NMR (100 MHz, C_6D_6)

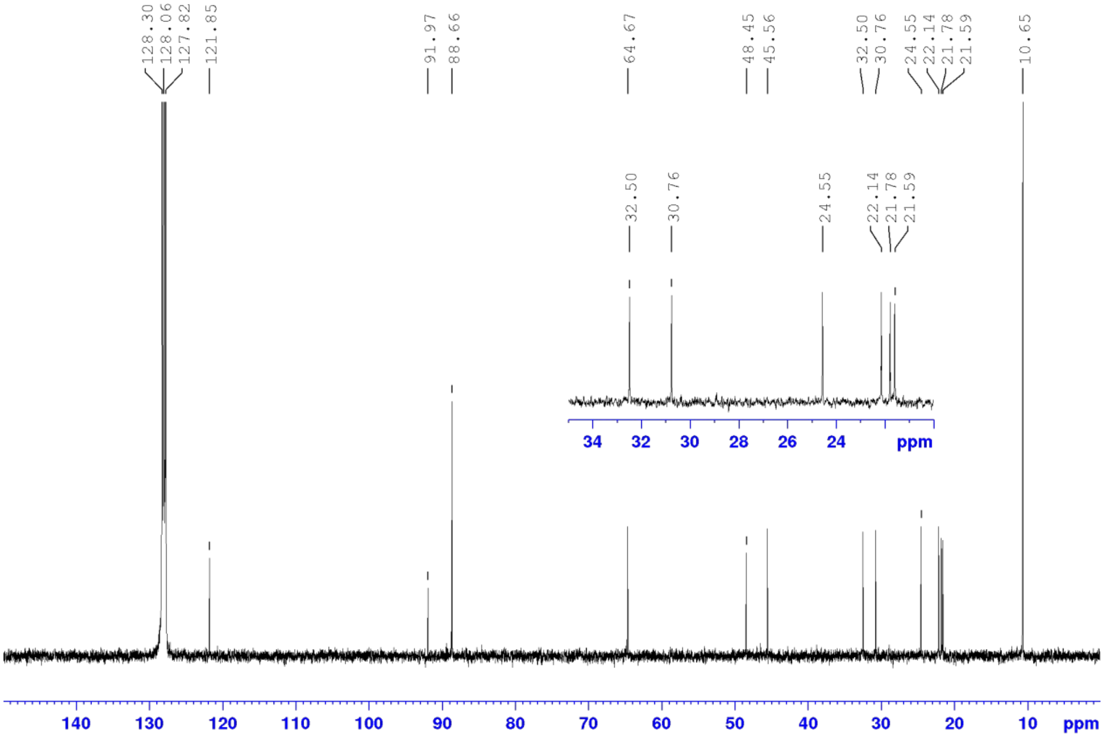

Complex **1l-d₂** - [Cp*Ir(η^4-1,4-diisopropylbenzene-d_2)]

^{1}H NMR (500 MHz, C_6D_6)

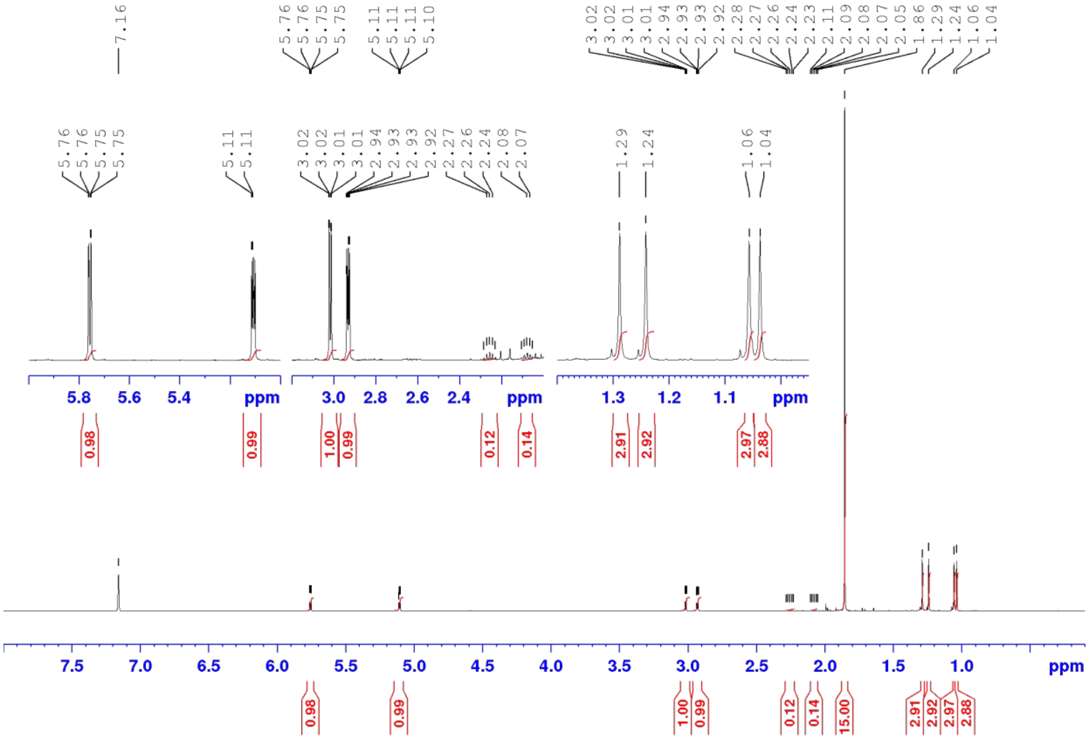

^{13}C{^{1}H} NMR (126 MHz, C_6D_6)

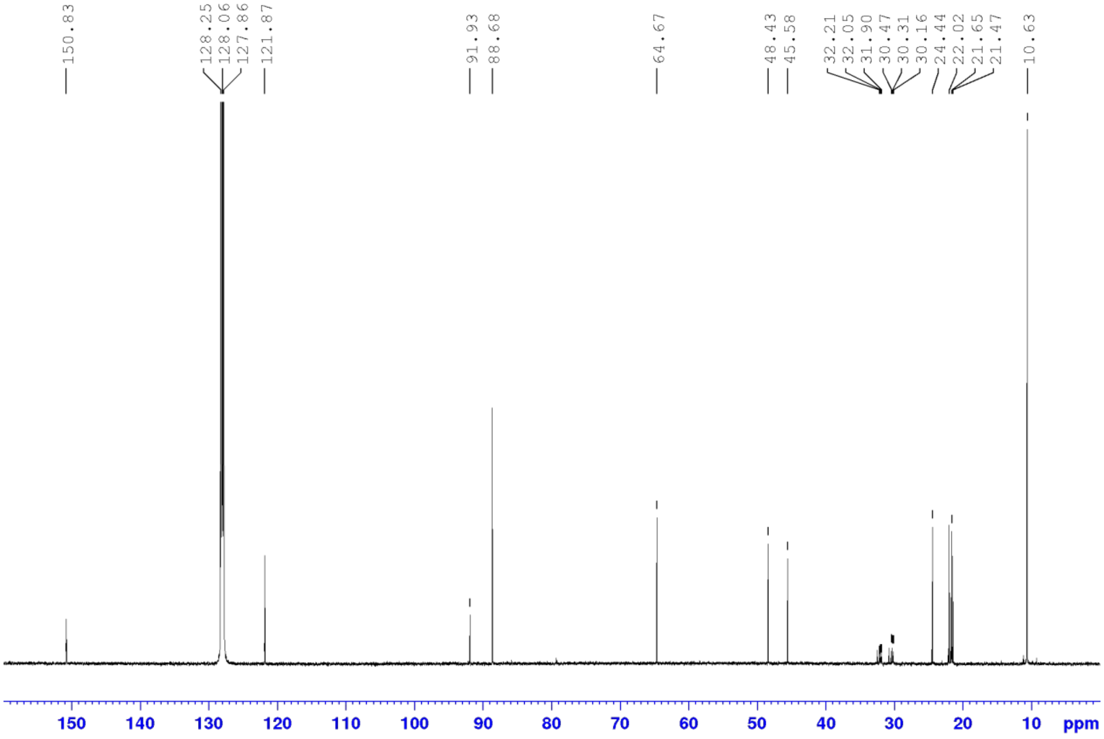

HSQC (500 MHz, C_6D_6)

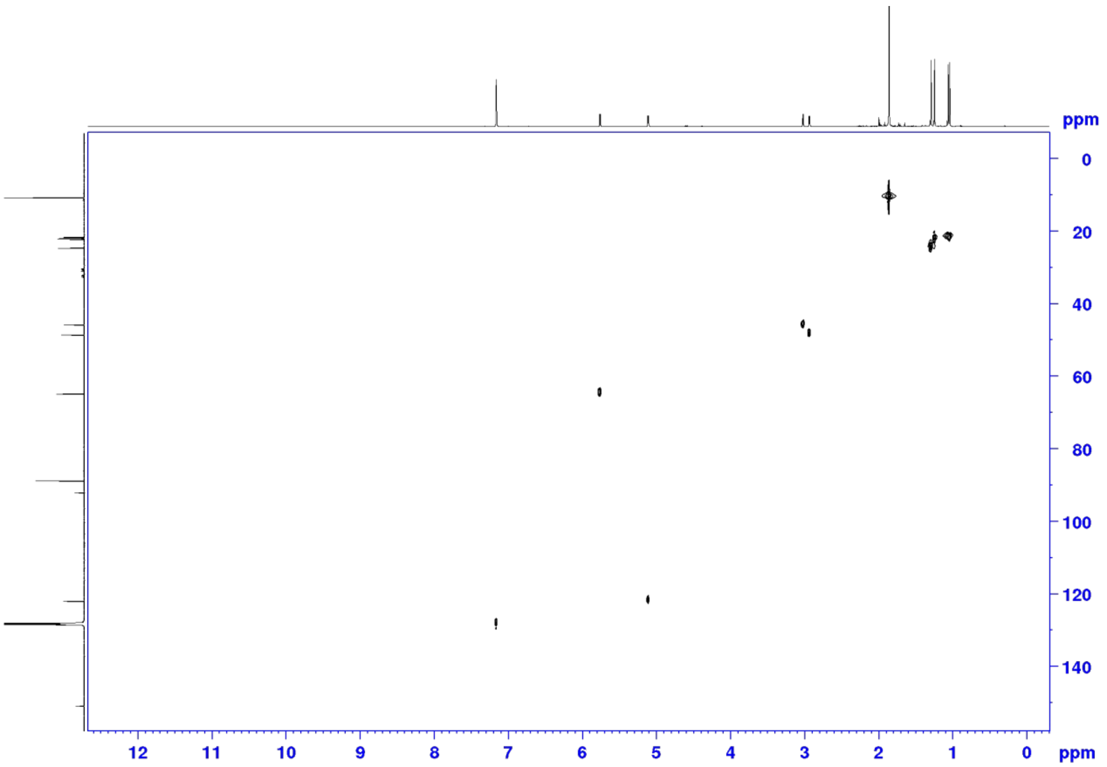

Complex **1l-d$_4$** - [Cp*Ir(η^4-1,4-diisopropylbenzene-d$_4$)]

^{1}H NMR (500 MHz, C_6D_6)

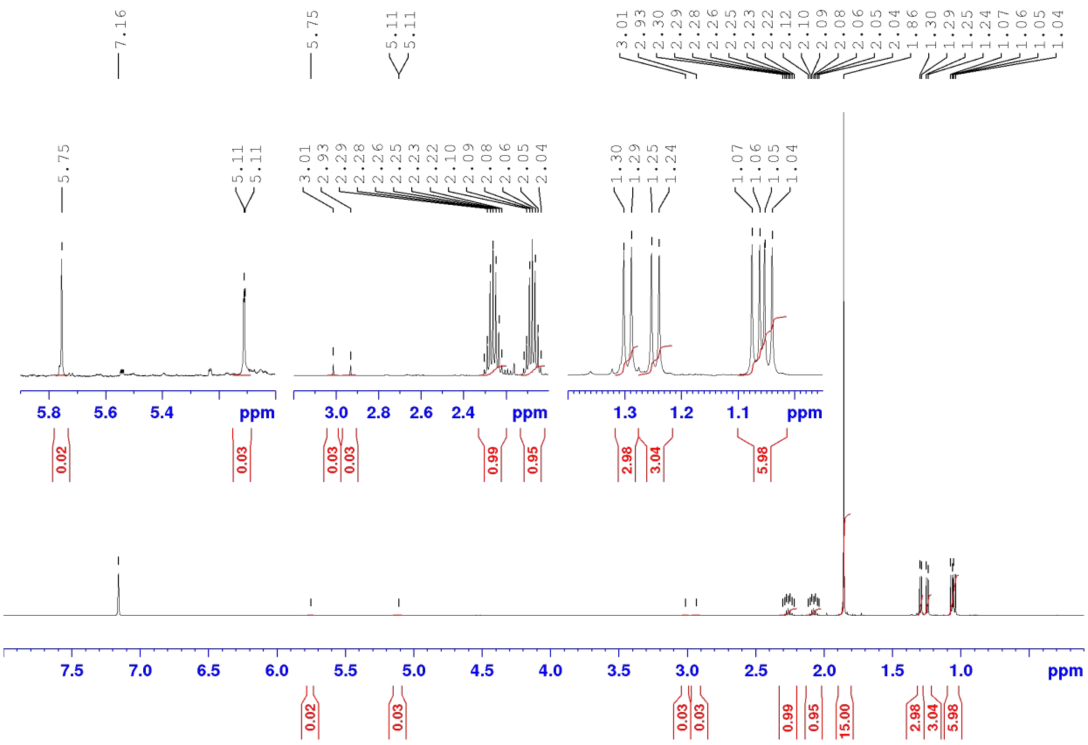

$^{13}C\{^1H\}$ NMR (126 MHz, C_6D_6)

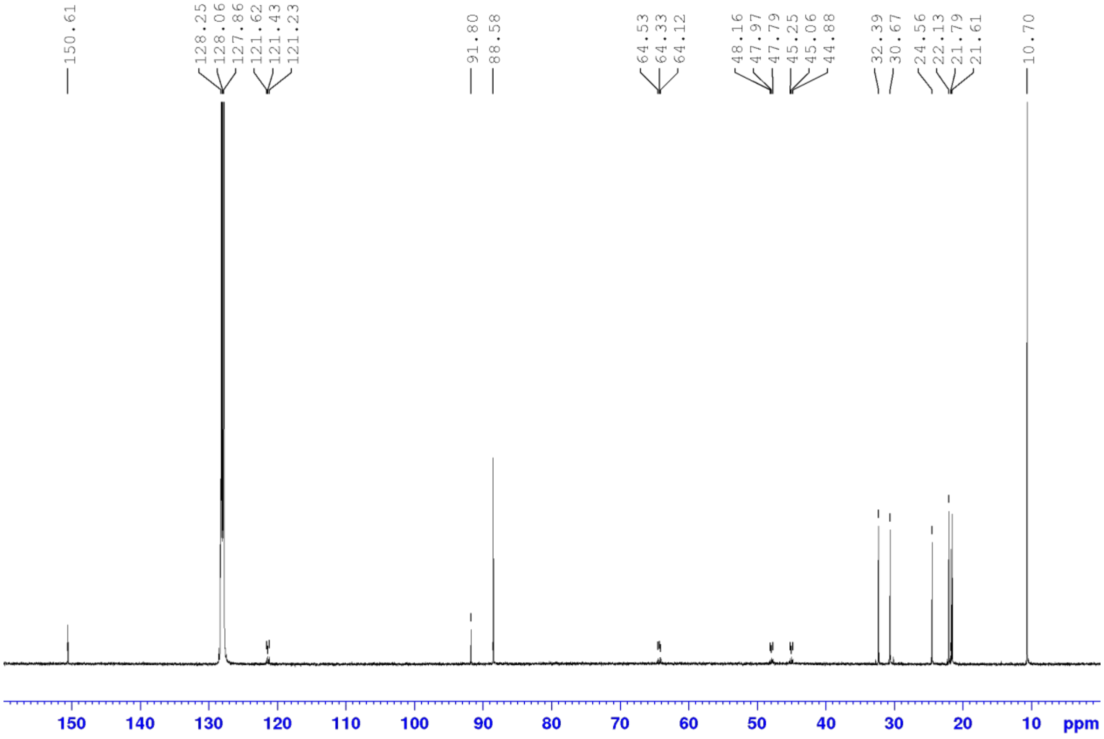

HSQC (500 MHz, C_6D_6)

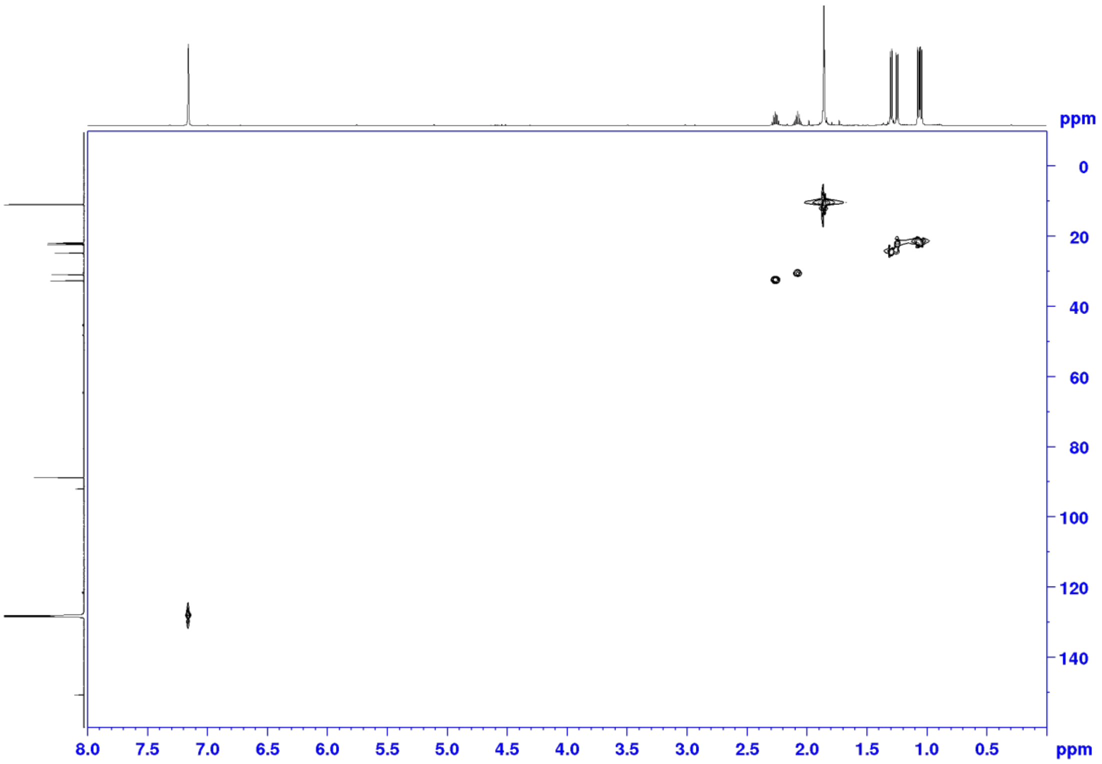

Complex **1m** - [Cp*Ir(η^4-4-isopropyltoluene)]

^{1}H NMR (500 MHz, C_6D_6)

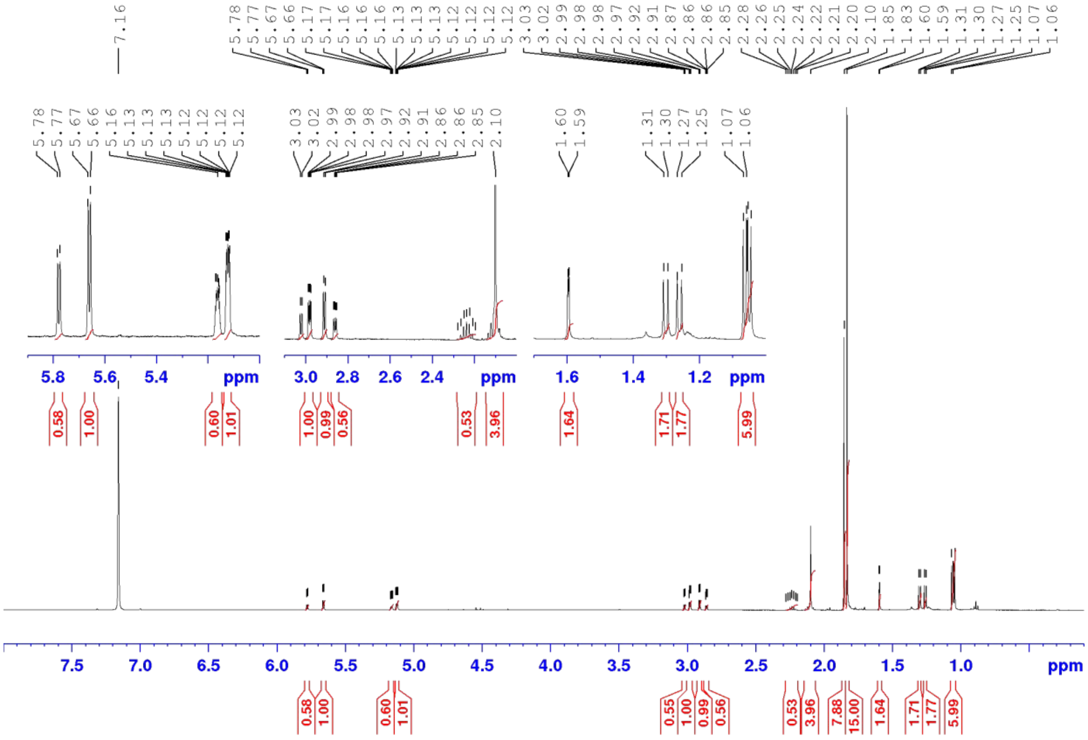

^{13}C{^{1}H} NMR (126 MHz, C_6D_6)

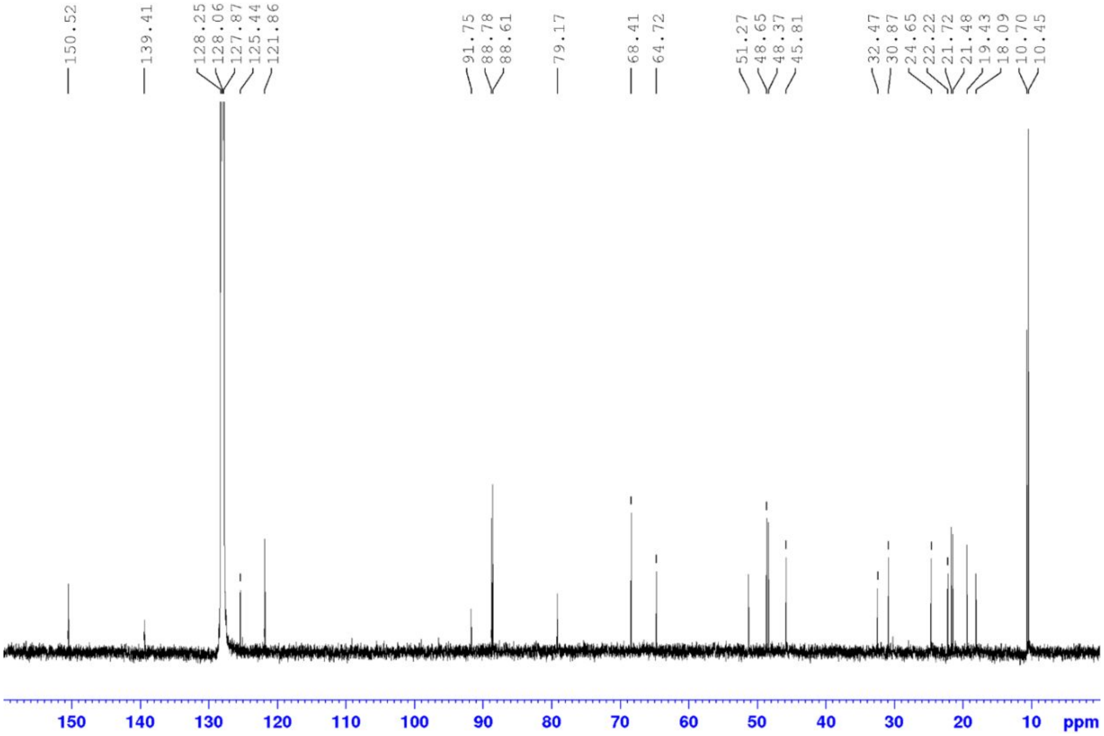

Compound **2f** - [Cp*Ir(PMe$_3$)(H)(2-ethylphenyl)]

^{1}H NMR (500 MHz, C_6D_6)

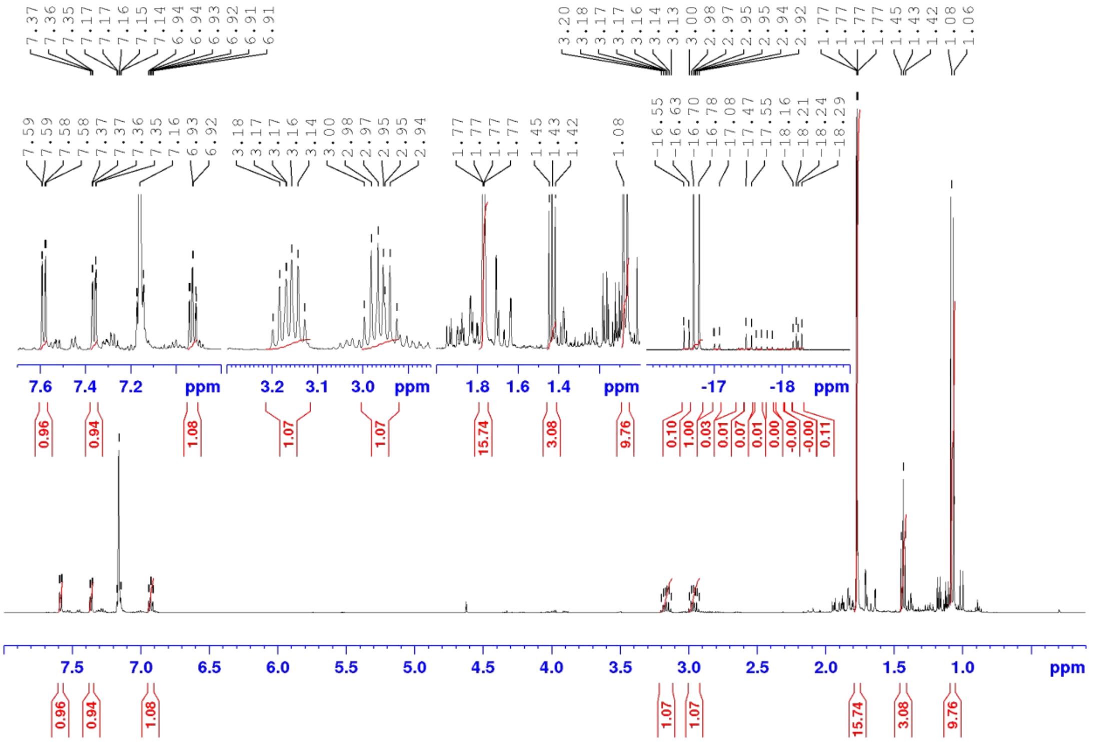

^{13}C{^{1}H} NMR (126 MHz, C_6D_6)

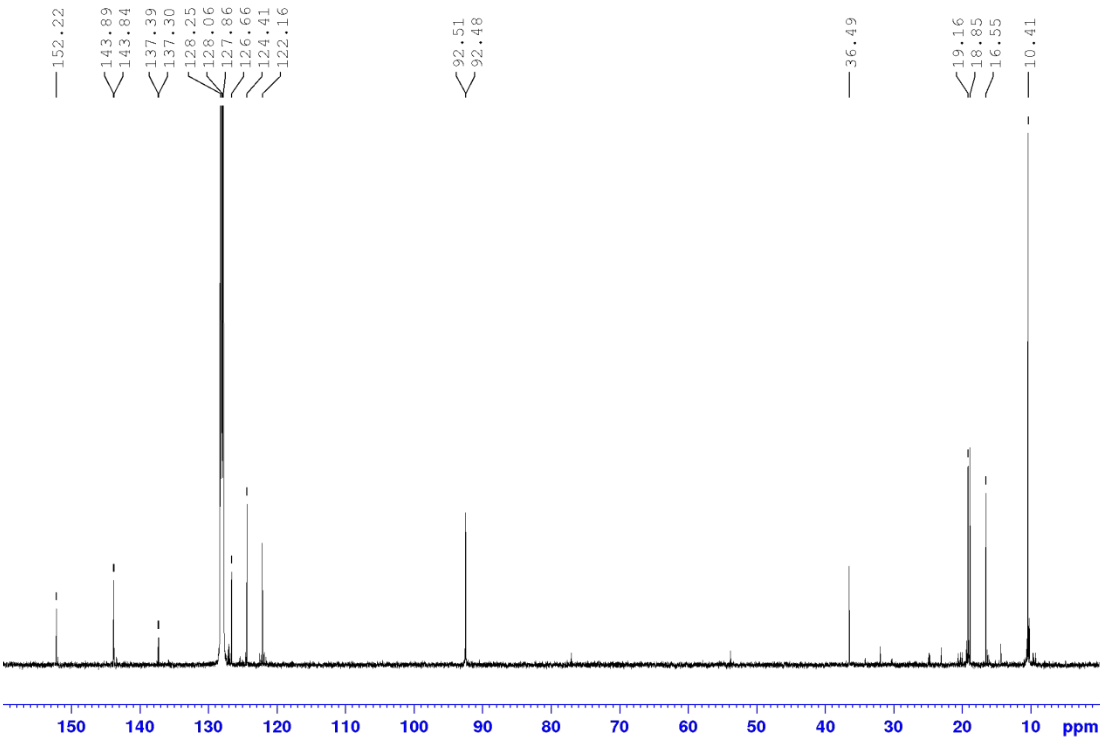

$^{31}P\{^1H\}$ NMR (202 MHz, C_6D_6)

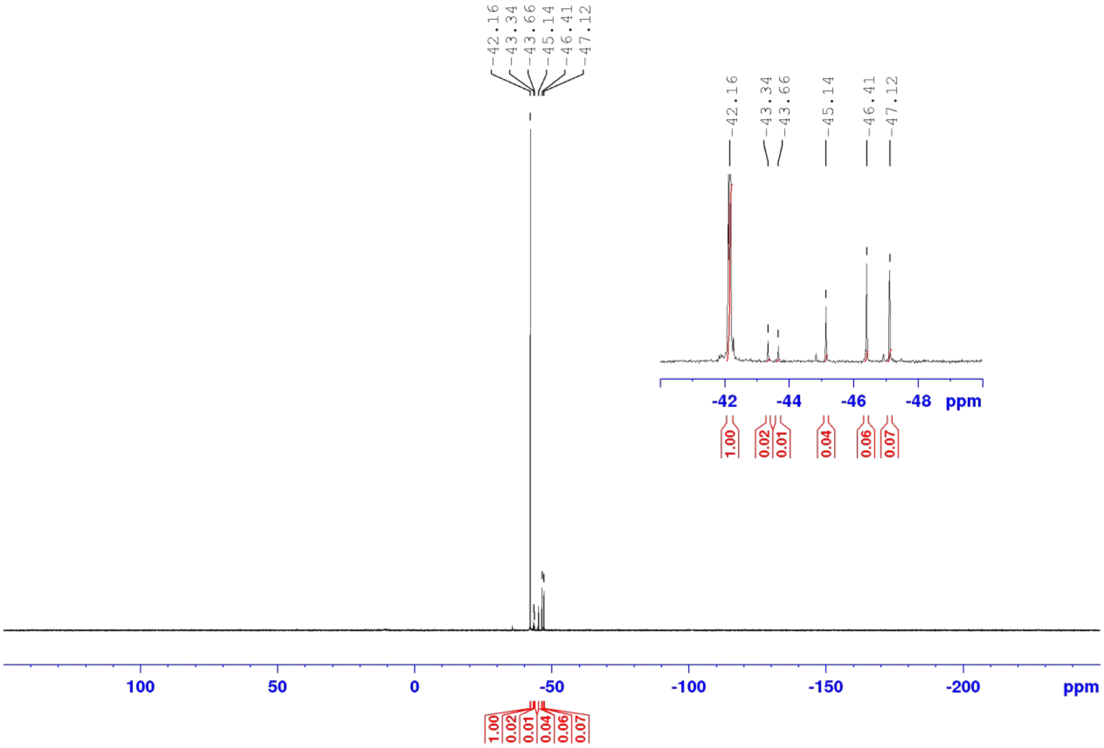

HSQC (500 MHz, C_6D_6)

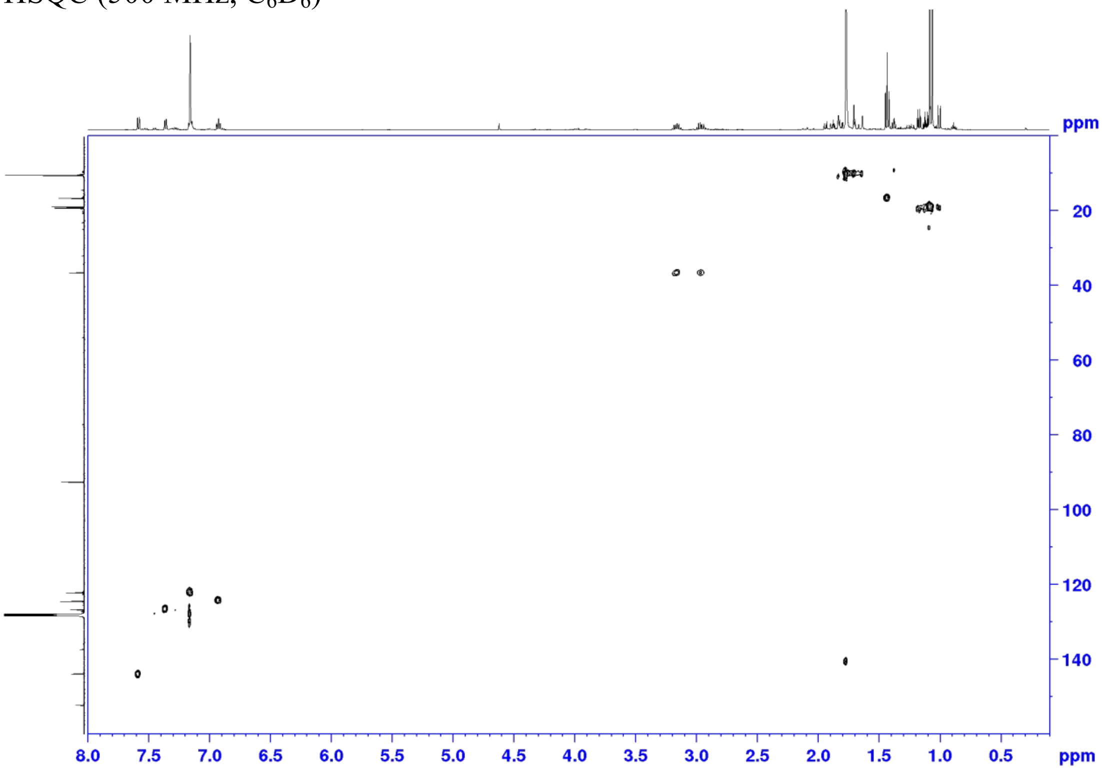

HMBC (500 MHz, C_6D_6)

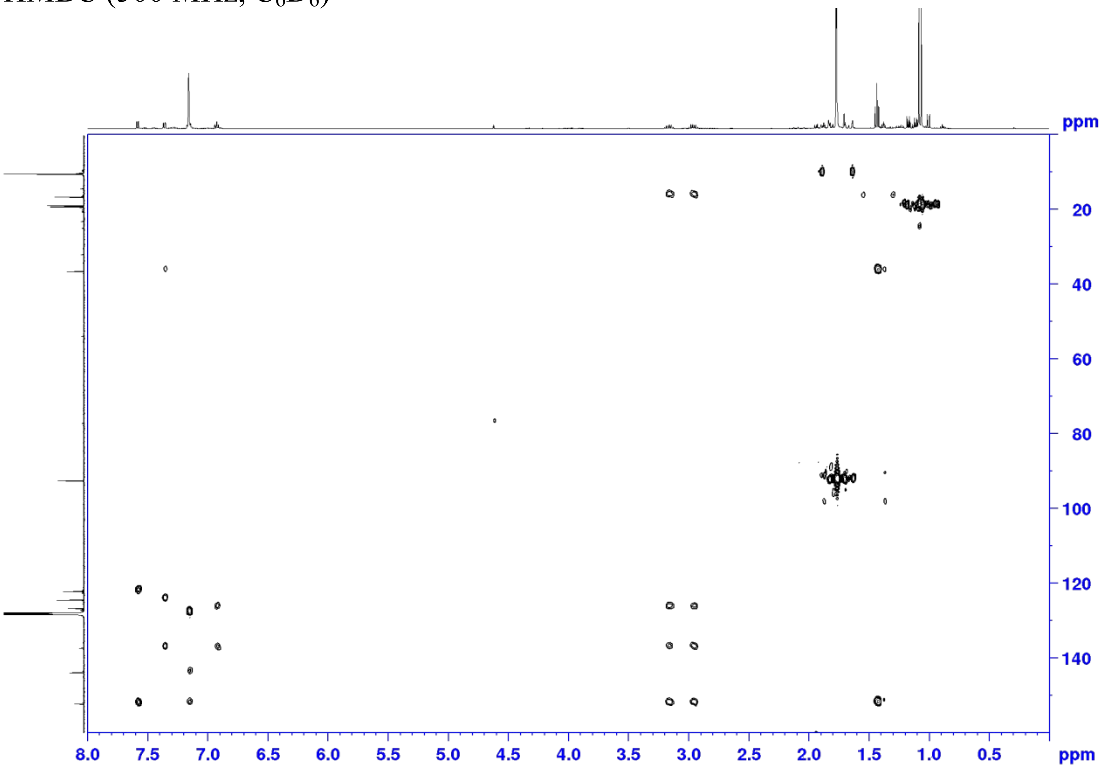

Compound **2a** - [Cp*Ir(PMe_3)(H)(2-isopropylphenyl)]

^{1}H NMR (500 MHz, C_6D_6)

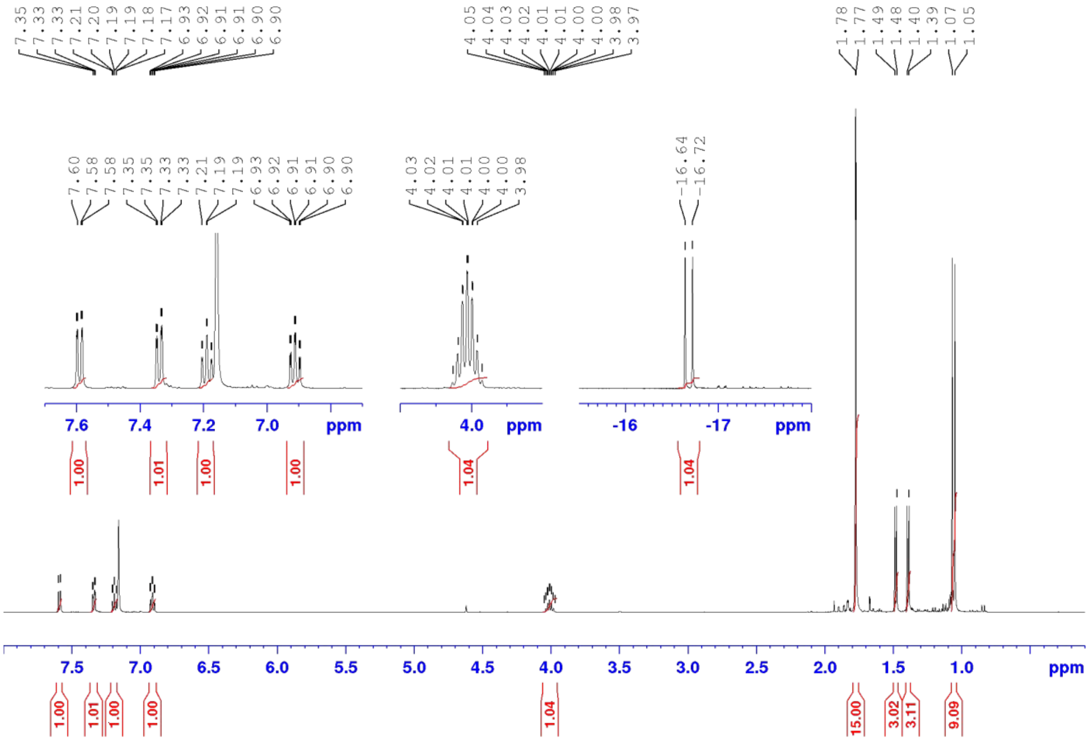

^{13}C{^{1}H} NMR (126 MHz, C_6D_6)

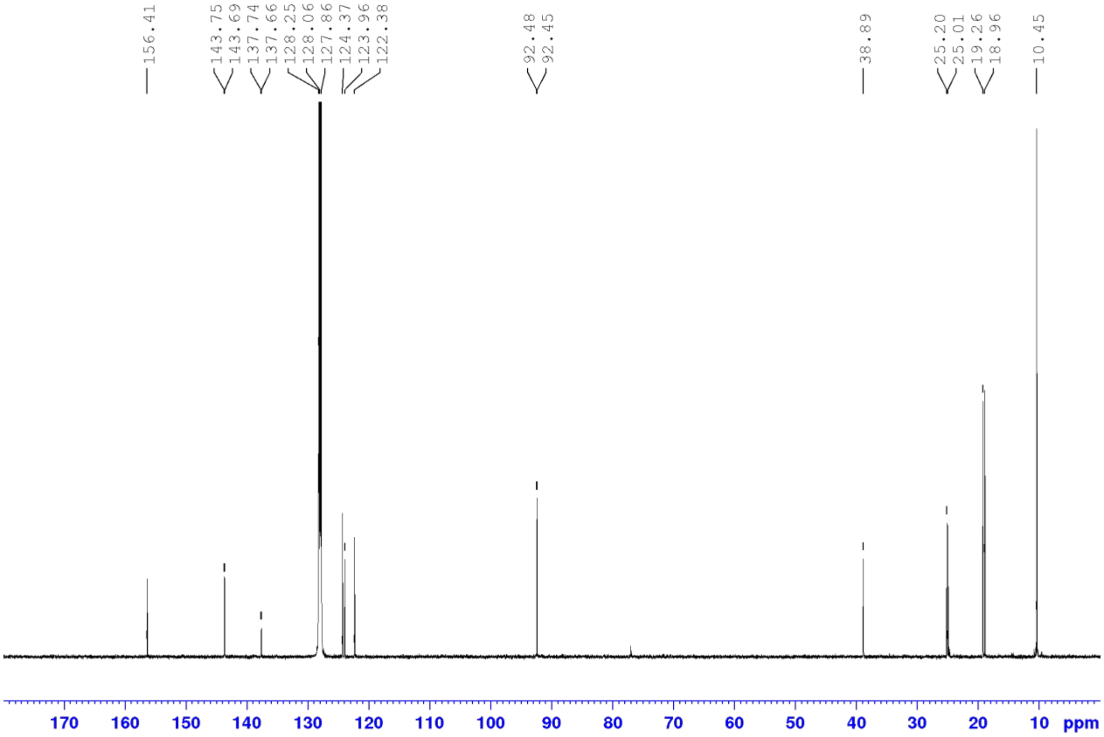

$^{31}P\{^1H\}$ NMR (202 MHz, C_6D_6)

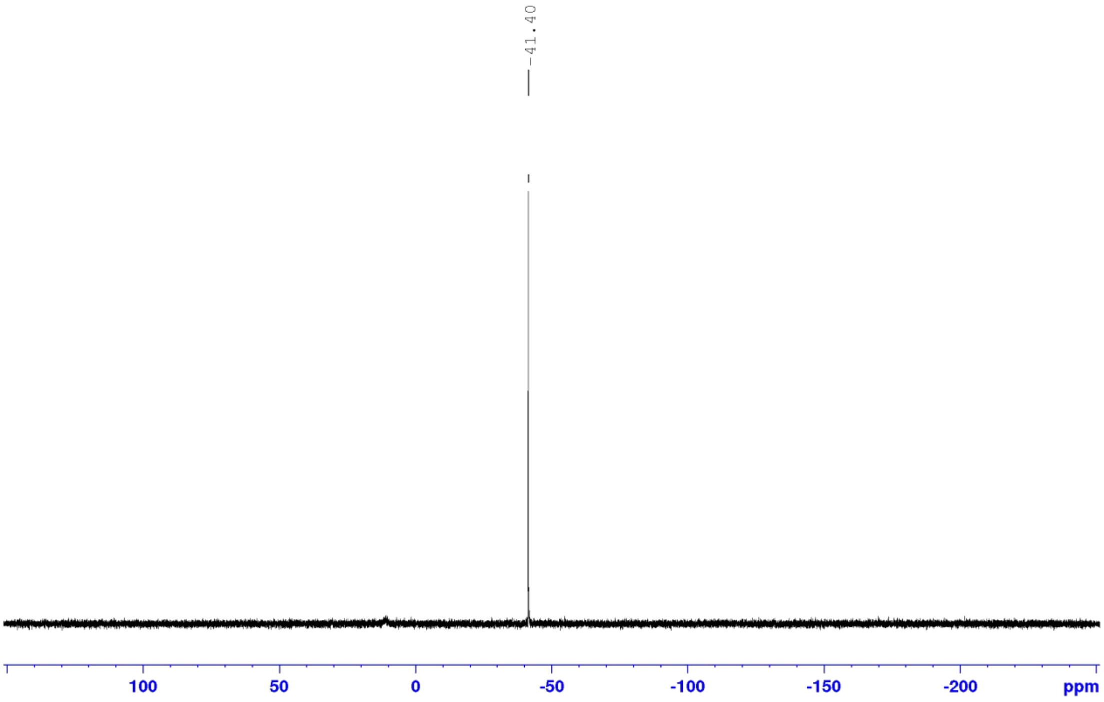

Compound **2a-ph** - [Cp*Ir(PPh_3)(H)(2-isopropylphenyl)]

^{1}H NMR (500 MHz, C_6D_6)

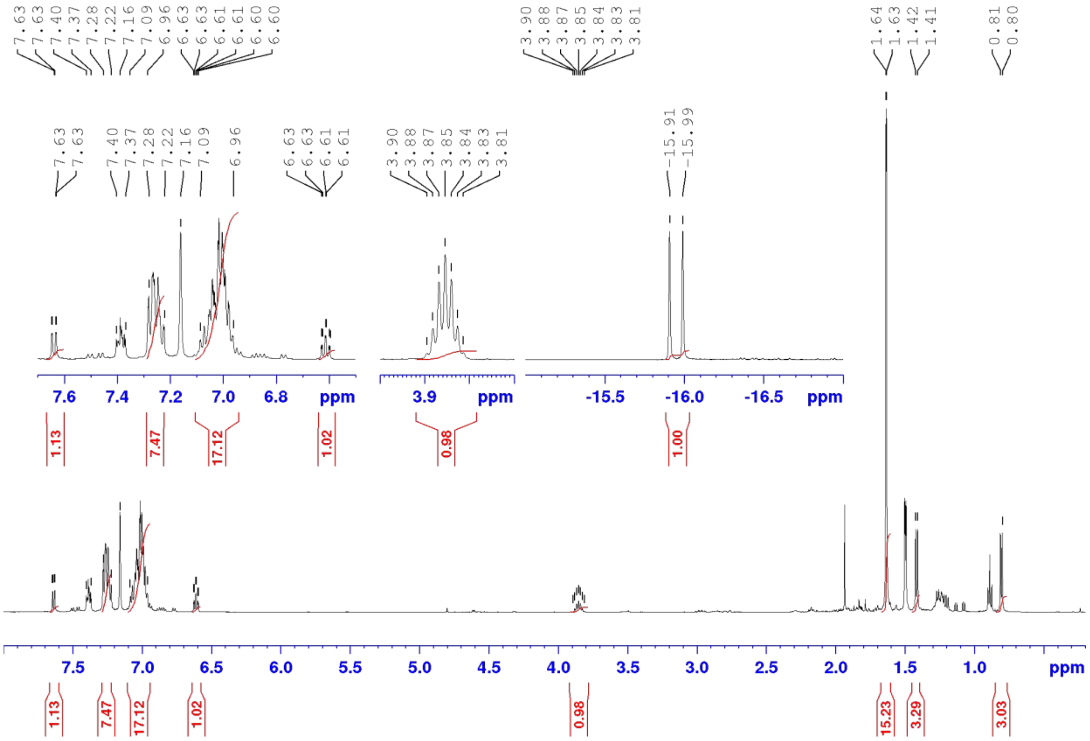

$^{13}C\{^{1}H\}$ NMR (126 MHz, C_6D_6)

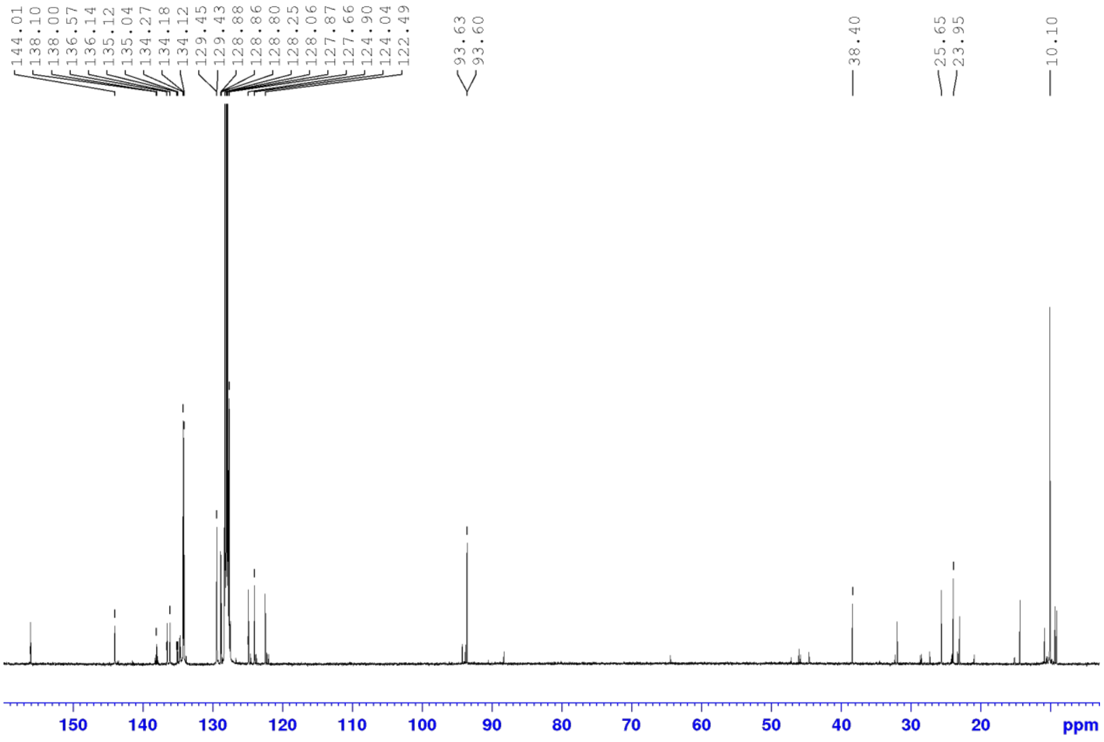

$^{31}P\{^1H\}$ NMR (202 MHz, C_6D_6)

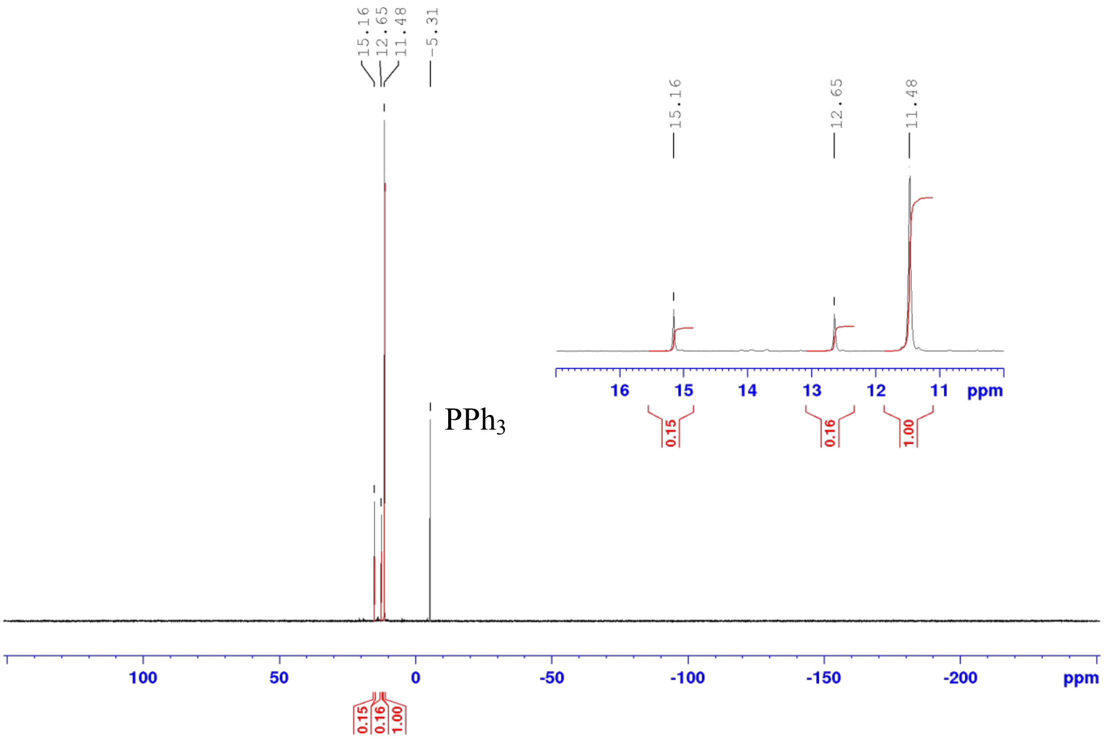

HSQC (500 MHz, C_6D_6)

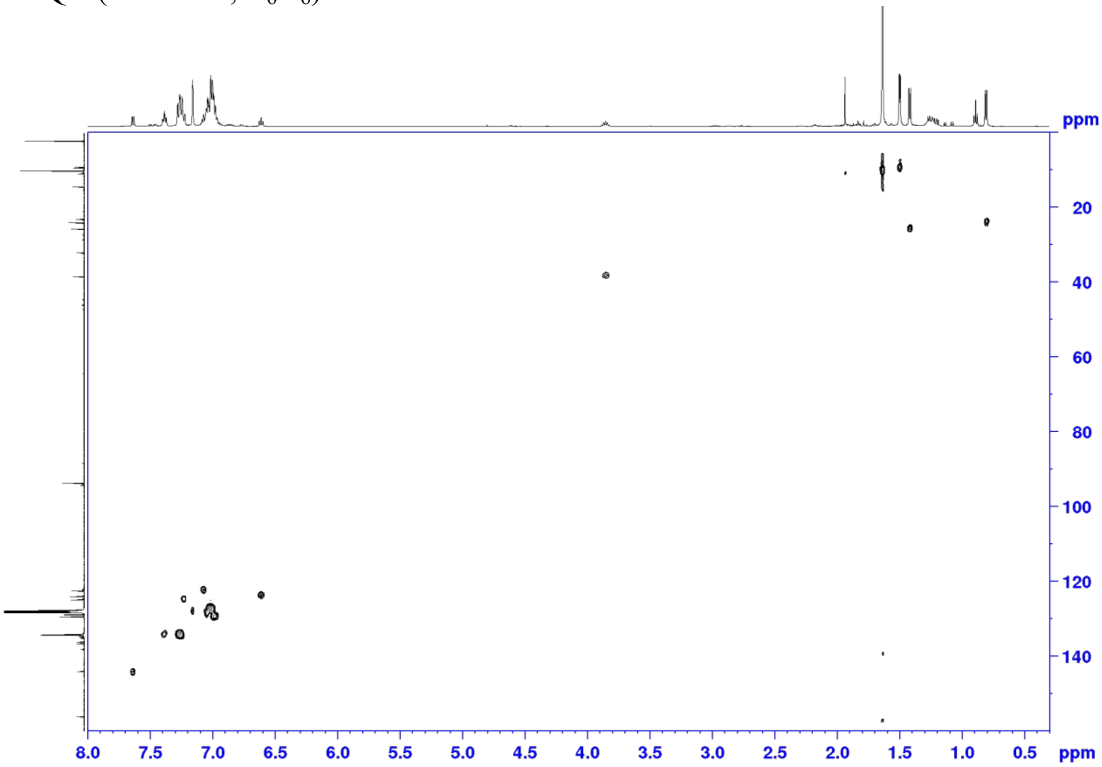

HMBC (500 MHz, C_6D_6)

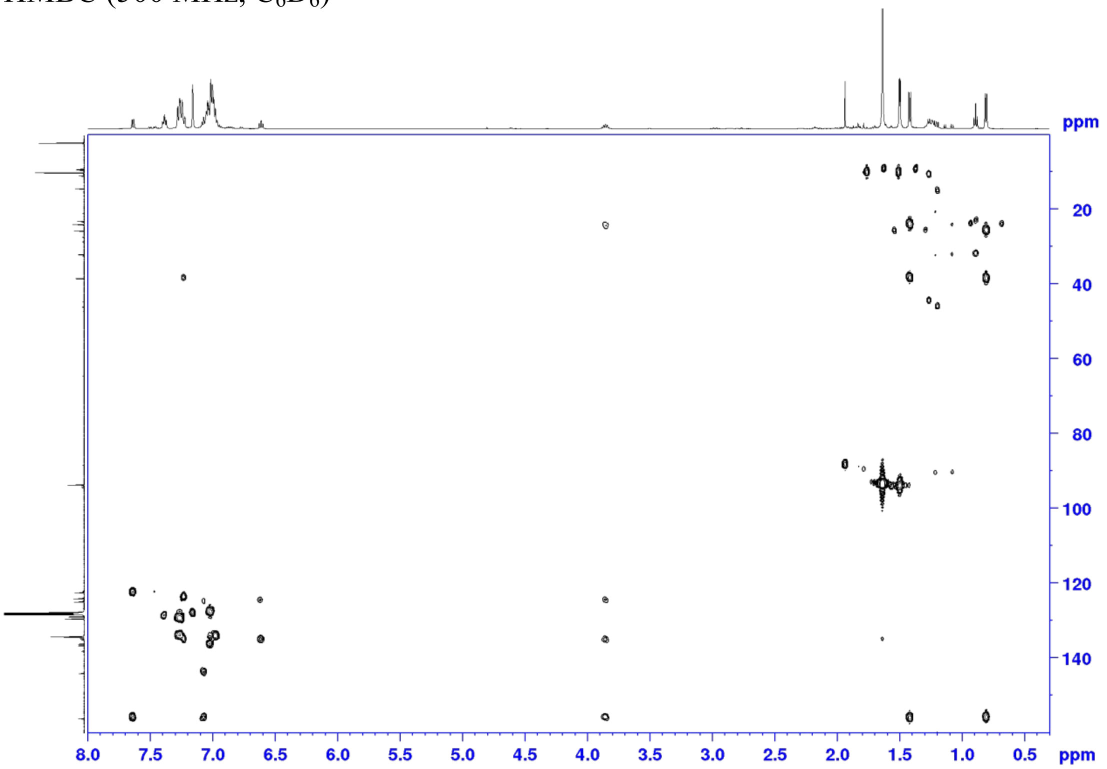

Compound **2b** - [Cp*Ir(PMe$_3$)(H)(2-*sec*-butylphenyl)]

^{1}H NMR (500 MHz, C_6D_6)

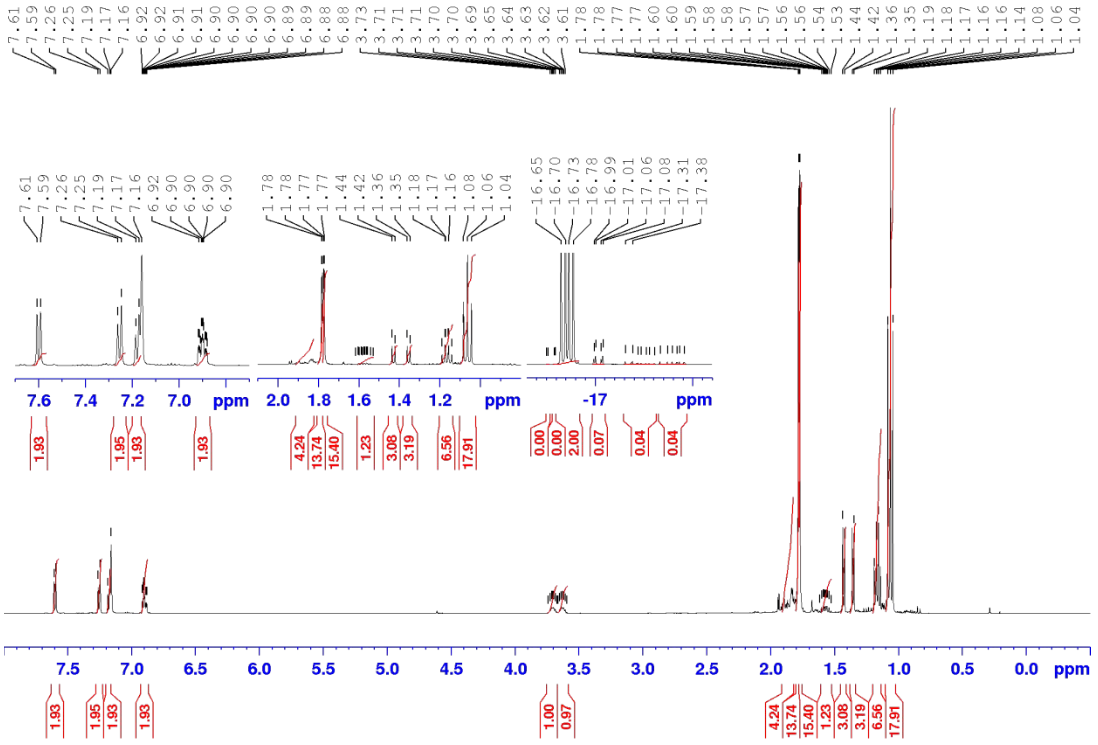

$^{13}C\{^1H\}$ NMR (126 MHz, C_6D_6)

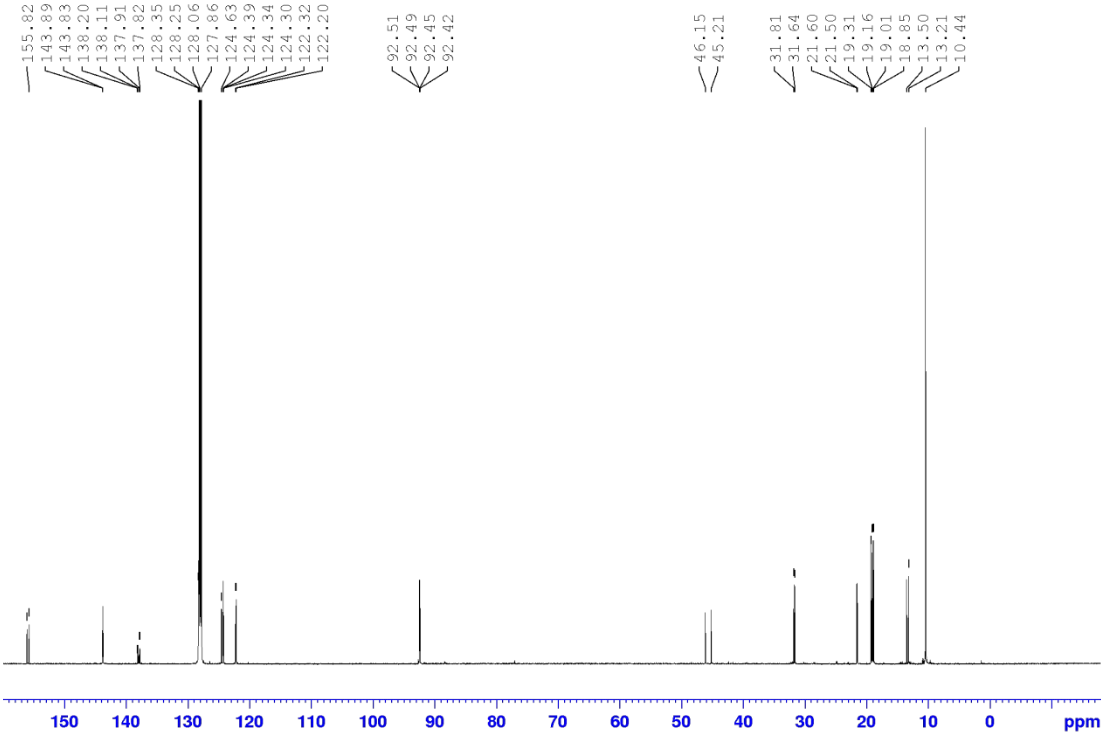

$^{31}P\{^1H\}$ NMR (202 MHz, C_6D_6)

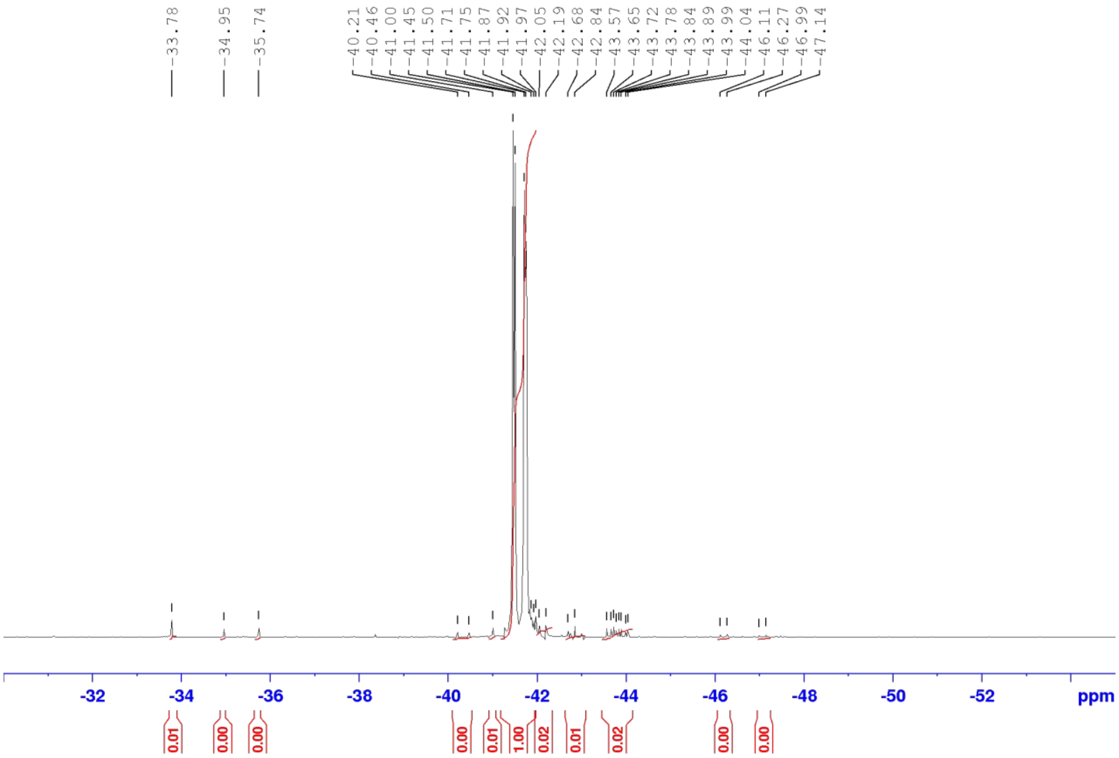

Compound **2c** - [Cp*Ir(PMe_3)(H)(2-(3-pentyl)phenyl)]

^{1}H NMR (500 MHz, C_6D_6)

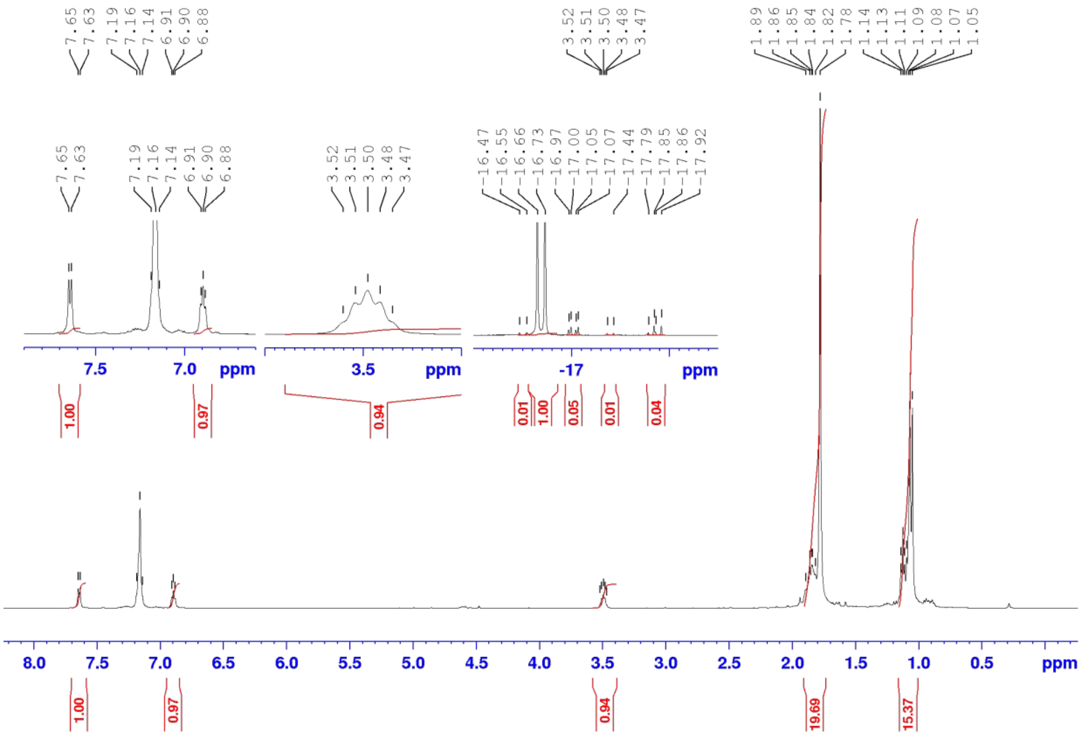

$^{13}C\{^{1}H\}$ NMR (100 MHz, C_6D_6)

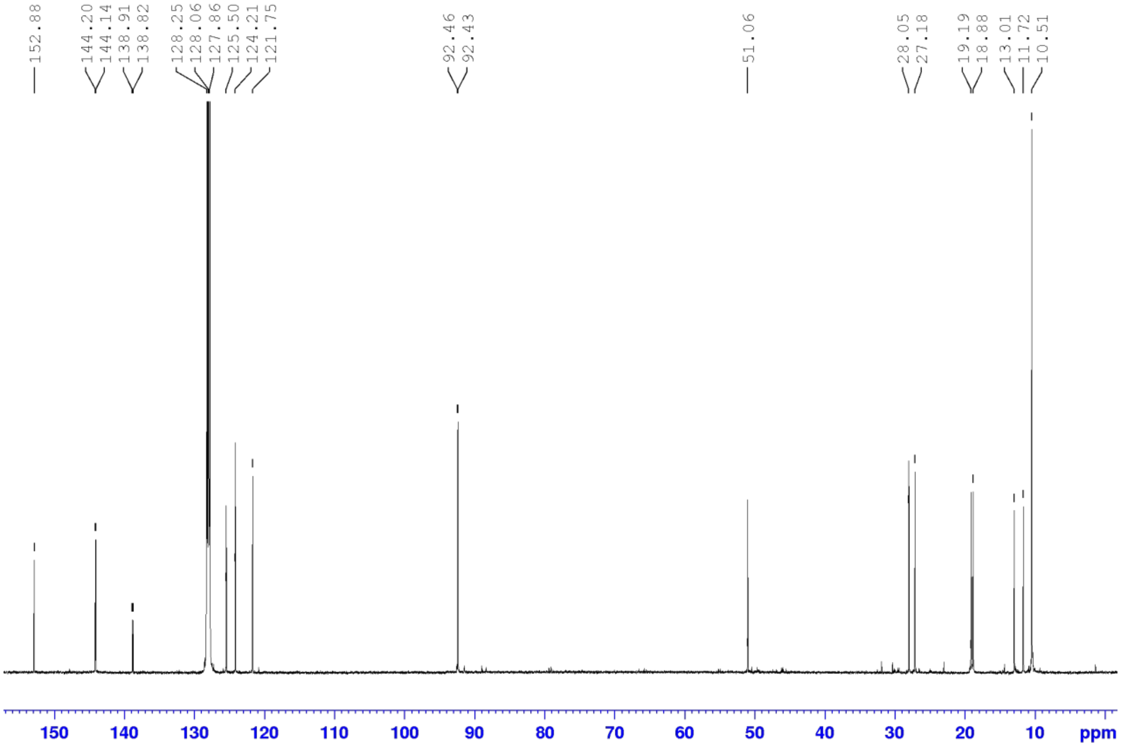

$^{31}P\{^1H\}$ NMR (202 MHz, C_6D_6)

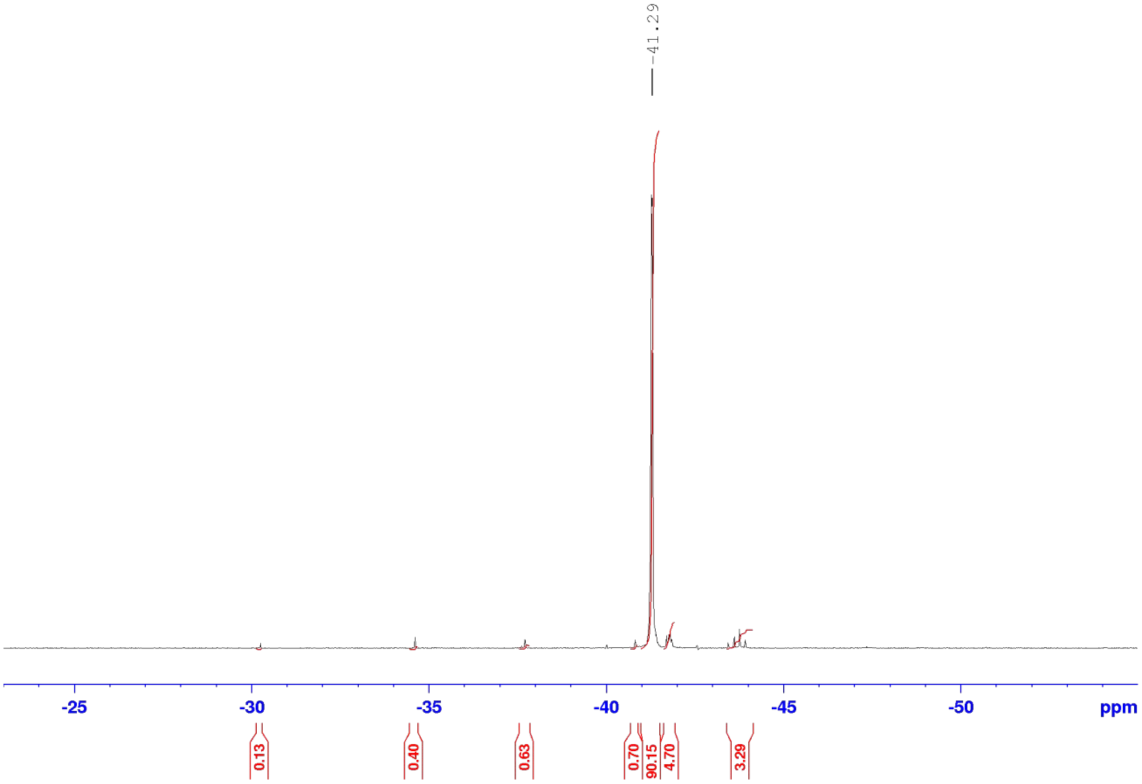

Compound **2d** - [Cp*Ir(PMe_3)(H)(2-cyclopentylphenyl)]

^1H NMR (500 MHz, C_6D_6)

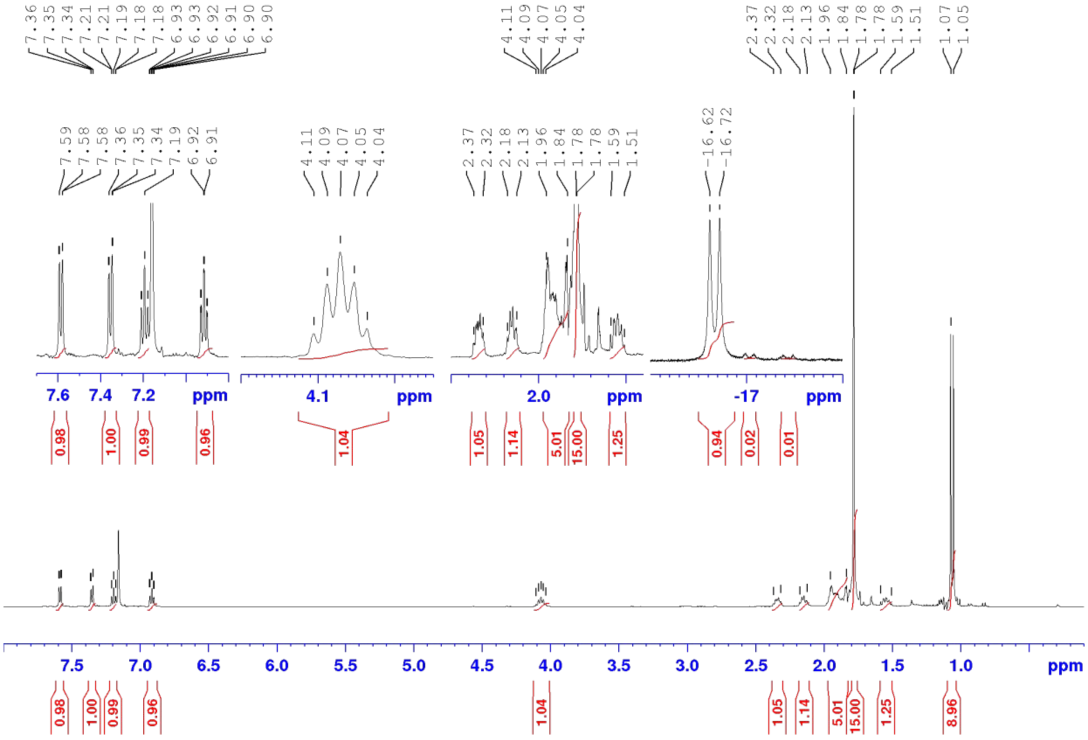

$^{13}C\{^1H\}$ NMR (126 MHz, C_6D_6)

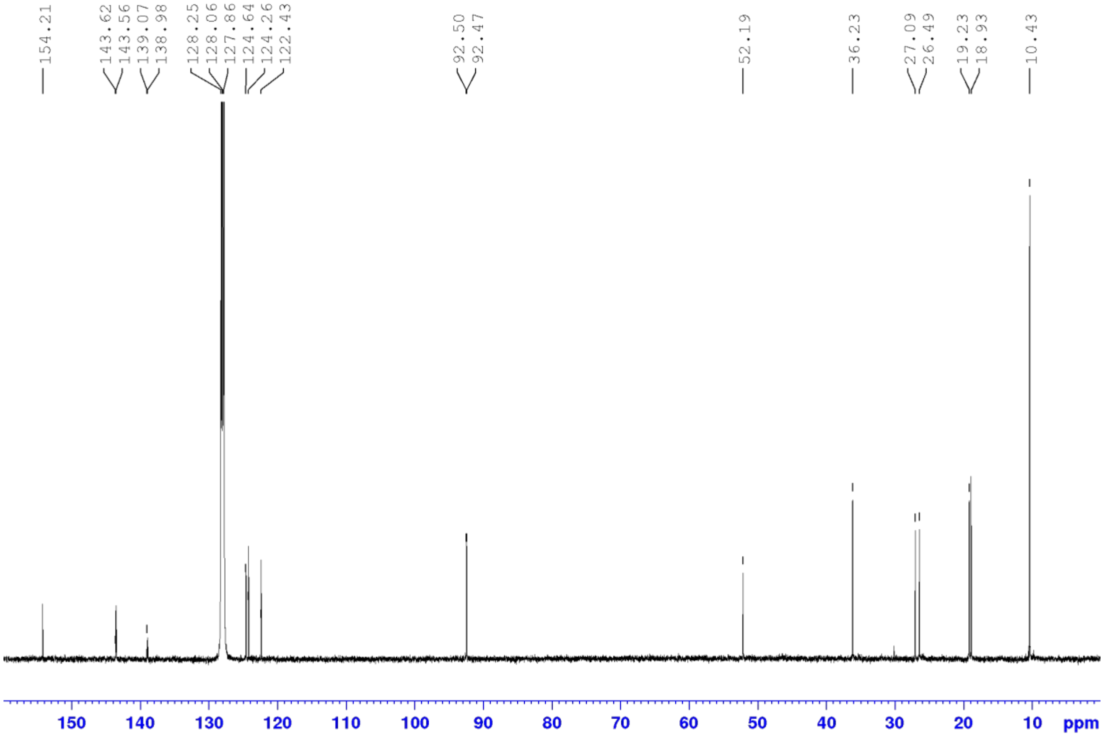

$^{31}P\{^{1}H\}$ NMR (202 MHz, C_6D_6)

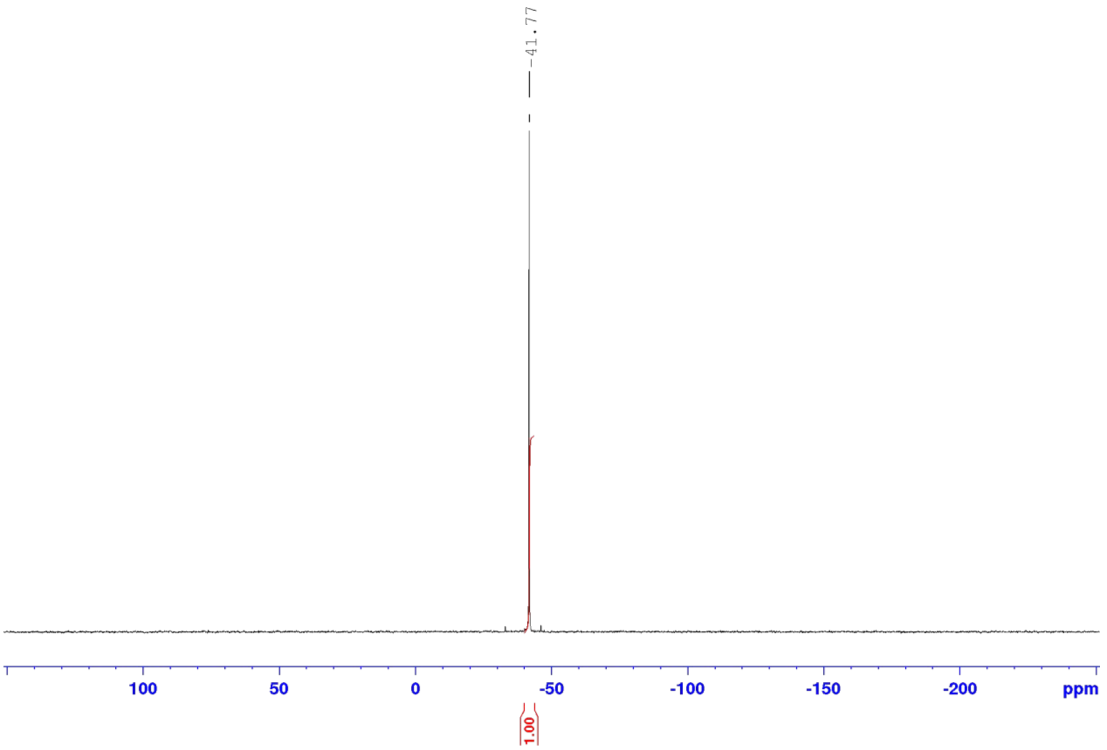

Compound **2e** - [Cp*Ir(PMe_3)(H)(2-cyclohexylphenyl)]

^{1}H NMR (400 MHz, C_6D_6)

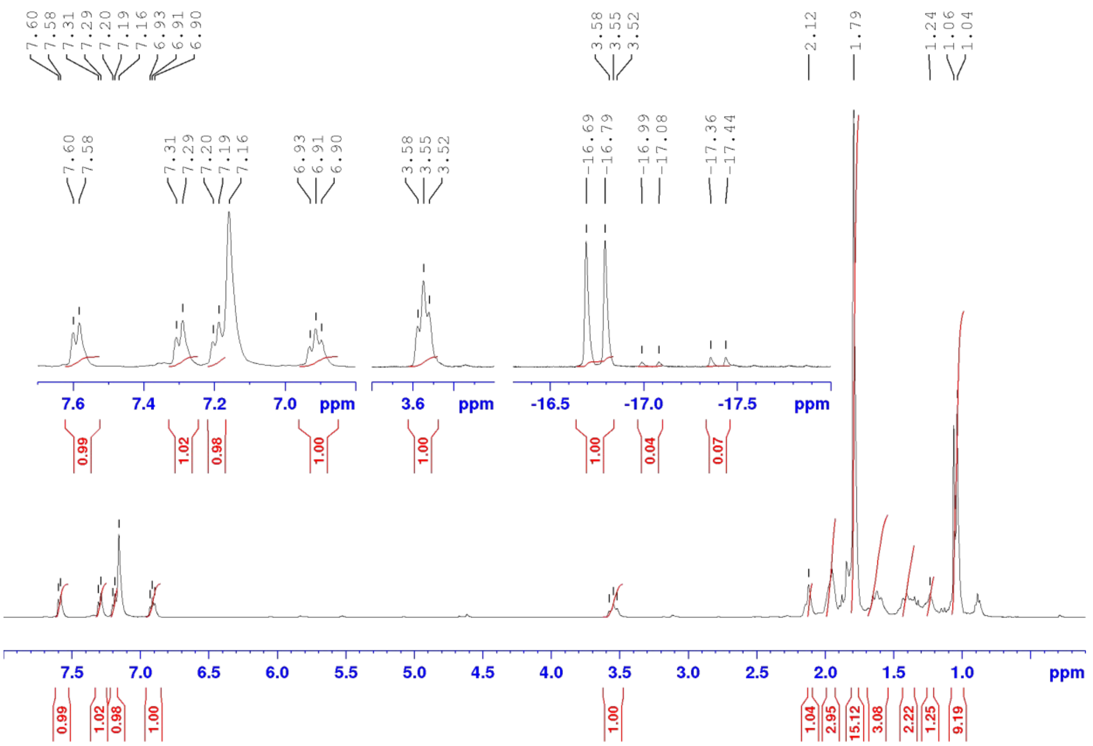

$^{13}C\{^1H\}$ NMR (100 MHz, C_6D_6)

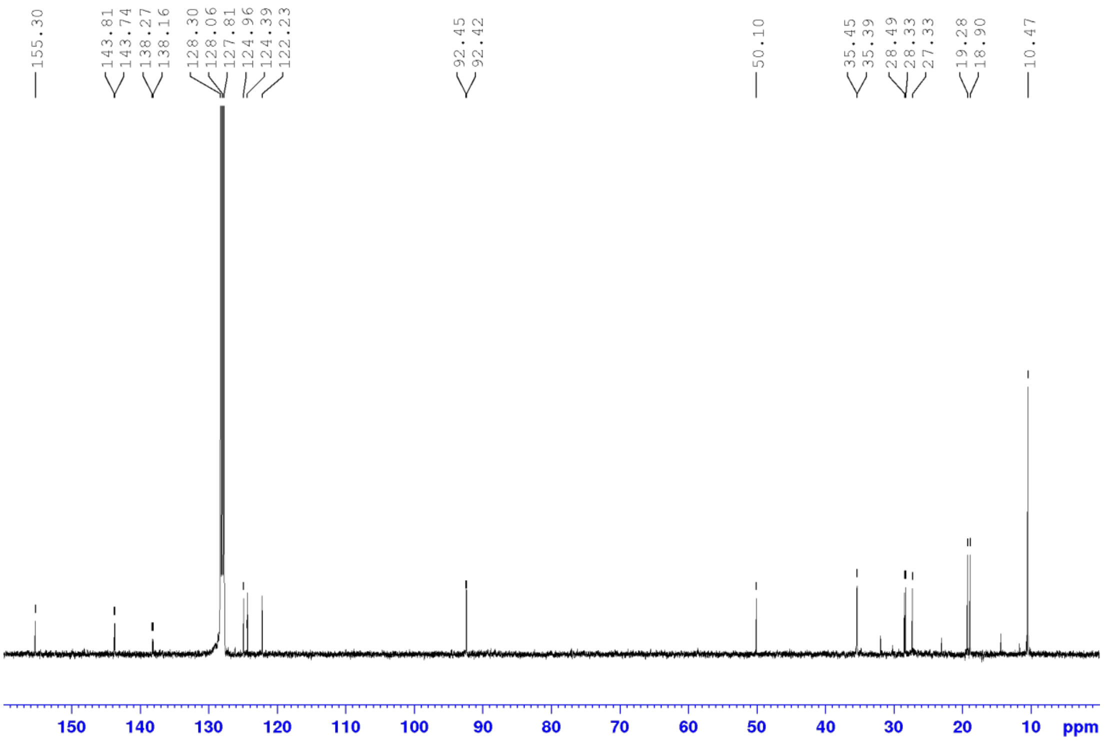

$^{31}P\{^1H\}$ NMR (162 MHz, C_6D_6)

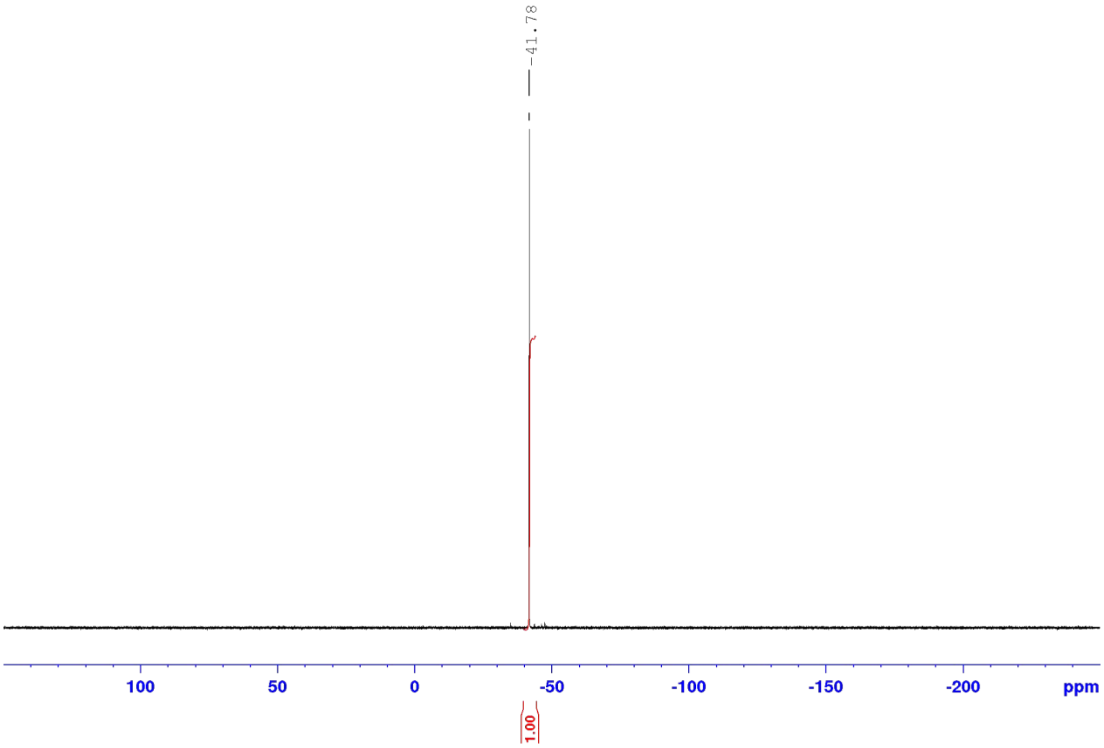

Compound **2g** - [Cp*Ir(PMe$_3$)(H)(2-*n*-propylphenyl)]

^{1}H NMR (500 MHz, C_6D_6)

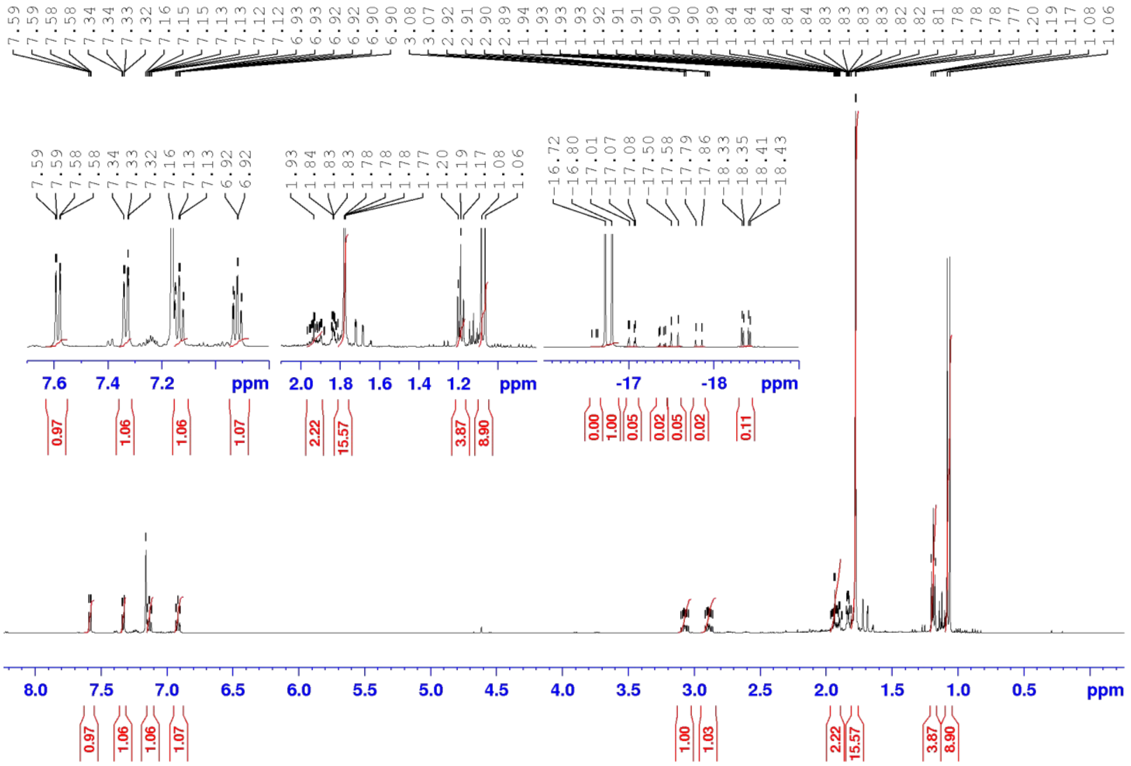

^{13}C{^{1}H} NMR (126 MHz, C_6D_6)

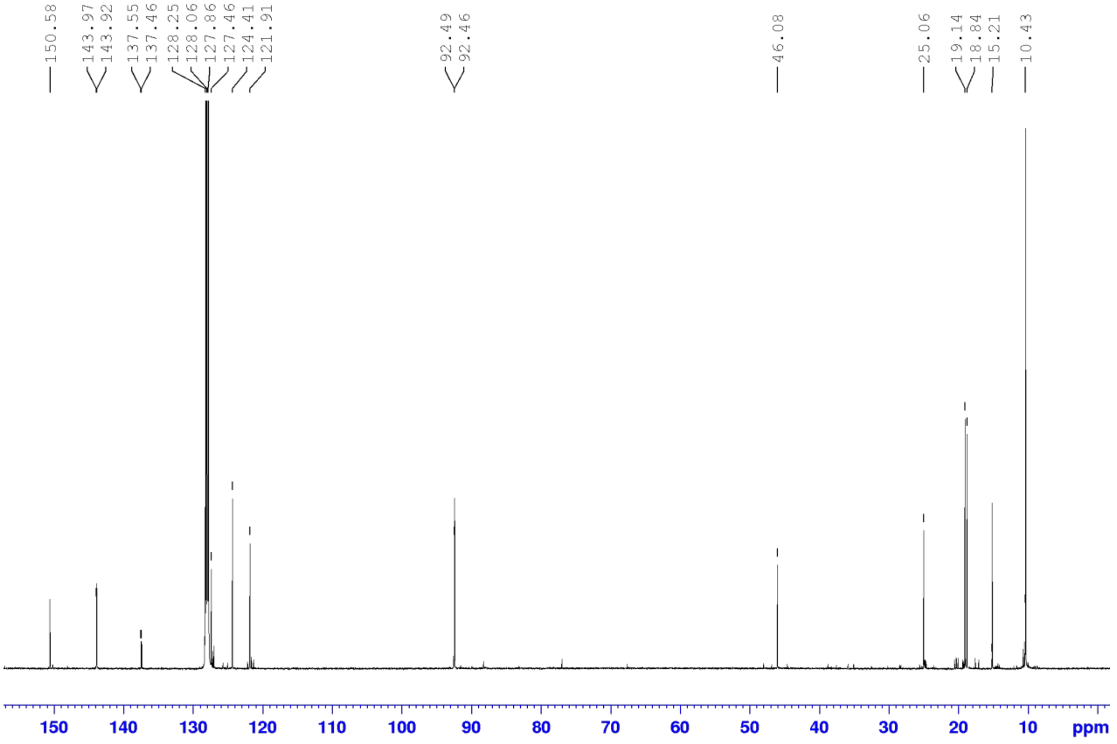

$^{31}P\{^{1}H\}$ NMR (202 MHz, C_6D_6)

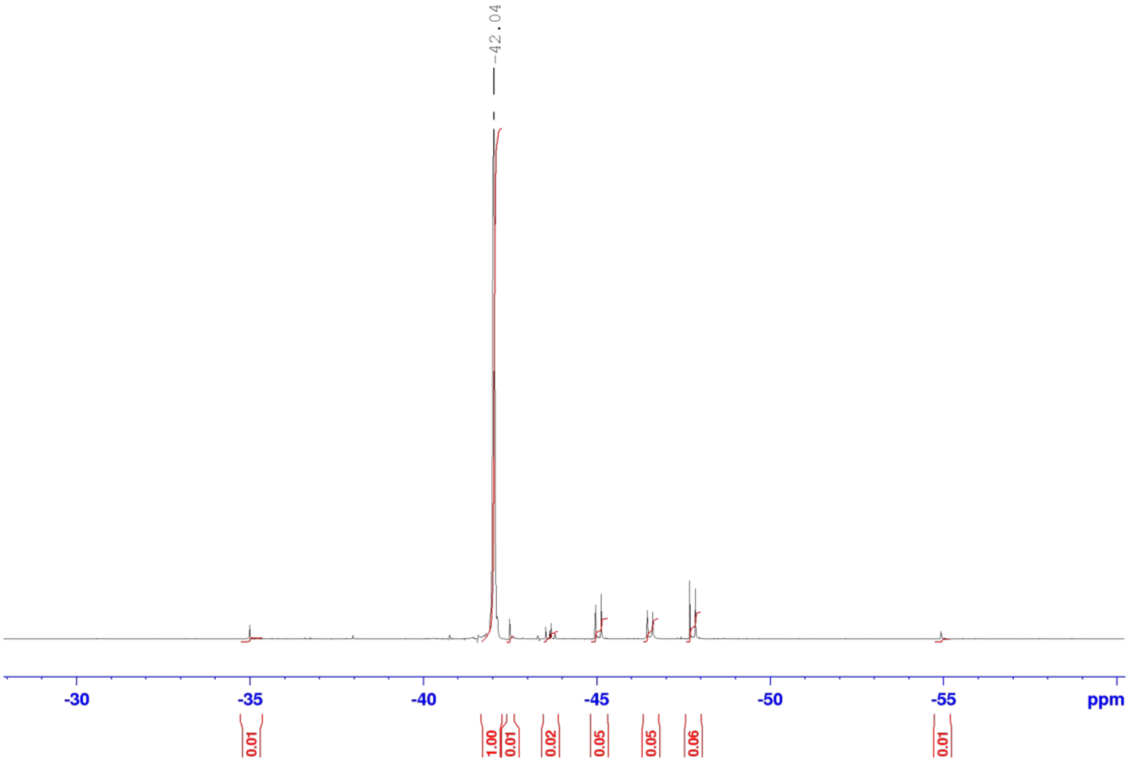

Compound **2h** - [Cp*Ir(PMe_3)(H)(2-*n*-butylphenyl)]

^{1}H NMR (500 MHz, C_6D_6)

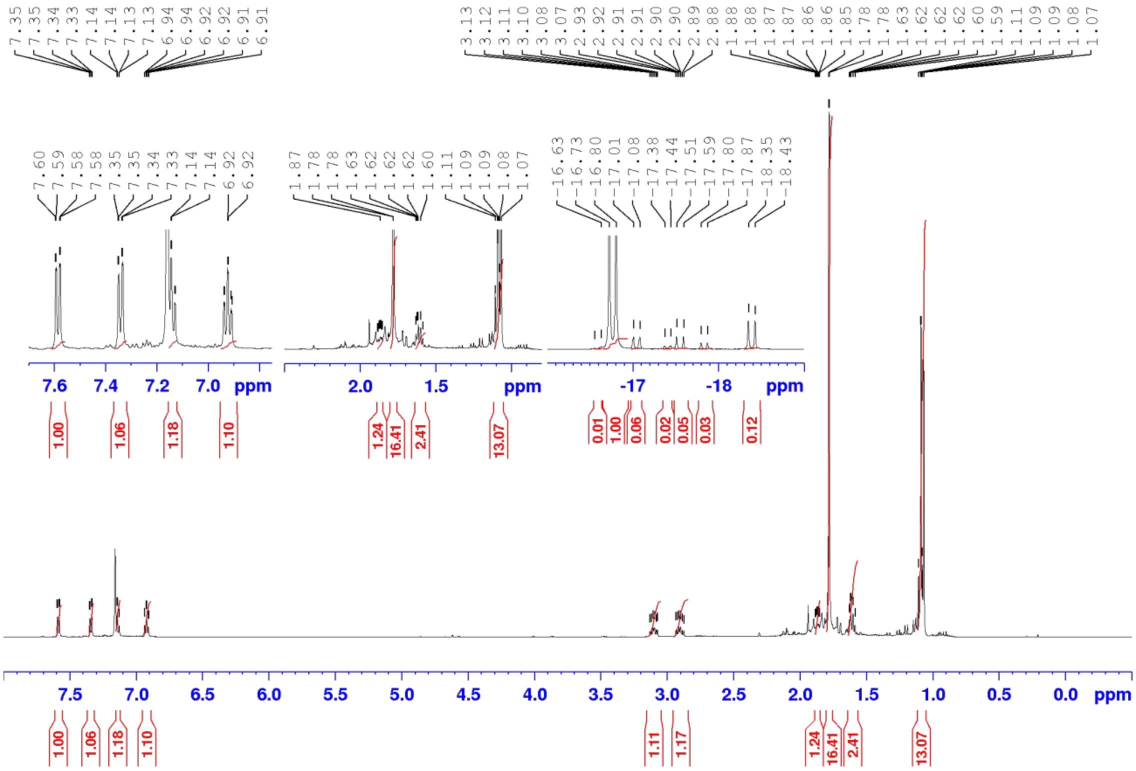

^{13}C{^{1}H} NMR (126 MHz, C_6D_6)

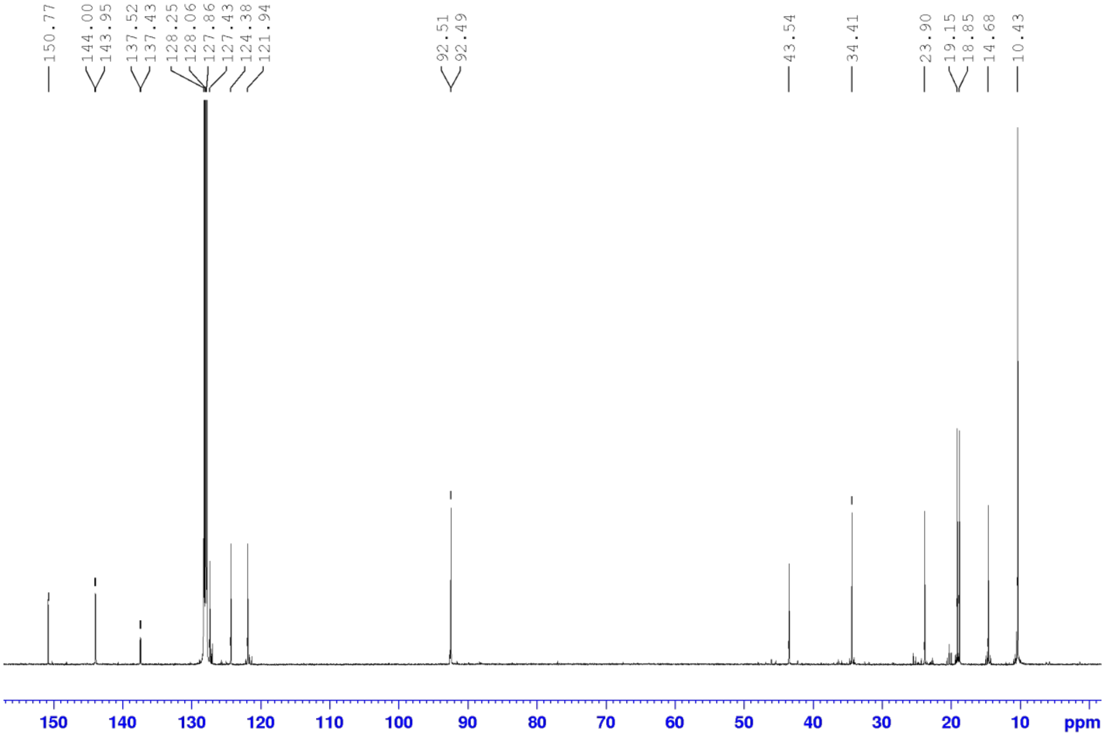

$^{31}P\{^{1}H\}$ NMR (202 MHz, C_6D_6)

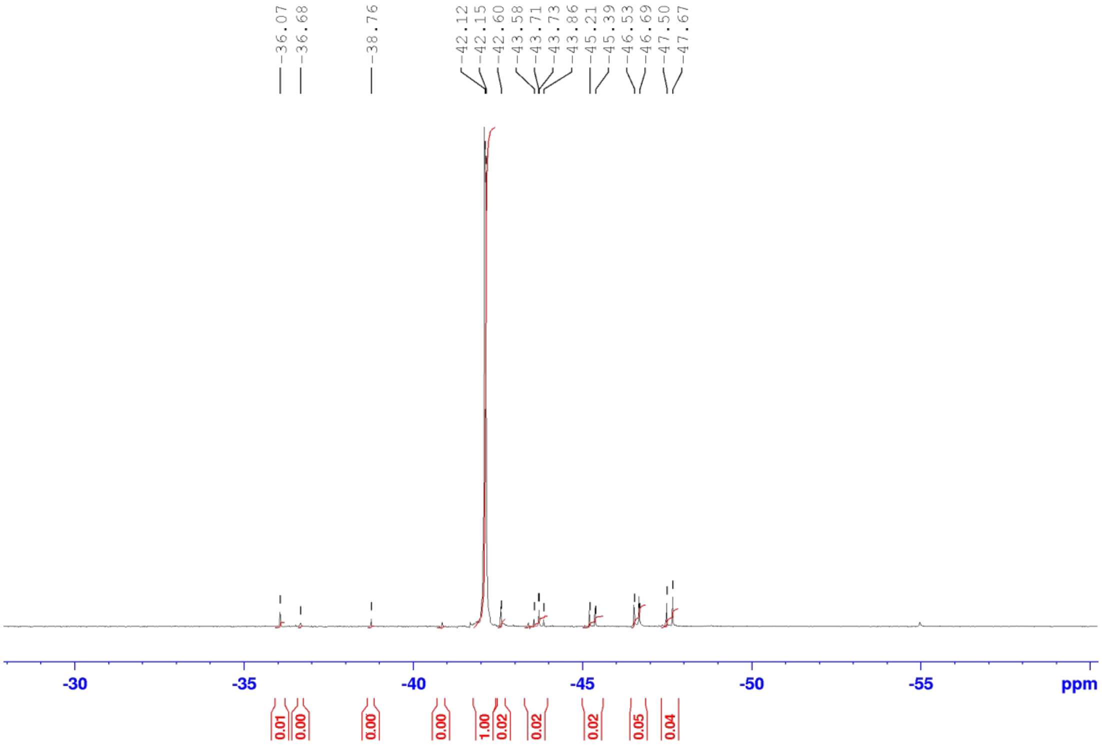

Compound **2i** - [Cp*Ir(PMe_3)(H)(2-*iso*-butylphenyl)]

^{1}H NMR (500 MHz, C_6D_6)

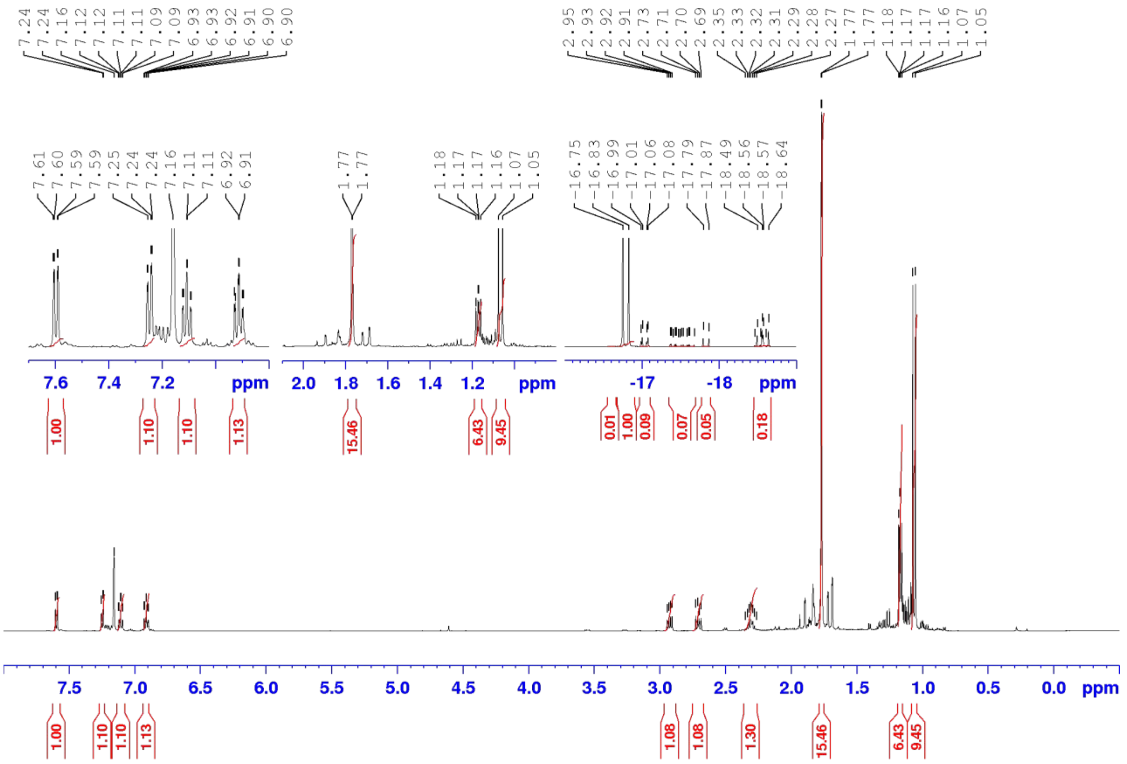

^{13}C{^{1}H} NMR (126 MHz, C_6D_6)

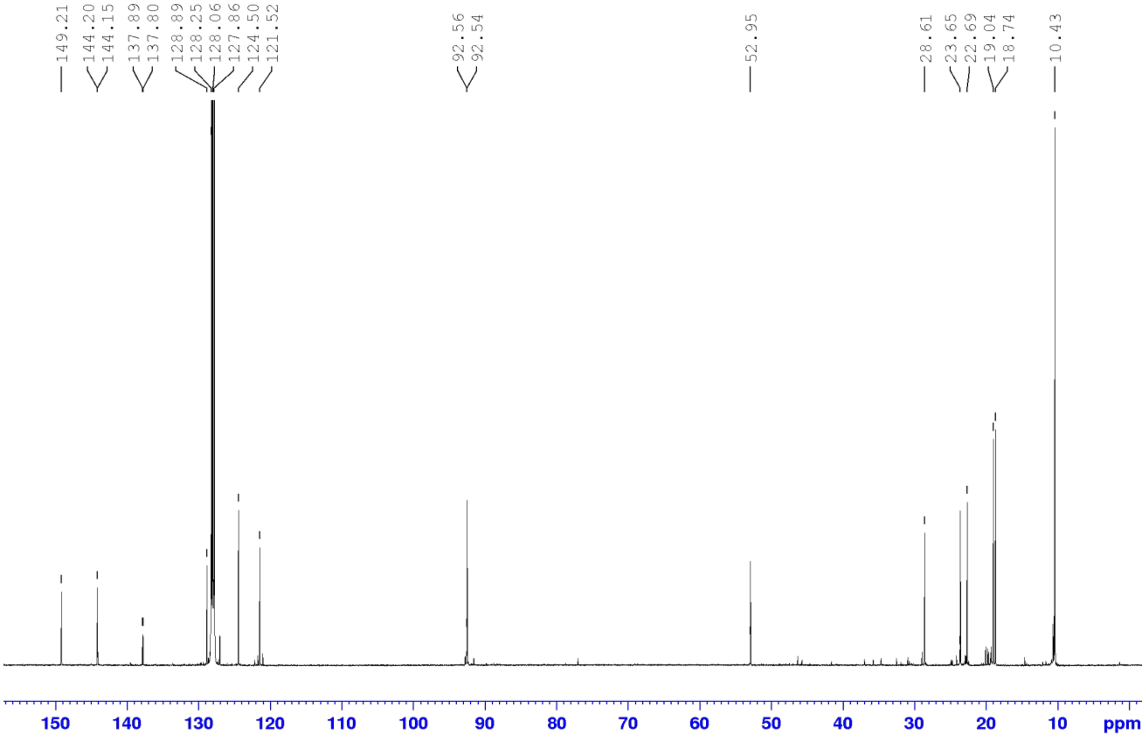

$^{31}P\{^{1}H\}$ NMR (202 MHz, C_6D_6)

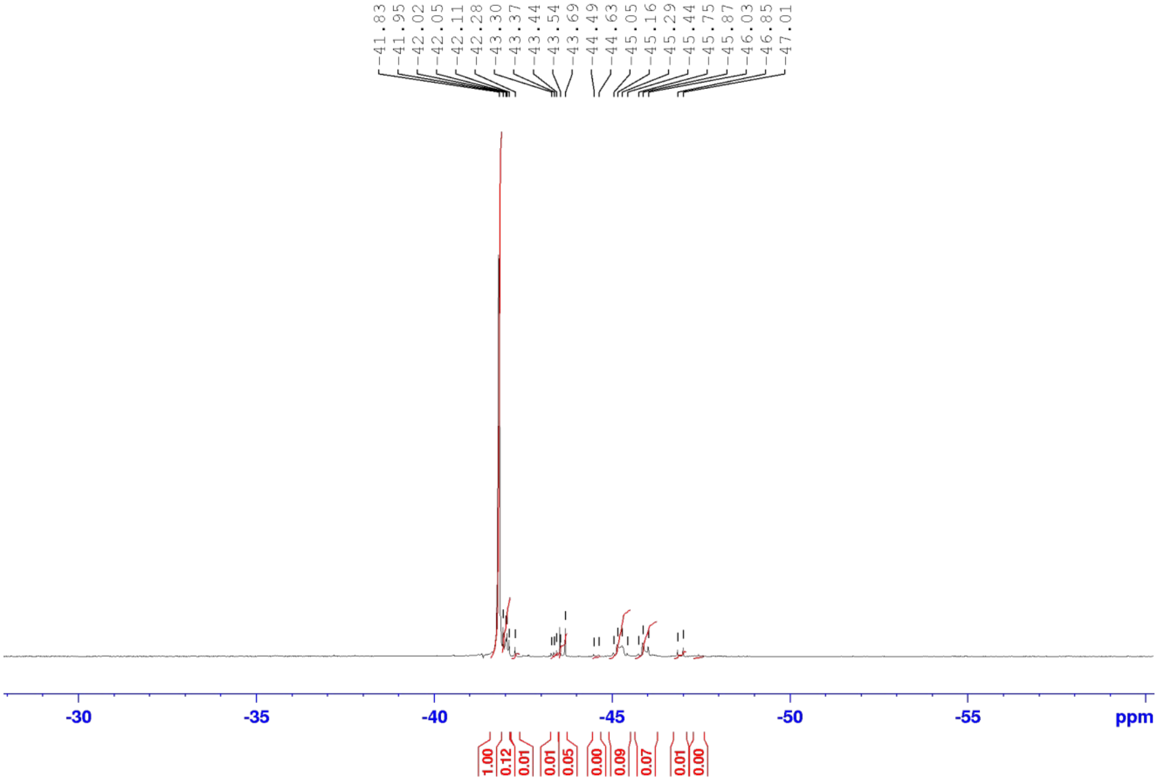

Compound **2j** - [Cp*Ir(PMe$_3$)(H)(2-neopentylphenyl)]

^{1}H NMR (500 MHz, C_6D_6)

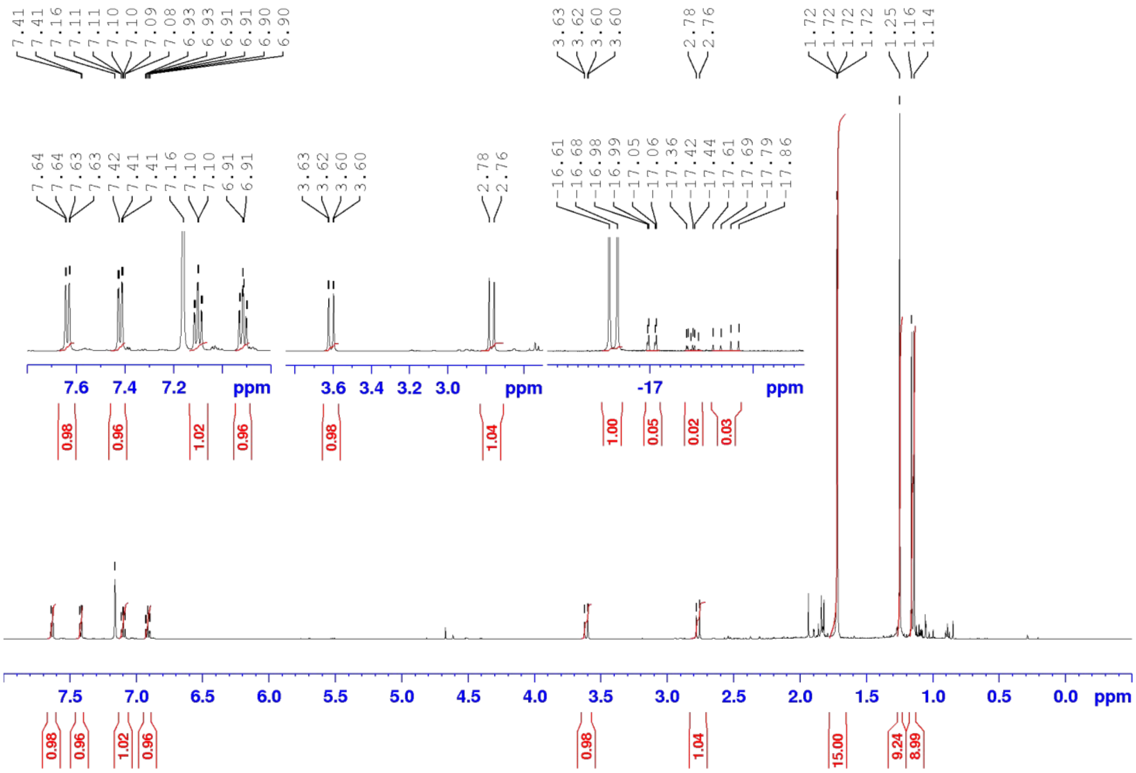

^{13}C{^{1}H} NMR (126 MHz, C_6D_6)

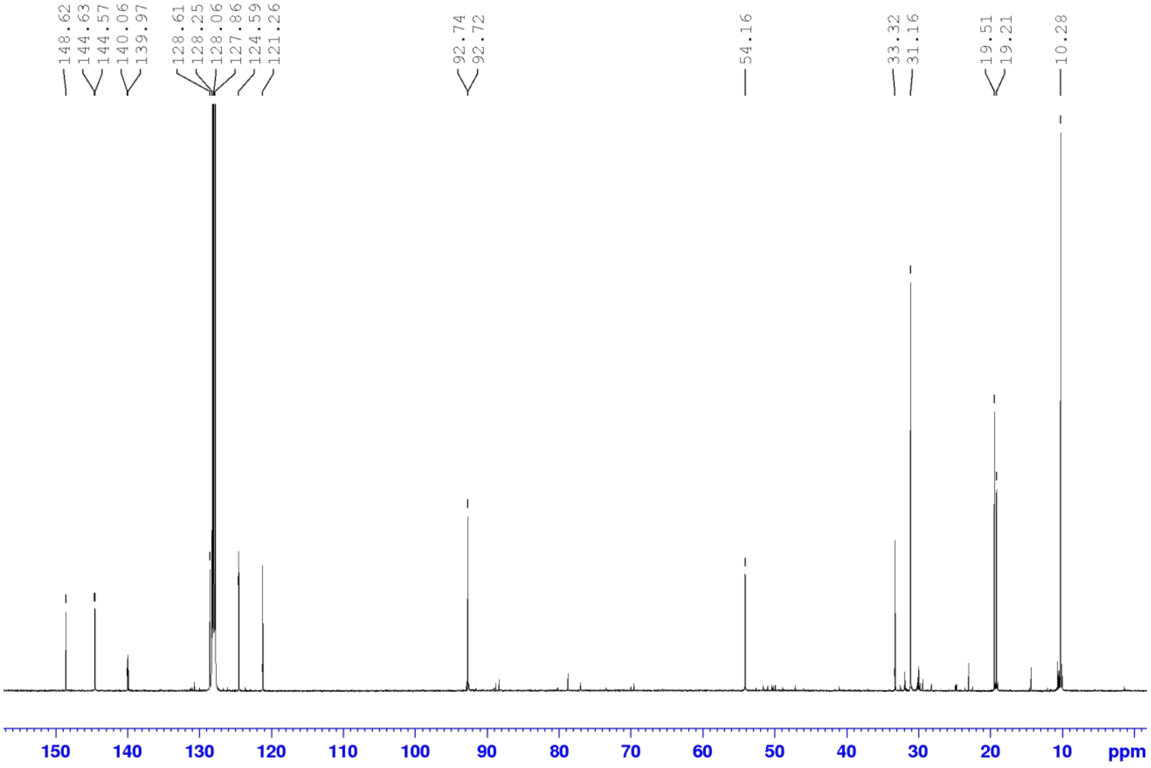

$^{31}P\{^1H\}$ NMR (202 MHz, C_6D_6)

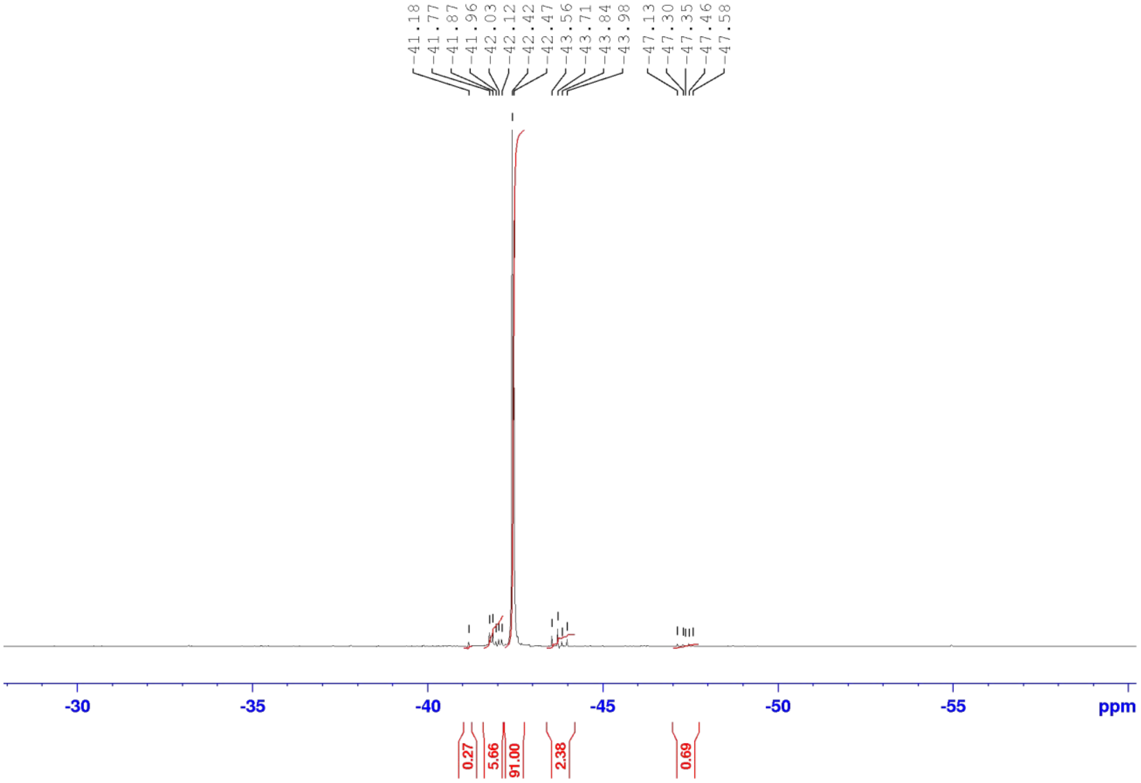

Compound **2l** - [Cp*Ir(PMe_3)(H)(2,5-diisopropylphenyl)]

^{1}H NMR (500 MHz, C_6D_6)

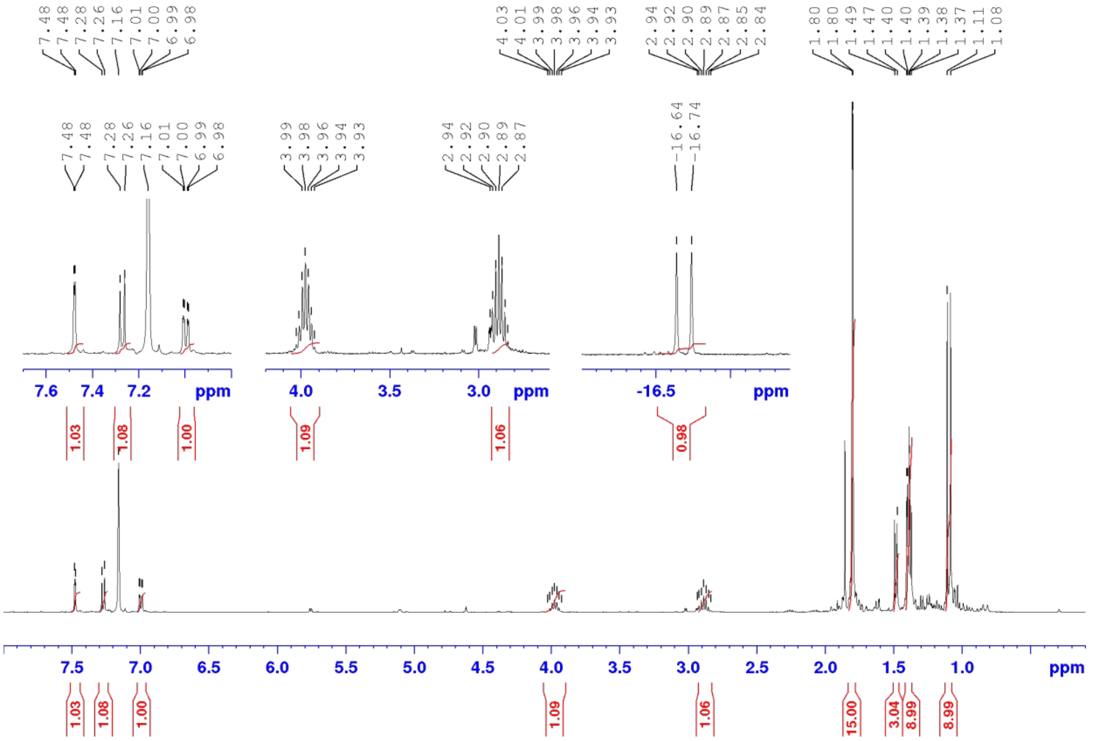

$^{13}C\{^{1}H\}$ NMR (126 MHz, C_6D_6)

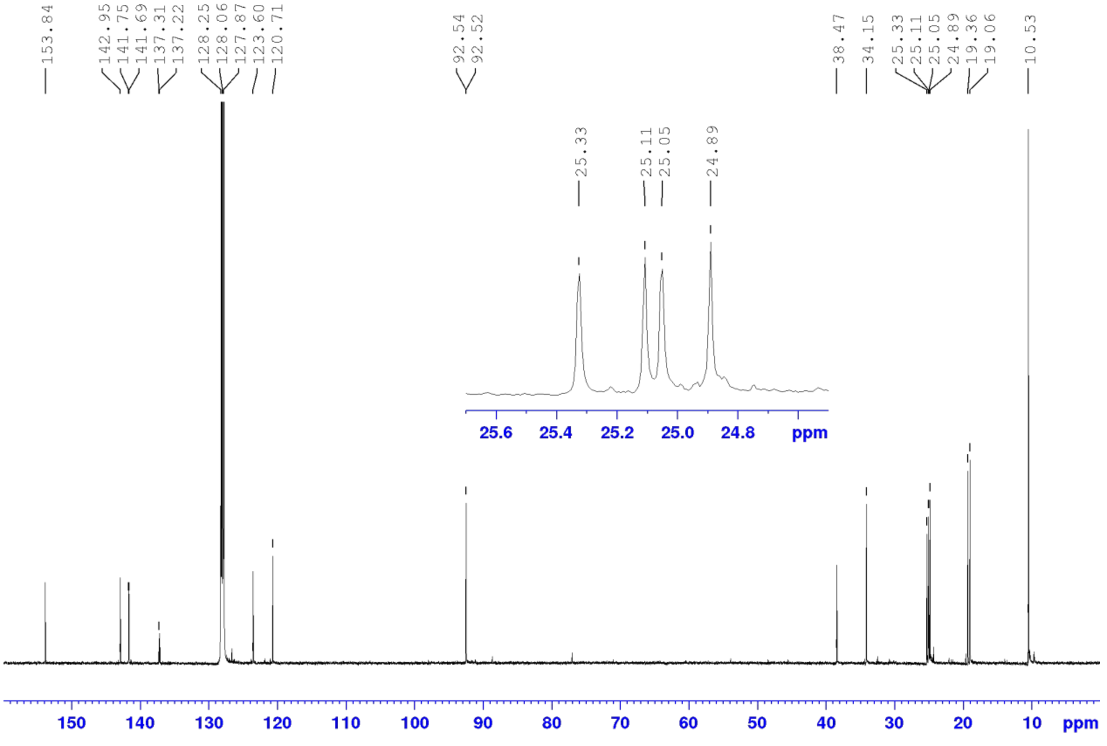

$^{31}P\{^1H\}$ NMR (202 MHz, C_6D_6)

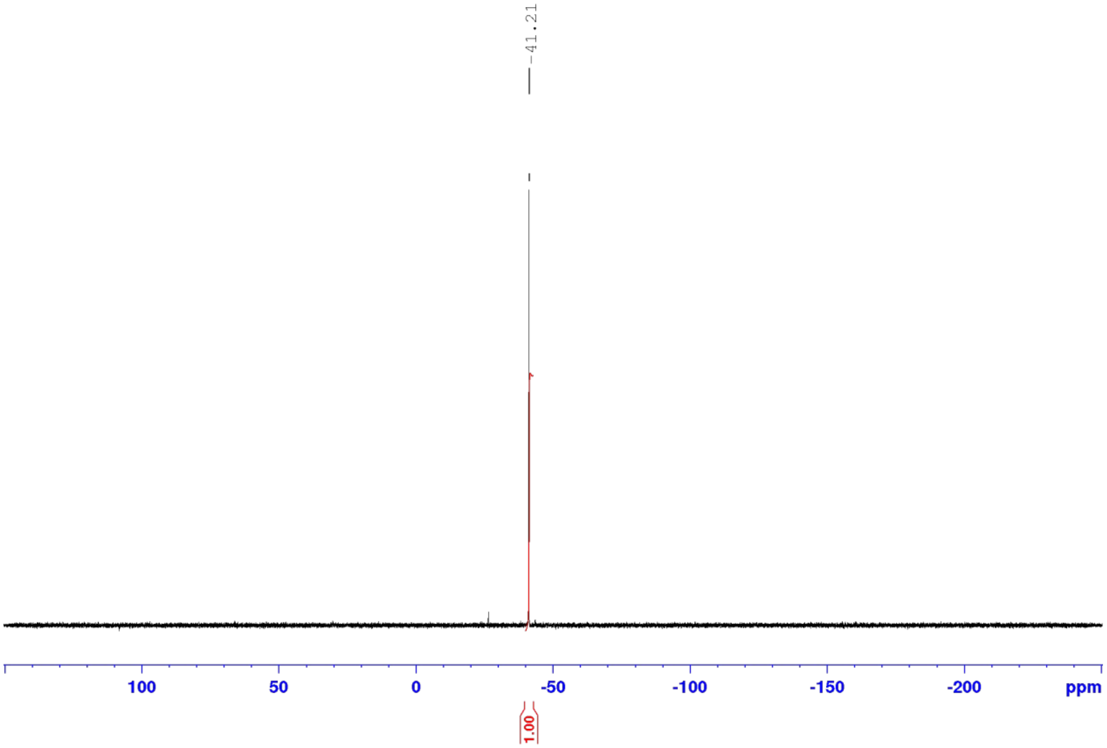

Compound **2l-d₂** - [Cp*Ir(PMe₃)(H)(2,5-diisopropylphenyl-d₂)]

^{1}H NMR (500 MHz, C_6D_6)

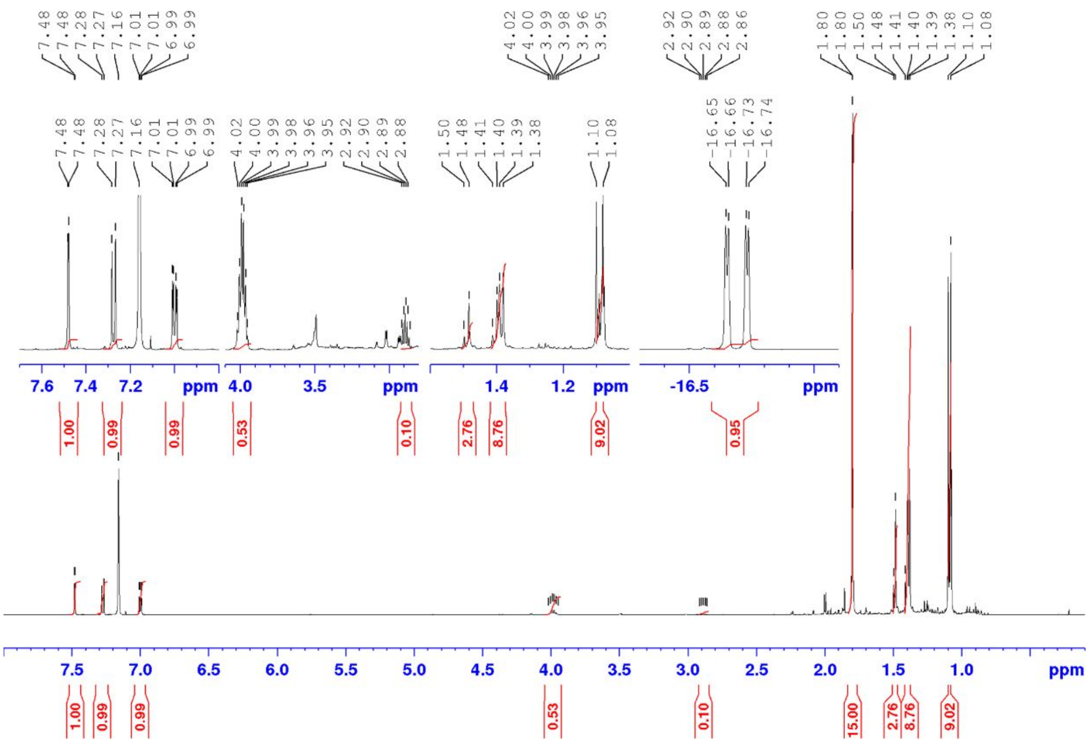

^{13}C{^{1}H} NMR (126 MHz, C_6D_6)

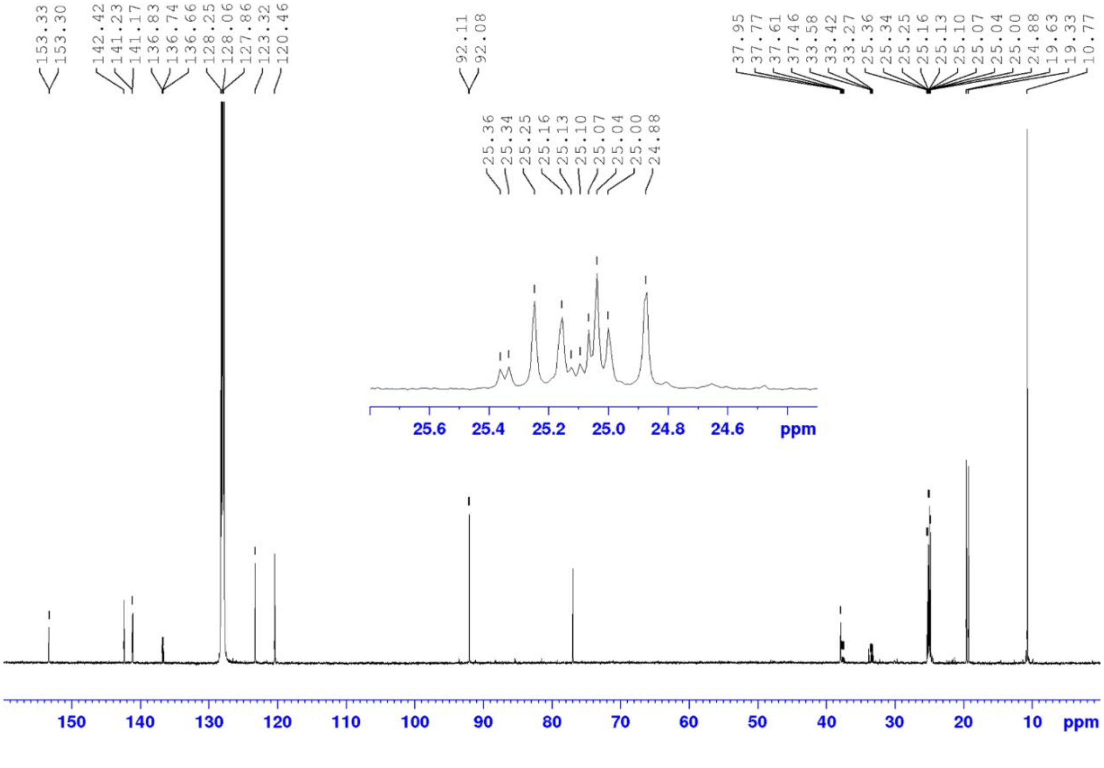

$^{31}P\{^1H\}$ NMR (202 MHz, C_6D_6)

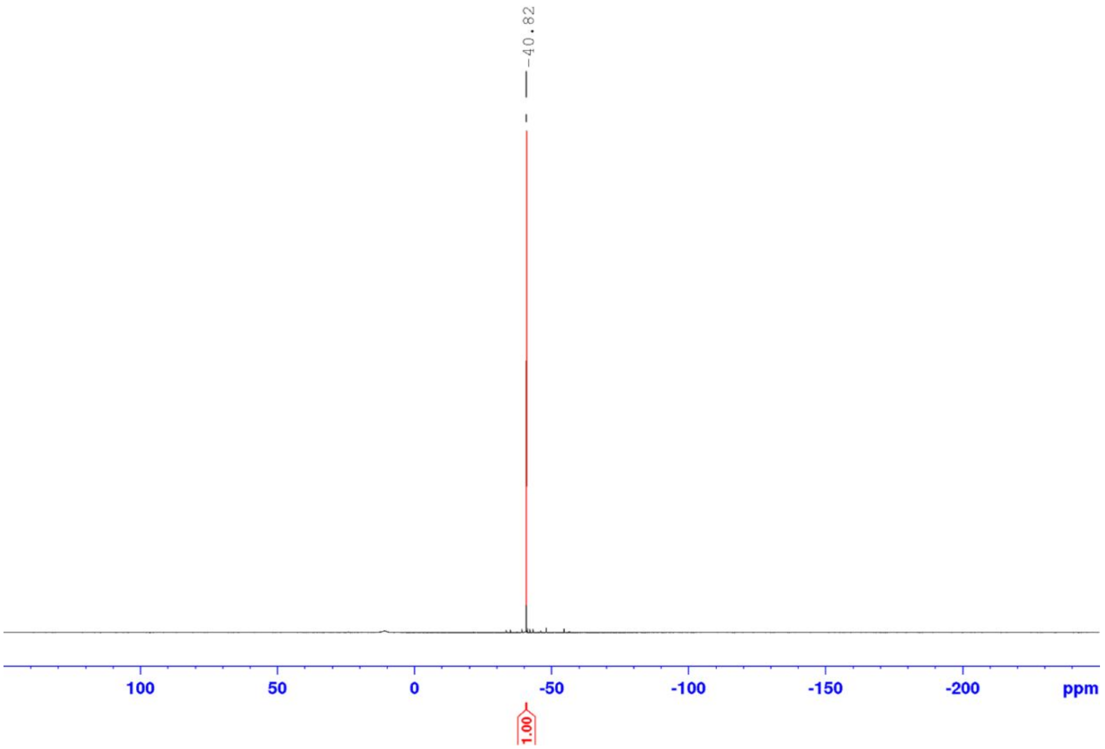

Complex **2l-d4** - [Cp*Ir(PMe$_3$)(H)(2,5-diisopropylphenyl-d$_4$)]

^{1}H NMR (500 MHz, C_6D_6)

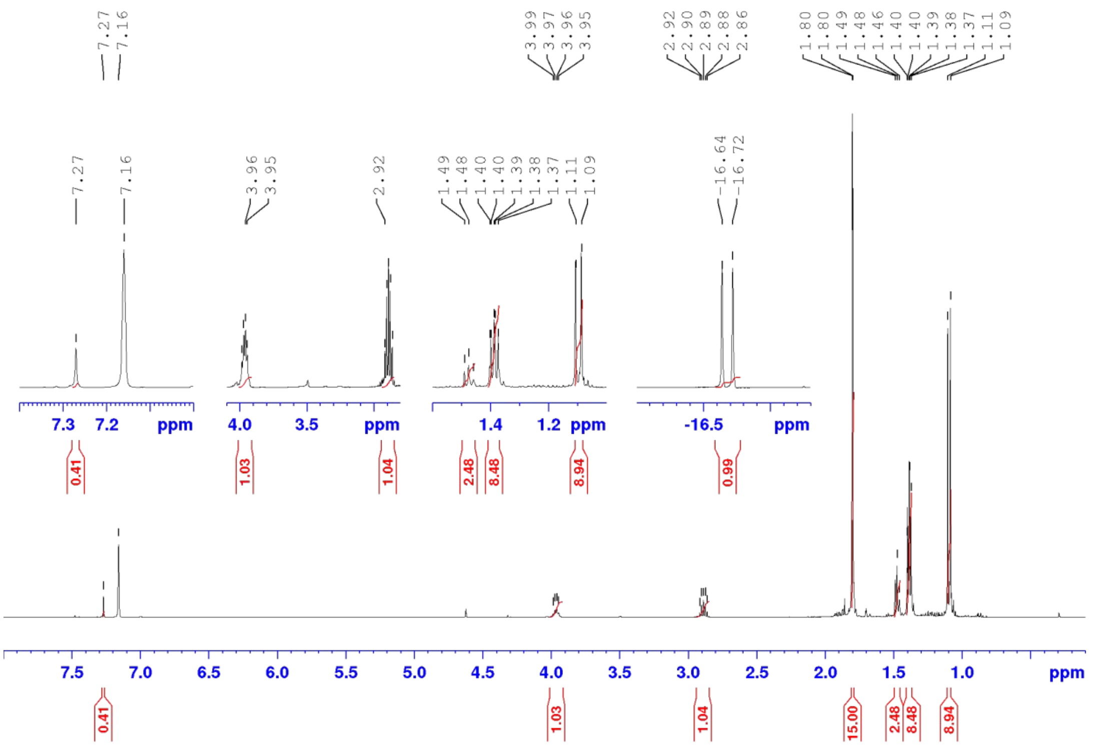

^{13}C{^{1}H} NMR (126 MHz, C_6D_6)

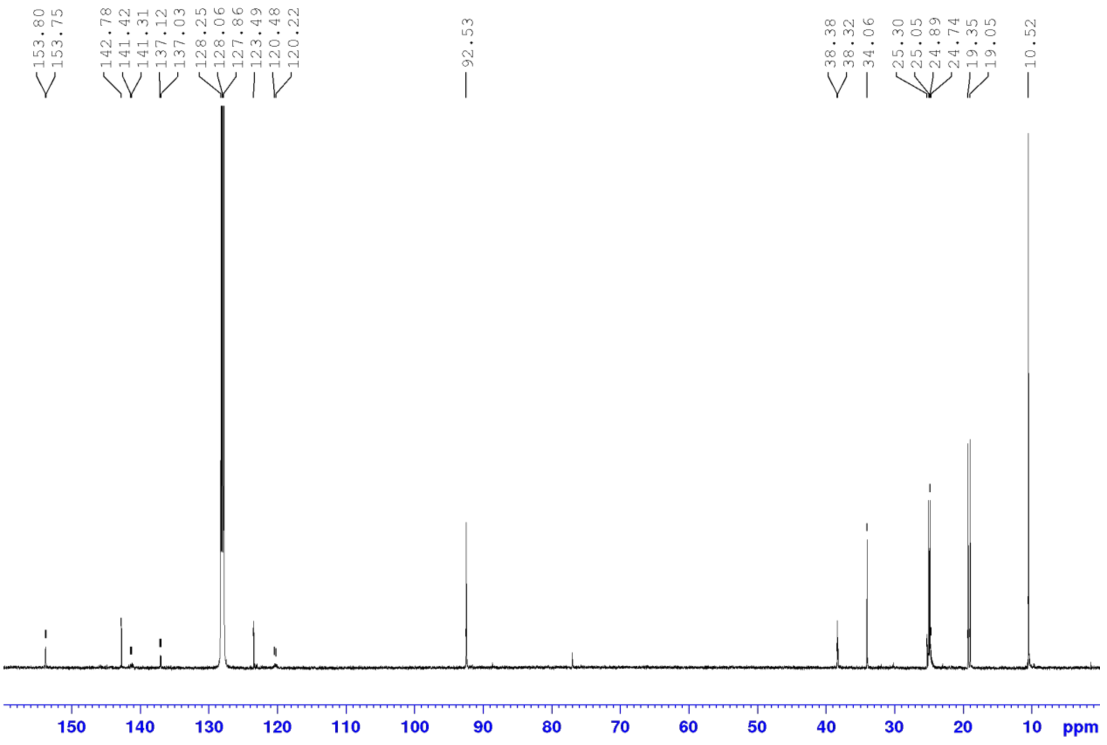

$^{31}P\{^{1}H\}$ NMR (202 MHz, C_6D_6)

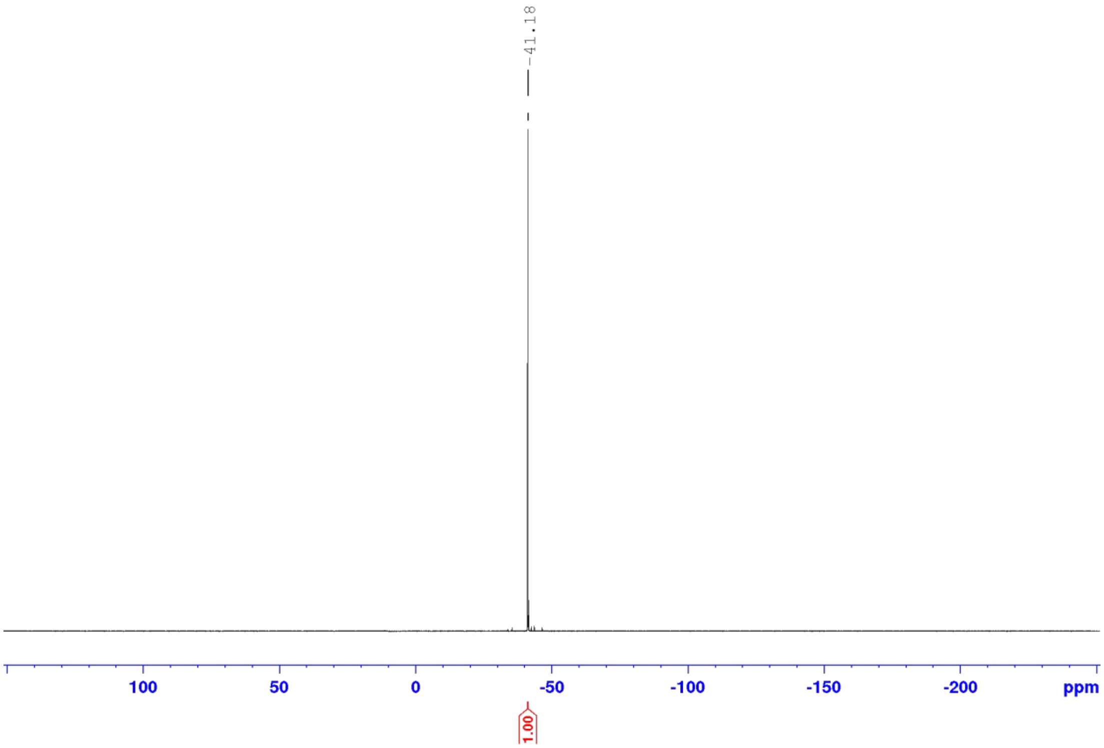

Complex **2m** - [Cp*Ir(PMe_3)(H)(2-^{i}propyl-5-methylphenyl)] and [Cp*Ir(PMe_3)(H)-(4-^{i}propylbenzyl)]

^{1}H NMR (500 MHz, C_6D_6)

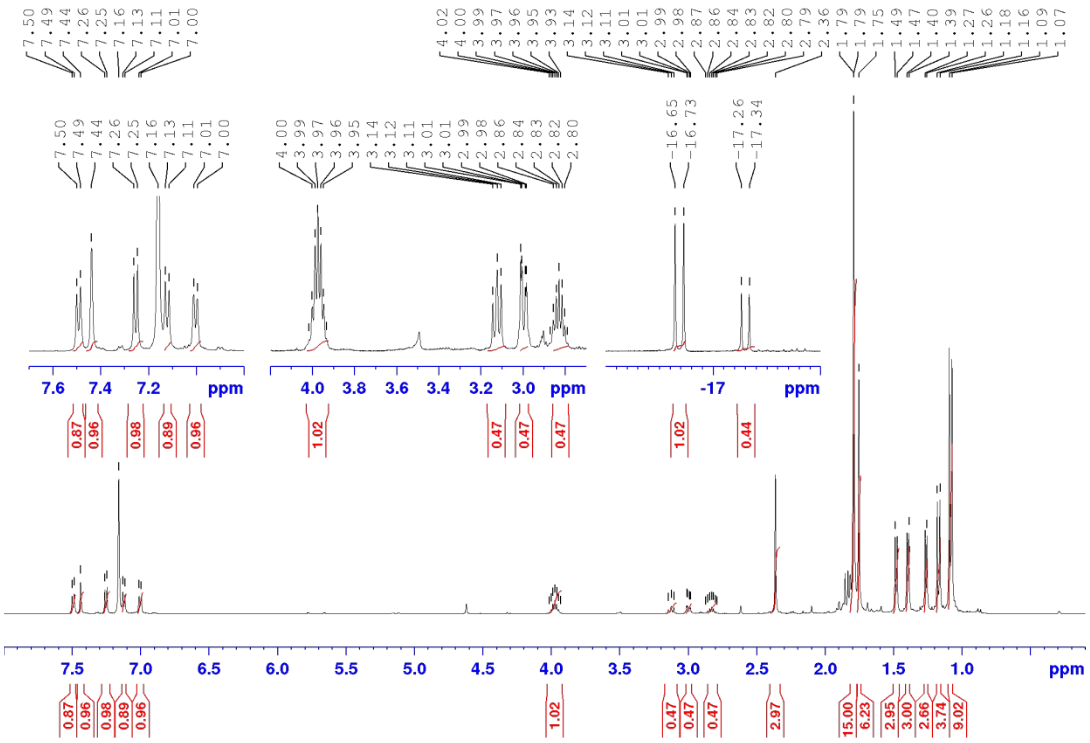

^{13}C{^{1}H} NMR (126 MHz, C_6D_6)

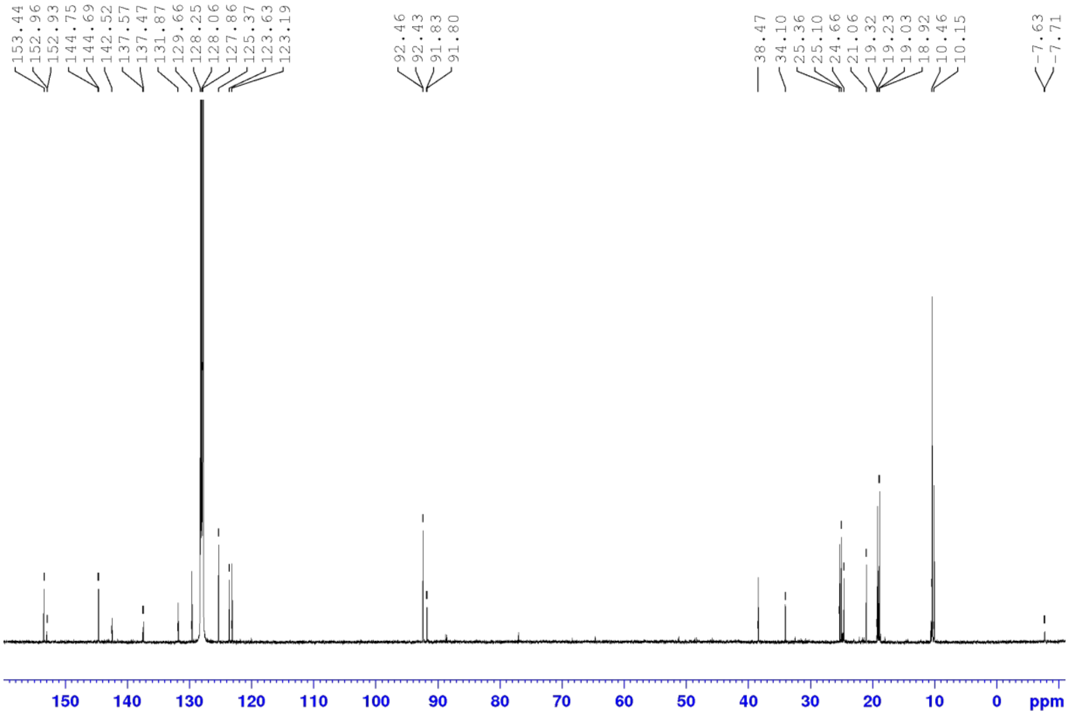

$^{31}P\{^{1}H\}$ NMR (202 MHz, C_6D_6)

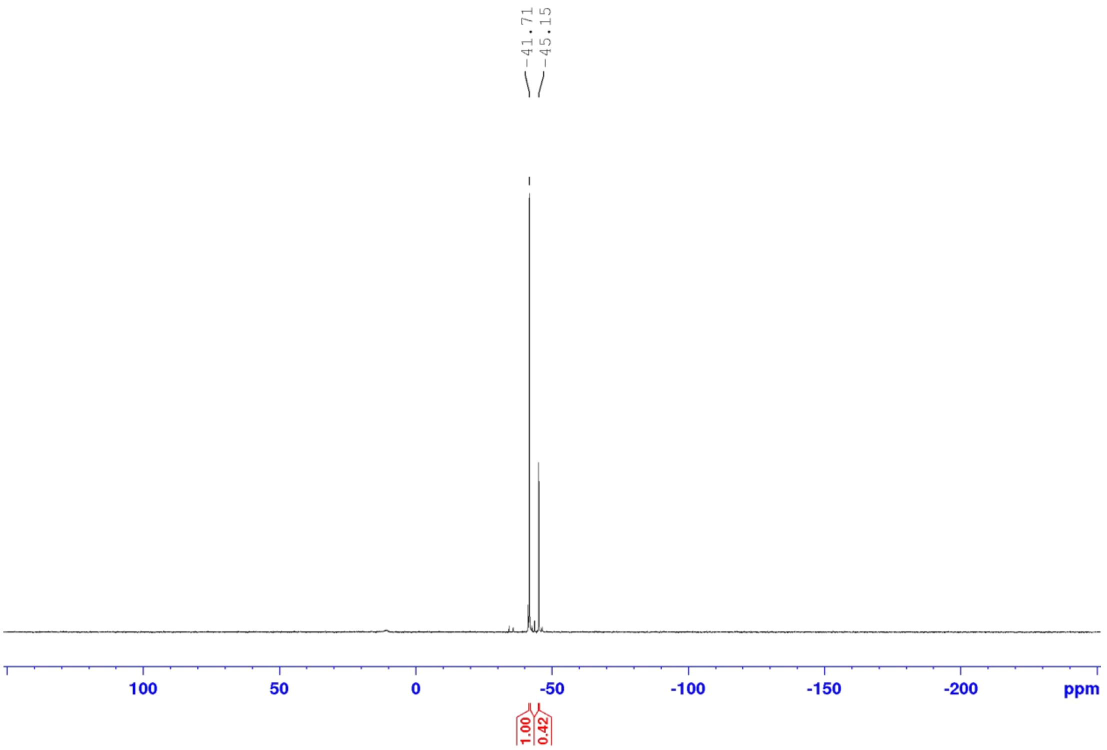

HSQC NMR (500 MHz, C_6D_6)

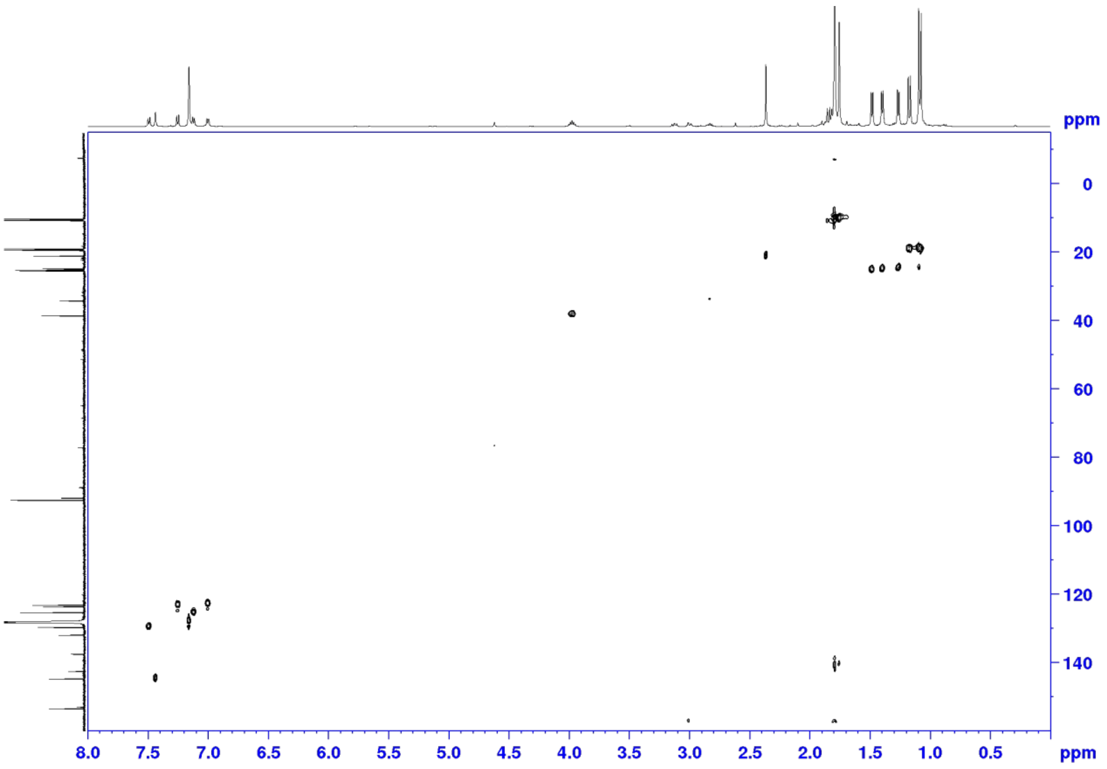

HMBC NMR (500 MHz, C_6D_6)

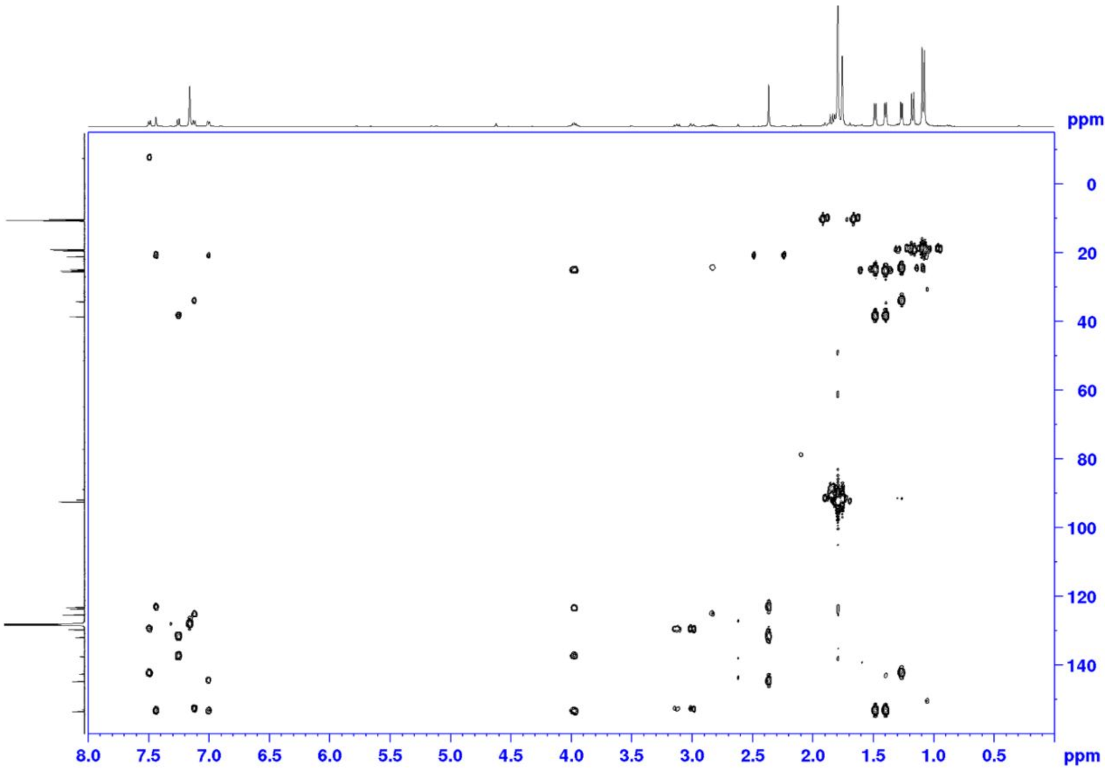

Complex 2a-br - [Cp*Ir(PMe_3)(Br)(2-isopropylphenyl)]

^{1}H NMR (500 MHz, C_6D_6)

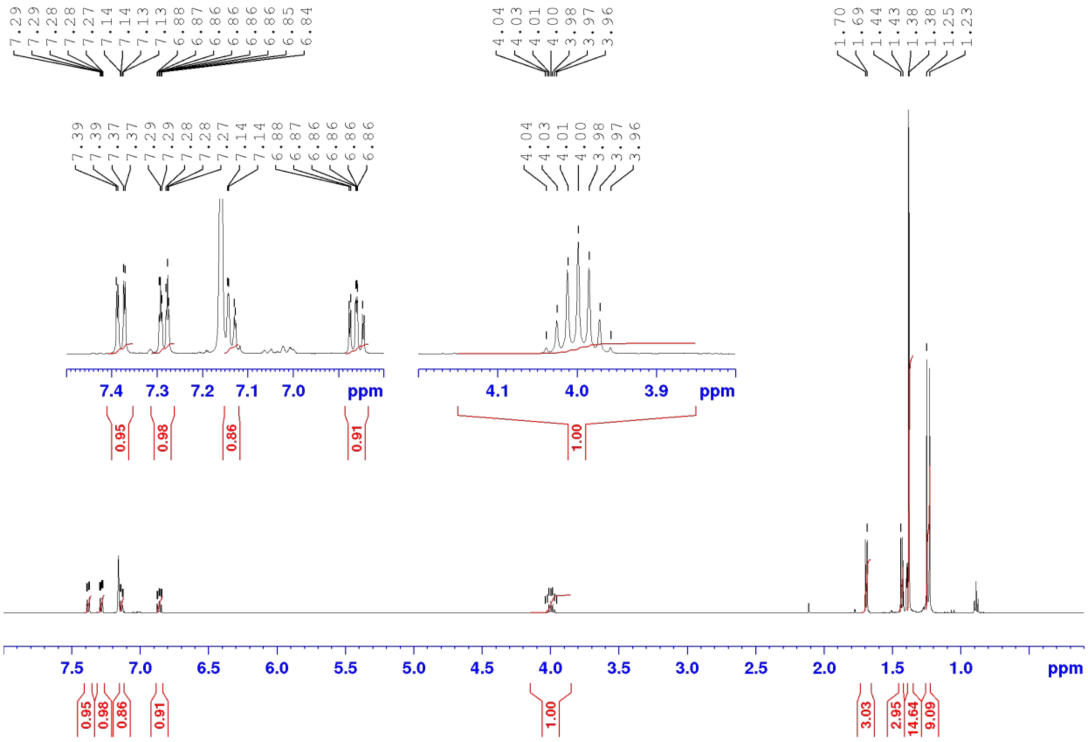

$^{13}C\{^{1}H\}$ NMR (126 MHz, C_6D_6)

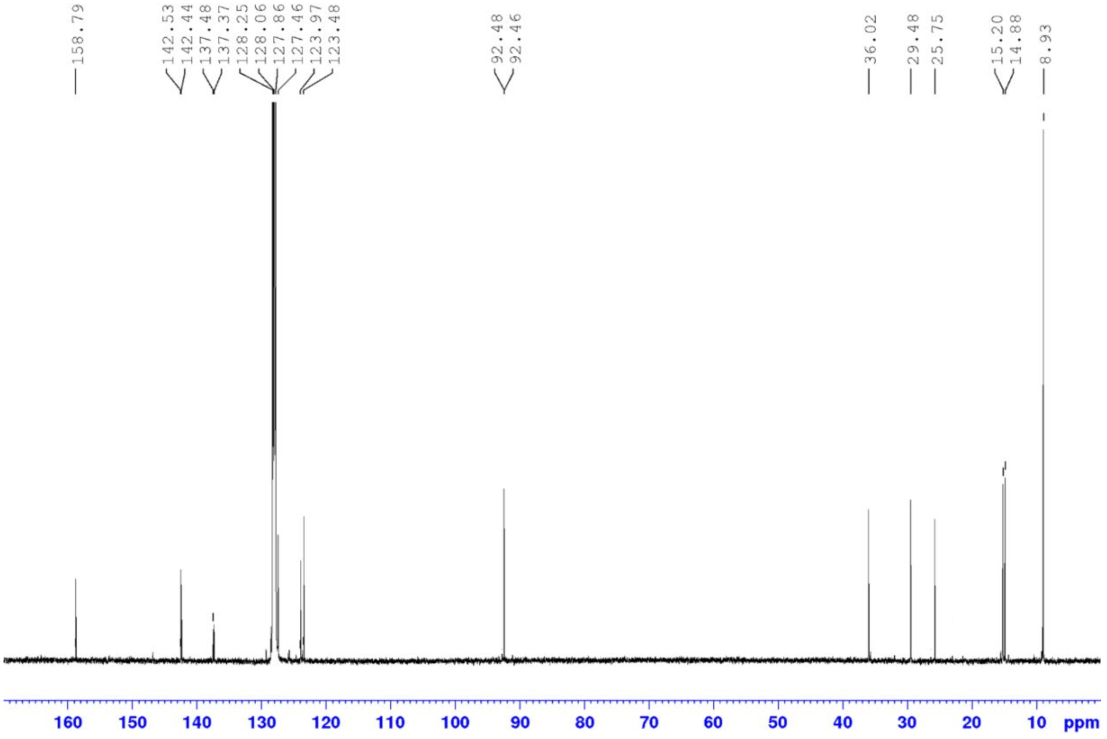

$^{31}P\{^{1}H\}$ NMR (202 MHz, C_6D_6)

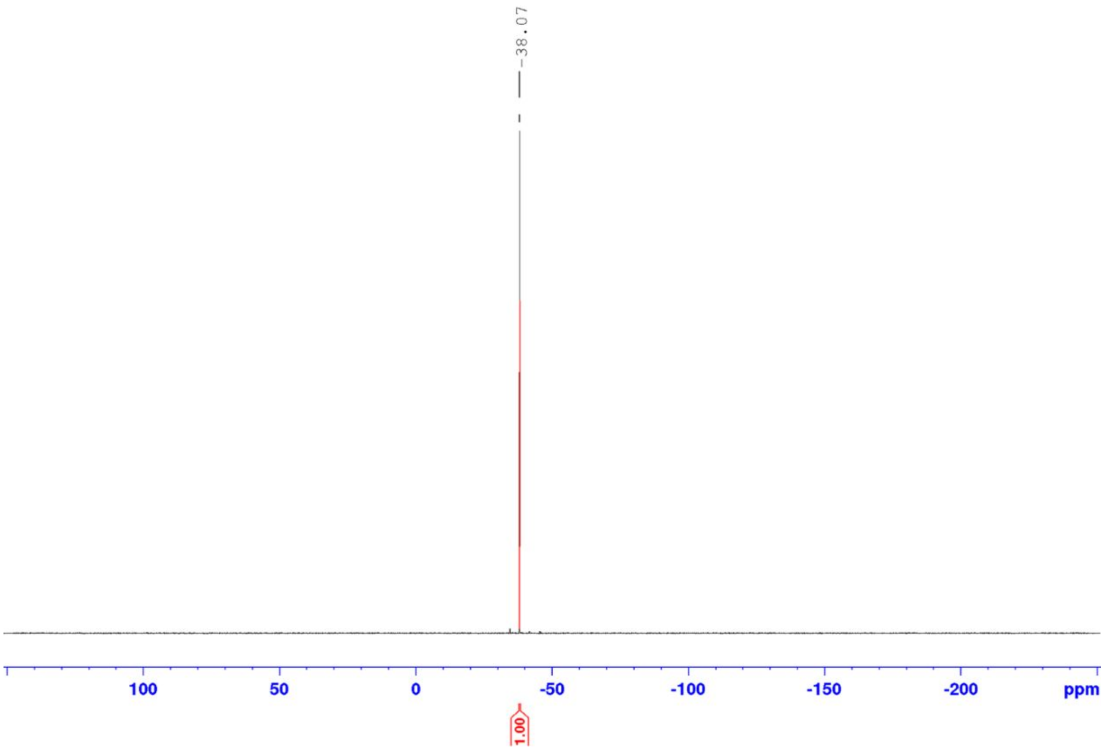

HSQC NMR (500 MHz, C_6D_6)

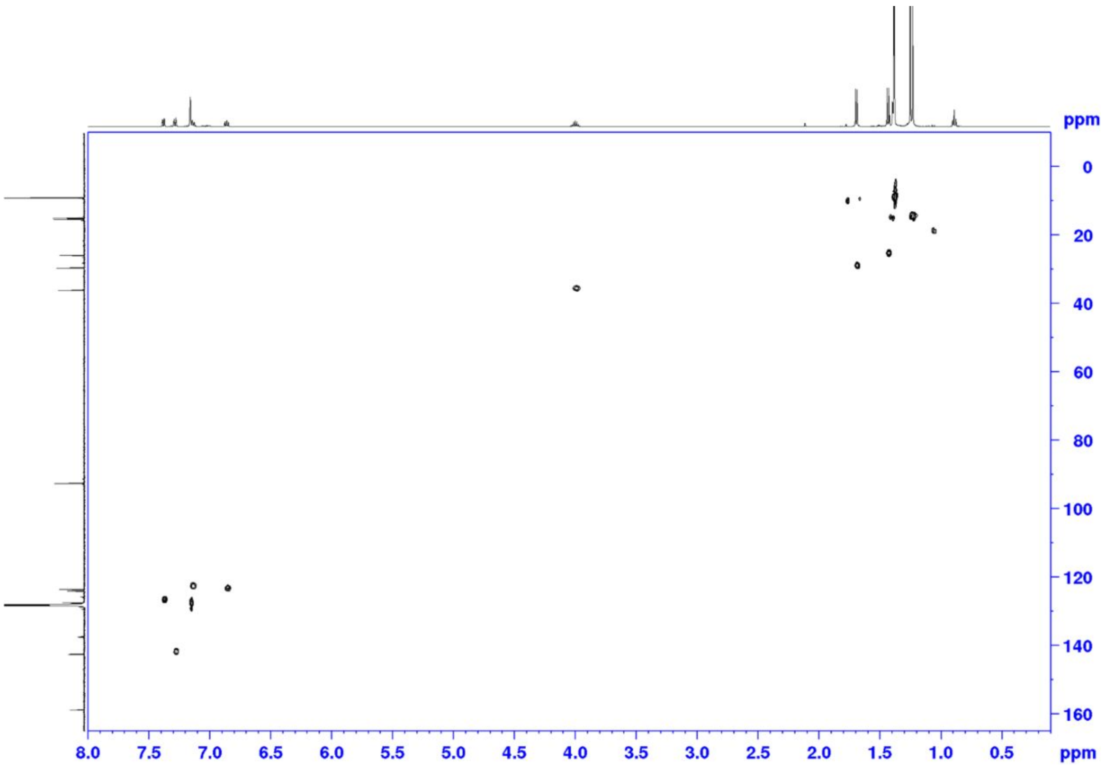

HMBC NMR (500 MHz, C_6D_6)

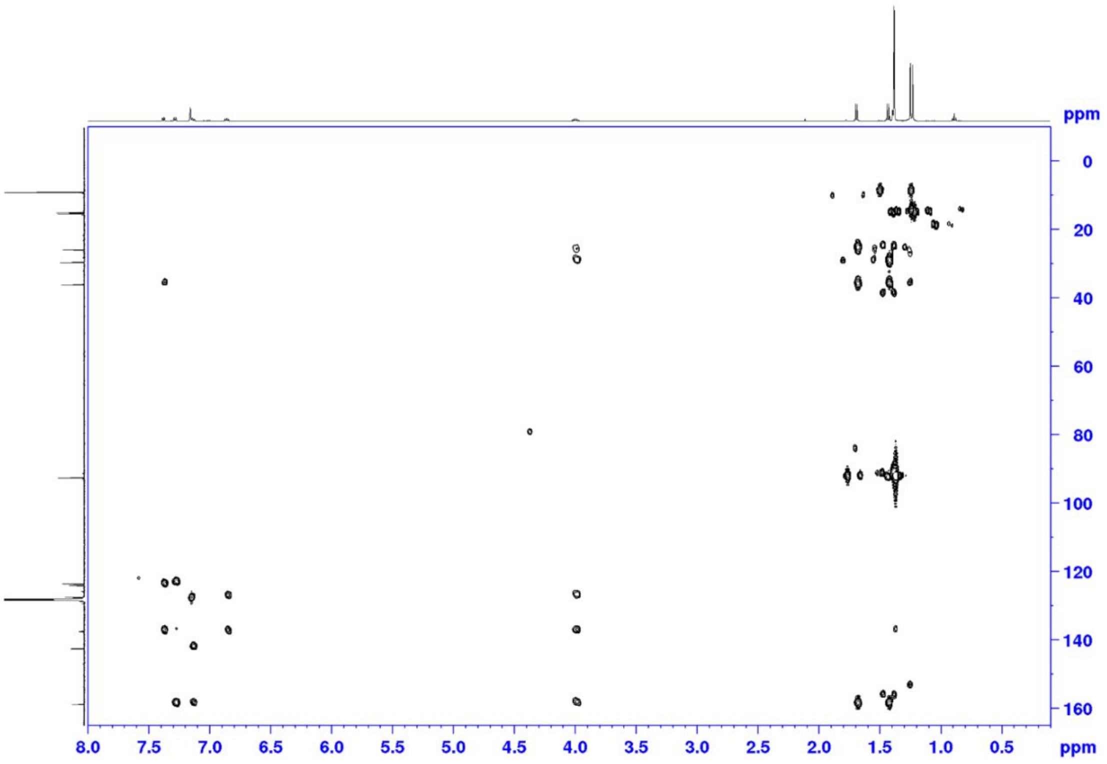

11. References

1. Jakoobi, M., Halcovitch, N., Whitehead, G. F. S. & Sergeev, A. G. Selective arene cleavage by direct insertion of iridium into the aromatic ring. *Angew. Chem. Int. Ed.* **56**, 3266-3269 (2017).
2. Wang, Y. *et al.* Visible-Light-Promoted Site-Specific and Diverse Functionalization of a C(sp^3)–C(sp^3) Bond Adjacent to an Arene. *Acs Catal* **10**, 6603-6612 (2020).
3. Chan, A. P. Y. *et al.* Selective, radical-free activation of benzylic C-H bonds in methylarenes. *Chem. Commun.* **57**, 7894-7897 (2021).
4. Qiao-Xia, G., Bao-Jian, S., Hai-Qing, G. & Tamotsu, T. Aromatic H/D Exchange Reaction Catalyzed by Groups 5 and 6 Metal Chlorides. *Chin. J. Chem.* **23**, 341-344 (2005).
5. Chen, T. S., Wolinska-Mocydlarz, J. & Leitch, L. C. Synthesis of deuteriomethyl aromatic hydrocarbons by exchange with dimethylsulfoxide-d_6. *J. Label. Compd. Radiopharm.* **6**, 285-288 (1970).
6. White, C., Thompson, S. J. & Maitlis, P. M. Pentamethylcyclopentadienyl-Rhodium and "Pentamethylcyclopentadienyl-Iridium Complexes .12. Tris(Solvent) Complexes and Complexes of Eta-6-Benzene, Eta-6-Napthalene, Eta-6-Phenanthrene, Eta-6-Indene, Eta-6-Indole, Eta-6-Fluorene and Eta-5-Indenyl and Eta-5-Indolyl. *J. Chem. Soc. Dalton Trans.*, 1654-1661 (1977).
7. Jeong, H., Joo, K.-S. & Chin, C. S. Iridium(Ⅲ) Complexes of η 6-Arenes with Olefinic and Cyclopropyl Substituents: Facile Conversion to η^3-Phenylallyl Complexes. *Bull. Korean Chem. Soc.* **18**, 402 (1997).
8. Dolomanov, O. V., Bourhis, L. J., Gildea, R. J., Howard, J. A. K. & Puschmann, H. OLEX2: a complete structure solution, refinement and analysis program. *J.Appl. Cryst.* **42**, 339-341 (2009).
9. Sheldrick, G. M. SHELXT - Integrated space-group and crystal-structure determination. *Acta Cryst. A* **71**, 3-8 (2015).
10. Sheldrick, G. M. Crystal structure refinement with SHELXL. *Acta Cryst. C* **71**, 3-8 (2015).
